# ZOOJEU: Using Q fever to highlight a participatory science approach for fostering the co-design of management strategies for neglected zoonotic diseases

**DOI:** 10.1371/journal.pntd.0014618

**Published:** 2026-10-15

**Authors:** Elsa Jourdain, Élodie Rousset, Émilie Ramillien, Patrice Cayre, Xavier Fourt

**Affiliations:** 1 Université Clermont Auvergne, INRAE, VetAgro Sup, UMR EPIA, Saint-Genès Champanelle, France; 2 French Agency for Food, Environmental and Occupational Health and Safety (ANSES), Sophia Antipolis Laboratory, Animal Q Fever Unit, Biot, France; 3 Origens Media Lab, Clermont-Ferrand, France; 4 DGER, Paris, France; 5 Collectif d’artistes Bureau d’Études, Ferme de la Mhotte, Saint Menoux, France; Colorado State University, UNITED STATES OF AMERICA

## Abstract

**Background:**

Decision-making is one of the most difficult aspects of managing neglected zoonotic diseases. Ideally, public health management strategies would account for all stakeholder perspectives. However, in practice, certain stakeholders, notably farmers, are often excluded. ZOOJEU is a participatory science approach designed to tackle this challenge; it uses Q fever in goats in western France as a target system.

**Materials and methods:**

ZOOJEU emerged from a transdisciplinary collaboration among researchers in epidemiology, social anthropology, and the public policy sciences. First, we wrote a comic strip based on earlier anthropological research that uncovered the different realities of stakeholders in a region that recently experienced a human Q fever outbreak. Second, a serious game was built to foster mutual understanding among stakeholders and encourage the collaborative co-designing of public health strategies with farmers. Game construction involved compiling lists of tangible and intangible elements that characterise stakeholder experiences of Q fever outbreaks. Then, the game was played by stakeholders at a workshop.

**Results:**

The comic strip highlighted issues related to Q fever for the stakeholders. The game was played by 23 stakeholders divided into three sets of players; each set of players included at least one farmer. The players were asked to collectively analyse a situation in which a goat farm is experiencing Q-fever-induced abortions. The players were invited to explain their perspectives and actions using game figures representing elements with which different stakeholders engage with regularly and/or in the context of a zoonosis. The goal was to establish a shared vision of what was happening on the farm.

**Conclusion:**

The approach appeared to be effective in establishing a common narrative and fostering a more democratic approach to public health while simultaneously accounting for human, animal, environmental, and social health concerns. Its utility as an educational tool should be explored further.

## Author summary

ZOOJEU is a participatory science approach that invites farmers and other stakeholders involved in livestock farming, human health, and animal health to explore a public health challenge together. The case study is a disease called Q fever, which affects humans and goats. ZOOJEU employs two main tools: a comic strip and a serious game. The comic strip graphically describes the results of anthropological research, which showed that stakeholders have very different experiences of Q fever. The serious game provides a way for stakeholders to discuss how they would experience and deal with a hypothetical Q fever outbreak on a goat farm. The perspectives of farmers are centred because the objective is to foster mutual respect among stakeholders and create space for constructive dialogue around disease management. Indeed, management strategies would gain from being co-constructed with farmers, who possess unique practical experience. We organised a workshop during which 23 stakeholders, organised into 3 groups, played the game. The workshop was successful in that it exposed players to multifaceted viewpoints while ensuring that input from farmers received specific attention. The results suggest that ZOOJEU provides a promising framework for innovatively advancing towards the more democratic management of neglected diseases. The approach also has applications in educational contexts.

## 1. Introduction

The Covid-19 pandemic and other recent sanitary crises have demonstrated the major economic, societal, and public health impacts of managing infectious diseases. Public health crises give rise to a key question: who should be advising decision-makers on the best actions to take? Should it be scientists, whose usual role is to understand the disease and develop tools for disease surveillance and management? Should it be public authorities, whose role is to protect the population from the disease and its consequences? Should it be stakeholders on the ground, such as farmers who interact daily with animal reservoirs or patients who are suffering from the disease? It is challenging yet crucial for public health strategies to account for all these perspectives while also addressing the unbalanced power dynamics that occur among stakeholders. Such is particularly important in the case of neglected and emerging diseases for which there are no specific regulations or dedicated sources of funding. This is the case of Q fever in France.

Q fever is a ruminant-borne zoonosis that causes recurrent outbreaks worldwide, including in Europe [1]. It is a WOAH listed disease regulated in many countries [2]. In the United States, for instance, *C. burnetii* is a Category B biological agent and is considered to potentially pose a severe threat to public health and safety. In contrast, in France, Q fever is not subject to any official regulations, although health authorities recognise that its surveillance and management are crucial to maintain animal and public health [3–5]. Thus, when clusters of human Q fever cases occur, French authorities call upon ‘experts’ to analyse zoonotic risks, recommend lines of investigation, and propose management strategies that may or may not be implemented by decision makers [1]. Local stakeholders may also reach out to ‘experts’ when animal abortions attributed to Q fever are identified in facilities open to the public (e.g., agricultural schools or zoos).

However, Q fever diagnosis and management are complex and remain the focus of extensive ongoing research on both human and animal health [6–9]. Such existing gaps in knowledge can make it difficult to assess the actual risks associated with *C. burnetii*, which means experts can struggle to come up with recommendations for management strategies [10,11]. The complexity of this situation is illustrated by the successive French scientific opinions and regulatory texts focused on *C. burnetii* [5,12–19] and by the diversity of public policies that have been implemented in different countries [20,21]. Overall, expertise gains greatly in relevance if it is constantly evaluated, challenged, and tailored to align with stakeholder realities and if it is not biased by conflicts of interest [22–24]. The stakeholders include not only veterinarians, human health specialists and risk managers, but also and above all, livestock farmers.

When livestock farmers were asked their opinion about routine recommendations for Q fever management, they expressed that recommendations are sometimes, if not often, too disconnected from their lived reality [25]. The fact is that farmers are rarely represented in discussions between ‘experts’ and health authorities, meaning the resulting recommendations may not be pragmatic or relevant. When public authorities see the lack of compliance with their recommendations, they tend to assume that farmers lack the requisite knowledge or have a poor understanding of the health risks, and their response is to create communication campaigns aimed at “correcting” these issues [26]. However, the reasons why farmers do not apply the authorities’ recommendations are more often related to intrinsic constraints in the field, of which the ‘experts,’ who are not farmers, are unaware [26]. Livestock farmers possess unique expertise [27] that they have cultivated through their daily interactions with communities of diverse species, including plant, animal [28], and microbial species [29]. They have gathered practical empirical knowledge about their animals and their working environment [30], which is critical to understanding the transmission dynamics of diseases such as Q fever [25]. Their daily observations, their experience with animal behaviour, and their awareness of subtle changes in farming conditions can provide valuable insights that are likely to be overlooked by academics or medical experts who are less familiar with realities in the field. Because of the nature of their work, farmers are automatically involved in zoonotic disease management, which makes them public health stakeholders, even if they do not have an official mandate to deal with public health matters.

We propose that respecting and valuing all forms of expertise, whether academic or empirical, is essential if we wish to effectively address the complex challenges of neglected or emerging zoonotic diseases. More specifically, we hypothesise that incorporating the professional expertise of farmers can enrich scientific findings with practical perspectives and help co-design management strategies that are more practical and aligned with realities on the ground. We consider that the first necessary step is to develop mutual understanding and respect among stakeholders. As a case study, we explored the situation of a goat farm experiencing Q-fever-induced abortions in an area where a cluster of Q fever cases had recently emerged. Our aim was to foster mutual understanding and respect among stakeholders and examine how Q fever management strategies could benefit from being co-designed with farmers. We thus developed a mediation approach that we successfully tested with the relevant stakeholders.

## 2. Methods

### 2.1 Ethics statement

The protocol was approved by the Data Protection Board of the National Research Institute for Agriculture, Food and Environment (Institut national de recherche pour l’agriculture, l’alimentation et l’environnement – INRAE) under approval number TRT-983, which ensured that the study was carried out in accordance with French General Data Protection Regulation (GDPR) principles. All study participants provided formal written informed consent. Recruitment of participants for the multistakeholder workshop of the serious game started on April 17, 2019 and ended on April 24, 2023.

### 2.2 Study site

We focused on the area in and around the Niort basin, a region in western France with a high density of goat farms [31] and a shortage of both medical and veterinary professionals. Our research took place after a Q fever outbreak, whose exact source remained unidentified despite intensive medical and veterinary efforts [32]. This outbreak occurred between April 15 and June 13, 2017, and involved 11 cases confirmed by the National Reference Centre for human Q fever [1,32]. It motivated the implementation of extensive epidemiological and anthropological research on Q fever as part of a research project called EXPAIRCOX [33].

### 2.3 Conceptual and methodological framework

Initial discussions regarding our project took place in 2018, during a symposium that invited the reinvention of research and other forms of inquiry through collaborations among artists, academics, and the general public [34]. The symposium was an opportunity to explore the challenges that we encounter in our respective scientific disciplines (veterinary public health and epidemiology, microbiology, anthropology, pragmatic sociology, and public health design). Starting from the observation that Q fever “experts” often feel helpless and incapable of action when confronted with the complex realities in the field, we conducted anthropological research to explore stakeholders’ different experiences of Q fever, outside of any pre-existing power dynamics. The result was data generated with a pragmatic approach, in which the narratives of research subjects are viewed as truth [35]. We used these data to better understand how farmers and other stakeholders see the world, what entities they pay attention to, and how they interpret them—a different perspective than the one emerging from scientific rationality [36]. We disseminated our findings at seminars and conferences for health professionals, epidemiologists, and anthropologists; we published them in scientific journals [25,37] and in a newspaper for farmers [38]. Yet, we found ourselves unequipped to clearly and more broadly disseminate the results of this work to stakeholders and help them become familiar with each other’s realities. Thus, we considered that it was essential to experimentally connect and align these diverse forms of knowledge and expertise.

To this end, we used public policy design, a scientific approach that facilitates the preparation, simulation, conception, and prototyping of novel forms of public action; it draws upon the findings of researchers while accounting for the perspectives of relevant stakeholders [39–42]. Public policy design balances the perspective of experts, who have specialised knowledge in a given field, and non-experts, who have a wealth of experience and in-depth locally acquired knowledge, with a view to revealing decision-making biases and tackling what design theorist Horst Rittel called “wicked problems” [43,44]. The latter can be addressed using a dispositive (*dispositif*), defined by Rittel as an assemblage of material or immaterial objects, forms of discourse, institutions, forms of architecture, regulatory decisions, laws, administrative measures, and scientific statements [45]. Designed to serve as a tool for scientific and technical democracy [46], a dispositive must allow the systematic consideration of the issues, practices, and entities with which each stakeholder is associated, allowing for any contradictions or incompatibilities between the three. It may make use of boundary objects, which are representations of concrete objects that represent dependencies among stakeholders and that can be transformed by all of them [47,48].

We created a dispositive named ZOOJEU, which has a dual purpose. First, it seeks to establish a common narrative for stakeholders who do not know each other and who experience different realities as they face Q fever. Second, it aims to highlight the multiplicity of objects that shape the various realities experienced by stakeholders and treat them with actual regard, allowing stakeholders to deepen their collaborative exploration and engage in dialogue. To achieve these goals, we created two complementary support tools, which were to be displayed one after the other. The first is a narrative expressed in the form of a comic strip that attempted to reveal and depict different stakeholder realities. The second is a serious game developed to help stakeholders engage in mutual dialogue and collectively experiment with scenarios involving practical action. We then tested out these tools by inviting a panel of stakeholders to use them and encouraged their use in different pedagogical contexts. All stages of the research process were observed by a third-party monitor from the Directorate for Policy Support (DAPP) at the French National Research Institute for Agriculture, Food, and Environment (INRAE). They provided external feedback on the project’s design and implementation.

### 2.4 Data

The data were first-hand accounts from stakeholders and the description of the entities, practices, and mechanisms that had been identified during previous research and in public action reports.

#### 2.4.1 Anthropological data.

We used the results of the anthropological research by Ramillien *et al.* [25], which was conducted within the framework of the EXPAIRCOX project. These data were presented as ideal types, which Weber [49] has defined as forms of storytelling that accentuate certain character traits to underscore differences. In each ideal type, stakeholders have a specific arrangement with or attachment to entities (human or non-human, material or immaterial). The ideal types were established using information on what stakeholders said and did and their underlying reasons, while also paying attention to how stakeholders were interacting with microbes, including with *C. burnetii*, during their daily practices. A main finding was that livestock farmers viewed themselves as straddling two “worlds” that represent co-existing realities: in World A, cooperation and care for living organisms predominate whereas, in World B, management and control of living organisms predominate [25]. The other main finding was that livestock farmers perceive the health situation of their farms very differently depending on how much they delegate health risk management to other stakeholders (e.g., veterinarians, goat feed advisers, dairy inspector). This situation is somewhat specific of the goat farming sector, given the low economic value of an individual goat: the cost of a veterinary appointment (€80) far exceeds the market value of the goat (€8–10), meaning that farmers end up managing their animals’ health issues, especially when they have moderate-sized herds. Farmers relying heavily on delegation often perceived Q fever as a somewhat elusive and threatening disease; they also tended to report that their work lacked meaning and a general feeling of restlessness in their lives [25]. The results of this research revealed that an individual’s relationship with Q fever varies depending on that individual’s lived reality [25].

#### 2.4.2 Data on public health strategies.

To explore how public health strategies are implemented within administrative bodies and on farms, we used data from documents containing health management recommendations for French livestock farmers, which emphasise the importance of biosecurity practices (e.g.[50,51],). They essentially adopt the approach of educating farmers about biosecurity practices and promoting their usage. We also used data from reports describing the organisational landscape of Q fever management in France that we had commissioned for this purpose [52,53]. Finally, we used data from the official report describing the recent Q fever outbreak in the Niort urban area [32].

### 2.5. Dispositive design

We produced the materials that make up the ZOOJEU dispositive (the comic strip and serious game) as well as materials that are intended to facilitate ZOOJEU’s implementation during workshops (an animated video, a scientific presentation, brochure, and posters). Because the creative process was transdisciplinary, we invested considerable time in becoming mutually acquainted with each other’s discipline-specific languages, approaches, and codes. From a methodological perspective, we organised working seminars interspersed with repeated exchanges around preliminary drafts. Fig 1 depicts the overall analytic framework. The process took two years overall.

**Fig 1:**
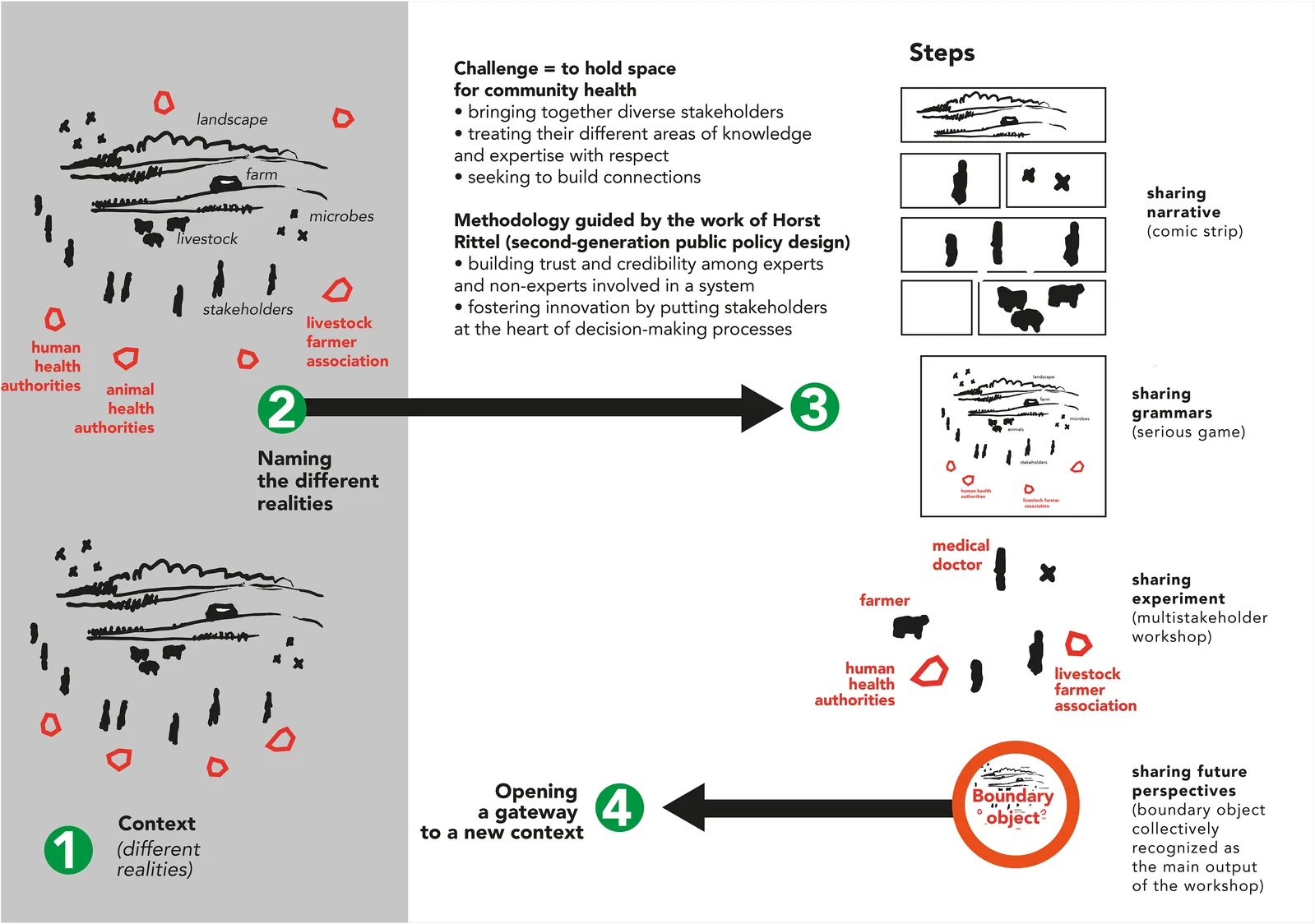
Flowchart describing the analytic framework of the ZOOJEU approach. The manuscript focuses on the third point outlined by the flowchart, which is divided into three steps: sharing a narrative (comic strip), sharing grammar (serious game), and sharing experiment (multistakeholder workshop); the later results in new shared perspectives.

#### 2.5.1 Comic strip.

It is said that a picture is worth a thousand words, so we created a comic strip to draw the attention of stakeholders to the issues related to Q fever that arose from the anthropological research. The idea was to highlight different stakeholder realities as a first step in establishing a shared narrative. We expected that using a comic strip would help enhance the impact of the messages by engaging with visual thinking, which brings both the rational brain and the emotional brain on board [54]. We also expected that pictures would introduce greater symmetry into stakeholder dynamics, as per Isabelle Stengers [55]: the pictures could better balance power between “those who know” (the “experts,” who are mandated to prescribe and control) and the “others”, who are supposedly ignorant and have to follow what is prescribed to them. Overall, we sought to highlight the diversity and interconnectedness of realities and stakeholders as well as to reveal administrative intricacies. From a methodological perspective, we first wrote a synopsis, using verbatim quotes from first-hand accounts obtained during previous research and reports; we then gradually added a pictorial narrative.

#### 2.5.2 Serious game.

We designed the serious game as a tool that allows players to collectively face a hypothetical Q fever outbreak on a goat farm and explore potential management strategies. We began the game creation process by compiling lists of tangible and intangible objects (whether existent or non-existent) that characterise relevant stakeholders, including farmers, public health authorities, veterinarians, medical practitioners, agricultural advisors, patients, and researchers. We thoroughly considered which boundary objects to use because they would offer a lens through which to examine interrelated perspectives and issues related to public health, economics, family, labour, regulations, technology, and more. To this end, we first created maps of the many objects identified. Then, we selected the objects to incorporate into the serious game and built the narrative that would guide object use by participants. Additionally, we considered object size to be a key issue because physical engagement is a critical part of the mediation process [47]. Ultimately, we made wooden stands and 3D game figures made out of cardboard; we had a plastic game mat printed for participants to play on. Three game sets were built to allow for three independent sets of players to play at once. A game set was designed to be played by a set of 7–9 players, which must include at least one farmer; the other players can be professionals working in human, animal, or environmental health, expert patients, everyday citizens, elected officials, or students.

#### 2.5.3 Animated video, presentation, brochure, and posters.

We created a brief animated video using the comic strip illustrations to provide an accessible, narrative introduction to the multistakeholder workshop of the serious game and to the ZOOJEU dispositive. The video was produced using Filmora (Wondershare): we created sequences of the comic strip’s images and recorded the voice-over narration directly within the software. In addition, we prepared a presentation for use during the workshop. The presentation aimed to introduce the transdisciplinary research team, share feedback on the epidemiological and anthropological research carried out as part of the EXPAIRCOX project, present the ZOOJEU dispositive, and formally thank all project participants. We also designed an informational brochure to describe the serious game and to guide the participants through the game’s stages. Finally, we created a set of three posters to serve as visual supports when presenting the ZOOJEU dispositive for communication or pedagogical purposes.

### 2.6. Multistakeholder workshop

We scheduled a full-day experimentation event during which participants representing different stakeholder groups played the ZOOJEU serious game. We qualitatively evaluated the participatory process and project outcomes using a self-evaluation guide developed for participatory science programmes [56]. The latter suggests using a combination of indicators—related to project monitoring, performance, audience characterisation, and impact—that fit with the project’s objectives and context.

## 3. Results

### 3.1. ZOOJEU dispositive

We developed a graphic language that could convey the story of the appearance of Q fever and the stakeholders involved, drawing upon the results of the published anthropological research (Fig 2). An original version of the comic strip, in French, was sent to the stakeholders who participated in the test of the serious game. A second version with minor modifications was later produced to better suit educational contexts; this version was translated into English (see S1 File). The frame-by-frame animated video created to help people follow the comic strip’s storyline in a smooth and engaging way is also available in supplementary materials (see S1 Video). The serious game was conceived for an estimated playing time of 2–3 hours. Each game set includes the following materials: a vertical wooden board, which is used to depict the characteristics of the farm chosen to serve as a case study for the game session (see Fig 3a); four identical horizontal boards in series (called A, B, C, or D) that represent the general background of a goat farm at a different stage of the game (see Figs 3b and 4); and 3D cardboard game figures that the players are invited to place on the boards throughout the game session (see Fig 5). These figures represent elements with which different stakeholders engage regularly and/or in the context of a zoonosis; these elements range from farm buildings to regulatory texts, bacteria to disinfectants, and human health authorities to animal health authorities. The elements are also depicted on vertical wooden boards for the players’ ease of reference (see Fig 4). The game is played over four successive stages, which are detailed in S2 File as well as in Figs 3 and 4.

**Fig 2:**
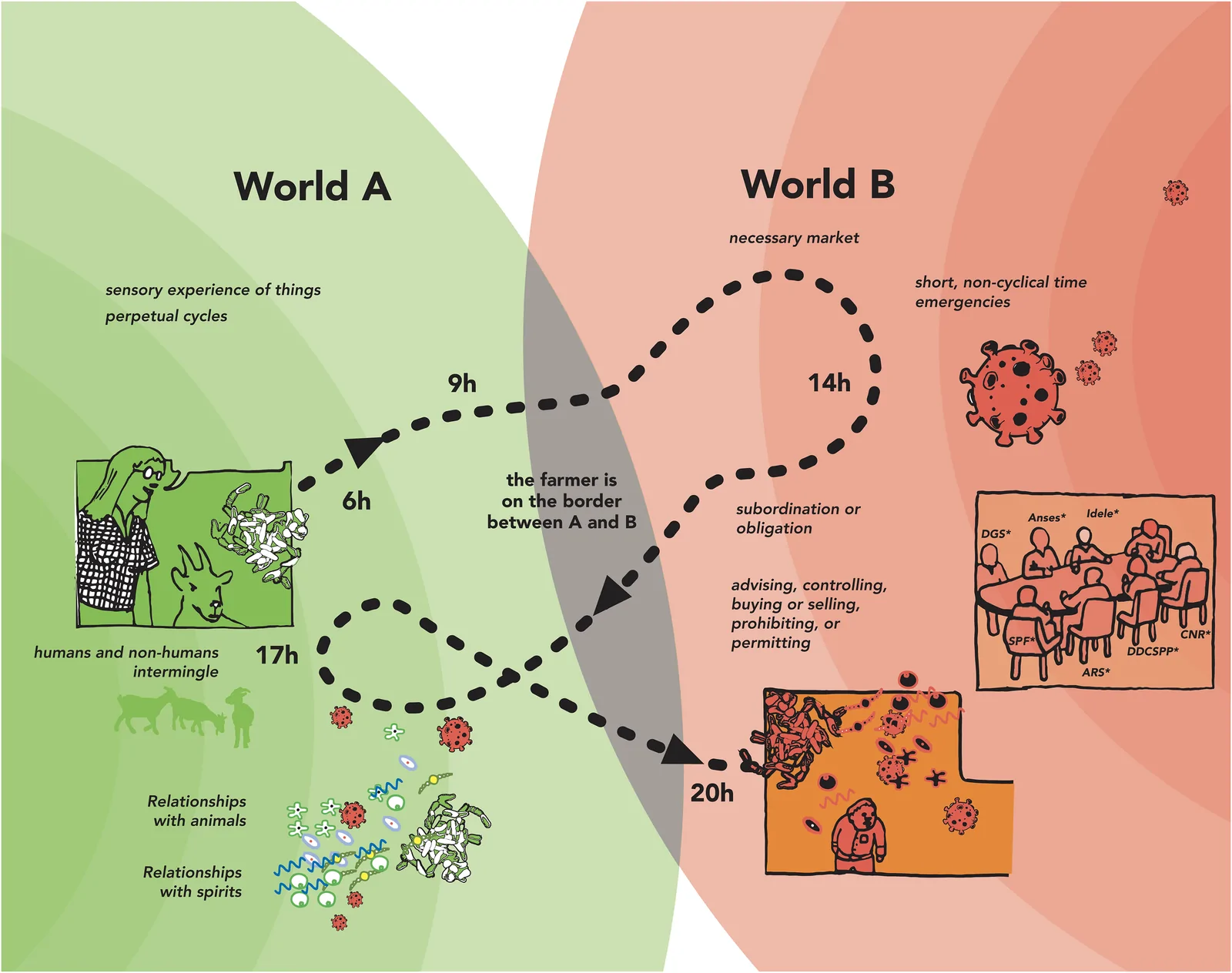
Graphical depiction of the two ‘worlds’ that livestock farmers straddle. World A and World B correspond to the ideal types described by Ramillien *et al*. [25] that represent co-existing realities. In World A, reality is made up of long timeframes and perpetual cycles, such as seasons or reproductive cycles. What predominates is the sensory experience of things that have always existed, like animals, meadows, microbes, and death. In this world, humans and non-humans intermingle and have complex and challenging relationships that are often experienced as authentic and nurturing. The causes of the events that disturb the world’s various cycles are obscure and complex. In World B, reality is essentially based on dualistic distinctions between humans and non-humans, nature and culture, objectification and subjectivity. It is made up of short, non-cyclical timeframes, punctuated by emergencies (e.g., disease outbreaks, necessary market adaptations, or sudden climatic events). Relationships in this world are mostly governed by subordination or obligation, such as advising, controlling, buying or selling, prohibiting, or permitting. Livestock farmers straddle both worlds daily. *DGS: *Direction Générale de la Santé* (a directorate-general of the French Ministry of Health); Anses: *Agence nationale de sécurité sanitaire de l’alimentation, de l’environnement et du travail* (French Agency for Food, Environmental and Occupational Health & Safety); Idele: *Institut de l’Elevage* (French Livestock Institute); SPF: *Santé Publique France* (French National Public Health Agency); ARS: *Agence Régionale de Santé* (Regional Health Agency); DDCSPP: *Direction Départementale de la Cohésion et de la Protection des Populations* (Departmental Directorate for Population Cohesion and Protection); and CNR: *Centre National de Référence de la Fièvre Q* (National Reference Centre for Human Q fever).

**Fig 3:**
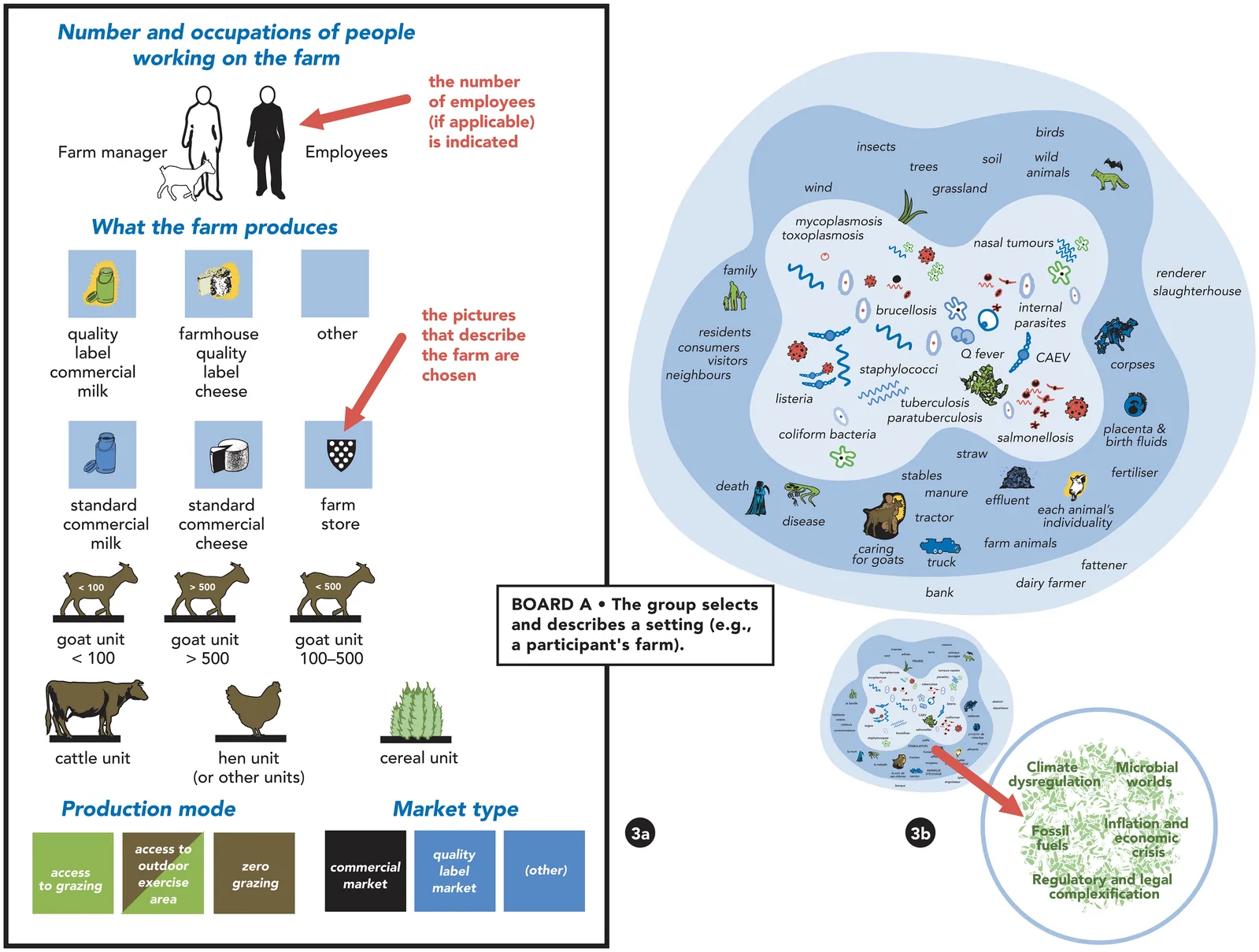
Set-up during the first stage of the game. (3a) Board describing the farm. The farmer playing the game should select the images that best describe their farm: the number of people working on the farm; what the farm produces; the farm’s production methods; and the market(s) for the farm’s products. (3b) General setting for a goat farm. This setting is printed on a long plastic mat (8 x 2 m) designed to cover eight standard classroom tables: it constitutes four game boards (Board A, B, C, or D), which represent the general setting for a goat farm at different stages of the game (see Fig 2). Each board is made of two superposed components. First, there is a 1-metre-diameter circle printed on the mat and repeated four times in sequence, which illustrates the immutable general conditions on the farm that affect everyone, such as climate change, inflation, economic crises, fossil fuels, regulatory and legal complexities, and microbes. Second, there is a 2-metre-large potato-shaped plastic circle that is separate from the mat, which illustrates the various elements that make up everyday life on the farm, such as technical tools, means of production, social and economic organisations, microbes, neighbours, and bank loans. The description is intentionally left incomplete and can be filled in by the players, who are invited to highlight what is important to them. As indicated by the red arrow, the moveable plastic circles representing the elements of everyday day are to be placed on top of the printed circles describing the farm’s general conditions.

**Fig 4:**
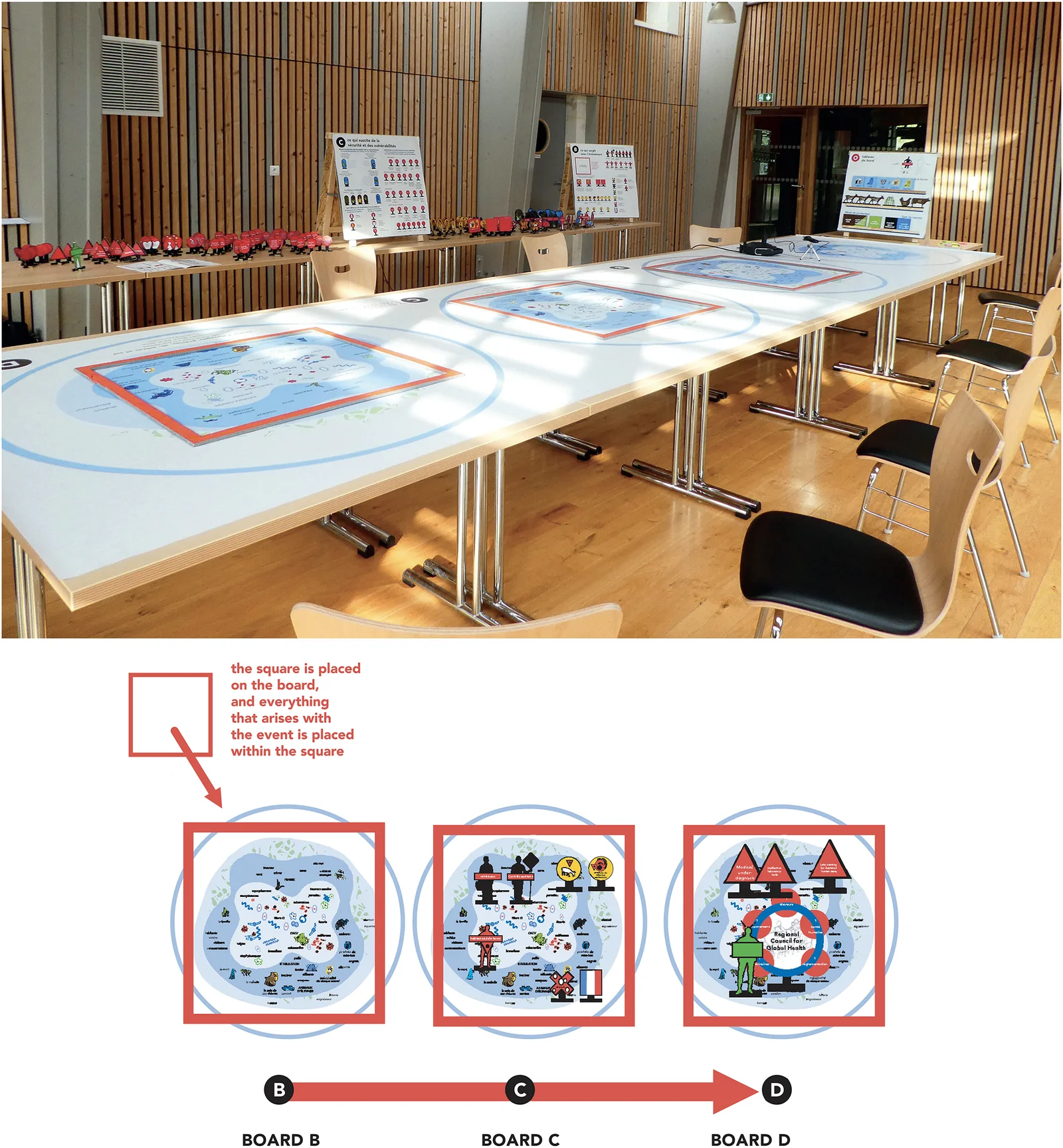
Overall view of the game boards and figures and description of the game stages starting from the outbreak of abortions. The red square is placed on Boards B, C and D to symbolise that there are on the farm an abnormally high number of abortions over a one-week period. The players are then invited to select cardboard game figures representing objects, stakeholders, and practices from among the figures. They focus successively on the following issues: on Board B, what plays key roles for them on the farm in the situation; on Board C, what creates feelings of security or vulnerability; and on Bord D, current health policy failures and potential changes that can be envisioned.

**Fig 5:**
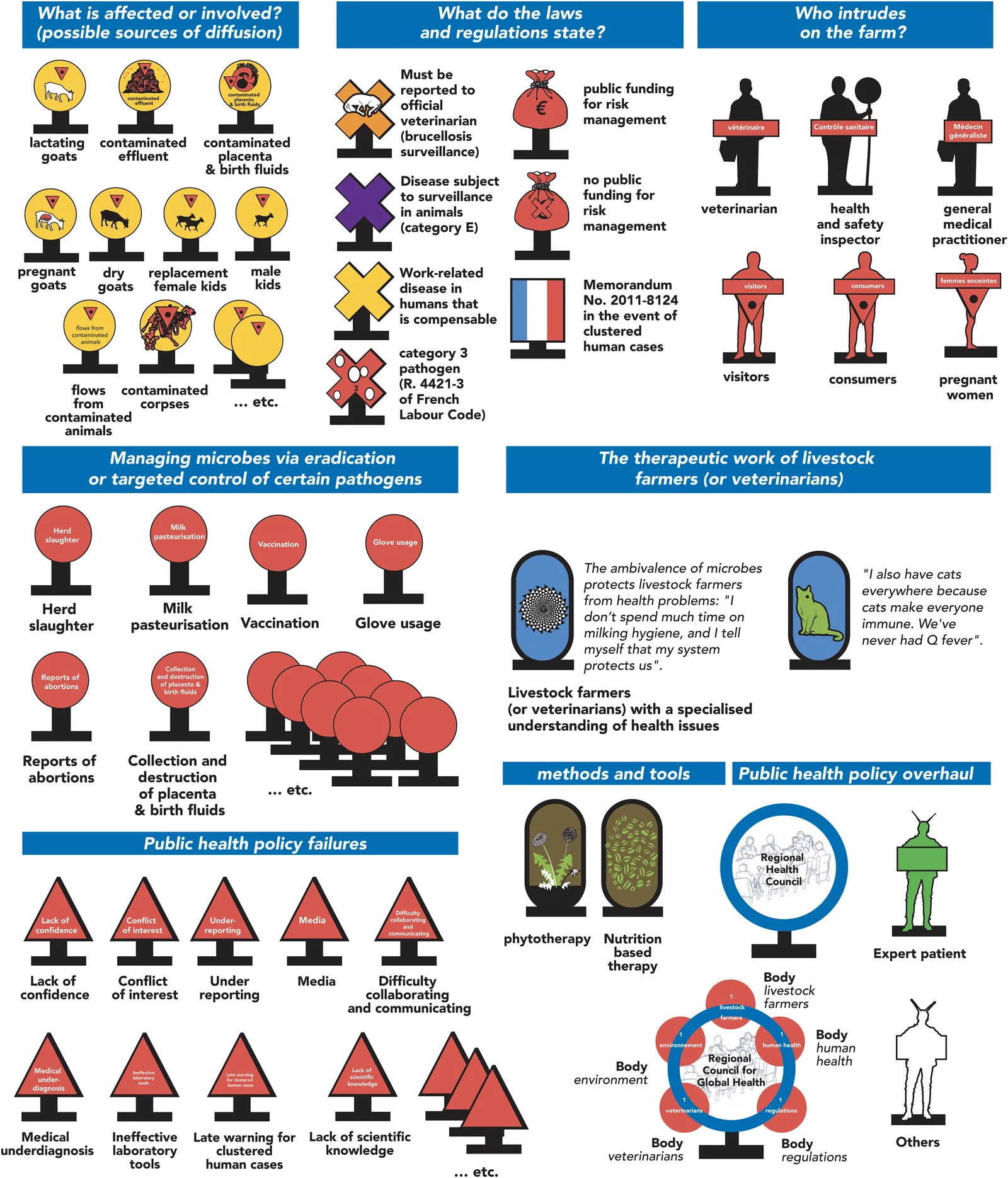
Non-exhaustive list of the cardboard figures made available to the players. The figures are selected and placed individually by the players on boards B, C, and D to encourage mutual acquaintance and discussion. Figures are grouped by categories: (5a) potential sources of disease diffusion, regulatory aspects, and stakeholders; (5b) microbes management approaches based on biosecurity or alternative therapeutic or healthcare practices; (5c) public health policy failures and potential overhaul.

### 3.2 Multistakeholder workshop

The ZOOJEU serious game was played by stakeholders during an event (i.e., experiment) held on May 4, 2023, in Prahecq, France. Details regarding the recruitment of the participants and the workshop practical organisation are provided in S3 File. The S4 File describes the self-evaluation process, providing details on implementation, output, and impacts. Overall, the project was seen as relevant, successful, useful, and effective. Its objectives were aligned with concerns on the ground; it produced results that responded to its objectives; it had impacts in the field; and it achieved effects at a reasonable cost. Briefly, there were 3 facilitators, 2 observers, and 23 participants. The participants had diverse professional and personal backgrounds. The group was composed of 4 goat farmers, 12 animal health professionals, 6 human health professionals, and one ‘expert patient’; one of the farmers had also been sick from Q fever and could therefore be considered as a second ‘expert patient.’ At the start of the event, the participants filled out GDPR forms and received a nametag with their first name. They also received a feedback form with open-ended questions to be completed before the end of the day and a 20-page brochure (see S5 File). After the introduction of the event, the participants were divided into three sets of players, each with a facilitator and seven to nine participants with expertise in human health, animal health, or goat farming. First, each set of players was asked to analyse the context of a particular goat farm; in practice, the farmer(s) playing the game were invited to choose their farm as the case study (see Fig 6a for details). Then, each set of players was given time to present their work, as shown in Fig 6b. The figure representing a hypothetical Regional Council for Global Health was put to work by all three sets of players. Overall, the players explained their perspectives and actions in an effort to establish a shared vision of what was happening on the farm. The conversations were both engaging and perceptive, culminating in moments of discovery (e.g., about the lack of scientific knowledge about the disease; or farmer workload during the goat kidding season; or the diagnostic odyssey of patients with chronic disorders). It became apparent that understanding alternative perspectives, such as those of farmers or patients, can lead other stakeholders to a rethink how disease management options should be designed. Tensions occasionally emerged regarding the handling of the abortions specifically and of diseases more generally (see verbatims in Fig 7). The main points that arose were that there is no connection at the local level between human and animal health, that farmers end up overwhelmed (e.g., too much info, too much responsibility, too much pressure), and that questions remain about how serious the risks actually are. An emphasis was placed on the importance of improving knowledge about risks and the utility of informing stakeholders about the lessons learned from past instances of Q fever. The event concluded with attendees agreeing to collectively pursue the work initiated during gameplay. In particular, they agreed on the need to further reflect on the potential role, nature, and governance of the hypothetical Regional Council for Global Health. The players made suggestions about the stakeholders who should contribute to this hypothetical Council. It was suggested that farmers should have a central position and that responsibility should be shared, so that the council could make decisions rather than simply offering advice. It was recommended that this council could especially deal with orphan zoonoses, depending on regional needs and gaps. A brochure summarising the day’s events was sent individually to participants by post three months after the event (see S6 File).

**Fig 6:**
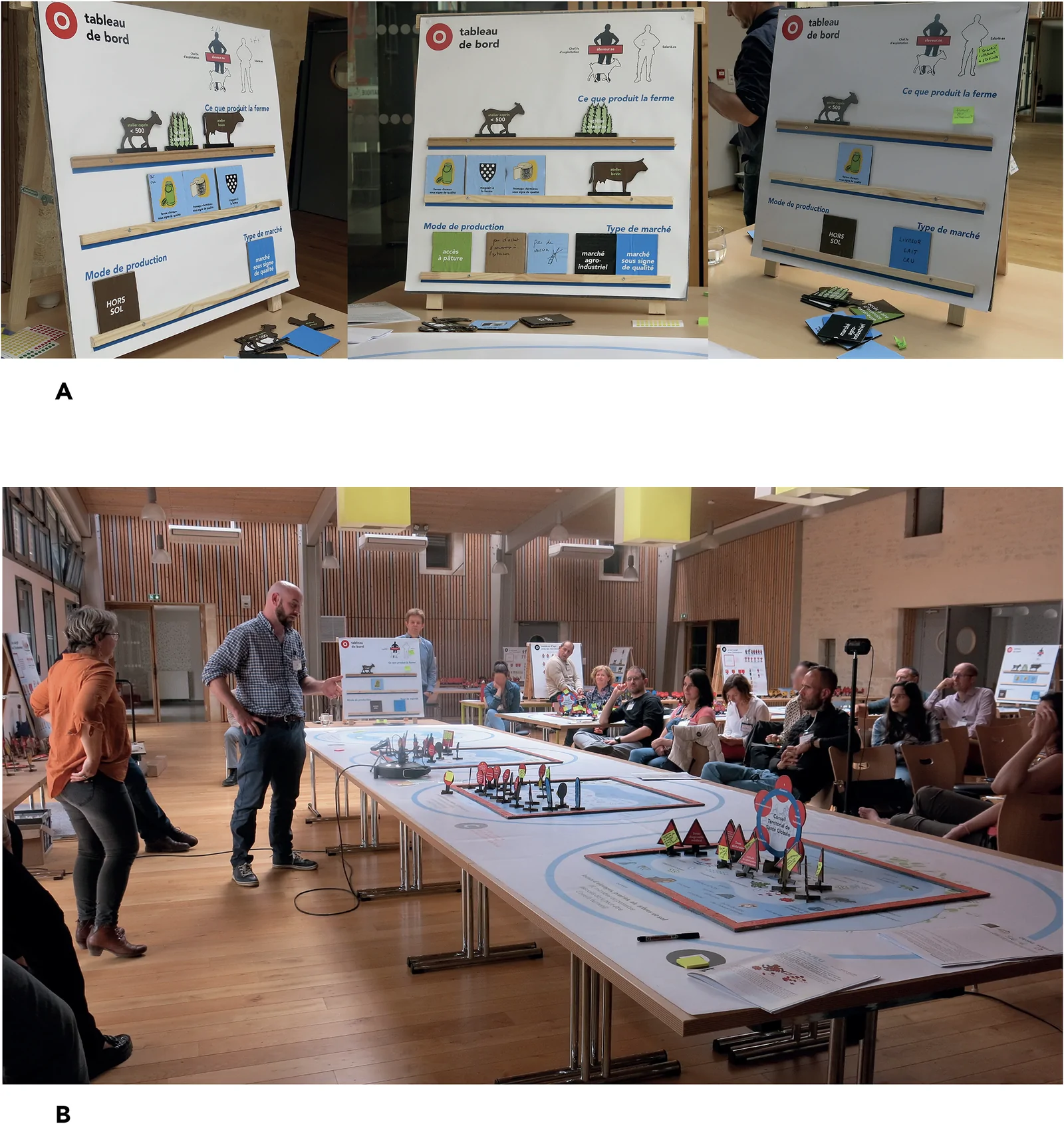
Photographs showing the ZOOJEU serious game when implemented with stakeholders. (6a) Boards illustrating the respective profile of the farms considered as a case study by the three sets of players and showing the diversity of the farm contexts examined: exclusively indoor farming *vs* with access to an outdoor exercise area; production of only milk *vs* production of milk and on-site sold raw milk cheese; organic farming *vs* conventional farming systems; other production facilities on the farm. (6b) Overall view of a game set during the session’s restitution by the players.

**Fig 7:**
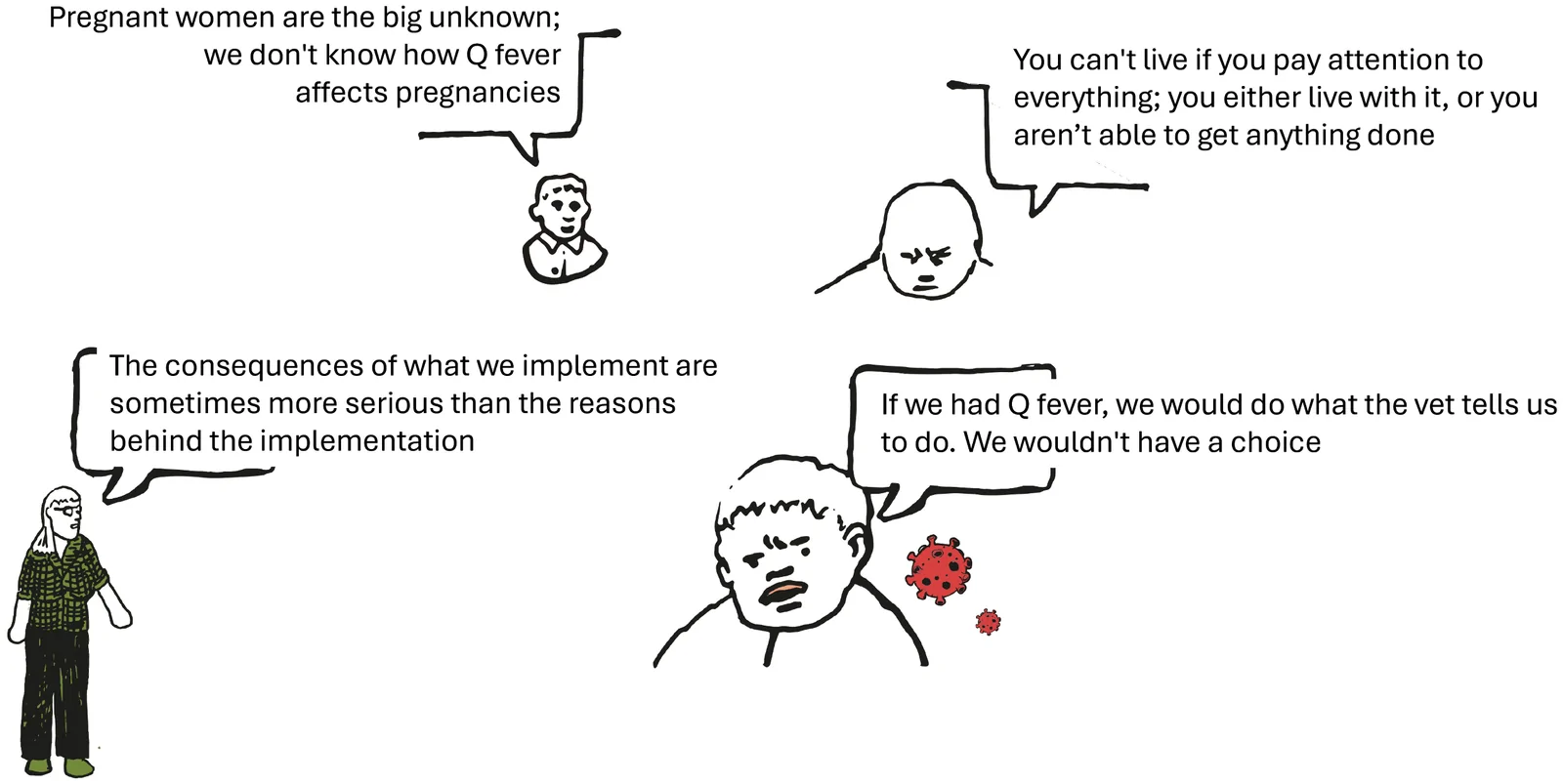
Illustration showing verbatim quotes collected during the workshop.

## 4. Discussion

Unlike highly regulated diseases, neglected and emerging diseases allow for greater collaboration among stakeholders when it comes to the possibility of developing comprehensive public health strategies. This study used Q fever in goats as a study system for designing an approach aimed at exploring alternative public health initiatives.

### 4.1 Research framework

Health policies related to livestock farming are often the work of professionals and government institutions or authorities, who, unlike farmers, have mandates to make decisions. One of ZOOJEU’s challenges was to address these asymmetrical power dynamics by exploring alternative approaches to developing public health strategies. We thus postulated that robustness (as per Olivier Hamant [57]) in the context of public health primarily stems from the ability of stakeholders to collaborate in defining courses of action based on their diverse knowledge and skills, and importantly, their shared responsibility. Thus, we view our approach as community based, as defined by the WHO [58]. Previous anthropological research has shown that there are diverse approaches to handling health issues related to livestock farming [25] but that this diversity has no outlet because public health policies are highly simplified and lack the technical, human and financial resources that would be required for a more complex approach; yet, the utility of management measures at local scales often depends on being able to handle greater complexity [59]. As research on zoonotic diseases occurs at an intersection of many scientific fields and areas of expertise, it is imperative to adopt a transdisciplinary approach, in which scientific, empirical and practical knowledge align. Next, research findings must be translated into actions to ensure that public health policies truly address the complex challenges related to animal, human, environmental, but also societal health [26,60]. An important aspect of this work is acknowledging that public health ‘experts’ are not the only ones with knowledge and skills: farmers are also highly knowledgeable and skilled even if they do not, at present, have any sort of official recognition.

ZOOJEU is an invitation to experiment with a new paradigm where farmers would have a mandate to address public health issues within a participatory research framework, meaning a collaborative effort by researchers and non-researchers to examine and find solutions for a problematic situation [48,61,62]. The key is to bring together diverse stakeholders, treat their different areas of knowledge and expertise with respect, and seek to build connections. To this end, we used public policy design, which fosters innovation by putting stakeholders at the heart of decision-making processes [46]. Our efforts were guided by the work of Horst Rittel, who criticised expert-driven planning design and advocated for a public policy design approach that could be applied to ‘wicked problems’ [43]. He proposed distinguishing between the two generations of public policy design [44]: the first being a linear, expert-driven, systemic approach suitable only for what he considers to be ‘docile’ problems and the second being a more mature version capable of handling more complex (’wicked’) non-linear situations, which require collective and democratic decision-making that considers ‘experts’ as occurring at the same level as other stakeholders. For Rittel, such is the ‘symmetry of ignorance,’ where experts in a specialised field of knowledge are united with non-experts who possess rich experience and in-depth local knowledge. Second-generation public policy design can be used to identify blind spots and decision-making biases. It also helps build trust and credibility among everyone involved in a system, whether they are experts or not.

First-generation public policy design, which draws exclusively on epidemiological or veterinary expertise, is not up to the task of handling zoonotic diseases: it is constrained by the limitations of the experts’ traditional avenues of action. For instance, economic questions are not addressed (who pays for any prophylaxis?), nor are political issues (conflicts of interest linked to promoting a vaccination-based public health strategy). Thus, Q fever is a ‘wicked problem’ that calls for a more democratic approach, but adopting a more democratic approach means evaluating the validity of various public health management strategies.

Overall, no single one of the four disease prevention models described by Dozon and Fassin [63] (profane constraint, magical religious, Pasteurian, and contractual) should serve as a universal standard. When we operate a democratic standpoint, precedence is not automatically given to recommendations based on biosecurity (profane constraint model) or vaccination (Pasteurian model). These two models have a place but only alongside the other two. The challenge is to hold space for community health, which requires the ability to reconcile contradictions.

### 4.2 ZOOJEU’s strengths and opportunities

A major strength of the ZOOJEU approach was that it created an environment for exploring stakeholders’ diverse realities, notably by making available representative objects that can be handled in the presence of others. It also encouraged all players to share their insights, unique perspectives, and areas of expertise in a climate of mutual respect—stakeholders with experience on the ground can exist on equal footing with government-appointed experts. The ZOOJEU approach fostered constructive dialogue in an unconventional setting, away from the formal framework of traditional meetings. It became apparent that discussing how risks are defined and perceived by each stakeholder can help clarify why different actions are taken by the different parties, thus prompting the group to reconsider the concept of shared risks. By fostering mutual understanding and acknowledging the specific needs and experiences of all stakeholders, disease management strategies could become inherently more effective and respectful of individual perspectives. Community health experiences in West Africa and South Asia are inspiring in this regard [48,64].

The ZOOJEU approach exposed players to multifaceted viewpoints toward regulation, health, and agriculture. It also shifted focus from risk and zoonosis to global health, including farmer and animal welfare. Stakeholders recognised the need to continue discussions on a shared vision of risk in the context of Q fever management and to address the imbalance in stakeholder power that arises because of legal and regulatory frameworks. Such was reflected in the desire to establish a suitable task force. The stakeholders proposed setting up a working group to further explore this idea, where collaboratively generated proposals could be tested in the relevant geographic area. Beyond the scope of Q fever, the workshop operated as an experiment for the general framework represented by ZOOJEU. It yielded highly promising results that validate its effectiveness and potential for fostering mediation and collaborative action among stakeholders in diverse contexts. ZOOJEU’s design appears to be particularly effective for addressing issues related to neglected or emerging infectious diseases, while simultaneously spurring the engagement of farmers. The latter is fostered by facilitators, who ensure that farmers’ input receives specific attention: their role is crucial in the success of the approach, particularly when there is a wide diversity of stakeholders, some of whom feel less legitimate than others to express themselves. As a result, the serious game specifically tackles the power imbalance traditionally experienced by farmers in disease management contexts.

We also explored using ZOOJEU as a teaching support tool, testing it out with different types of students such as future farmers, agricultural advisors, agricultural engineers, veterinarians, medical doctors, health officials (see S7 File for details). When ZOOJEU was employed in educational contexts, it was essential to emphasise that the serious game does not involve role-playing. Role-playing would negate the approach’s emphasis on respecting the expertise of stakeholders and particularly that of farmers.

### 4.3 Limitations, risks and future challenges

Many methodological challenges exist in transdisciplinary research, above all when the research involves participatory processes [27,62,65]. Such was also our experience during our project. In this section, we discuss some of the potential biases and weaknesses of our approach as well as the methodological challenges that we encountered. Further information can be found in the Supplementary Materials (S4 File).

First, we have no evidence that our participatory process succeeded in capturing the stakeholders’ different perspectives. We created space for a magical religious model of disease prevention [63] to be expressed alongside other models of disease prevention, in parallel with or as a substitute for the profane constraint or Pasteurian models that would probably have been promoted by the veterinarians with health mandates. Although we clearly stated at the workshop, prior to gameplay, that everyone was free to voice their opinions about what they consider appropriate in terms of health measures, the presence of health authorities may have been enough to suppress the expression of non-conformist views. As facilitators, we presented the biosecurity narrative in a way that minimised any sense of coercion. This effort was essential since the occurrence of a large-scale zoonotic event could effectively result in coercive health policies, destroying attempts to democratise public health strategies. Yet, at the same time, we considered that it was important to include numerous figures related to biosecurity approaches (see **S5 File**) because we felt it is crucial to allow for critical collective discussions, given the predominance of such approaches in public health management guidelines. Before sharing the serious game more broadly, we need to consider how multiple users, including in educational contexts, can play the game without biosecurity approaches immediately pushing out all other approaches. To deal with this issue, we have started collaborating with student groups with the aim to produce a version of ZOOJEU that can be used without the research team needing to be present to provide expertise and facilitation. This version of ZOOJEU should give equal importance to the various public health stakeholders (e.g., doctors, veterinarians, regulatory authorities, farmers, researchers) while also holding space for the friction and tensions that usually arise from differences in stakeholder approaches. Second, we did not assume that the players were acting as representatives for others of the same profession or social category. Rather, our approach takes into account the specific characteristics of the study context, which affects not only the stakeholders but also the ways in which regulations are applied, or livestock farming is conducted. Furthermore, we have no evidence that the stakeholders who played the game were all similarly interested in a comprehensive approach to health. The results may notably have been impacted by the fact that the study area had been hit by an outbreak of human Q fever, and that some participants had previously been infected with Q fever. During gameplay, we witnessed multiple and not necessarily compatible perspectives on health and public health strategies, which fits with what was found by the anthropological research highlighted in the comic strip. However, the fact that all three sets of players independently selected the figure representing the hypothetical Regional Council for Global Health reflects that stakeholders see a need for a regional body that considers the viewpoints of all stakeholders, not just those who are usually called upon by health authorities. We are currently pursuing the participatory process to further investigate this issue with a panel of stakeholders, including farmers, health and farming professionals, everyday citizens, and elected officials [66]. A major challenge that may arise in the long term is keeping the stakeholders engaged, given the different time scales of research and ‘real life.’

Third, it may seem frustrating that the workshop did not yield a suite of management recommendations agreed upon by all the stakeholders. The fact is that there existed an inherent diversity of opinions, which also means conflicts, disagreements, and differences when it comes to establishing a common framework for coordinating the actions and contributions of all parties. The main recommendation that arose from the game was the following: Q fever is associated with uncertainty and complexity, but employing a democratic approach makes it possible to respond to the situation in a way that accounts for all stakeholders. Here, a democratic approach is defined as a participatory research process based on the sharing and joint exploration of experiences and knowledge. It is for this reason that, although it is included in the Gamae serious game library (https://ludotheque.gamae.fr/game/117), ZOOJEU is not playful; it also does not produce immediately applicable results [67]. It is primarily a design-based mediation approach. A significant outcome of the workshop in this regard was the strengthening of relationships between the Mutuelle Santé Agricole (Health Insurance Company for Agricultural Professionals) and stakeholders involved in goat farming or animal health: these stakeholders are now organising regular meetings and have launched together an awareness campaign focused on zoonotic diseases (including Q fever). Overall, the project’s main outcome is its continuation via *Transition ZOOJEU*, during which we have organised other participatory science events. The latter have allowed stakeholders involved in goat farming or animal health to discover regional health organisations with democratic structures, such as the Regional Congress for Health and Autonomy (*Conférence Régionale de la Santé et de l’Autonomie* [CRSA]) and the Regional Health Councils (*Conseils Territoriaux de Santé* [CTS 79]) of Nouvelle-Aquitaine); stakeholders have also been able to engage in exchanges with some of their members.

Finally, a major weakness during the ZOOJEU workshop was that we lacked the means to properly assess and capture the perspectives of the various players, despite the audio recordings, photographs, and films. Also, despite our best efforts, our approach may have created an implicit power imbalance between experts and non-experts as well as between researchers and non-researchers: the ZOOJEU approach was created by researchers, who also presented it and acted as facilitators. By asking the farmers to speak first and by emphasising the perspectives of farmers via the comic strip, we have however highlighted that researchers can use their privilege to underscore how knowledge from different sources (e.g., stakeholders with or without government mandates) should hold equal weight in public health discussions. Nevertheless, we consider that it is essential to name this concern. This is why, for current events, facilitators are now not only researchers but also people that are familiar with how the approach works, to whom we provide instructions.

## 5. Conclusions

The ZOOJEU approach has the potential to serve as a unifying framework, facilitating the establishment of a ‘common narrative’ that could inform a more democratic form of public health when there are of conflicts, crises, or misunderstanding among different parties. The ZOOJEU approach is particularly well-suited to addressing issues surrounding the management of zoonoses, especially neglected and emerging diseases in the context of current global changes. Its approach may also be useful for tackling health issues associated with non-communicable diseases. Finally, its utility as an educational tool should be explored further.

## Supporting information

S1 File Comic strip summarizing the main results of the anthropological studies published by Ramillien et al. [25] in an illustrated and narrative form. A former version of this document (in French) was mailed to stakeholders who signed up to participate in the serious game workshop. This second version was produced to better suit educational contexts: it begins with the question of how to deal with a zoonosis when regional stakeholders do not take action and are operating in different realities. The document is divided into four parts: (i) the first page introduces goat farming as a major agricultural industry in the Niort urban area, citing agricultural statistics from the Deux-Sèvres Chamber of Agriculture; (ii) next, the comic strip delves into the reality experienced by public health stakeholders, presenting a dramatisation of how health authorities handled the 2017 cluster of human Q fever cases in the Niort urban area; (iii) the subsequent section shows how stakeholders may tackle issues related to zoonotic diseases by contrasting regulatory and public health strategies with the experiences of farmers; the content underscores that a farmer’s experience with Q fever depends on how much they delegate health risk management; (iv) the document concludes by acknowledging the diversity of realities and positions, and it invites stakeholders to participate in the serious game workshop to get to know each other better and mutually discuss their experiences.
(PDF)

S1 Video Animated video presenting the storyline of the comic strip. English-language version of the video. The original French narration was translated and re-voiced using Wondershare Filmora (v15.2) AI translation/text-to-speech tools; English subtitles were generated automatically and manually corrected by the authors. All visual and audio materials in the video are original and are provided under the article’s CC BY 4.0 license.
(MP4)

S2 File Facilitation directives and step-by-step description of the ZOOJEU game stages. (DOCX)

S3 File Description of the methodology followed for the recruitment of the participants and the multistakeholder workshop practical preparation and implementation. (DOCX)

S4 File ZOOJEU self-evaluation document following the self-evaluation guide for participatory science programs *Collectif Vigie Mer.* (DOCX)

S5 File 20-page brochure (in French) delivered to the workshop participants the day of the event. The brochure is divided into four sections: the schedule for the day, graphical feedback on the anthropological study performed by Ramillien *et al.* [25], a description of the stages and elements of the serious game, and the acknowledgements.
(PDF)

S6 File 12-page brochure (in French) sent by mail to the workshop participants three months after day of the event. (PDF)

S7 File Brief description of the use of ZOOJEU in educational contexts. (DOCX)
