# Supplementary material for: ZOOJEU: Using Q fever to highlight a participatory science approach for fostering the co-design of management strategies for neglected zoonotic diseases
Source: PLoS Negl Trop Dis. 2026 Oct 15;20(10):e0014618. doi: 10.1371/journal.pntd.0014618 (PMC13671574; doi:10.1371/journal.pntd.0014618)
Supplement: Supplementary file 1 [file pntd.0014618.s001.pdf]

Q Fever

Building a shared narrative

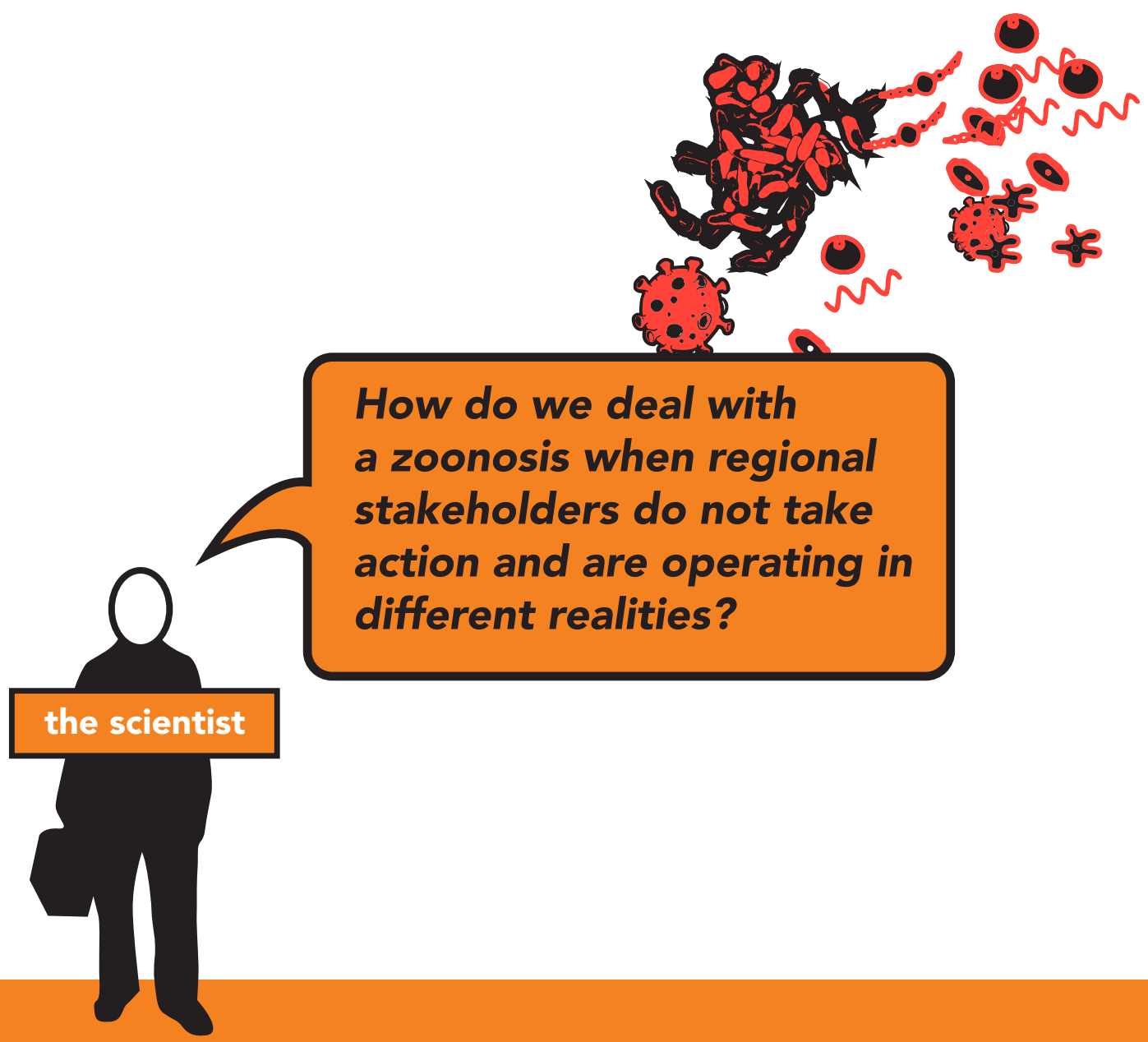

The goat industry of Deux-Sèvres

The French administrative department of Deux-Sèvres produces the largest number of goats and greatest volume of goat milk in the country. Goat farmers in Deux-Sèvres rear 37% of the goats found in France and more than 50% of the goats found in the region of Nouvelle-Aquitaine. These 150,440 goats are found across 523 farms with herds of more than 20 goats each; they produce 1,252,302 hl of milk, corresponding to 20% of national production levels. Consequently, the department of Deux-Sèvres leads in goat milk production in France.
Organic goat milk production remains limited in Deux-Sèvres. In 2018, 3,407 goats were reared on organic farms in the department, which represents 2.2% of the goats being reared in Nouvelle-Aquitaine. Twenty-three farms have been certified organic, and eight are in the process of becoming certified. There is only one PDO goat cheese: Chabi-chou du Poitou, which accounts for around 1% of production in Deux-Sèvres. Over the last 30 years, the number of goats per farm has steadily increased, but the number of farmers has decreased. On average, there are 289 goats per farm in the department.

Distribution of goat herd sizes in Nouvelle-Aquitaine (number of goats per km2) in late 2010

number of goats per km2 (Deux-Sèvres department)

- less than 10
- from 10 to 30
- over 30

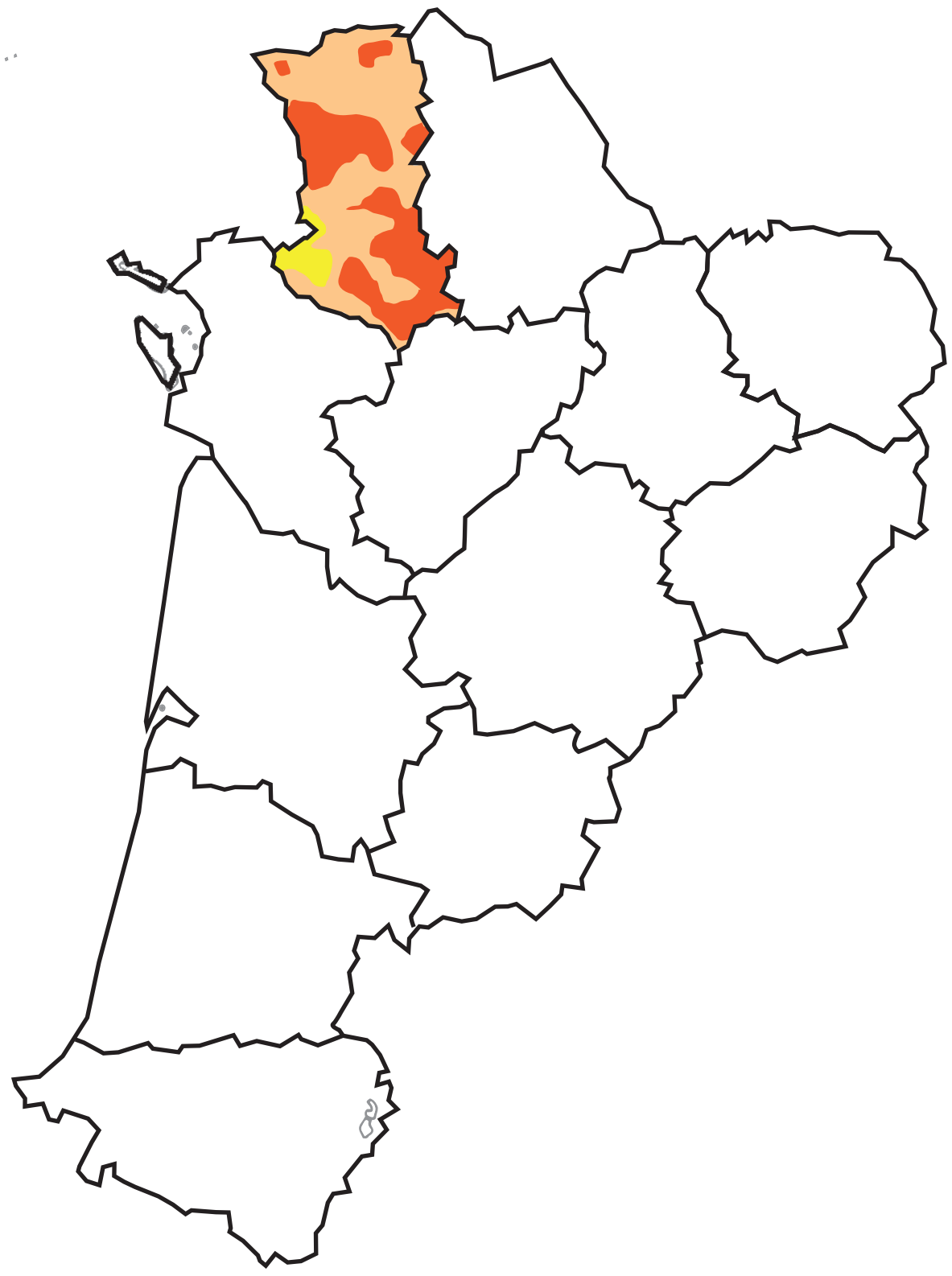

Distribution of herd sizes across farms

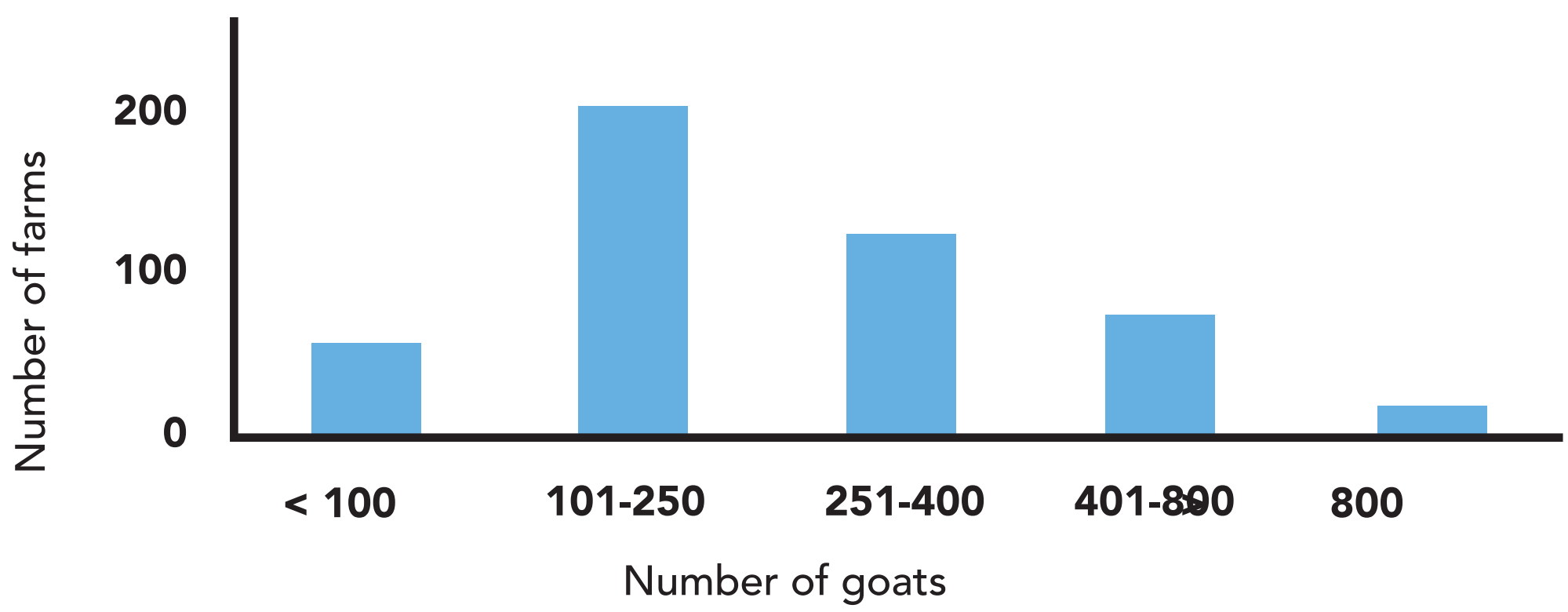

Source: Deux-Sèvres Chamber of Agriculture

Clarifying the different public health responses to a zoonosis

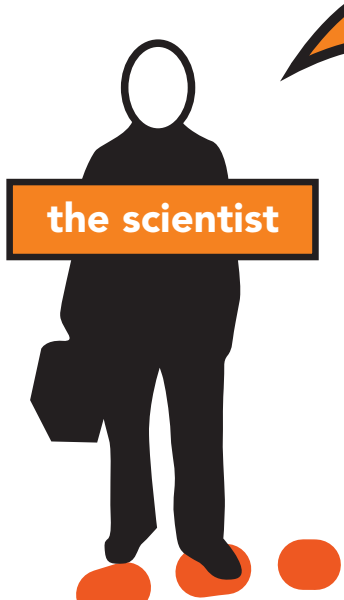

There are several possible responses to a zoonosis. However, given the absence of an objective cross-disciplinary evaluation approach, it is challenging to identify the best course of action for all stakeholders, whether they are involved in regulation, public policy, research, or livestock farming. *Which criteria should be used? Which factors should be taken into account?*

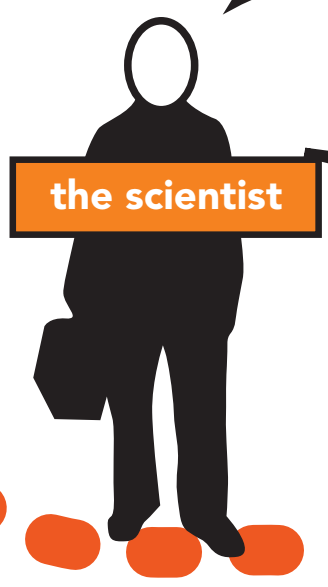

To better understand the range of responses, we first describe how the appearance of a zoonosis in a region of France resulted in regulatory action by the national government...

...then we analyse the regulatory action's scope, including
- *how it affected government agencies*
- *how it affected farmers, depending on whether or not they delegate health risk management to others...*

It is difficult to take all stakeholders into account. Zoonoses also elicit the involvement of other stakeholders, such as local residents, hospitals, and general medical practitioners. We do not discuss them here, but they will come up later...

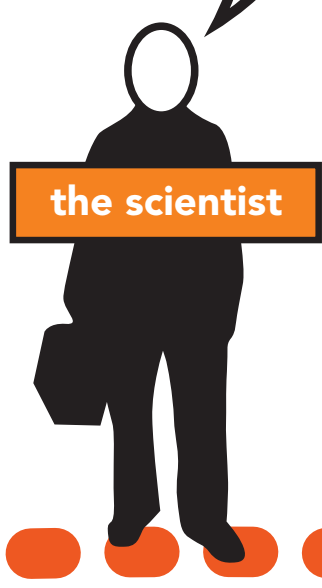

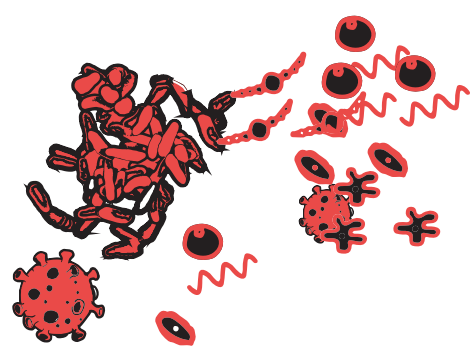

What are the regulatory responses to a zoonosis like Q fever?
When do these responses come into play?

regulations provide a biosafety framework to protect public health

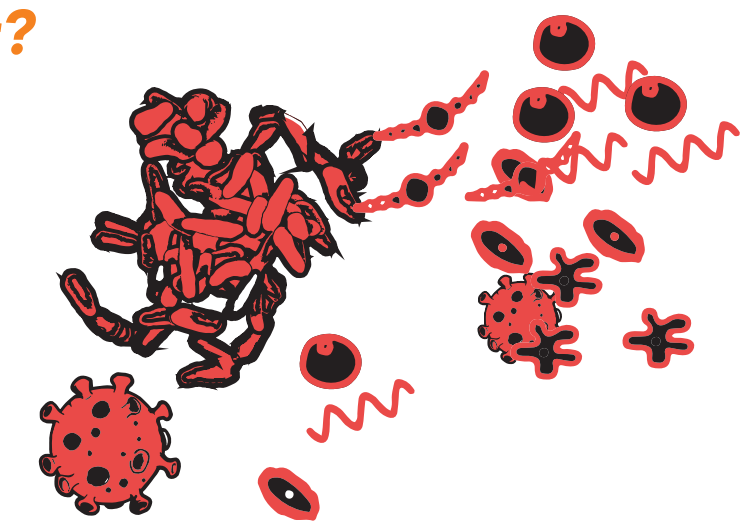

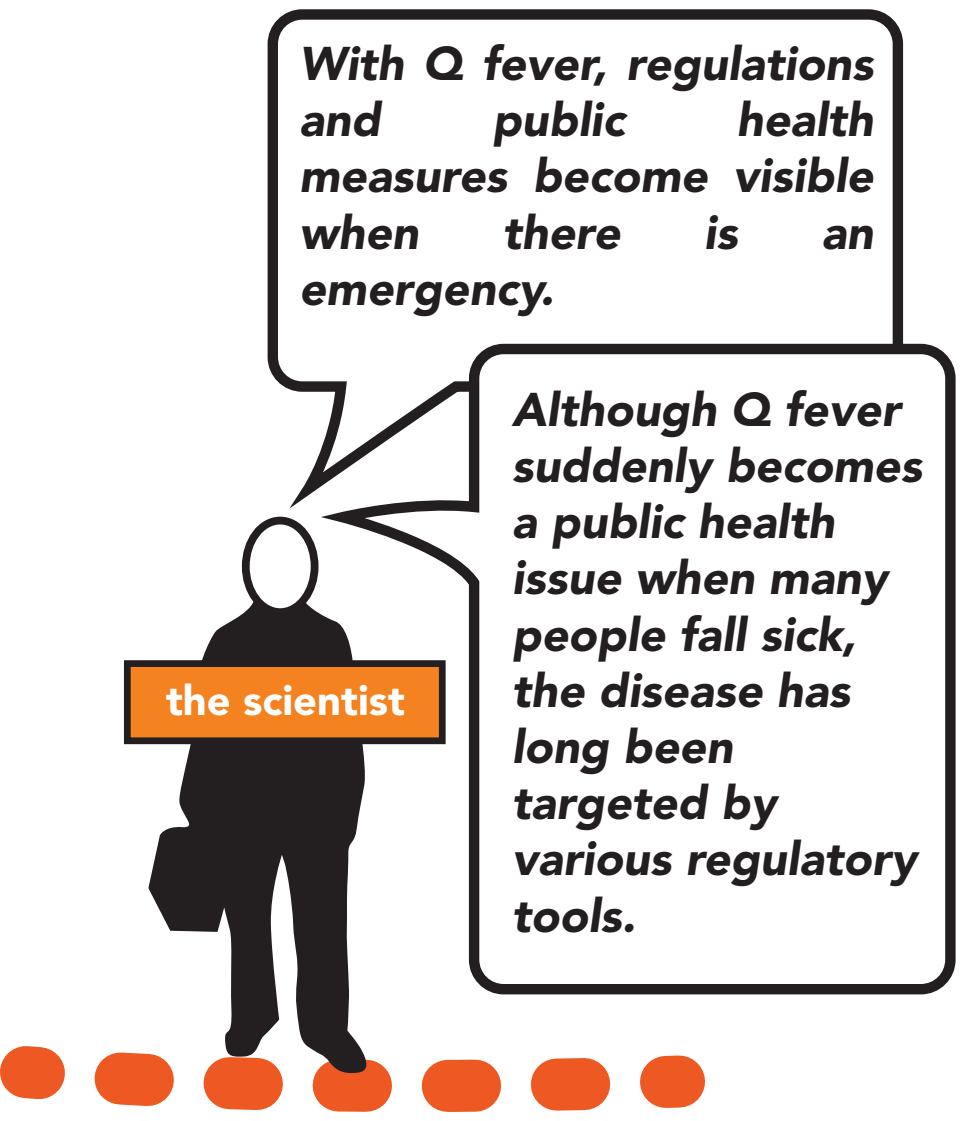

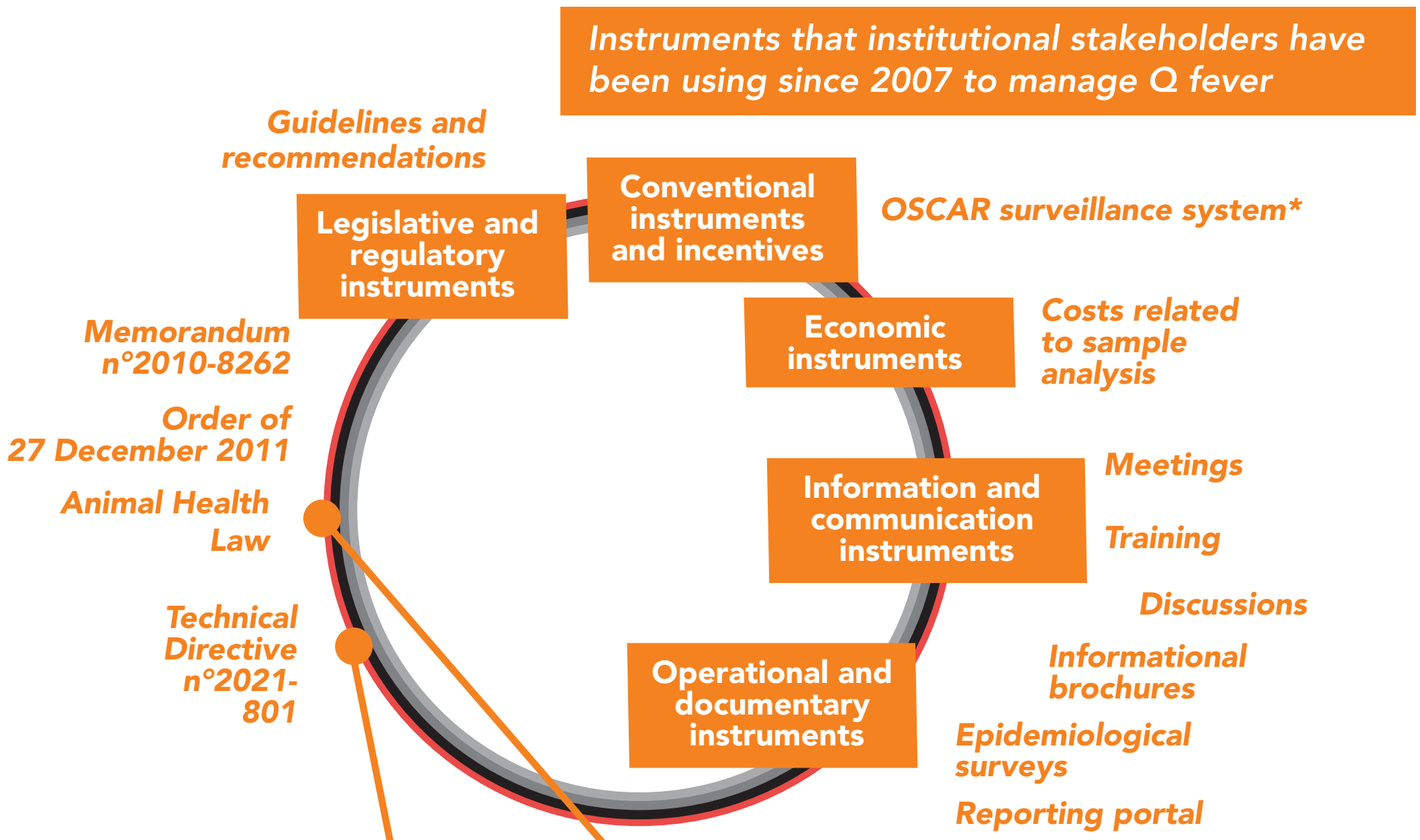

Before the adoption of Regulation (EU) No. 2016/429 of the European Parliament and of the Council of 9 March 2016, known as the Animal Health Law, along with the Implementing Regulation (EU) No. 2018/1882 of the Commission, Q fever was a category 3 health hazard in France, meaning disease prevention, surveillance, and control measures were not the government's responsibility. In the context of livestock farming, no regulations were applicable except when clusters of human cases occurred. In such situations, it was theoretically the responsibility of the prefect to implement public health measures (Articles L. 2212-2 and L. 2215-1 of the French General Code for Local Authorities). Control strategies were primarily based on the measures described in the control plan for clinically affected livestock farms, drawn up by the French Association for Animal Health Certification (ACERSA) in 2007. After the regulation's adoption, Q fever fell into category E and became subject to surveillance within the EU, requiring all Member States, including France, to report any cases of Q fever identified within the country. To this end, Technical Directive No. 2021-801 established that a portal must be used for reporting suspected or confirmed cases of animal diseases listed in the Animal Health Law. Furthermore, France's Departmental Directorates for Population Protection (DDecPPs) can use Memorandum No. 2010-8262 as a reference: it describes the diagnostic tools available for Q fever screening and the procedures for interpreting positive results.
Finally, when Q fever jumps from livestock to humans, the disease becomes a public health issue and must be managed by the national government, even if no such requirements existed prior to the adoption of the Animal Health Law. To meet this objective, France's Directorate-General for Food (DGAL) issued Memorandum No. 2011-8124, which provides the DDecPPs with an investigation protocol and management measures that can be used on small ruminant farms if clusters of human cases occur.

A cluster of human Q fever cases in the Niort urban area in 2017*

Starting in early May, several people were admitted to the local hospital with hepatitis...

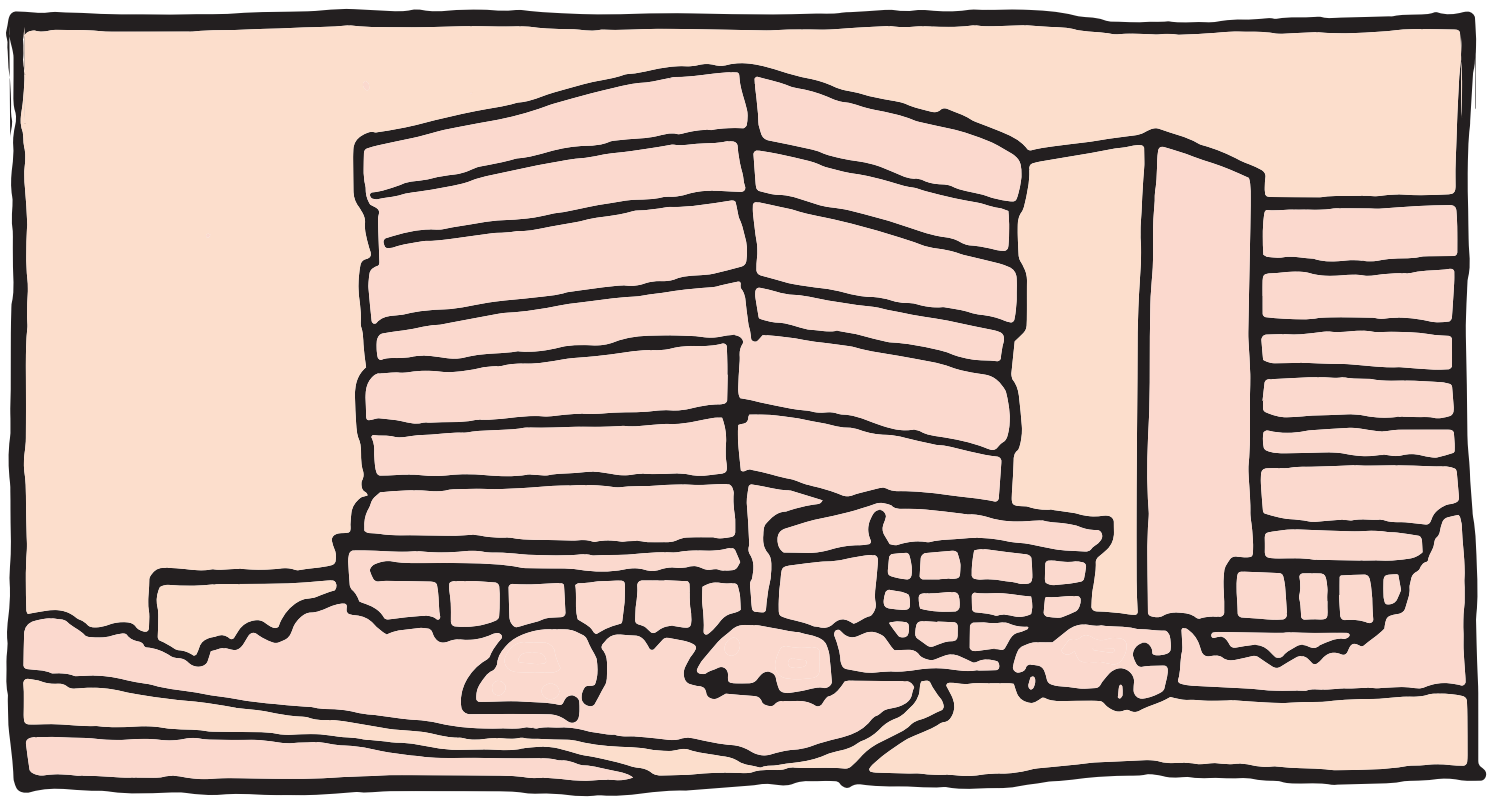

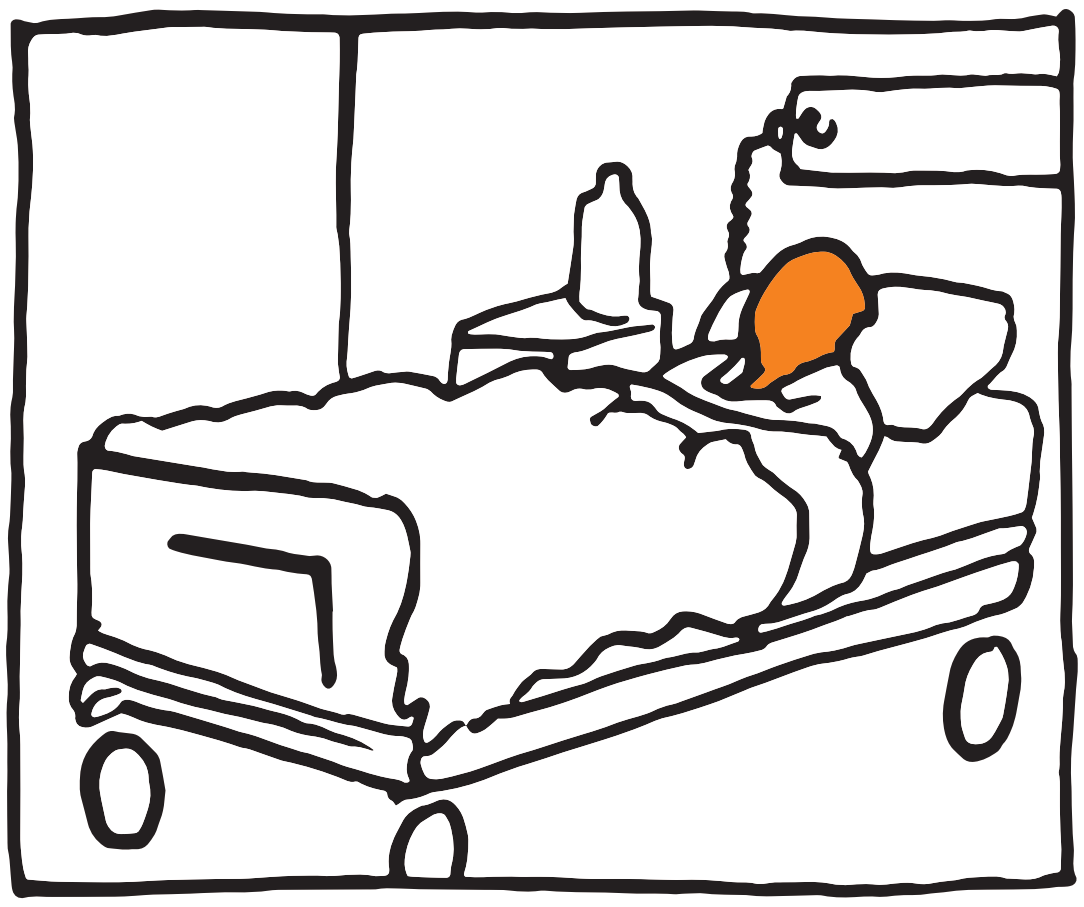

Blood samples were taken from these patients, and the serological tests showed...

...the presence of antibodies against a small bacterial species...

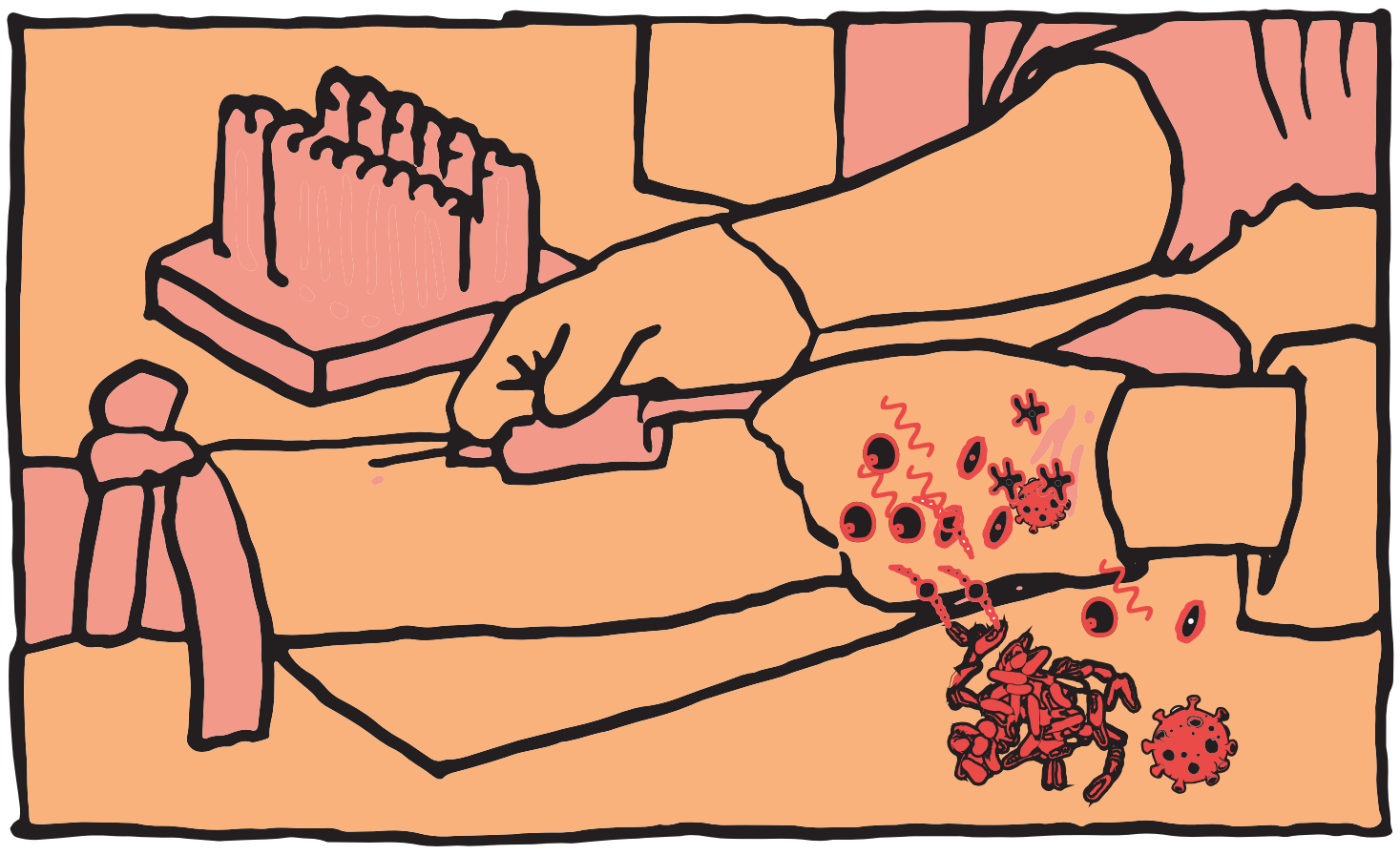

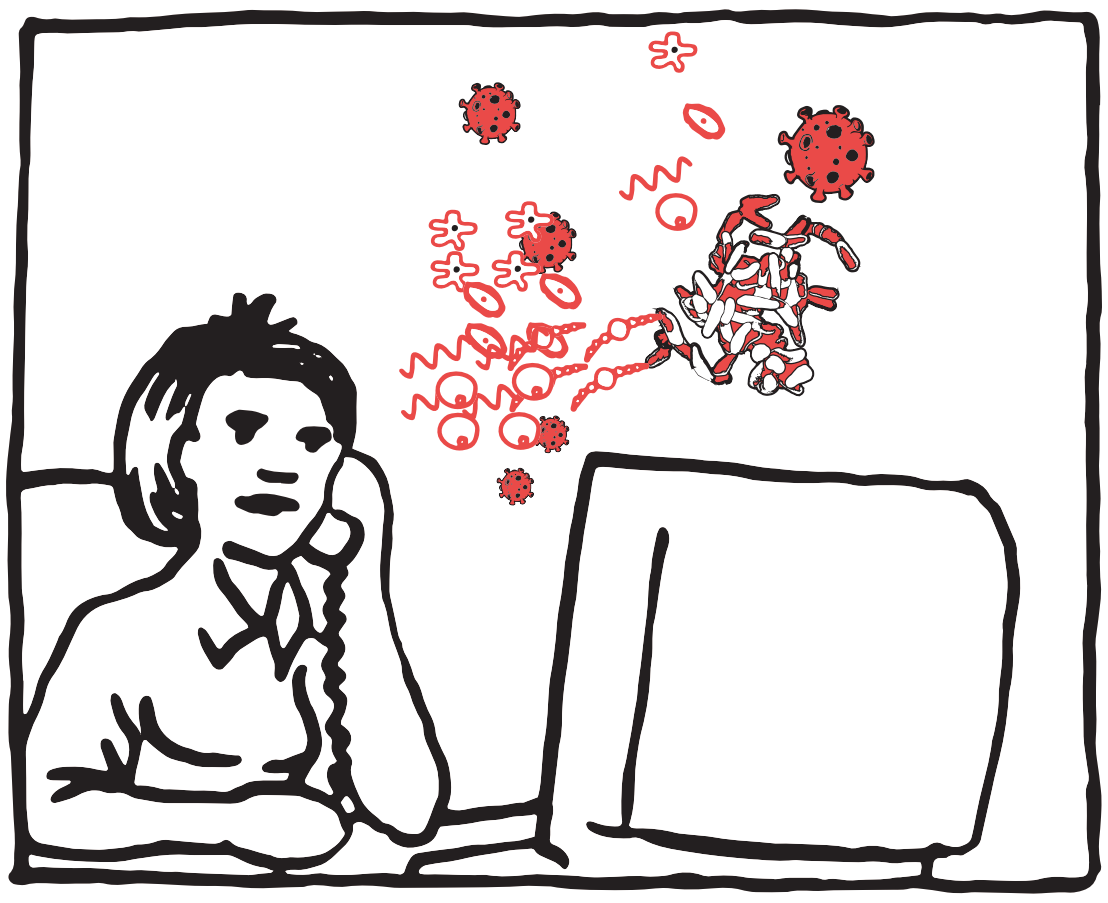

The bacterium was *Coxiella burnetii,* the pathogen that causes Q fever

The blood samples were sent to a national reference centre (CNR) to confirm the results

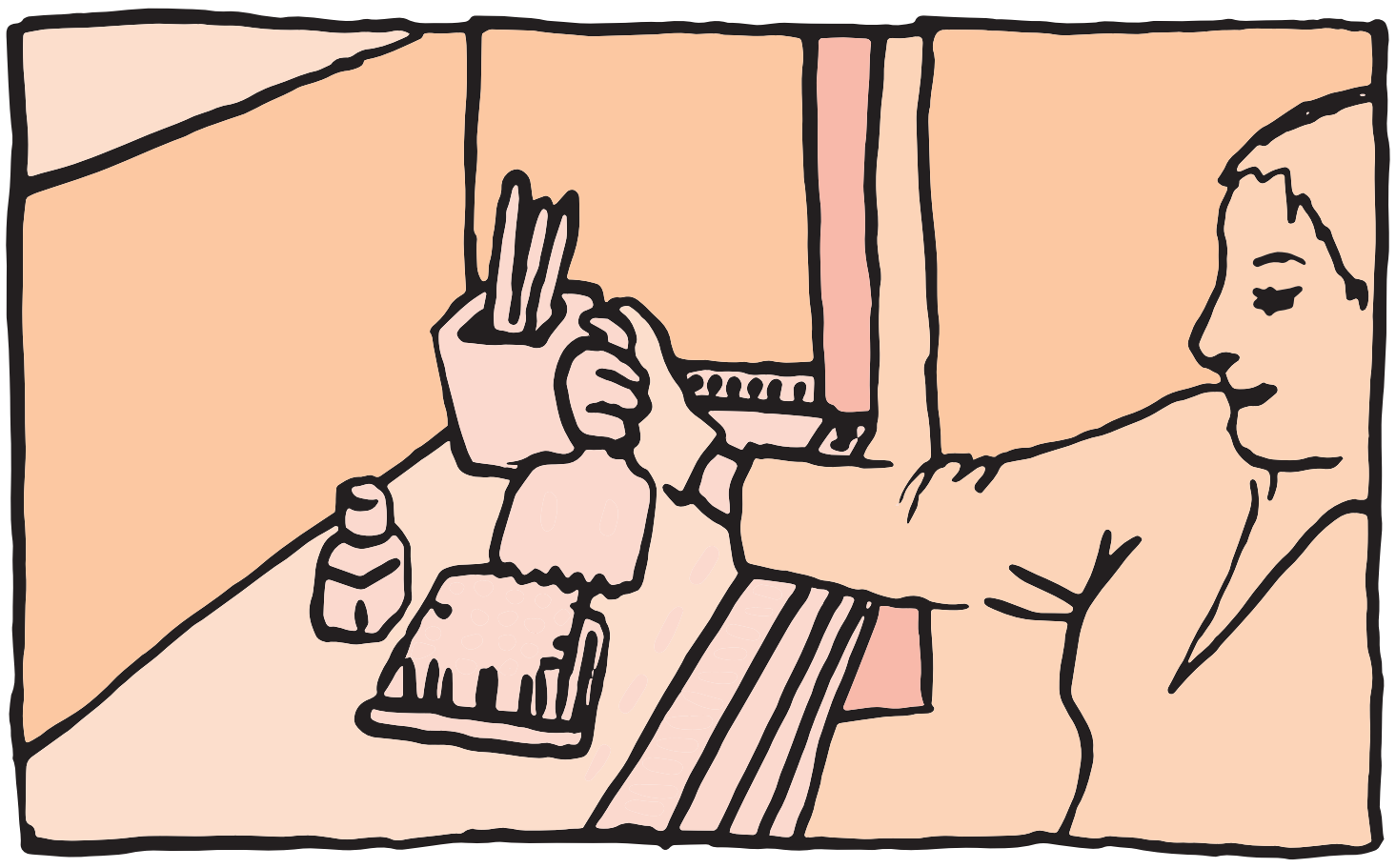

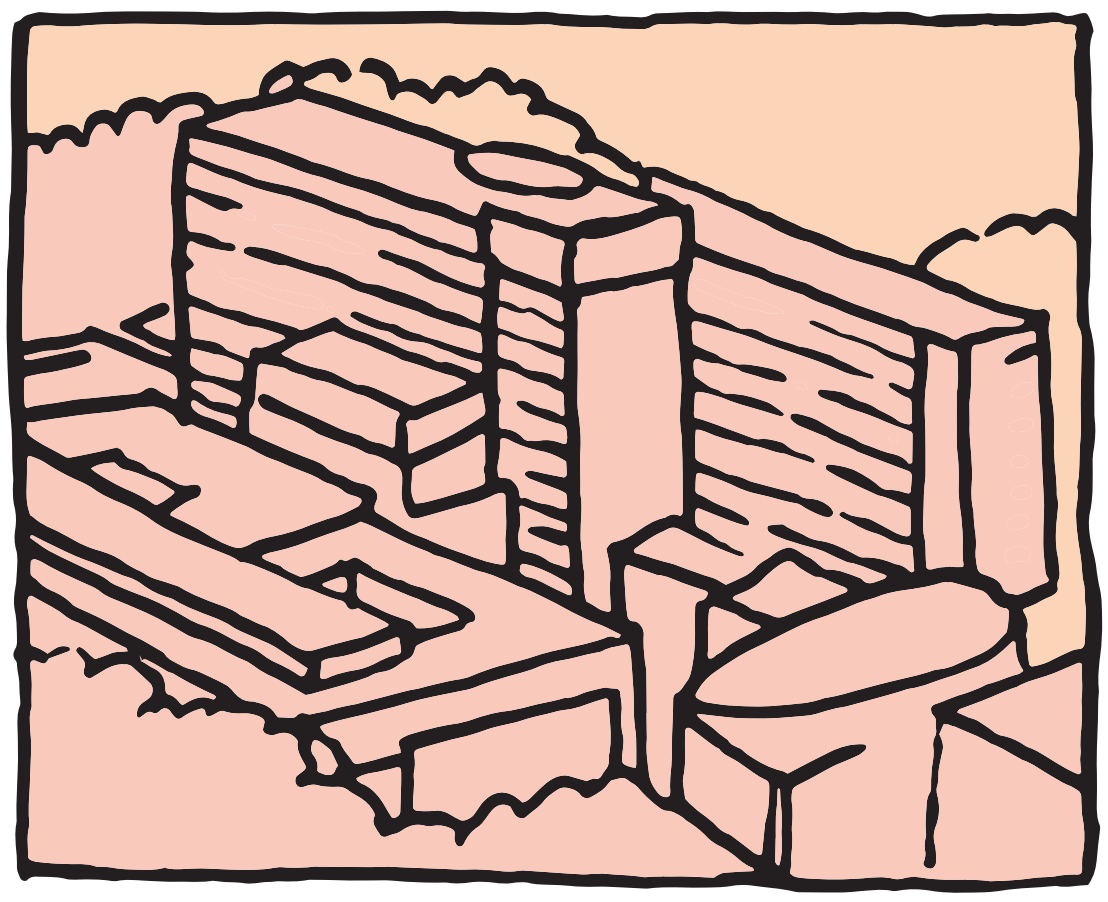

On Tuesday, June 6, the hospital's infectious disease specialist reported the situation

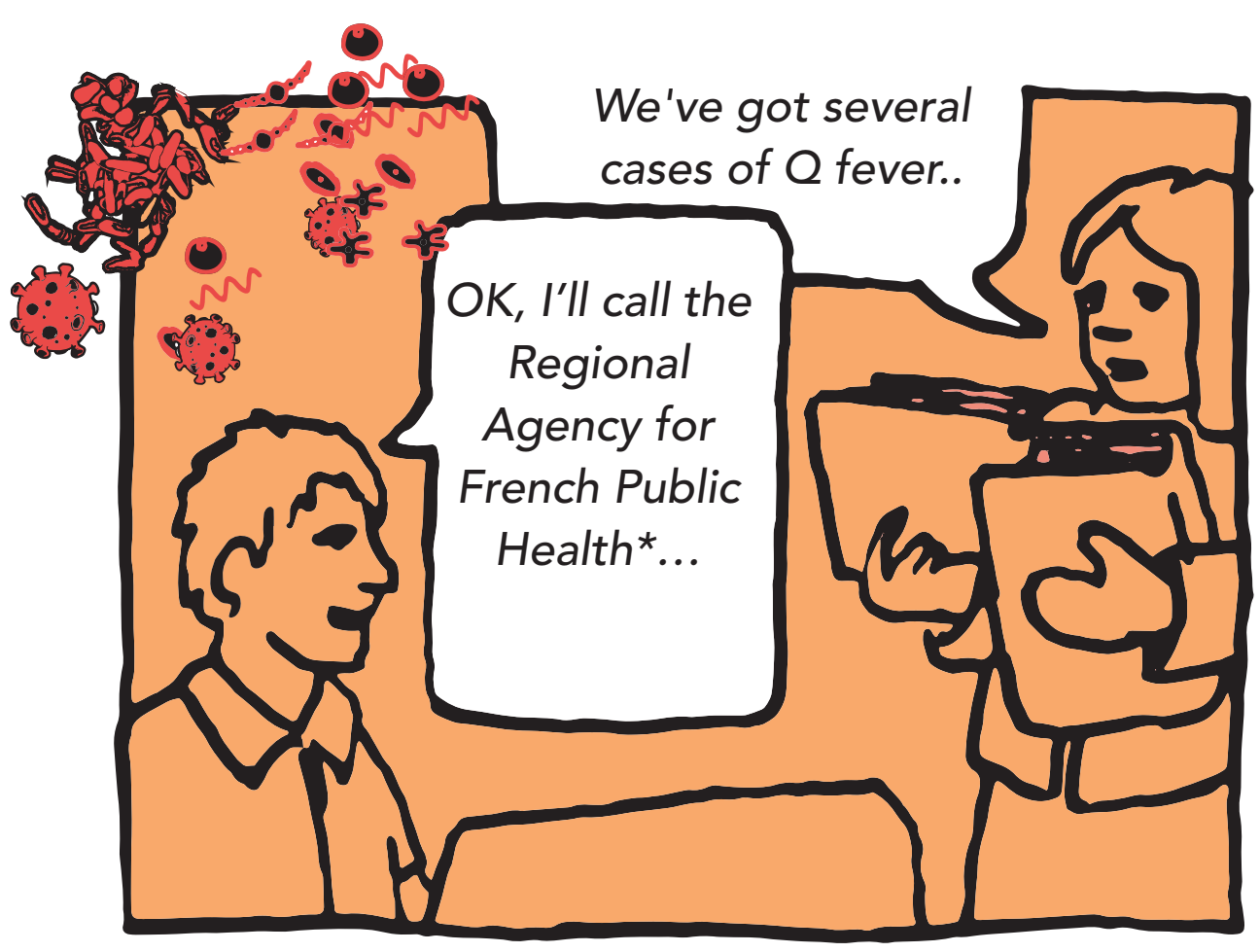

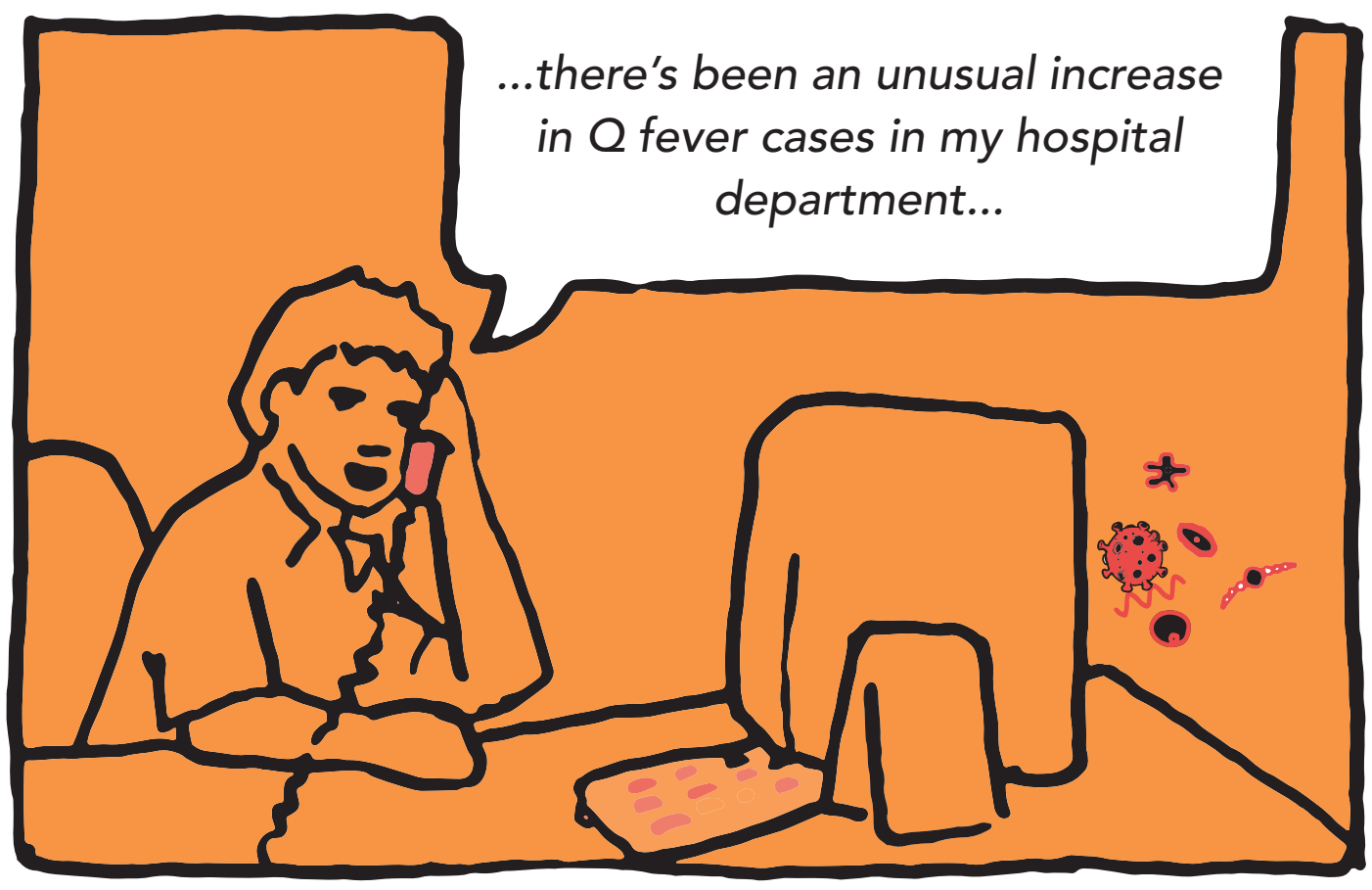

* Based on the following report: Bernadou A, Lambert Y, Gache K. Cas groupés de fièvre Q, CH Niort, avril-mai 2017. Saint-Maurice: Santé publique France, 2019. 30 pages. Available at https://www.santepubliquefrance.fr/)

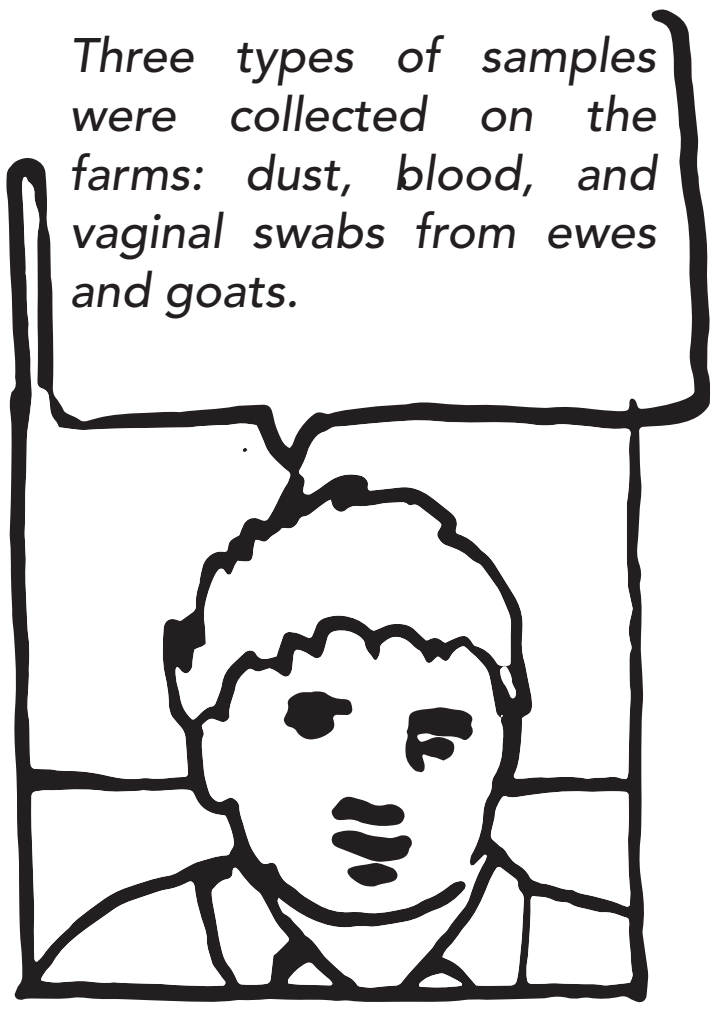

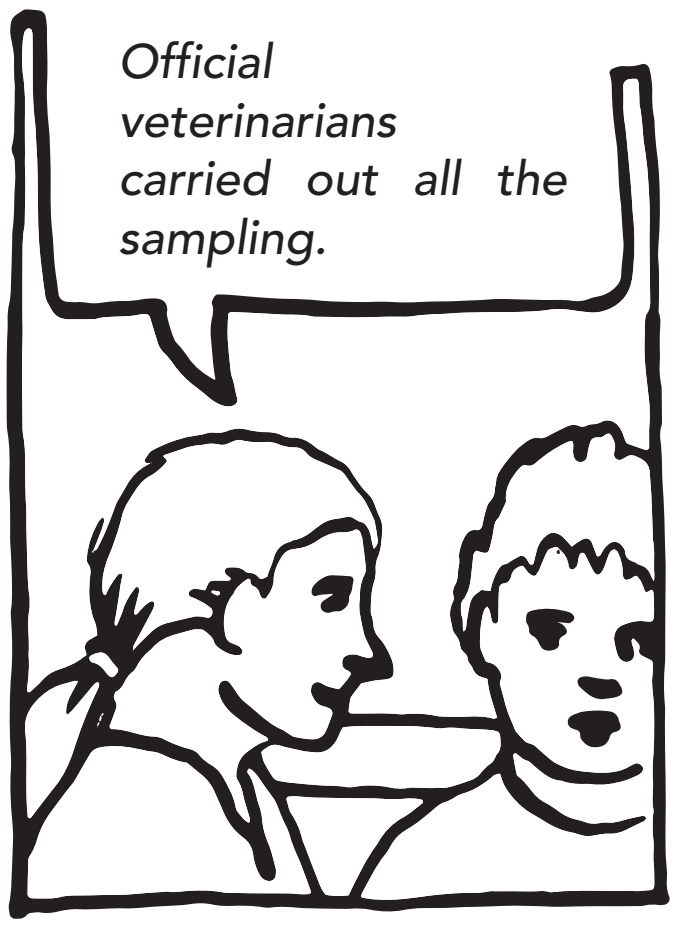

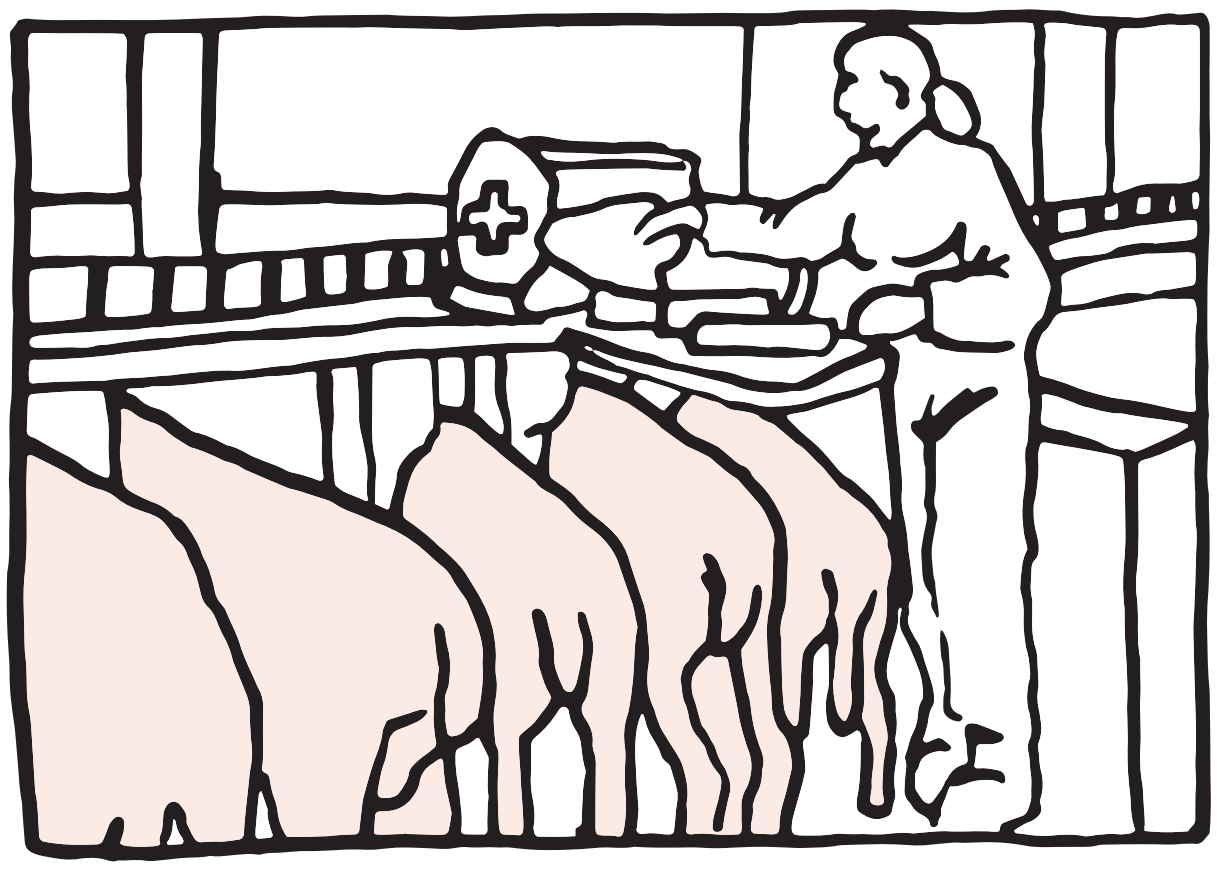

Dust samples complex to analyse, and research is still being conducted on their use as indicators. They were analysed at the national reference laboratory.

The blood samples and vaginal swabs were analysed by a local veterinary laboratory.

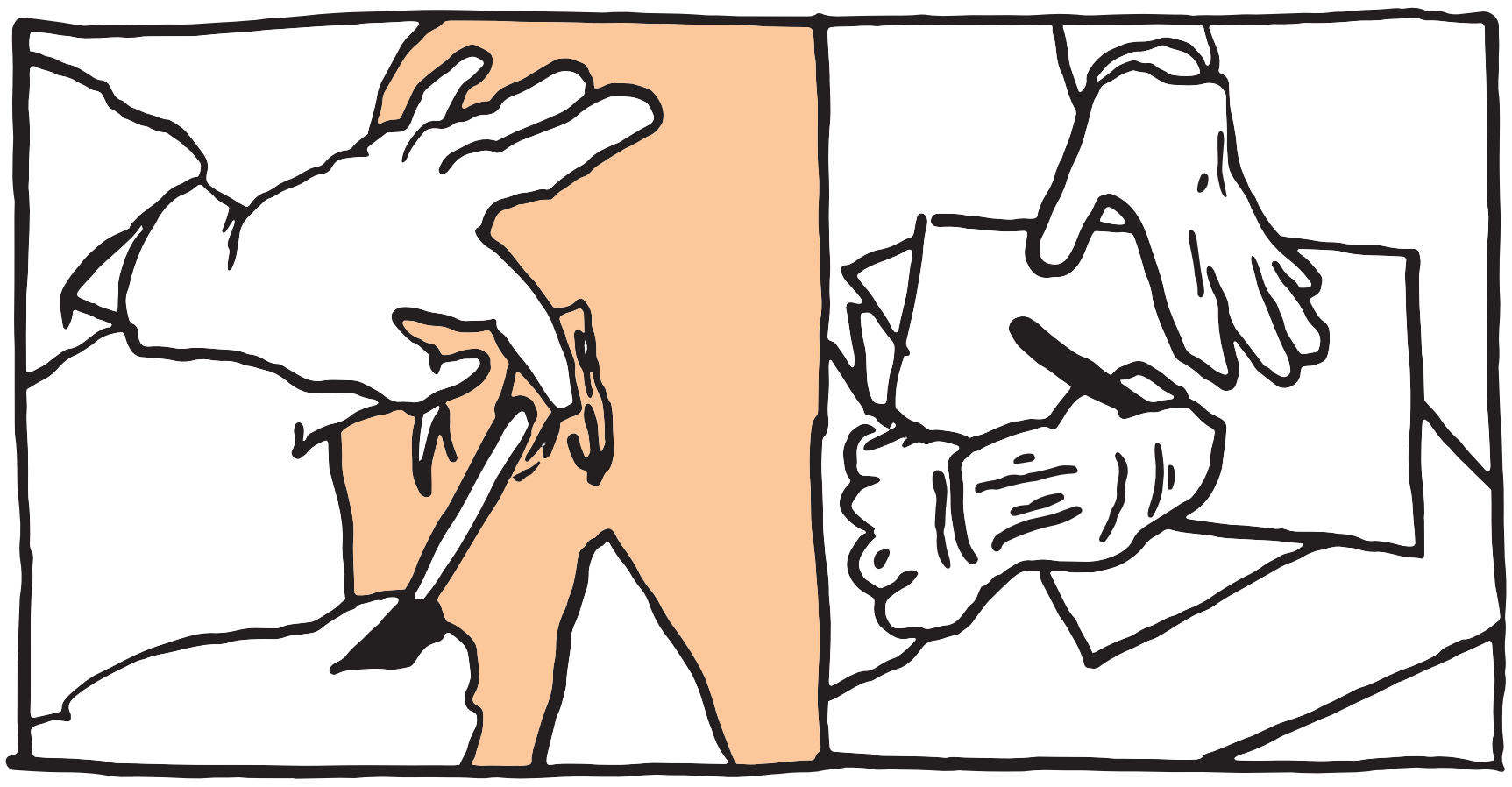

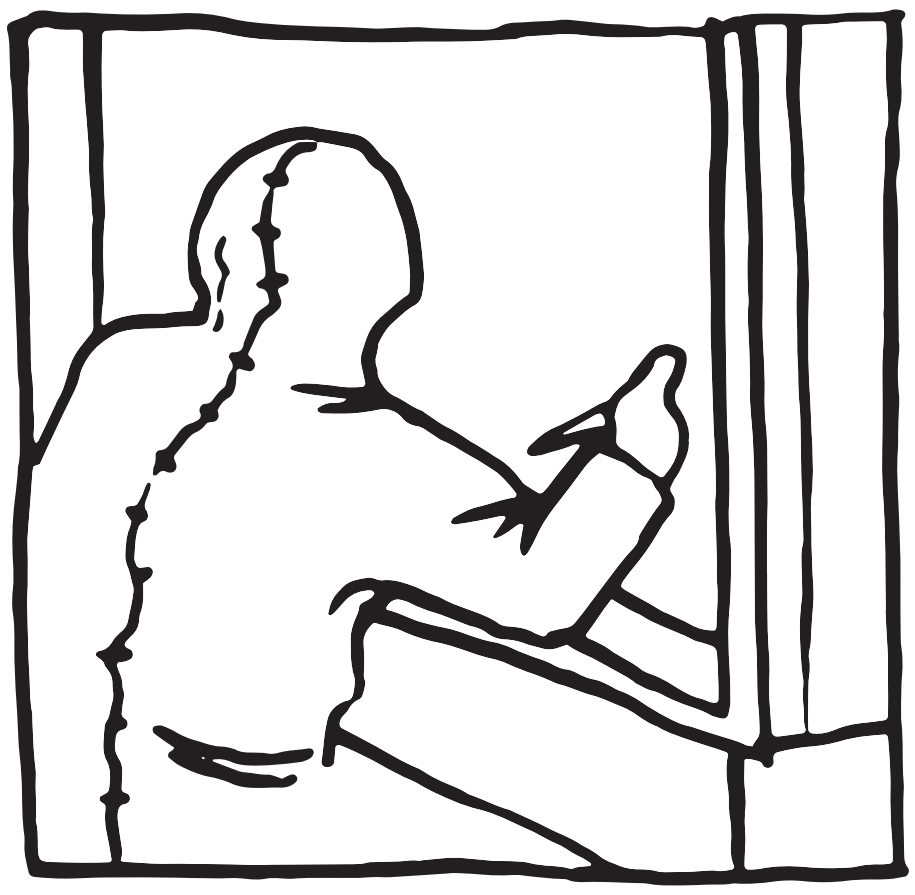

A few weeks later, the results came in for all the farms...

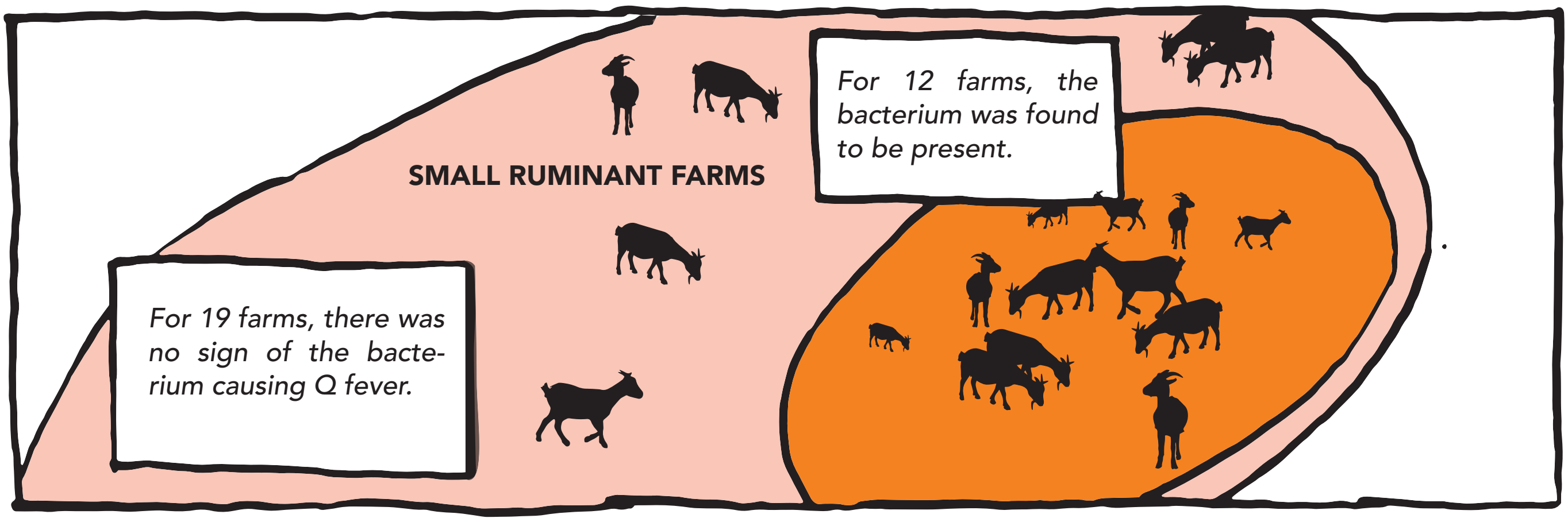

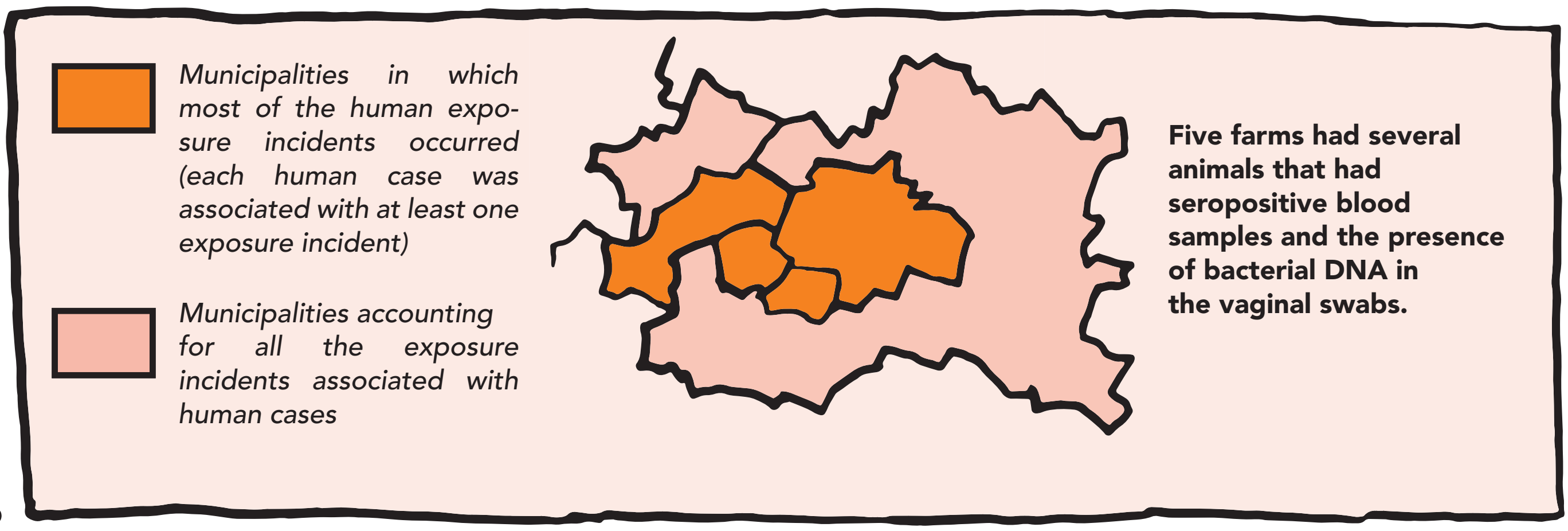

Several meetings were held to discuss the situation with experts and to decide on management measures; they involved all public health organisations and agencies...

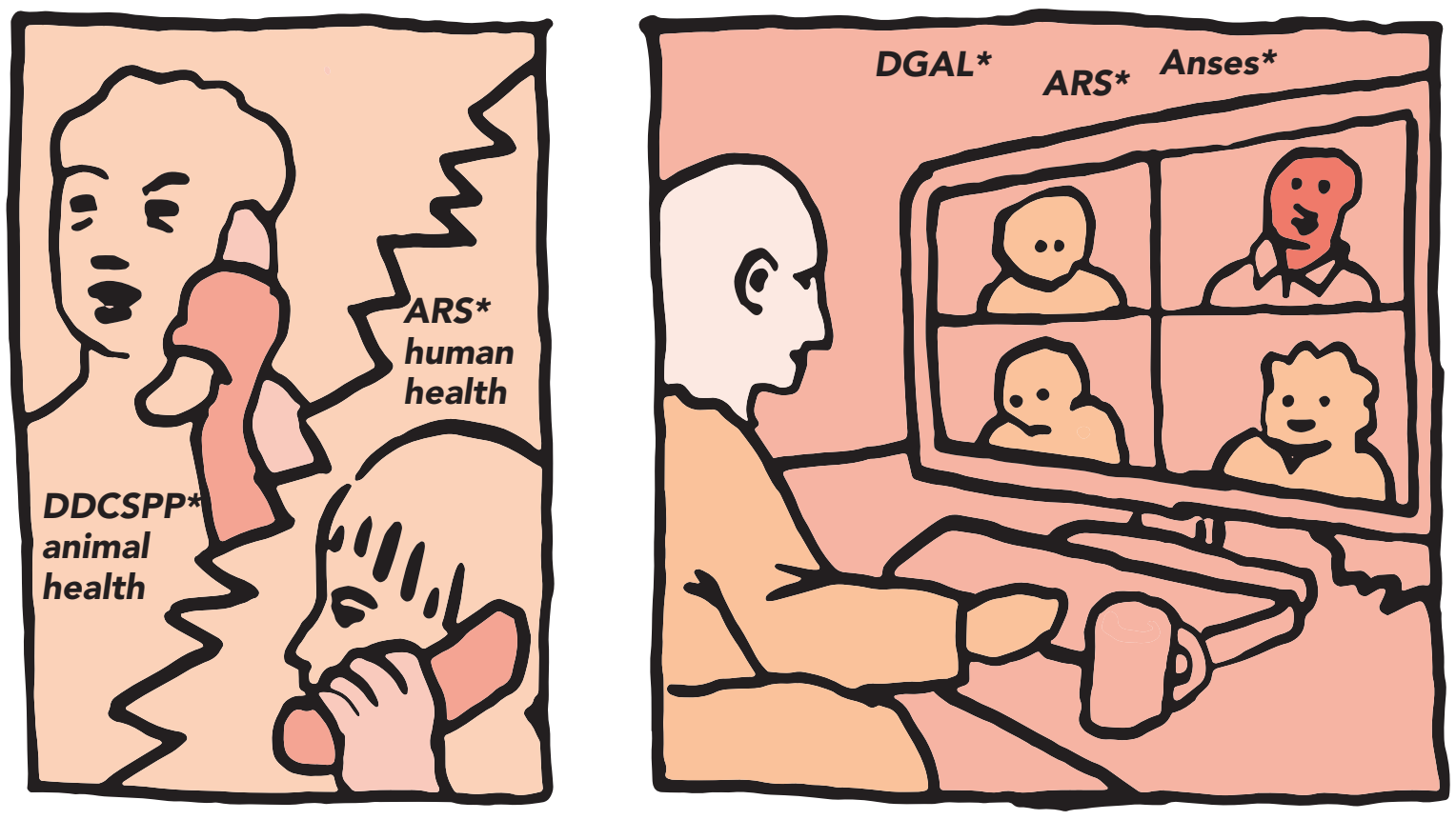

A meeting was held between certain local stakeholders and representatives from various public health agencies.

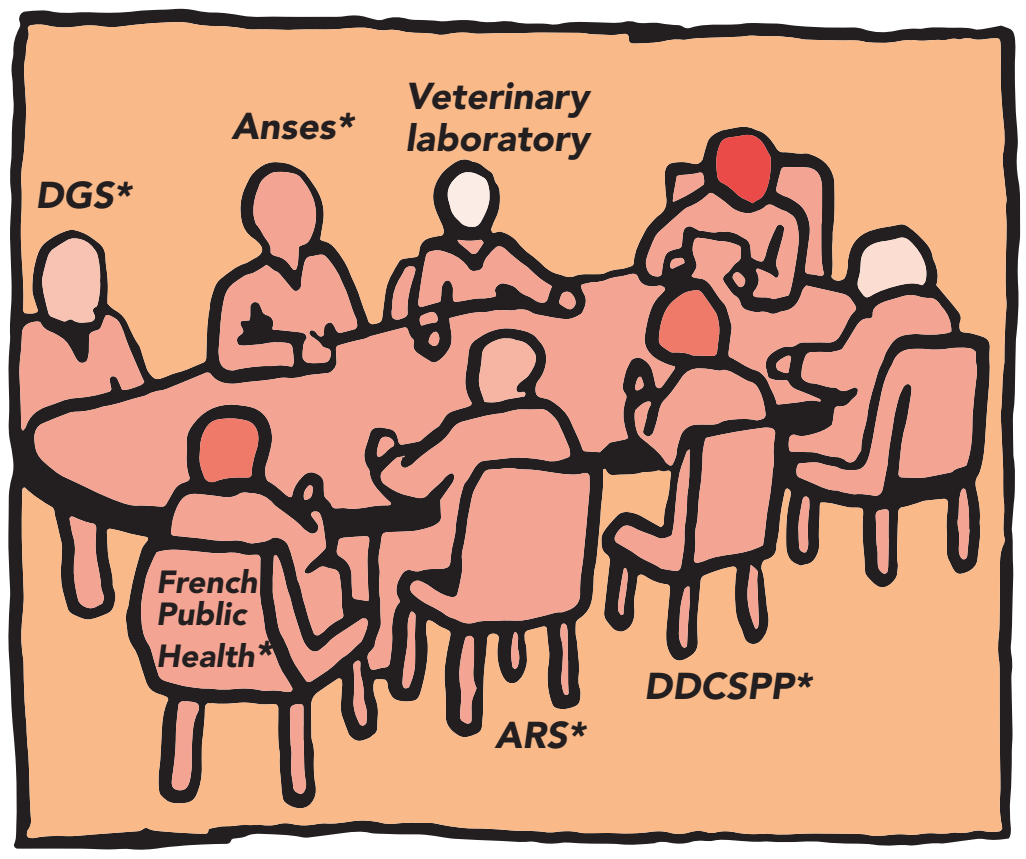

The regional health agency (ARS*) sent information memoranda to general medical practitioners and hospitals.

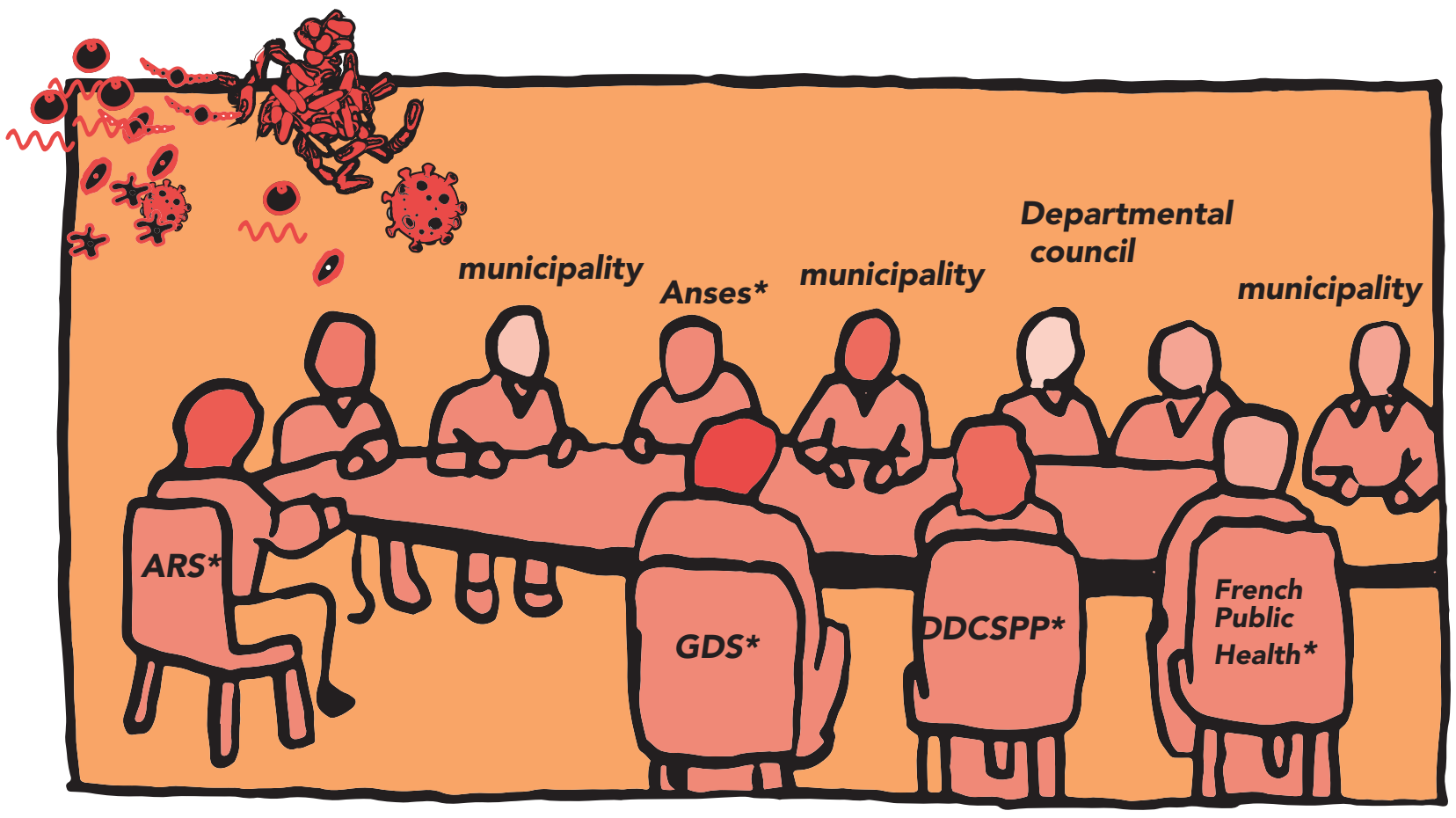

Local stakeholders contributed to OSCAR*, the national abortion surveillance system.

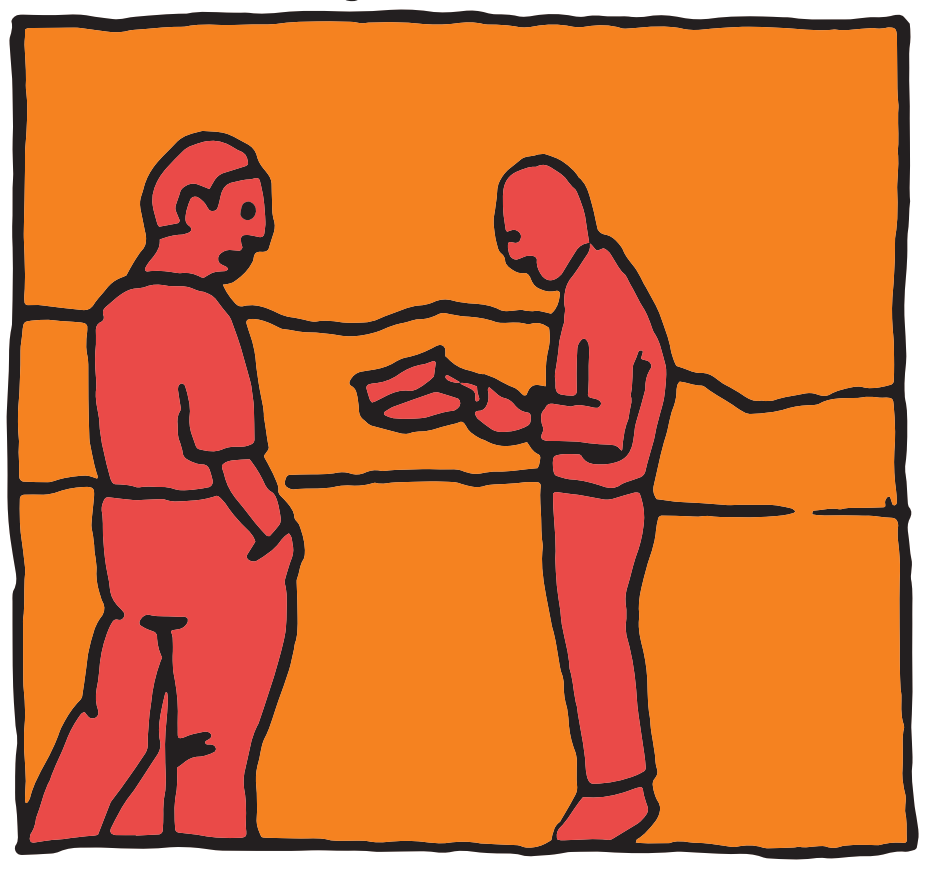

Letters were sent to farmers falling under the scope of the investigation.

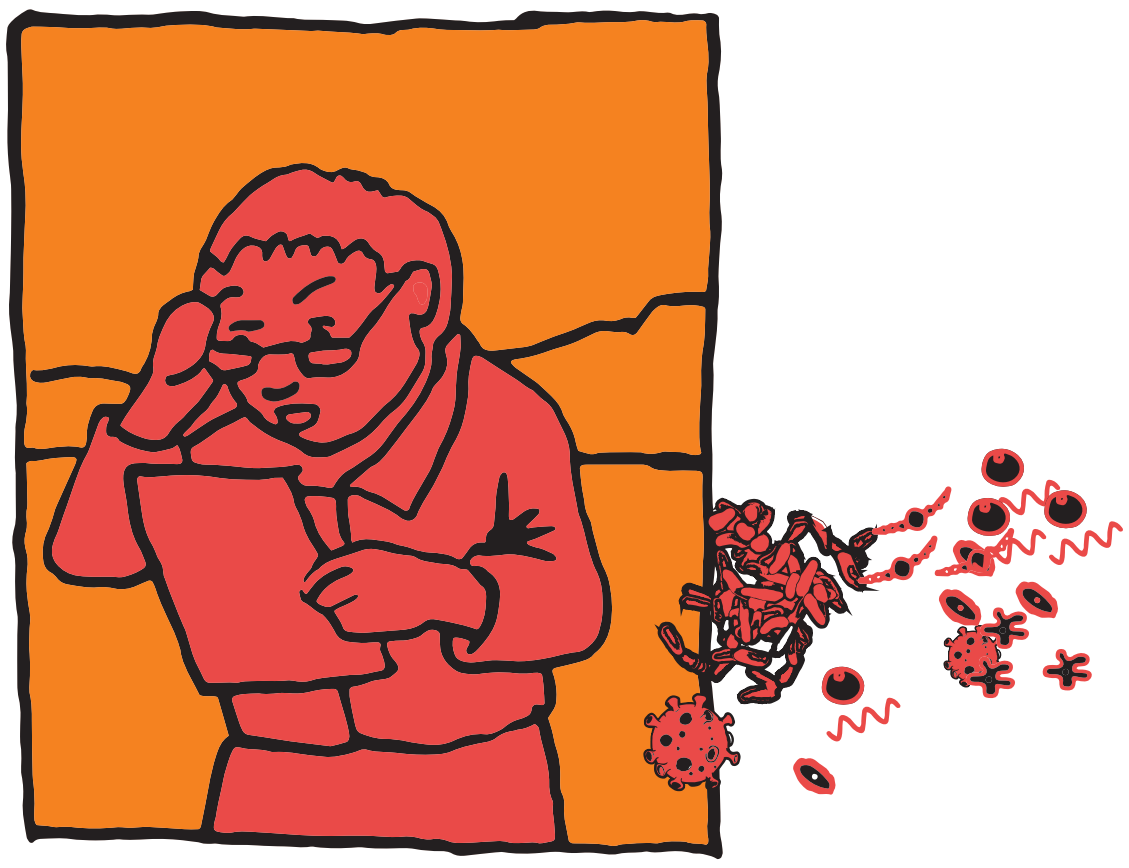

"...We encourage you to talk to your vet about vaccinating your herd against Q fever."

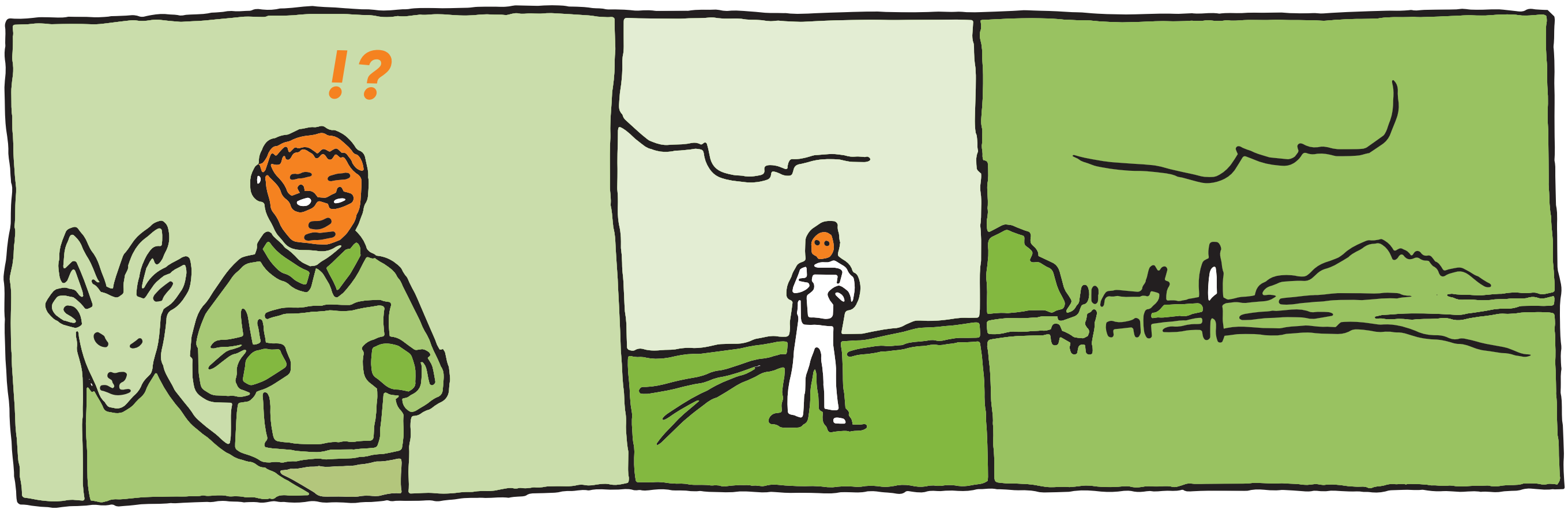

* *Acronyms for various human and animal health management institutions in France*

Public action
in response to a disease outbreak

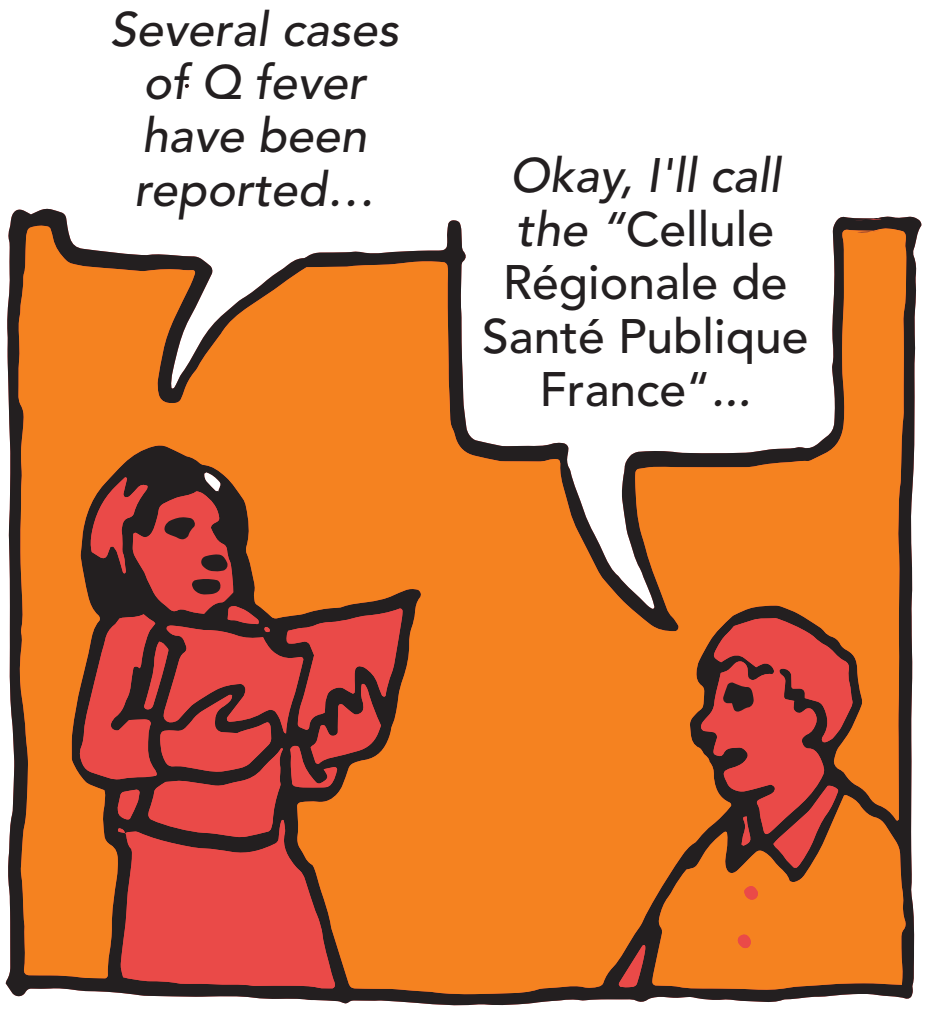

Management measures are taken only when the disease goes beyond its normal scope and affects human health to a level high enough to trigger the alert unit.

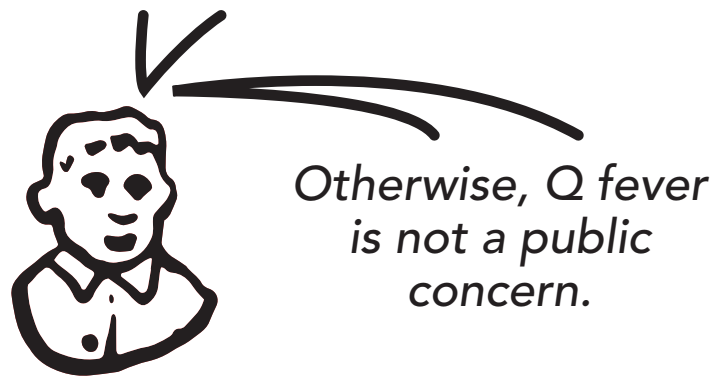

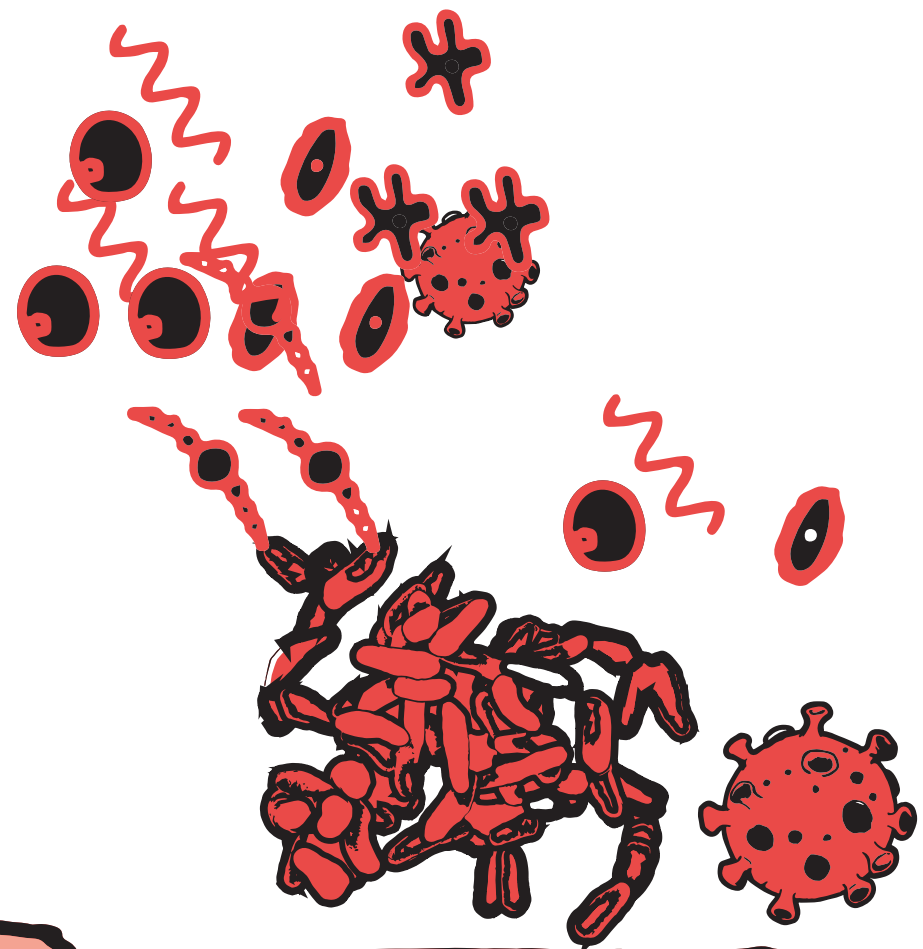

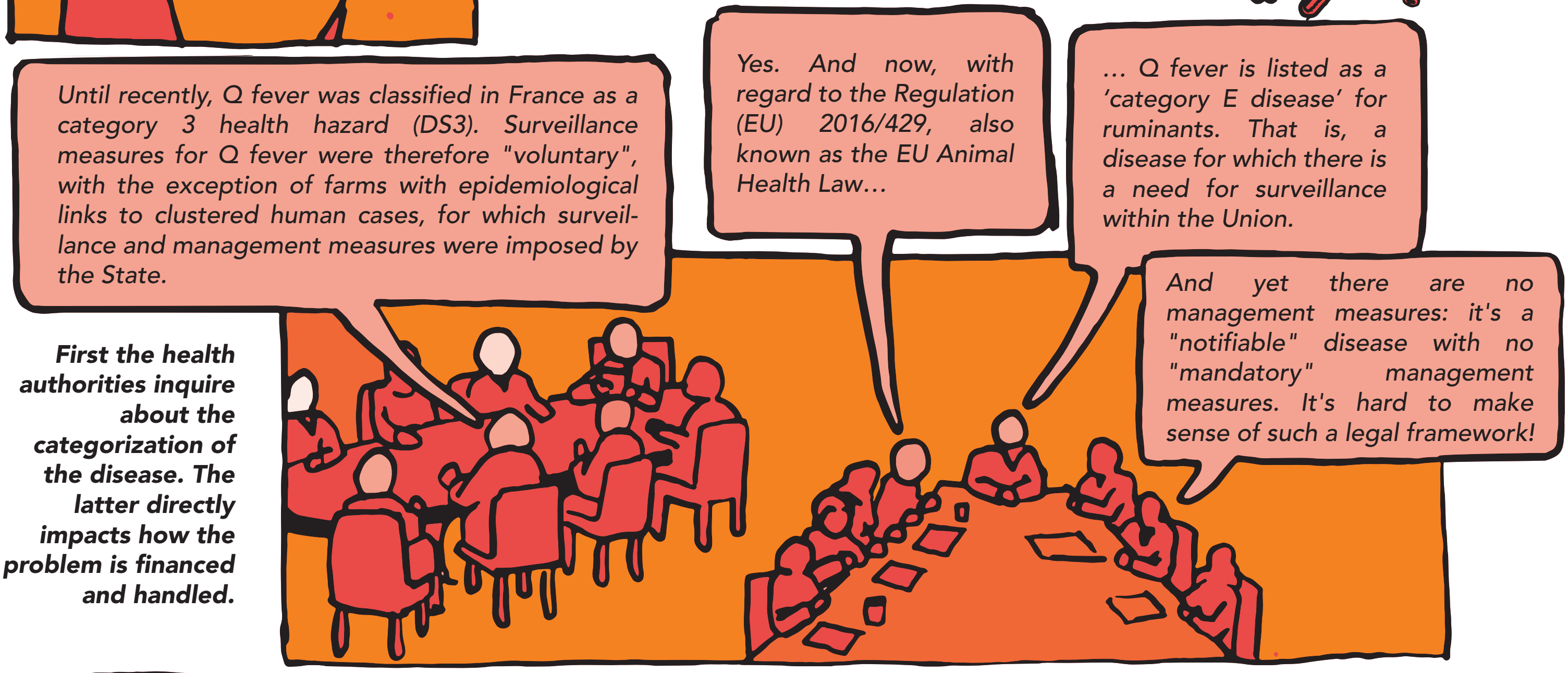

First the health authorities inquire about the categorization of the disease. The latter directly impacts how the problem is financed and handled.

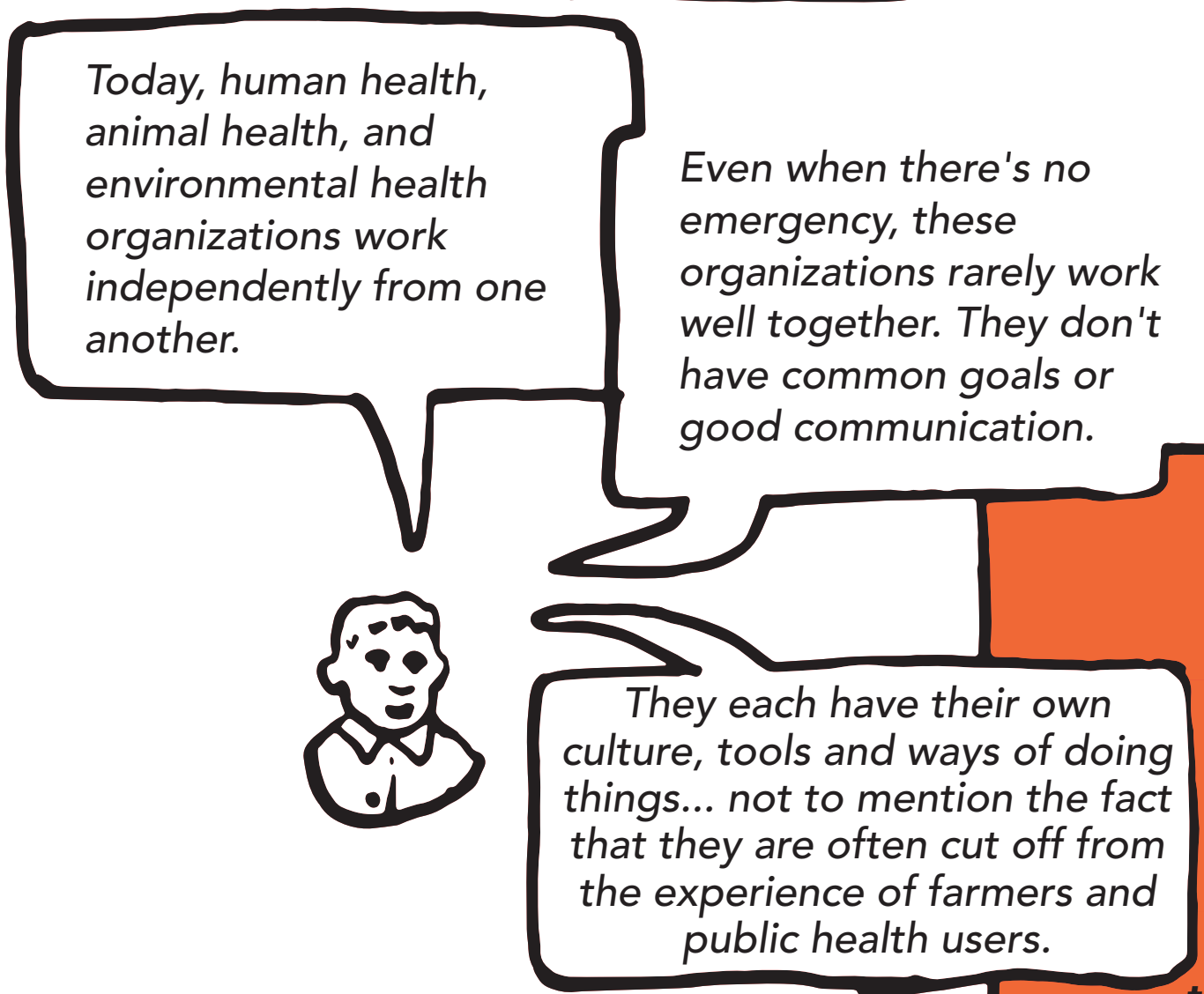

In other words:
(1) there are many public stakeholders and they work in very different ways. They use different tools and respond to different health issues.
(2) It's impossible to deal with every health issue, because of a lack of resources, insufficient coordination between the people involved and, sometimes, limited knowledge…

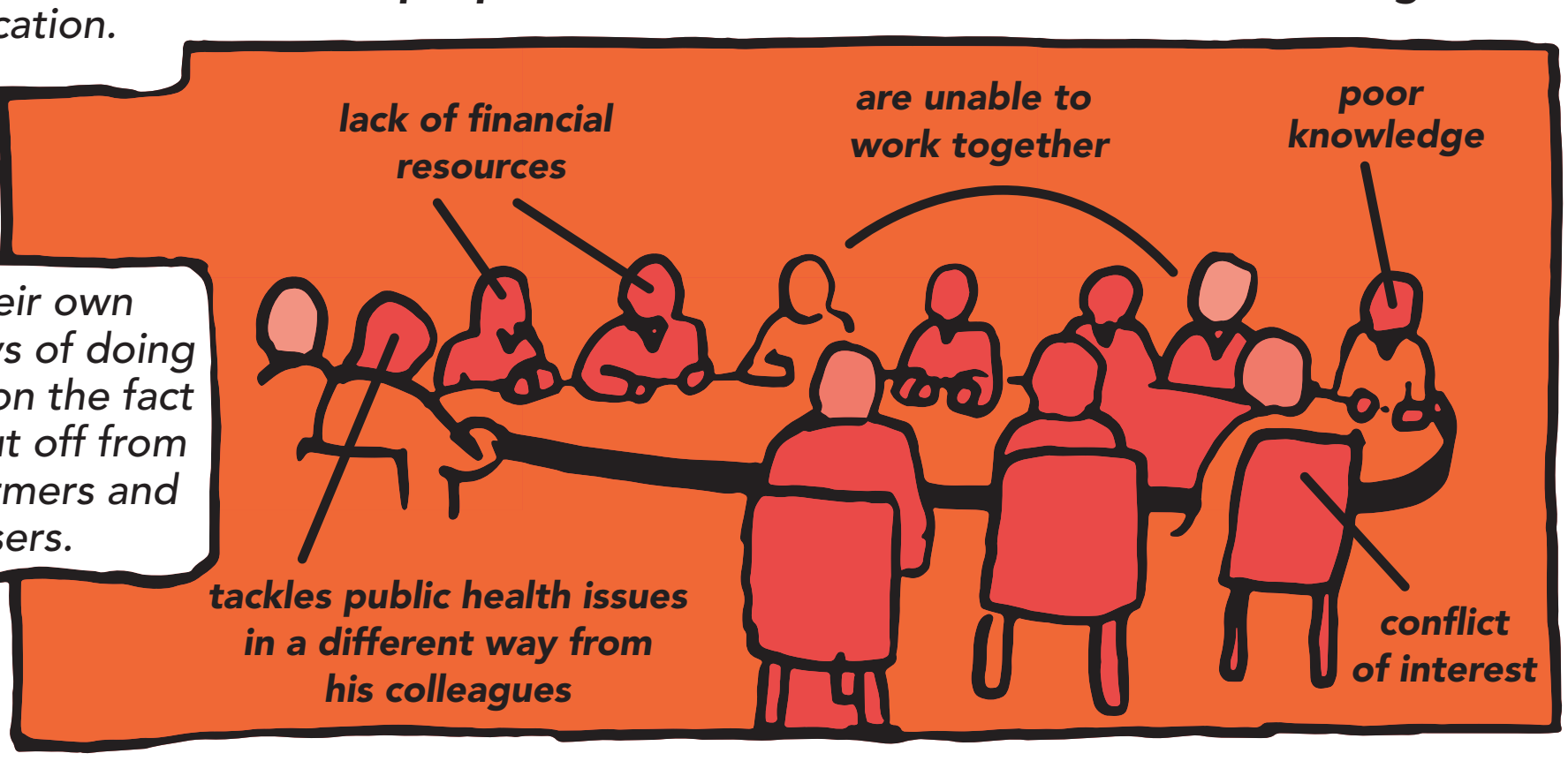

Integrating all existing organizations into a smooth-running "One Health" system is a complex task.

when the disease comes to the attention of small ruminant farmers

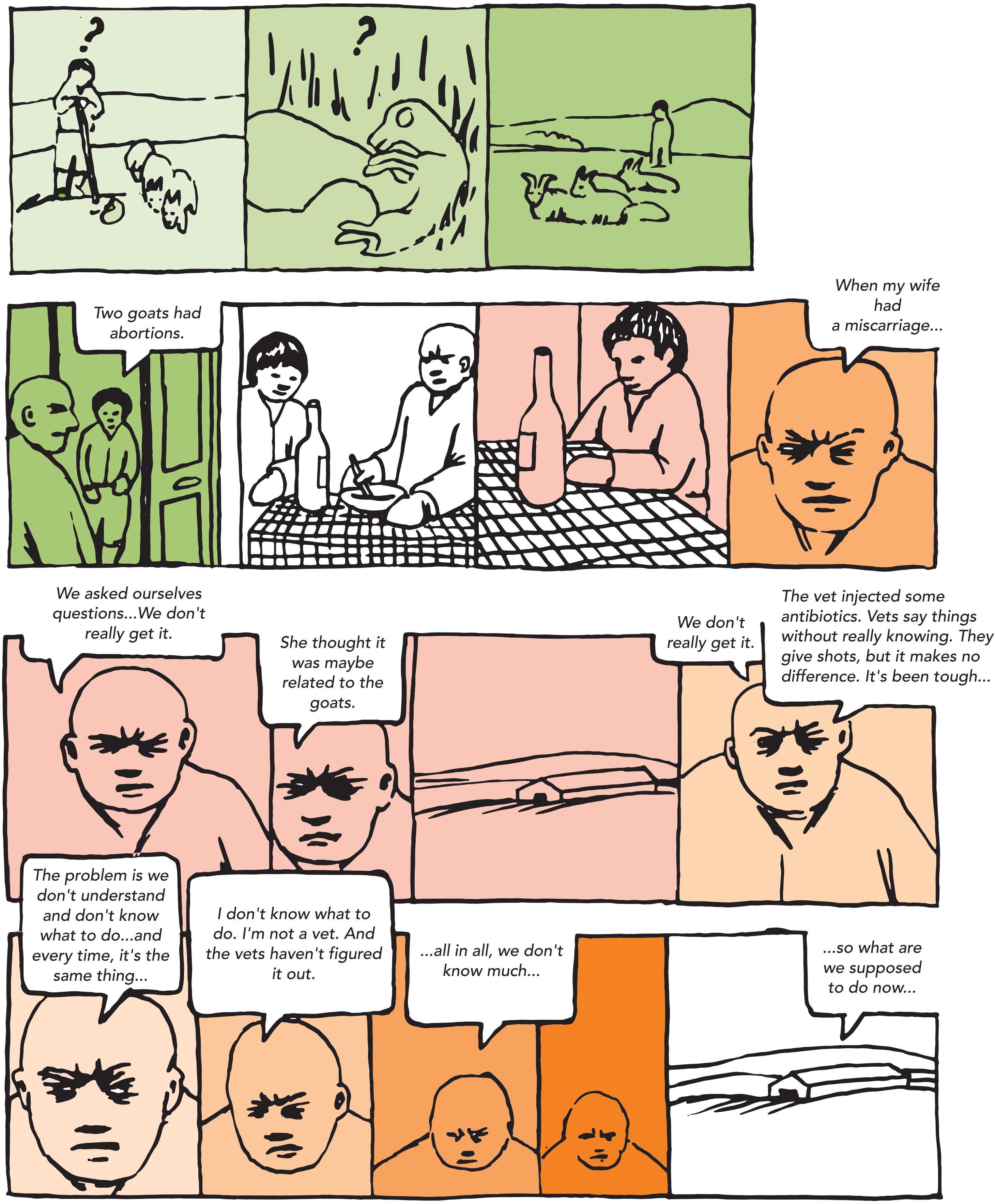

responding to disease on the farm
two examples of Q fever experience

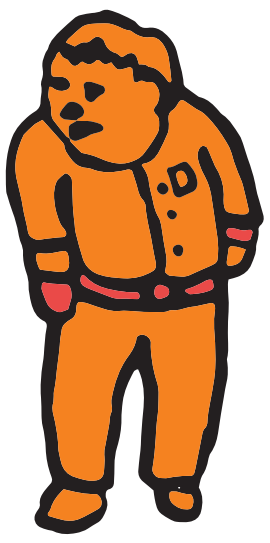

Exemple 1 : *Farm with risk management largely delegated to experts and public health action*

The characters presented here do not exist in real life: they have been composed from phrases collected from various farmers.

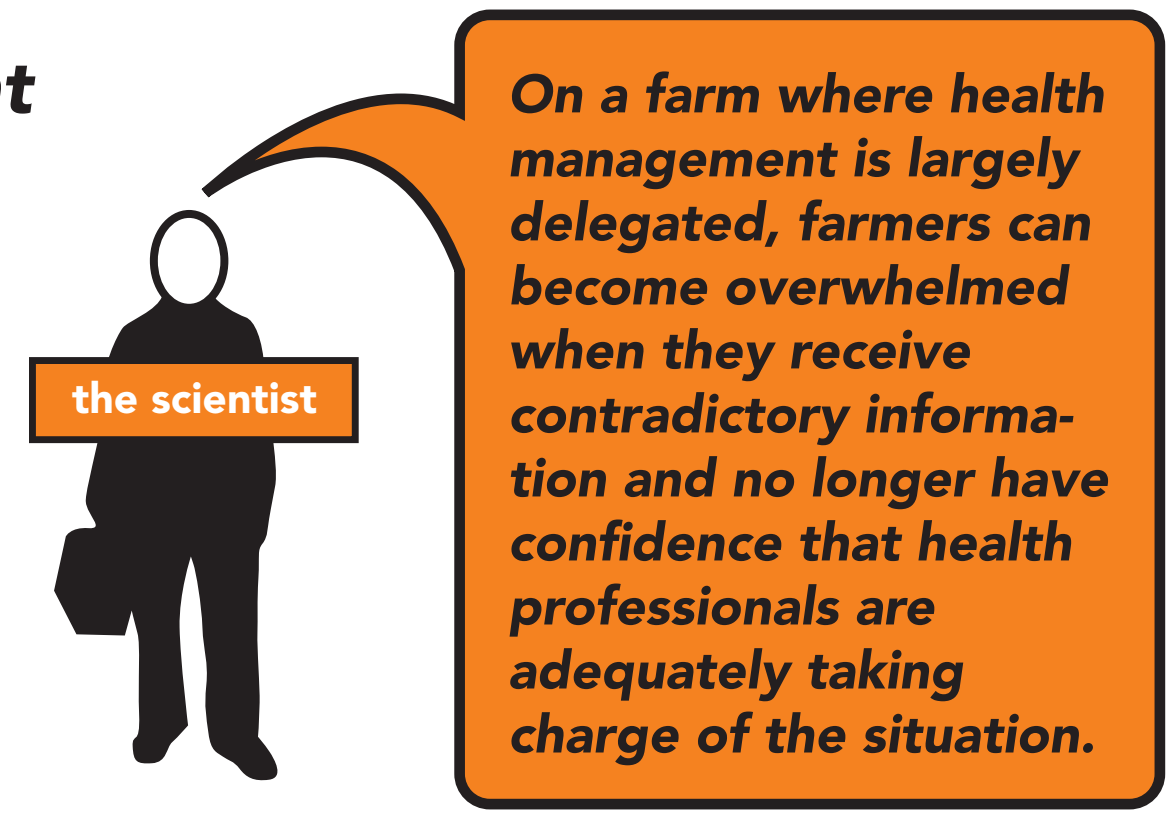

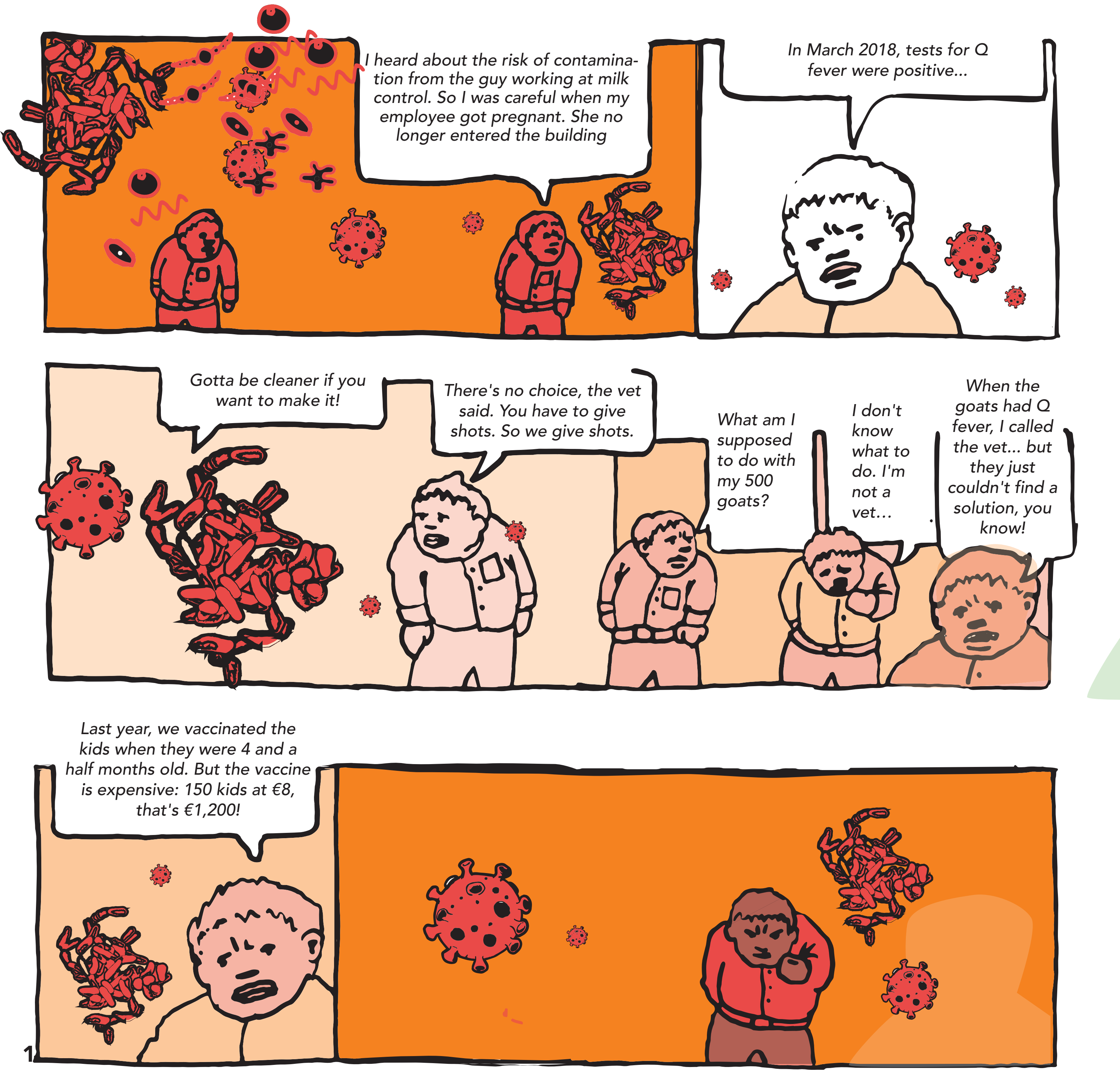

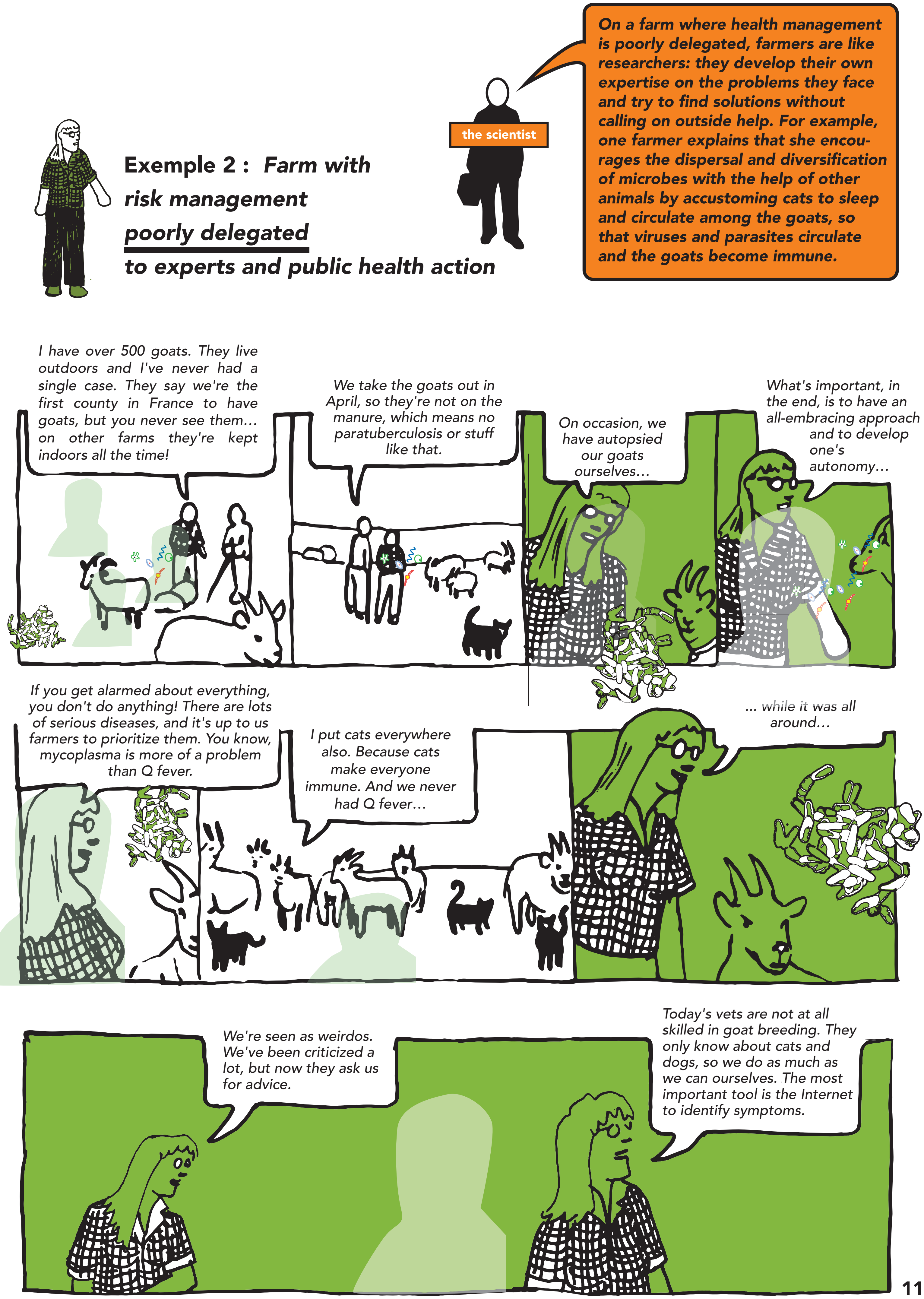
Exemple 2 : Farm with risk management poorly delegated to experts and public health action
the scientist
On a farm where health management is poorly delegated, farmers are like researchers: they develop their own expertise on the problems they face and try to find solutions without calling on outside help. For example, one farmer explains that she encourages the dispersal and diversification of microbes with the help of other animals by accustoming cats to sleep and circulate among the goats, so that viruses and parasites circulate and the goats become immune.
I have over 500 goats. They live outdoors and I've never had a single case. They say we're the first county in France to have goats, but you never see them… on other farms they're kept indoors all the time!
We take the goats out in April, so they're not on the manure, which means no paratuberculosis or stuff like that.
On occasion, we have autopsied our goats ourselves…
What's important, in the end, is to have an all-embracing approach and to develop one's autonomy…
If you get alarmed about everything, you don't do anything! There are lots of serious diseases, and it's up to us farmers to prioritize them. You know, mycoplasma is more of a problem than Q fever.
I put cats everywhere also. Because cats make everyone immune. And we never had Q fever…
… while it was all around…
We're seen as weirdos. We've been criticized a lot, but now they ask us for advice.
Today's vets are not at all skilled in goat breeding. They only know about cats and dogs, so we do as much as we can ourselves. The most important tool is the Internet to identify symptoms.

and what do we do with all this?

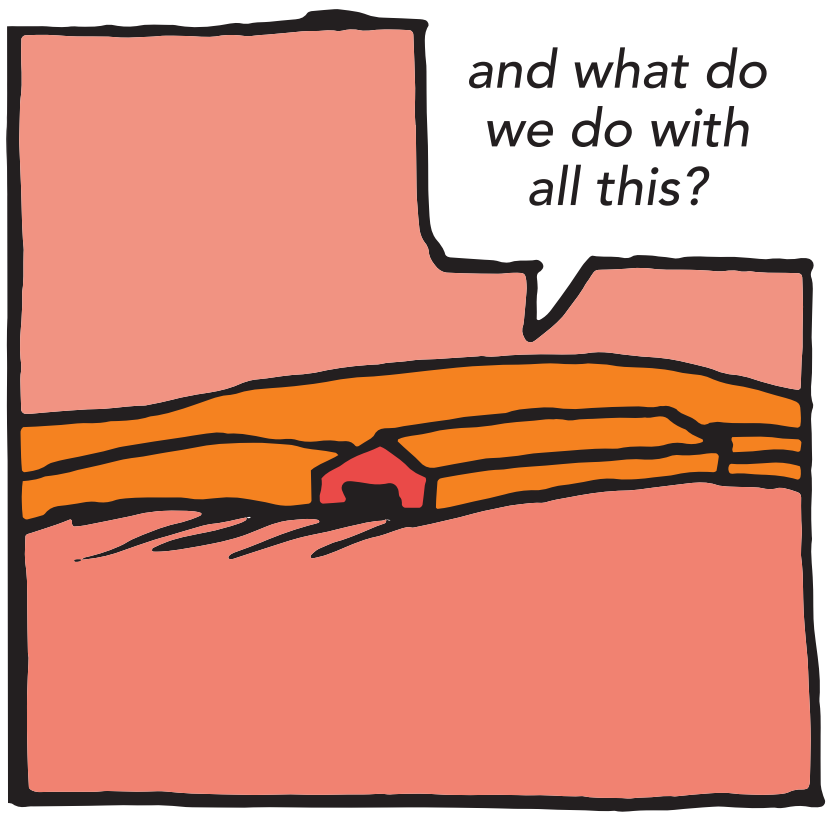

***Here**, there are men and women who hold meetings and try to answer, at the same time, questions about regulations and public health, including that of residents, animals and ecosystems.*

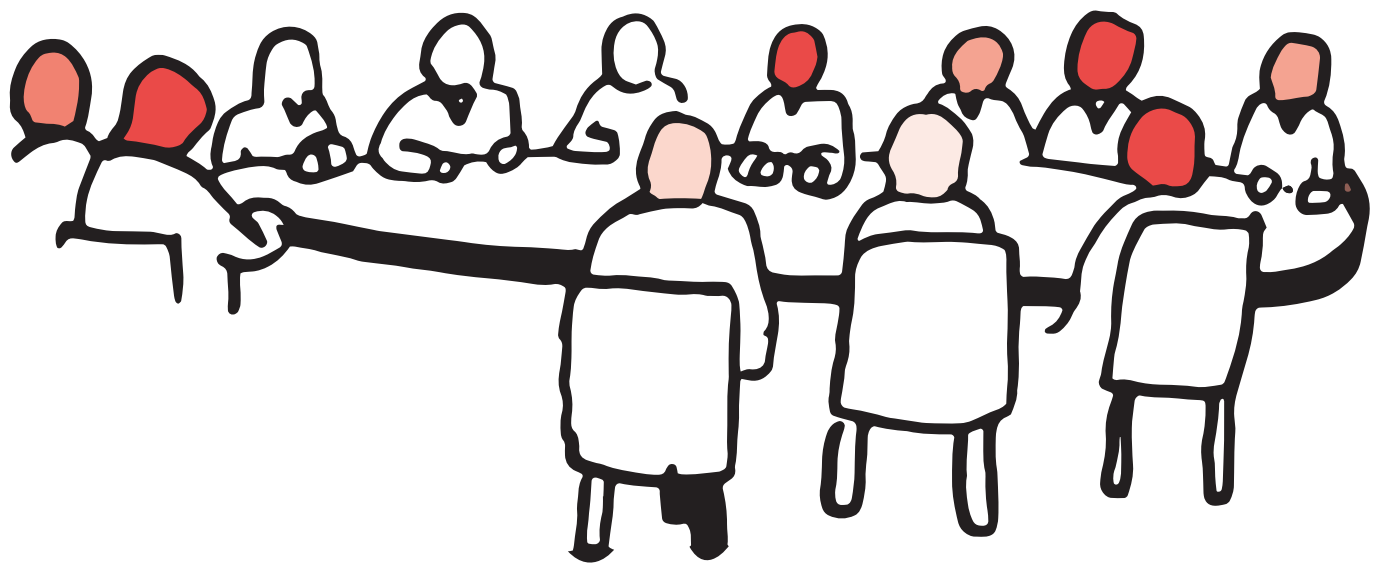

***Hereabout**, there are farmers who delegate their health problems... without always finding a solution.*

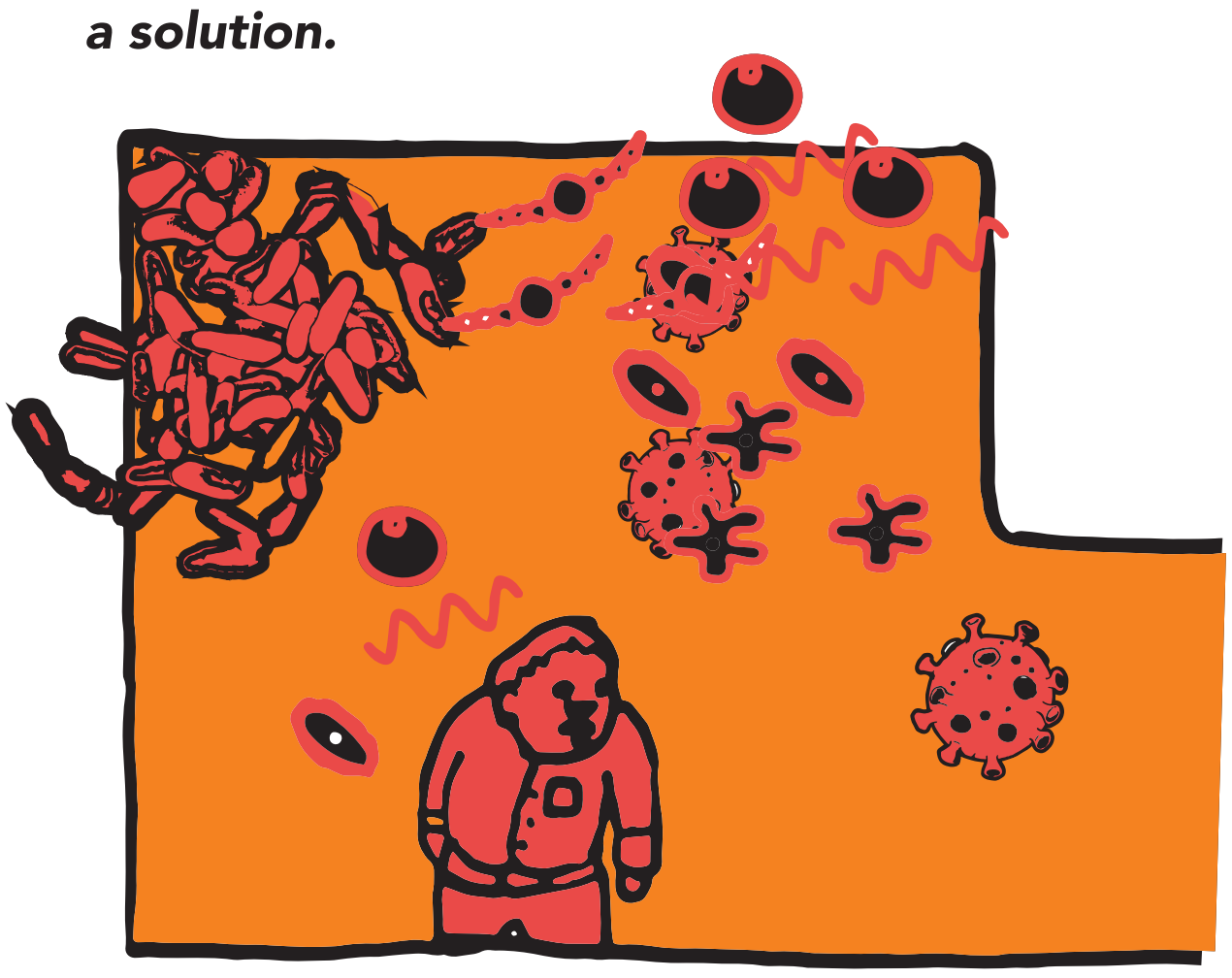

***There**, doctors are treating (or not) patients with Q fever.*

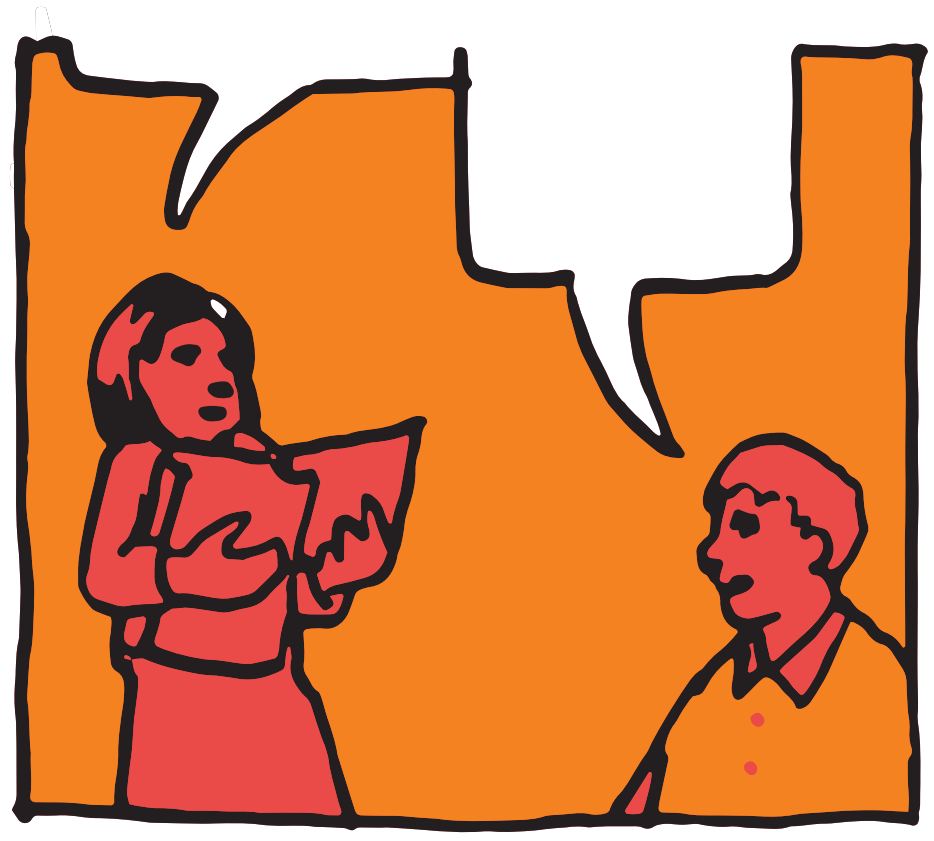

***Here**, farmers are (re)learning how to manage on their own.*

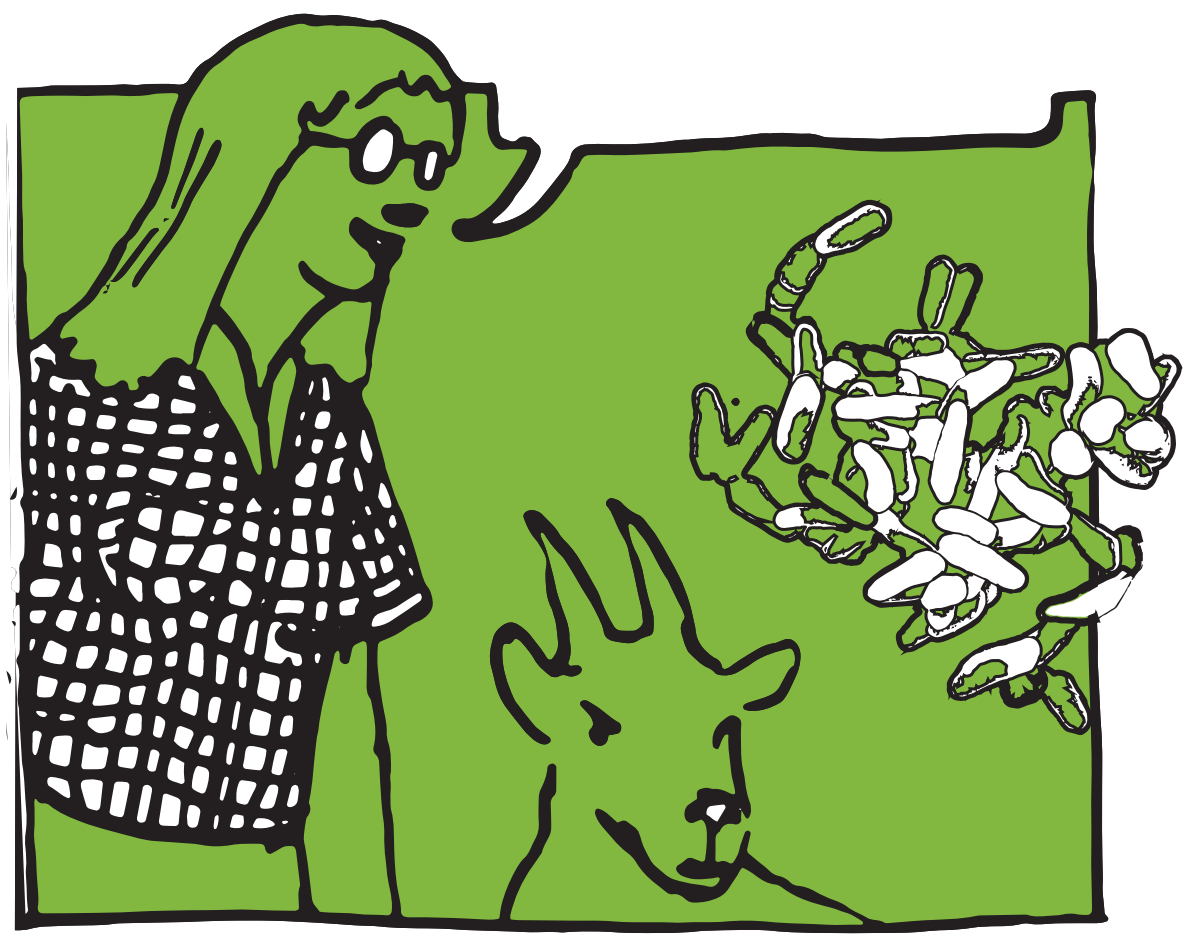

***Over here**, researchers are trying to understand the risk factors for transmission to humans.*

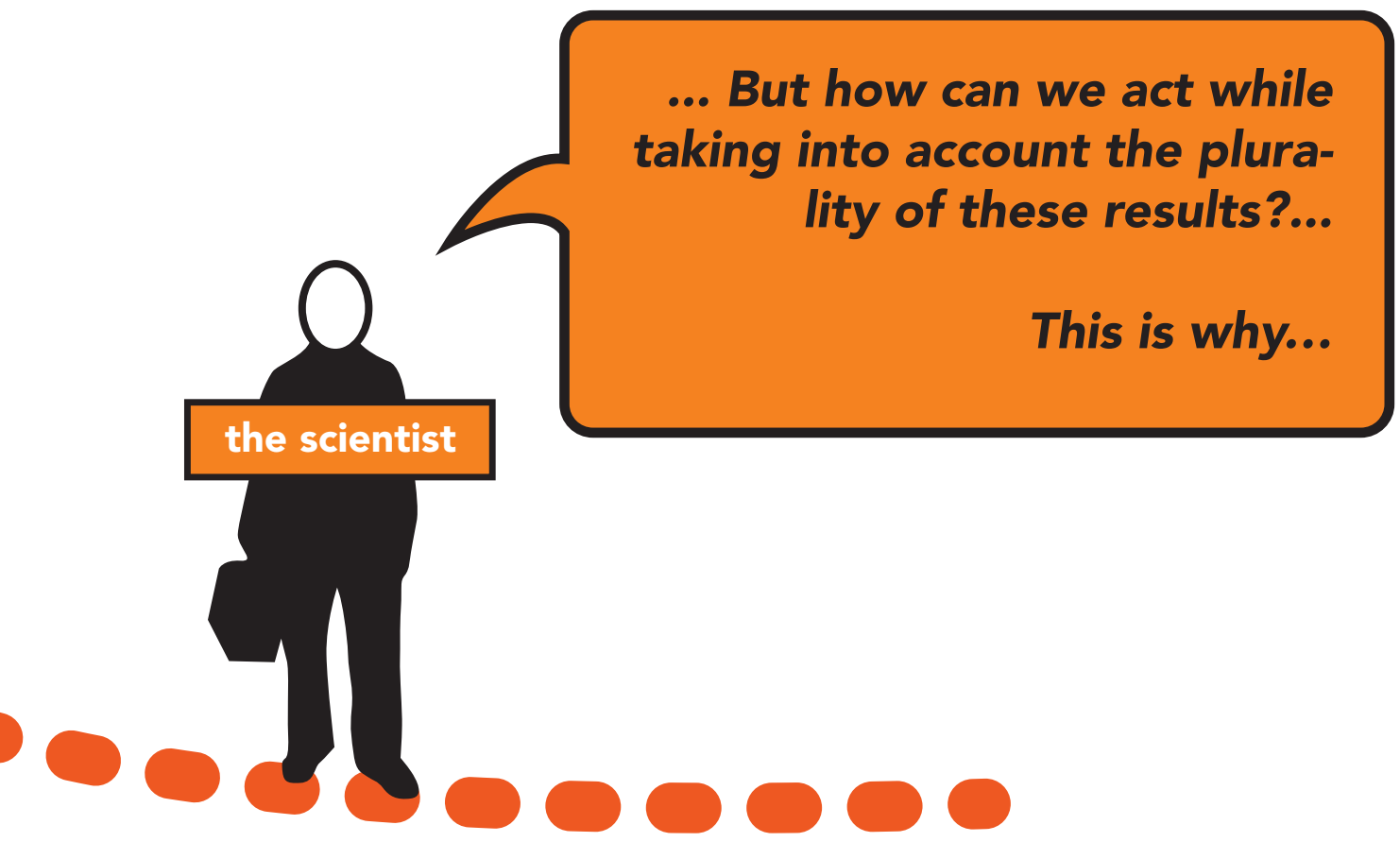

... We meet up and consider what each of us is doing and why we're doing it the way we are.

... And we try to do better together

References

• Ramillien E, Cayre P, Fourt X, Rousset E, Jourdain E. Q fever as an anthropological prism for revealing how farmers live with microbes Agriculture and Human Values 2025;42(1):527-43.

• Demota F., Dezempte J.-C., Godiot C., Grassin A., Hinoux V., Lesellier C., Sundermann T. Q fever and institutional stakeholders: perceptions, practices, and knowledge [in French]. Student Report. Master 2 : Politiques publiques et gouvernements comparés. Institut d'études politiques de Lyon – École Nationale des Services Vétérinaires – France Vétérinaire International. Groupe d'Études des Politiques Publiques. Mach 2022. 103 pages.

• Bernadou A., Lambert Y., Gache K. Clustered cases of Q fever, Niort hospital, April-May 2017 [in French]. Saint-Maurice: Santé publique France, 2019. 30 pages. Available from: https://www.santepubliquefrance.fr/regions/nouvelle-aquitaine/documents/rapport-synthese/2019/cas-groupes-de-fievre-q-dans-le-pays-niortais-avril-mai-2017

Acknowledgements

We thank Émilie Ramillien and Élodie Rousset, who helped design the ZOOJEU system and implement its use with stakeholders in Prahecq (Niort urban area) on May 4, 2023; Emilie Wimmer-Bonneau, Denis Boulenger, Karine Monnet, Corinne Dempuré, Nathalie Hucteau, Sofia Mlala, and the students earning their BTS in animal production at Jacques Bujault High School, who contributed to system testing on April 5, 2024 in Melle; all the stakeholders in the goat industry and the Niort urban area who took part in the anthropological surveys and participatory science days as part of the ZOOJEU project; and all those who contributed in any way to the success of this project.

Project funding was provided by
- **the Regional Public Health Agency of Nouvelle-Aquitaine**
- **INRAE's General Directorate for Expertise and Support for Public Policies**

To learn more:

- https://epia.clermont.hub.inrae.fr/sites-web/zoojeu
- https://epia.clermont.hub.inrae.fr/faits-marquants/zoojeu
- https://ludotheque.gamae.fr/game/117

• **Contacts**

Elsa Jourdain : elsa.jourdain@inrae.fr
Xavier Fourt (artists group Bureau d'études) : xavierfourt2025@gmail.com
Patrice Cayre : patrice.cayre@resothem.fr
