# Supplementary material for: ZOOJEU: Using Q fever to highlight a participatory science approach for fostering the co-design of management strategies for neglected zoonotic diseases
Source: PLoS Negl Trop Dis. 2026 Oct 15;20(10):e0014618. doi: 10.1371/journal.pntd.0014618 (PMC13671574; doi:10.1371/journal.pntd.0014618)
Supplement: Supplementary file 2 [file pntd.0014618.s006.pdf]

ZOOJEU

jeudi 4 mai 2023
déroulement de la journée

faire face aux zoonoses du point de vue des éleveurs

une expérimentation proposée par le collectif de recherche Zoojeu pour regarder ce que fait chacun, réfléchir ensemble sur la possibilité d'une co-construction de politiques publiques sanitaires avec les éleveurs

en prenant pour exemple la prévention et la gestion de la fièvre Q dans un territoire concret (le territoire niortais) et en tenant compte de la diversité des points de vue des éleveurs

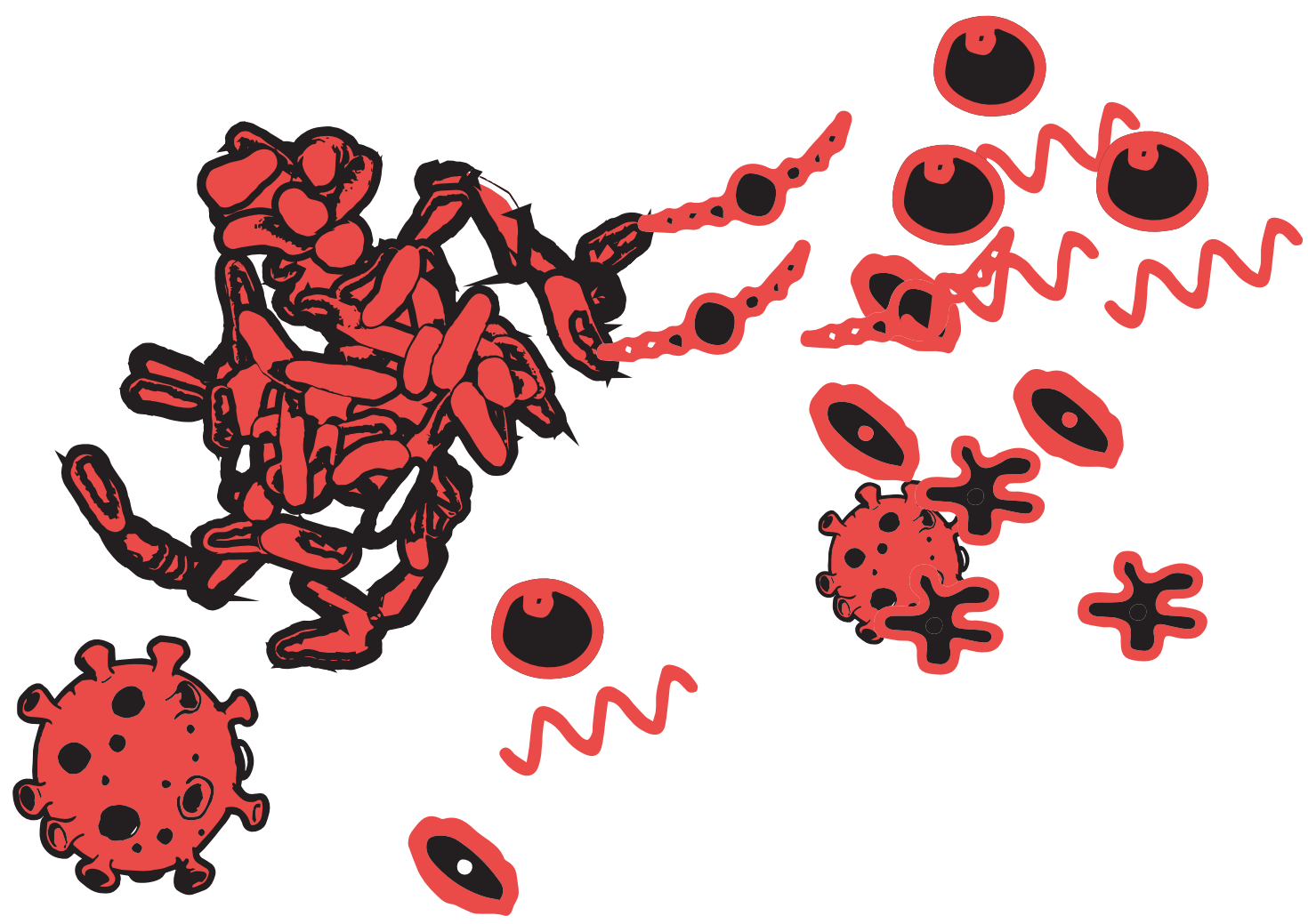

Le présent document montre le déroulement de la journée d'expérimentation collective. Il rend compte de toutes les choses dont on fait usage quand on a à faire avec la fièvre Q. Il rend compte donc de tout ce dont nous allons faire usage ensemble, au cours de la journée, pour comprendre ce que chacun fait, et pourquoi il le fait comme il le fait. Pour cette journée, **le parti pris est de s'intéresser au point de vue des éleveurs,** qui sont directement affectés par la fièvre Q ; les autres acteurs concernés (action publique, réglementation, recherches,...) seront donc abordés du point de vue des éleveurs.

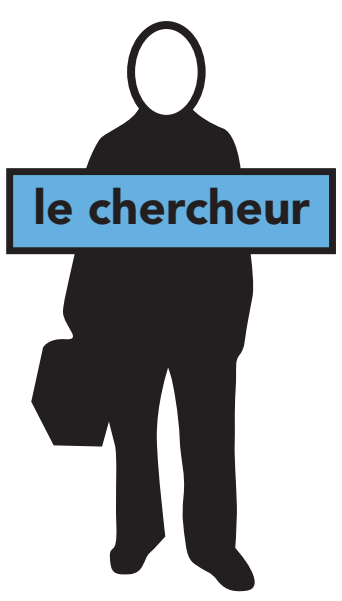

Le collectif de recherche Zoojeu est composé d'une anthropologue *(Emilie Ramillien, anthropologue au laboratoire de recherche associatif Origens Media Lab),* **d'un designer** *(Xavier Fourt, designer de recherche et d'action publique, membre du collectif d'artistes Bureau d'études),* **d'une épidémiologiste** *(Elsa Jourdain, vétérinaire épidémiologiste à INRAE),* **d'un sociologue** *(Patrice Cayre, sociologue membre du collectif Reso'them de la DGER, rattaché à l'UMR Territoires et à Origens Media Lab),* **d'une microbiologiste** *(Élodie Rousset, responsable du Laboratoire National de Référence de la fièvre Q animale à l'Anses).*
Les membres du collectif ZOOJEU n'ont aucun conflit d'intérêt.

matinée introduction générale

Réfléchir ensemble à ce qui protège les chèvres et les humains de la fièvre Q à la ferme

Principes de la journée
Chacun est libre de se positionner comme il/elle le souhaite et personne n'a de compte à rendre sur ce qu'il/elle va dire ou faire au cours de la journée. L'objectif de cette mise en scène est de réfléchir collectivement à des scénarios et des positionnements possibles : il ne s'agit donc pas nécessairement de défendre ses positions personnelles ni les obligations telles qu'elles sont mises en œuvre dans ses activités mais d'explorer un univers des possibles. En revanche, chacun conserve sa position professionnelle.

9h / **accueil**

9h30 > 10h15 / **introduction**

Elsa Jourdain présente les résultats de l'enquête épidémiologique.

La bande-dessinée préalablement diffusée auprès des participants.es est présentée.

10h15 > 11h15 / Emilie Ramillien présente les résultat des enquêtes anthropologiques

Xavier Fourt présente les objets et le plateau de jeu

11h15 > 11h30 / pause

11h30 > 13h00 / jeux de plateau par groupes

13h00 > 14h00 / repas

14h00 > 15h00 / restitutions des jeux du matin

15h00 > 15h15 / pause

15h15 > 16h00 / jeux de scénarios par groupes

16h00 > 16h45 / échanges sur les scénarios joués

16h45- > 17h00 / clôture de la journée ZOOJEU

Retour sur l'enquête anthropologique

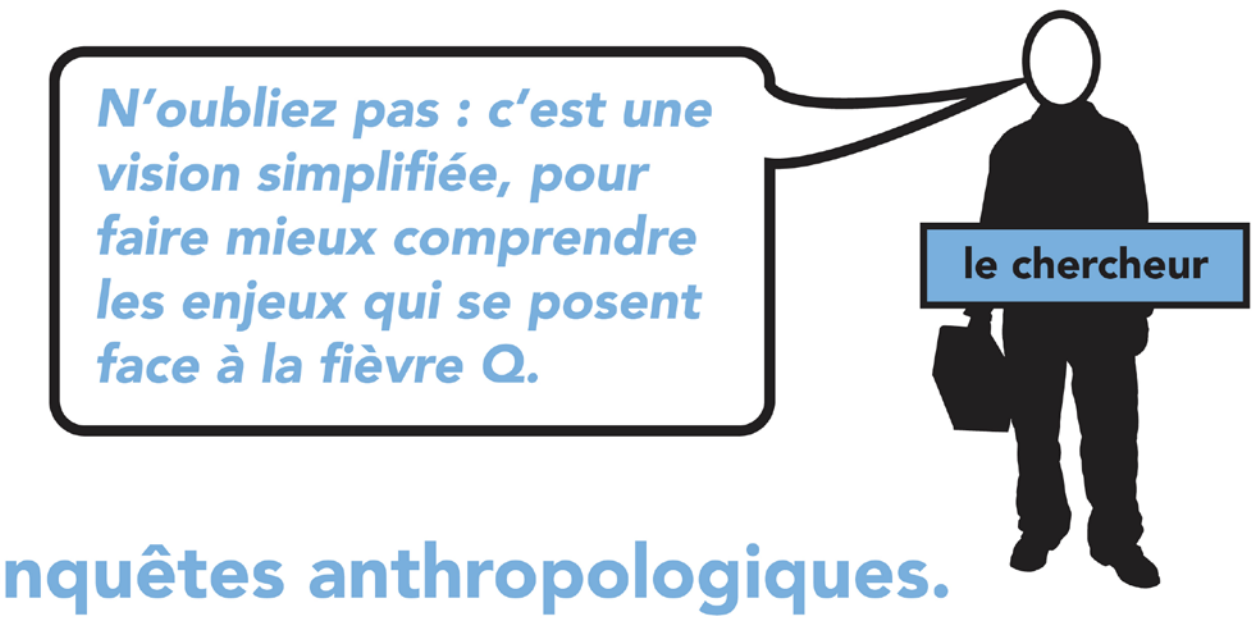

Émilie Ramillien présente les résultats des enquêtes anthropologiques.
Les éleveurs se perçoivent comme étant à la frontière entre deux mondes auxquels ils n'appartiennent jamais totalement et avec lesquels ils négocient des espaces de dialogue et des relations diplomatiques.

On définit deux fermes stéréotypées, l'une dans laquelle prédomine la relation au monde A (monde de la collaboration et du soin au vivant), l'autre dans laquelle prédomine la relation au monde B (monde de la gestion et de l'administration du vivant).

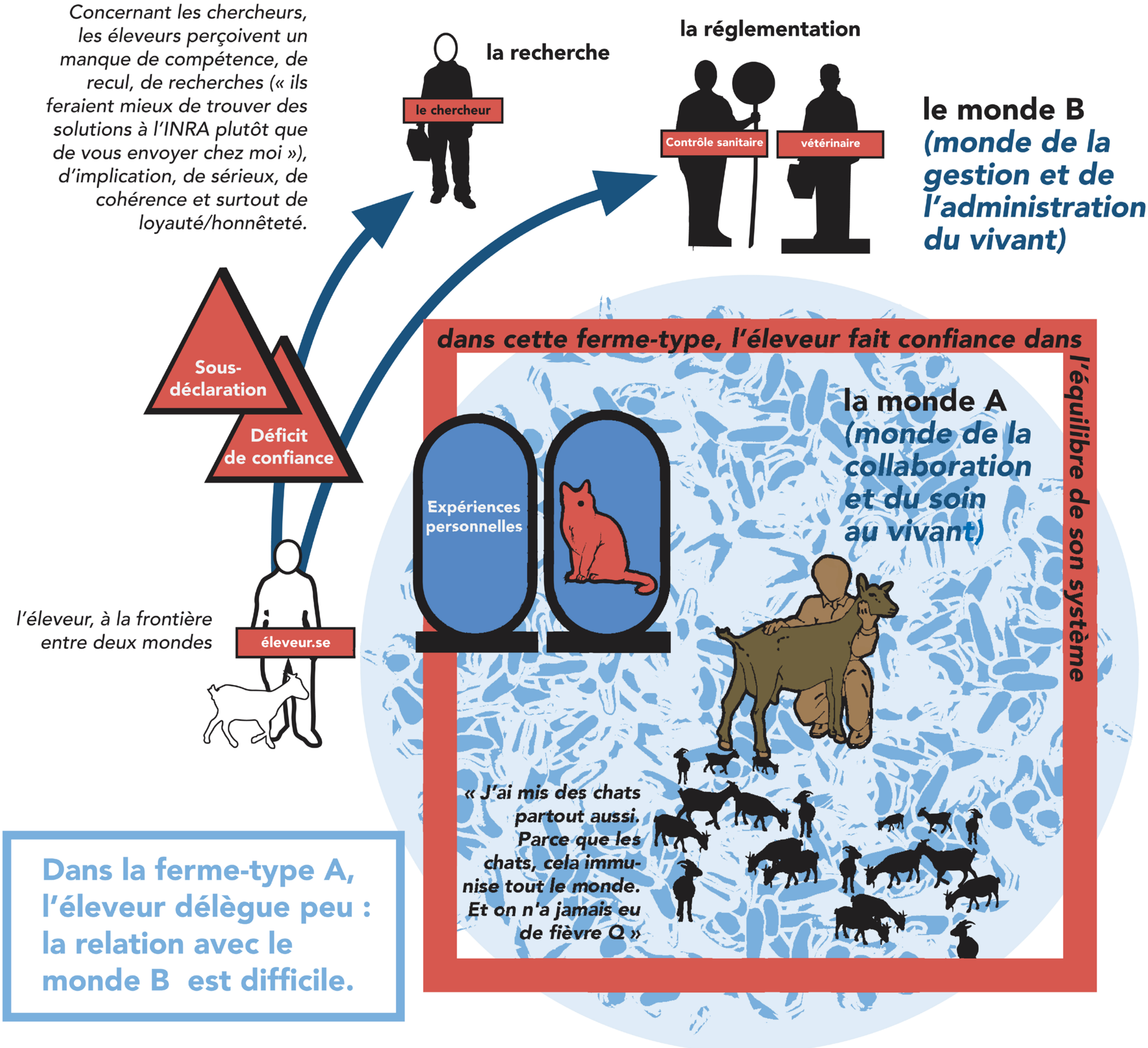

Dans la ferme-type A, l'éleveur délègue peu : la relation avec le monde B est difficile.

le monde A (monde de la collaboration et du soin au vivant)

Dans **le monde de la collaboration et du soin au vivant**, *la réalité est faite de temps longs, de cycles permanents où prédomine l'expérience sensible des choses qui ont toujours existé comme les animaux, les prés, les plantes, les mises bas, la mort, l'organique. Humains et non-humains s'y entremêlent, par le biais de relations complexes et difficiles (mais perçues comme authentiques et nourrissantes), fondées sur des interdépendances réciproques. C'est un monde où les causes des événements qui troublent les différents cycles sont obscures et complexes.*

le monde B (monde de la gestion et de l'administration du vivant)

Dans le **monde de la gestion et de l'administration du vivant,** la réalité repose sur les distinctions dualistes entre humains et non-humains, nature et culture, objectivation et subjectivité, etc. La réalité est vécue sur le mode du temps court, non cyclique. Elle est faite de relations peu complexes, souvent de subordination ou d'obligation (achat/vente, conseil, contrôle, interdiction/autorisation), rythmées par les urgences, les irruptions d'événements (maladies, adaptations au marché, aléas climatiques soudains, etc.).

Les vétérinaires considèrent qu'ils donnent une information juste et suffisante, qu'il revient alors aux éleveurs de prendre leurs responsabilités en appliquant leurs préconisations : vacciner, traiter aux antibiotiques, faire les tests de manière suivie, mettre en place des mesures de protection pour les employés, vacciner en dose complète, etc.

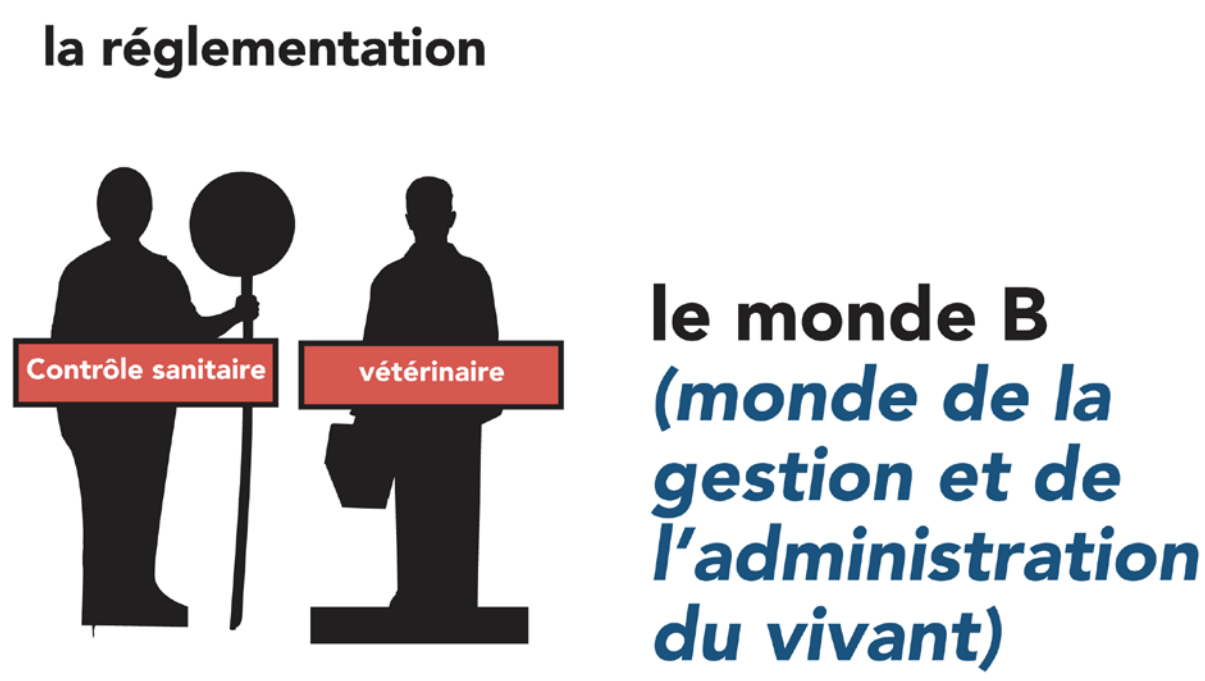

C. burnetii est insaisissable et difficile à surveiller.

Le monde de la gestion et de l'administration du vivant est régi par la temporalité de l'urgence, par l'« obligation de faire quelque chose » lors des alertes sanitaires. Il est dans l'incapacité de saisir la prolifération et la complexité microbienne.

Les éleveurs, par un discours positif sur la science, l'innovation, la propreté, le marché, la rationalité, contre l'obscurantisme, l'irrationnel, essaient d'éviter l'image d'archaïsme qu'on leur colle et de se rapprocher du monde de la gestion et de l'administration du vivant

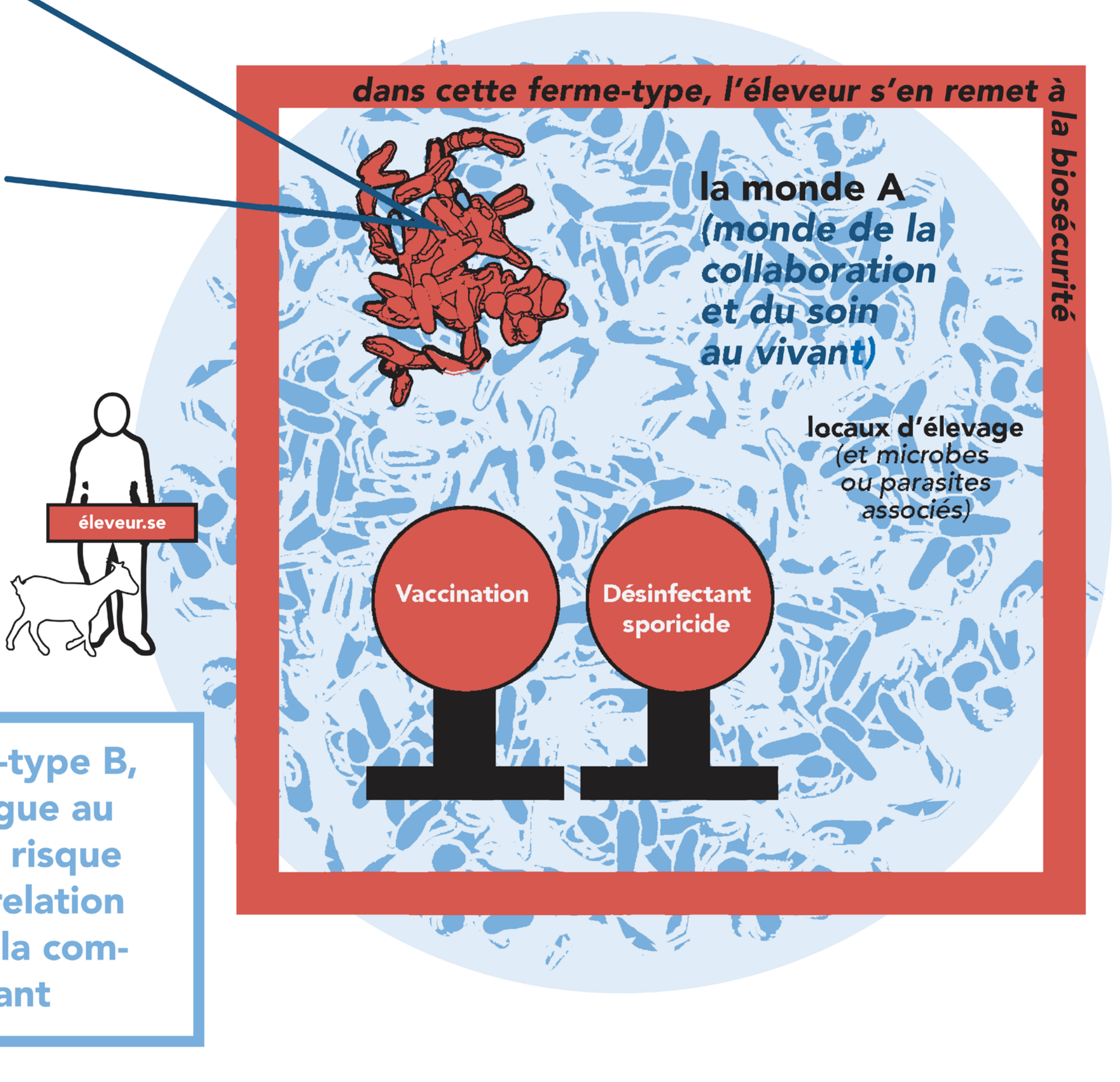

Dans la ferme-type B, l'éleveur délègue au monde B… au risque de perdre sa relation personnelle à la complexité du vivant

Retour sur l'enquête anthropologique

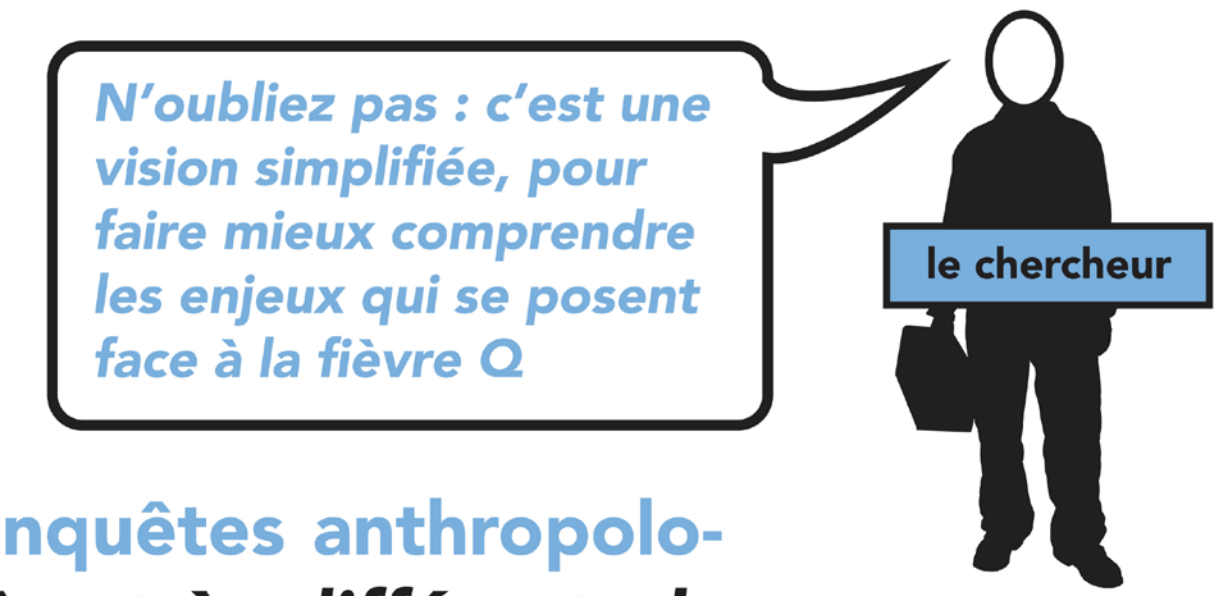

Emilie Ramillien présente les résultats des enquêtes anthropologiques: ***les éleveurs appréhendent de manière très différente la situation sanitaire de leur élevage selon le degré de délégation au monde B (monde de la gestion et de l'administration du vivant). Or, la relation aux microbes est très différente selon que le monde A ou le monde B prédomine.***

Dans la ferme-type B, les microbes sont les ennemis de la santé publique qui est dans l'incapacité de saisir la prolifération et la complexité microbienne. Elle est alors obligée de s'en remettre à ce qu'elle sait mesurer, suivre et contrôler : les microbes pathogènes.

les relations aux microbes selon un mode de gestion par l'éradication (microbes = agents pathogènes)

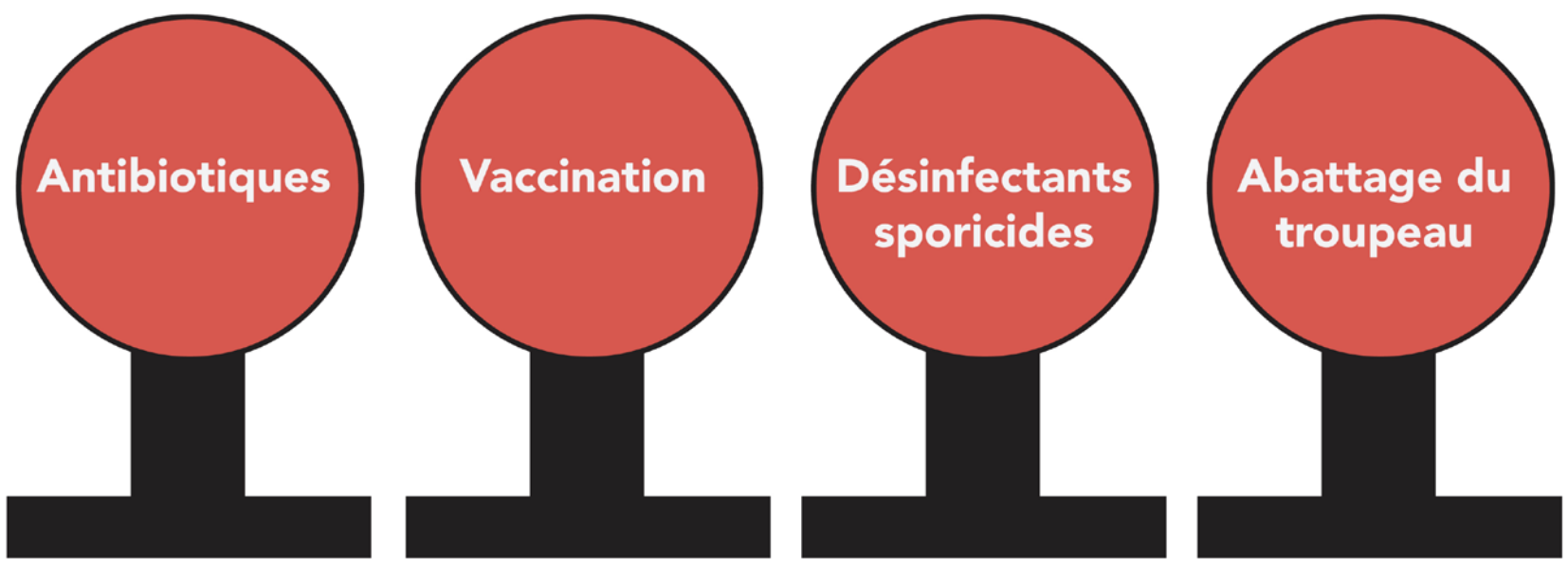

… et comment est traité *C. burnetii* dans le monde B (de la gestion et de l'administration du vivant) ?

C. burnetii dans les pratiques sanitaires d'éradication

C. burnetii est un agent biologique pathogène dont il faut se méfier compte tenu des risques sanitaires qu'elle fait courir aux animaux et aux humains. La dangerosité de C. burnetii est encore mal définie par la science, qui rapporte l'existence d'incertitudes encore grandes quant à l'écologie de la fièvre Q et aux combinaisons des facteurs favorisant sa transmission et sa pathogénicité

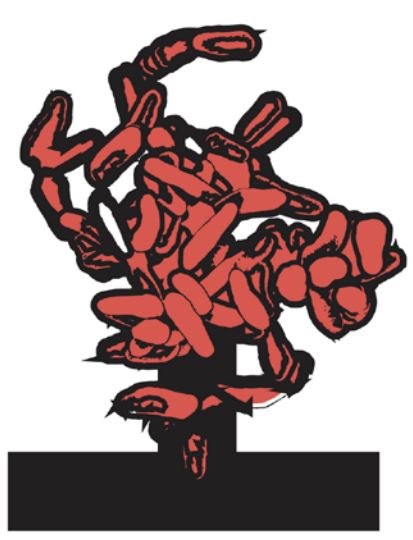

C. burnetii **isolé de son milieu**

C. burnetii dans la métagénomique

Les connaissances issues de la métagénomique heurtent les manières dont le monde de la gestion et de l'administration du vivant doit penser l'action face à une alerte sanitaire. Elles suggèrent qu'il est très difficile, voire impossible, de domestiquer et de contrôler des microbes intriqués dans des écosystèmes complexes

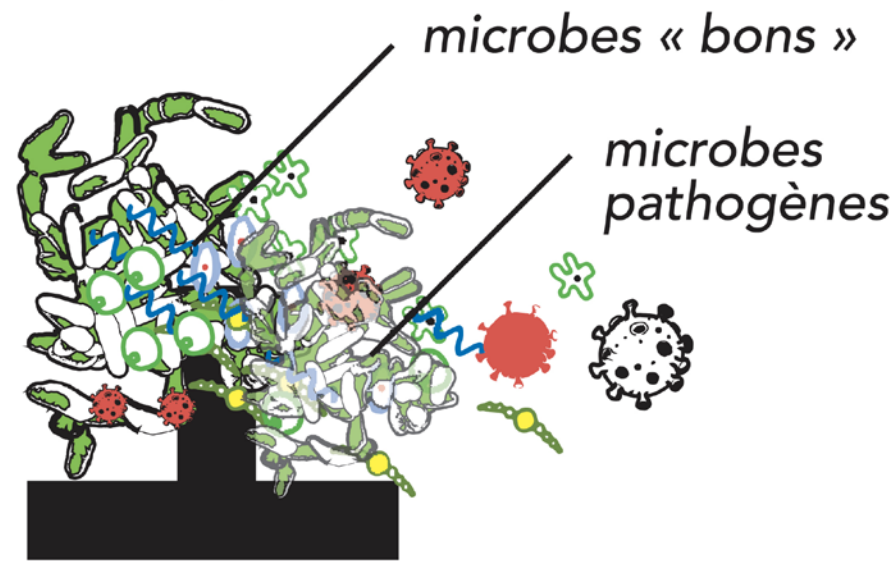

C. burnetii **perdu dans l'holobionte, le microbiote, le microbiome**

Dans la ferme-type A, l'éleveur entretient une relation diplomatique avec les microbes.

formes d'expérience des éleveurs dans la gestion des risques sanitaires

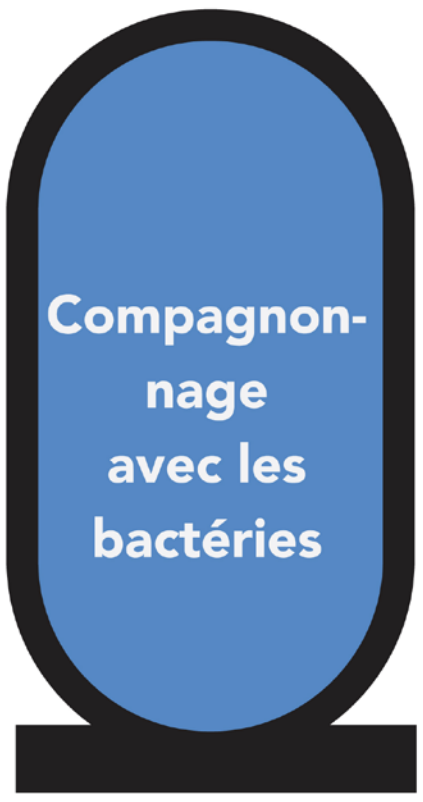

L'ambivalence des microbes préserve les éleveurs de problèmes sanitaires : « je fais très peu d'hygiène de traite et je me dis que mon système nous protège ».

« J'ai mis des chats partout aussi. Parce que les chats, cela immunise tout le monde. Et on n'a jamais eu de fièvre Q »

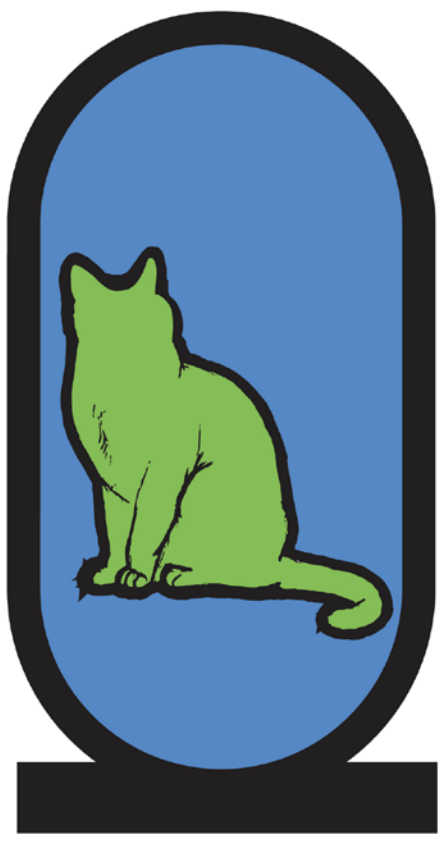

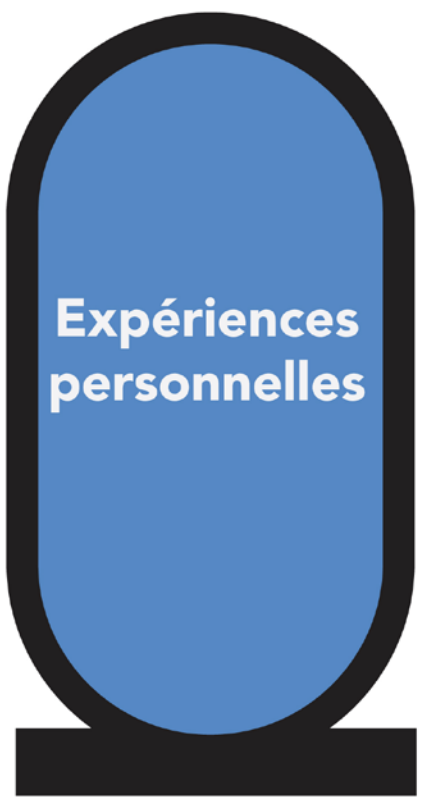

Une relation de compagnonnage, d'entraide avec les bonnes bactéries, les bonnes levures (penser à elles, faire en sorte qu'elles soient bien accueillies, en évitant les désinfections violentes, les protéger, les défendre, face à ceux qui veulent interdire le lait cru par exemple) ; favoriser leur dispersion et leur diversification avec l'aide d'autres animaux

… et comment est traité *C. burnetii* dans le monde A (de la collaboration et du soin au vivant) ?

C. burnetii **dans les pratiques de diplomatie avec les microbes**

La bactérie C. burnetii se trouve incluse dans un monde microbien plus grand où coexistent « les bons et les mauvais microbes ». Les éleveurs mettent en oeuvre une diplomatie avec les microbes fondée sur des expériences intimes. Ils pensent les microbes dans un continuum à travers lequel se relient les êtres

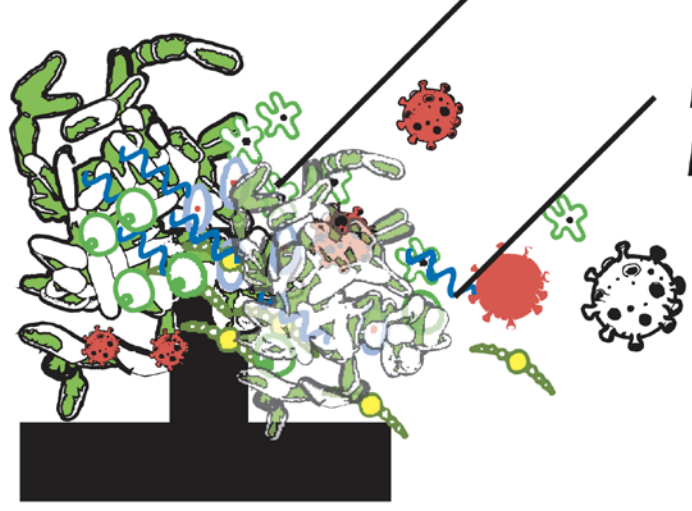

matin Zoojeu (1)

Visite du plateau de jeu + visite des objets

Les scénarios sont mis en oeuvre non pas dans l'idéal mais contraints par un contexte nouveau :
- bouleversements écosystémiques et climatiques
- émergence de nouvelles maladies infectieuses
- tension sur l'économie de l'élevage
- résistance aux antibiotiques

Composition de 4 groupes

Chaque groupe va définir une ferme-type. Le type choisi peut s'inspirer d'une ferme que l'éleveur connait bien.
- *Les fermes-type sont en difficulté économique.*
- *Les fermes-type sont en bon état sanitaire et l'éleveur prend soin de ses animaux.*

A

CONTEXTE CHOISI PAR LE GROUPE

action 1 : les éleveurs du groupe déterminent pour le type de ferme, si la ferme comporte seulement **(a)** un atelier caprin (livreur), **(b)** un atelier caprin (fromager) ou **(c)** un atelier caprin + autres ateliers (pictogramme «bovin», «poule»,…).

action 2 : les éleveurs du groupe déterminent pour le type de ferme, le nombre de chèvres (moins de 100, moins de 500, plus de 500)

action 3 : les éleveurs du groupe déterminent pour le type de ferme, si les élevages sont hors sol ou à l'herbe.

action 4 : les éleveurs du groupe déterminent pour le type de ferme, si le marché est «agro-industriel» ou «sous signe de qualité» (AOP,…)

B

MISE EN PLACE DE L'ÉVÈNEMENT

Un nombre anormalement élevé d'avortements survient sur une période d'une semaine (3 ou 10 avortements selon que la taille du troupeau est <100 ou >100, respectivement). L'éleveur a en tête que ces avortements peuvent être causés par la fièvre Q car, l'année précédente, ses animaux avaient fait l'objet de prélèvements dans un contexte de cas humains groupés

Une possibilité est que ces avortements soient causées par *Coxiella burnetii*. Excrétée essentiellement au moment de la mise-bas ou lors d'un avortement, *Coxiella burnetii* est une bactérie très résistante dans le milieu extérieur, qui peut rester détectable dans les fumiers et les poussières de bâtiments d'élevage pendant plusieurs mois voire années.

action 5 : Quels sont les objets qui, potentiels jusqu'ici, s'imposent dans la ferme ?

C

SITUATION DES RISQUES : CE QUI SUSCITE SÉCURITÉ ET VULNÉRABILITÉ

action 6 : les éleveurs, les vétérinaires et la santé publique placent **sur le plateau, tous les objets IMPORTANTS qui donnent de la sécurité ET des incertitudes ou vulnérabilités.**

D

MANIÈRES D'AGIR AVEC LES MICROBES : DEUX MONDES QUI FONT L'ÉPREUVE DES MICROBES DIFFÉREMMENT (COMMENT SONT ÉTABLIES LES HIÉRARCHIES ET LES ÉQUILIBRES)

L'enquête anthropologique a montré deux grands types de relations aux microbes, l'un basé sur la délégation aux gestionnaires publiques du vivant et l'autre sur l'expérience au vivant

Question *(la parole est aux éleveurs)* : quelle est la manière d'agir qui apporte de la sécurité à la ferme dans le cas de la fièvre Q ? Qu'est-ce qui amène de la confiance dans l'équilibre de votre système ?

Avec pour chaque groupe :
1 animateur du collectif ZOOJEU
2 rapporteurs en charge de restituer l'atelier l'après-midi

le PLATEAU de Zoojeu

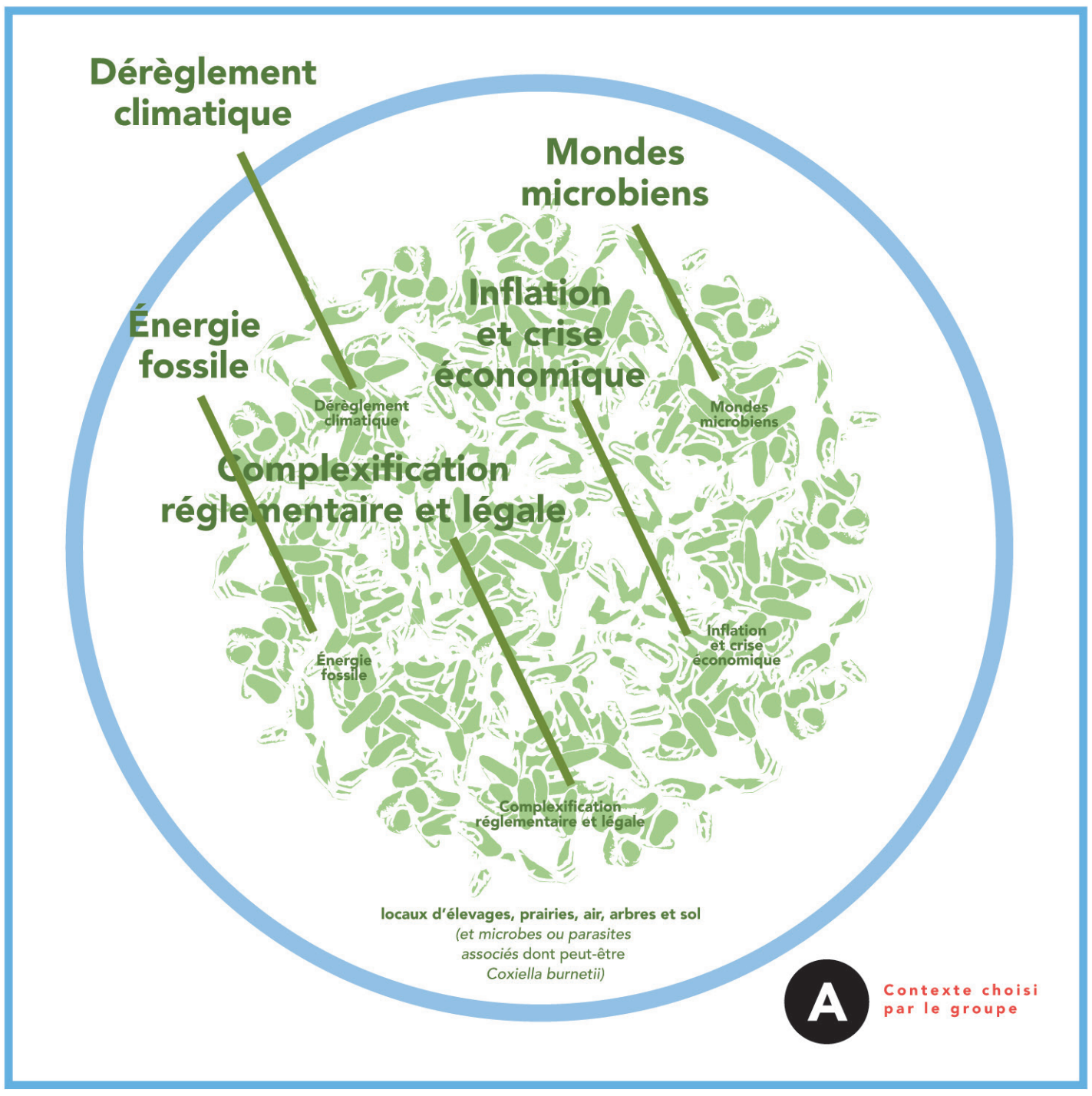

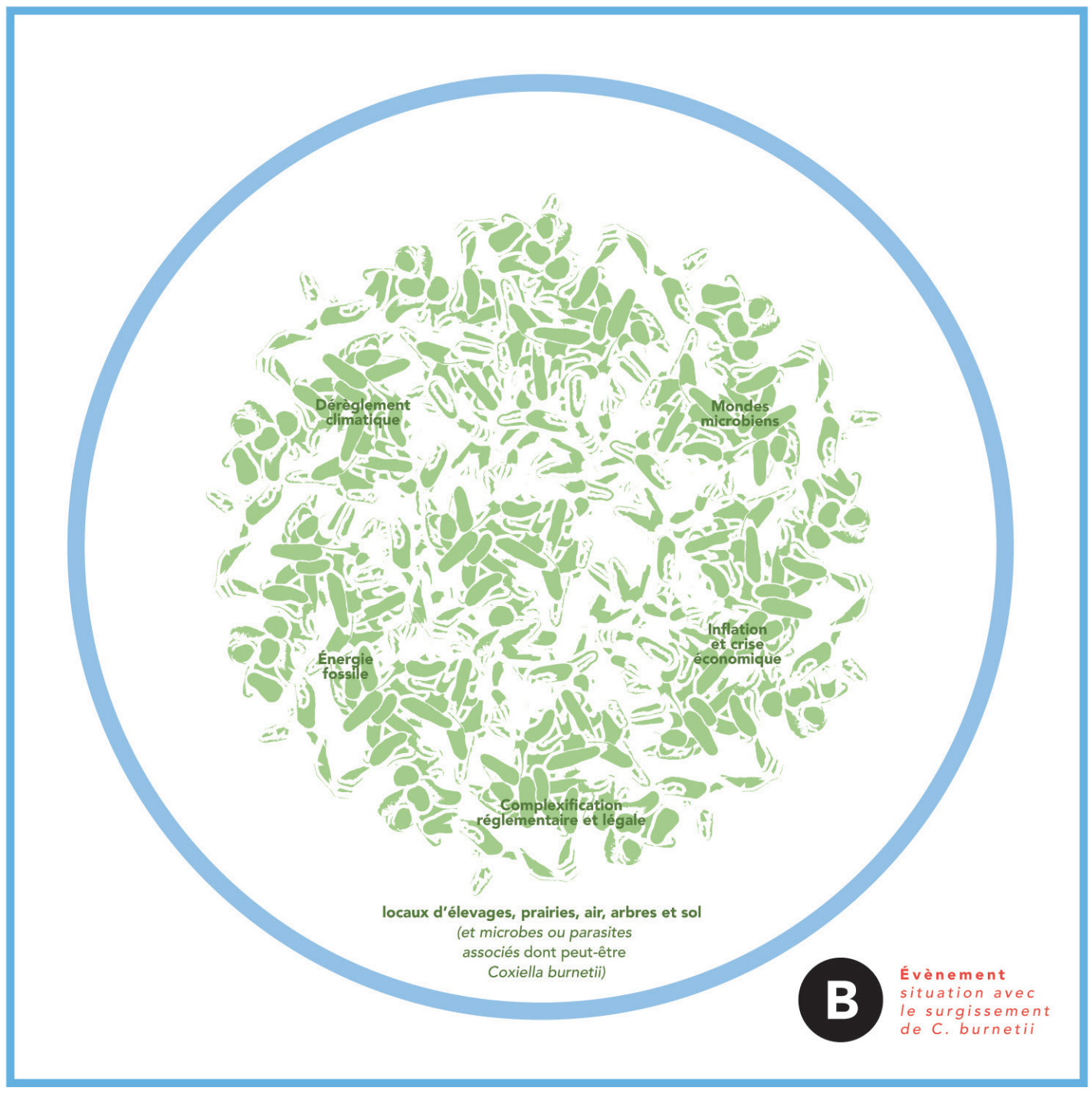

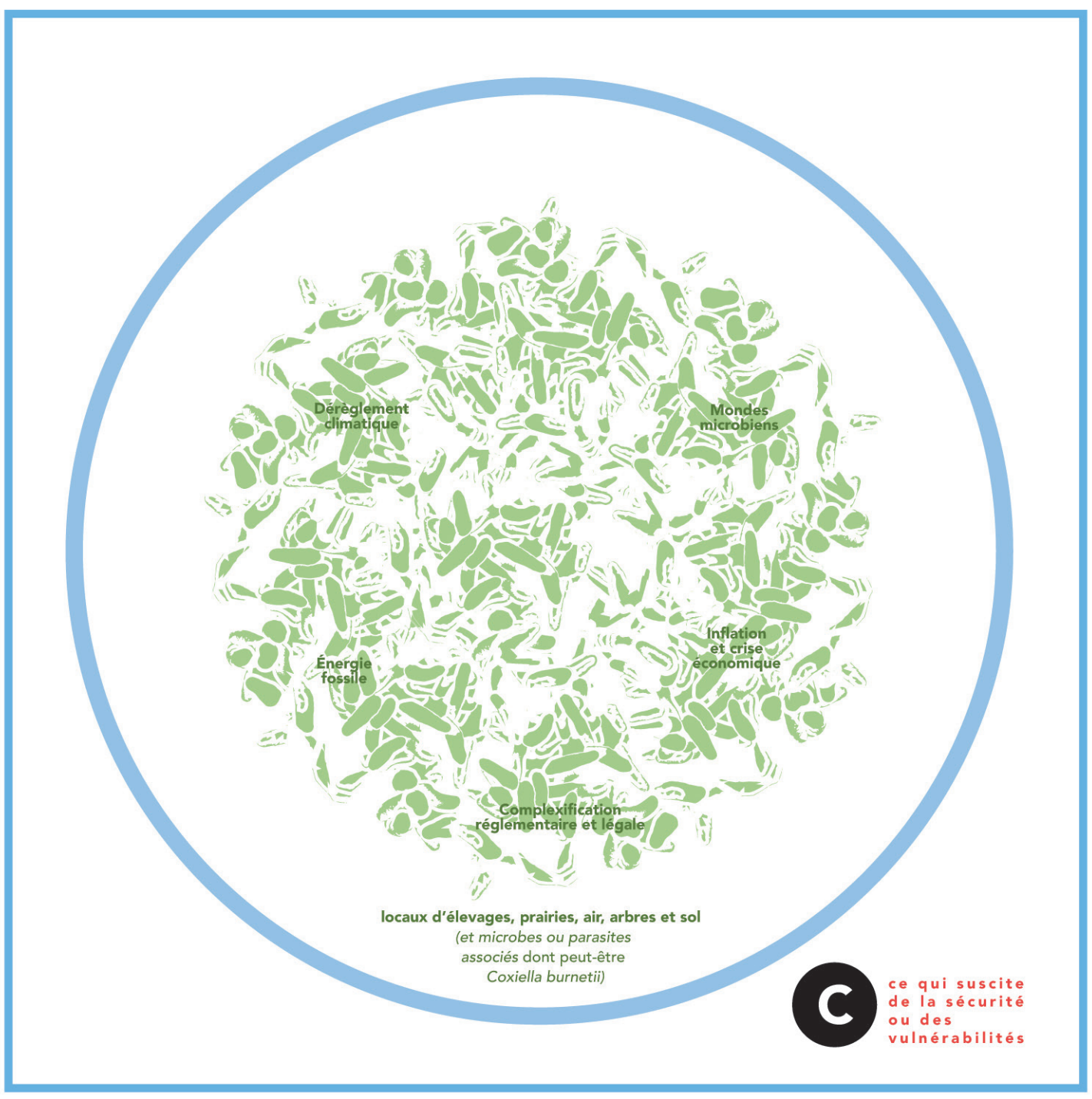

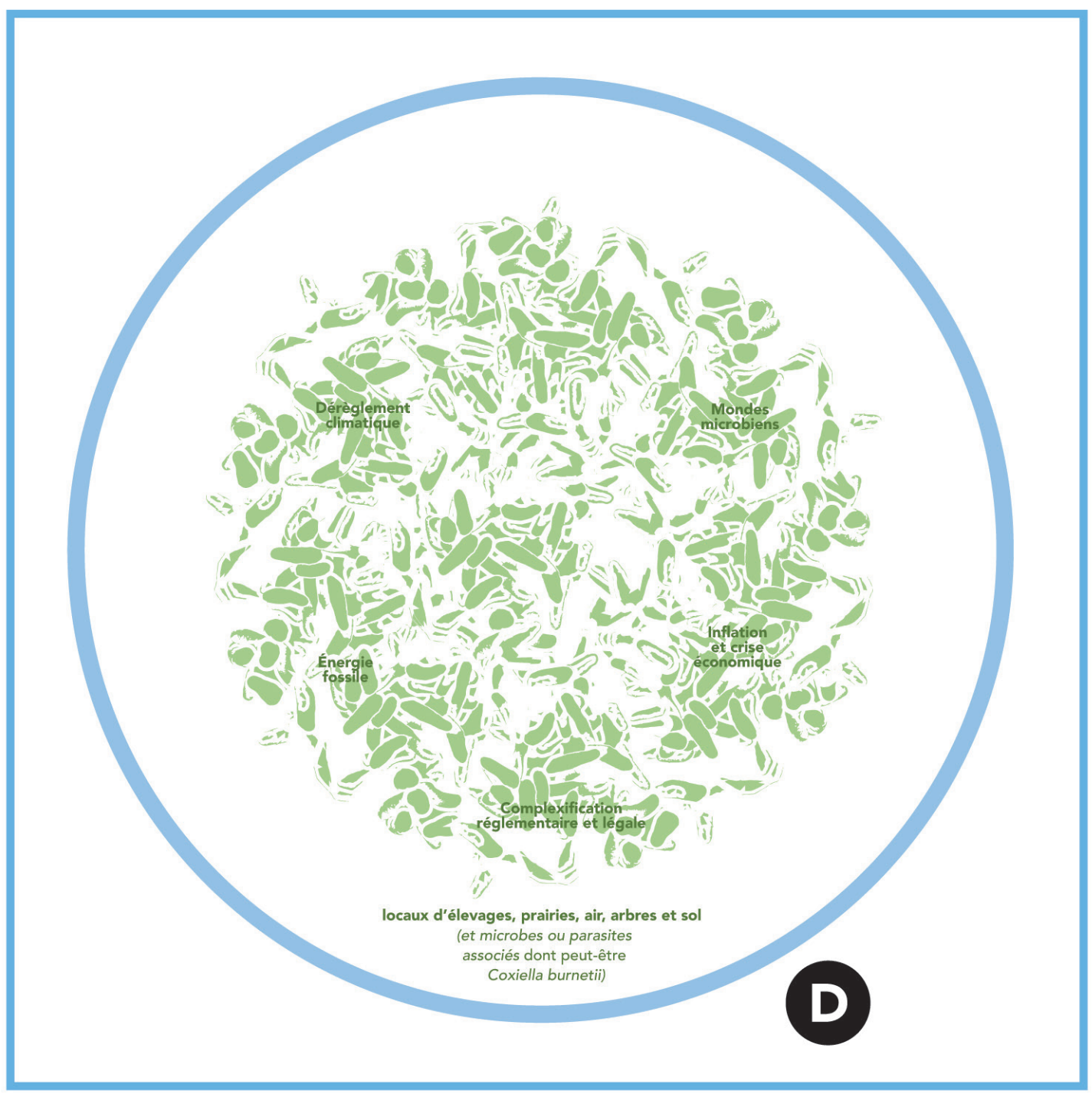

A **Contexte choisi par le groupe**

C **Situation des risques**
ce qui suscite de la sécurité ou des vulnérabilités

B **Évènement**
situation avec le surgissement de C. burnetii

D **Manières d'agir avec les microbes**

A la ferme

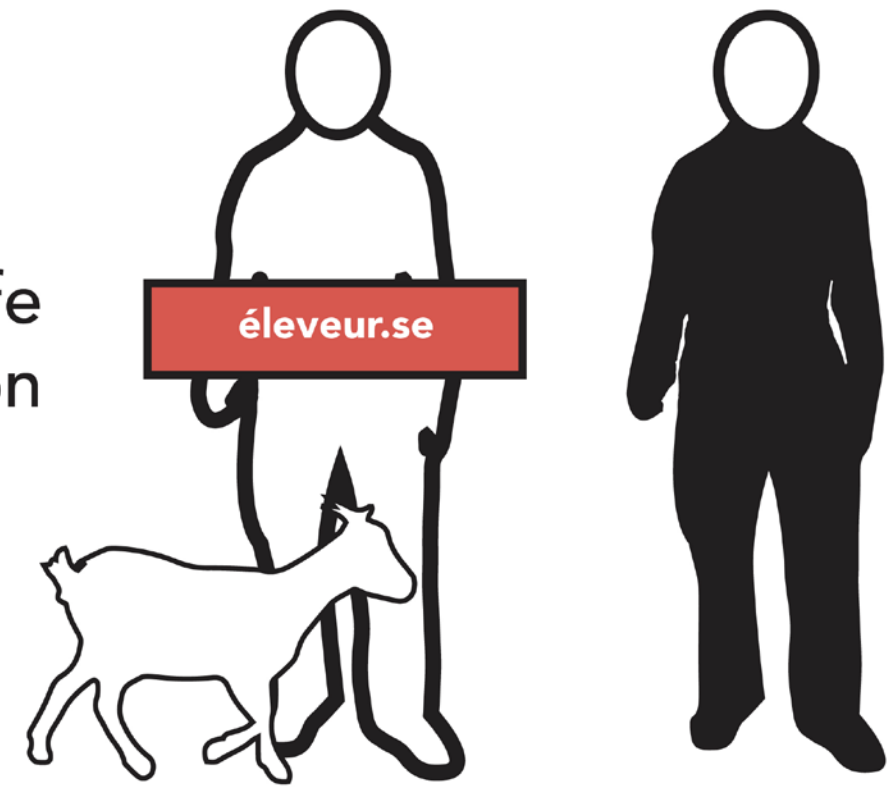

Chef.fe d'exploitation

Salarié.es

composer le tableau de bord

Ce que produit la ferme

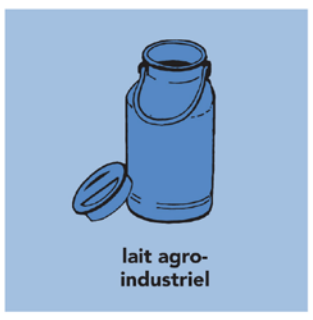

lait agro-industriel

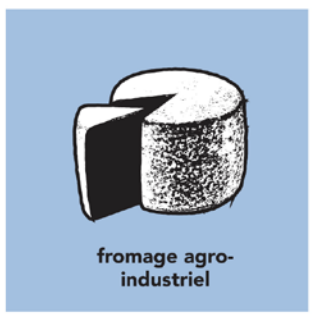

fromage agro-industriel

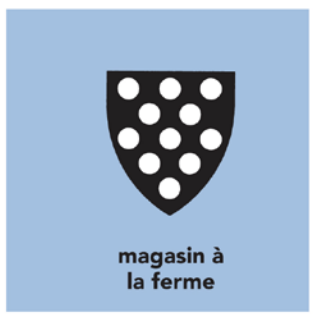

magasin à la ferme

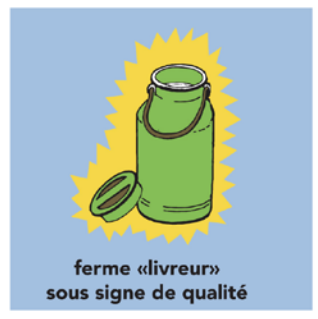

ferme «livreur» sous signe de qualité

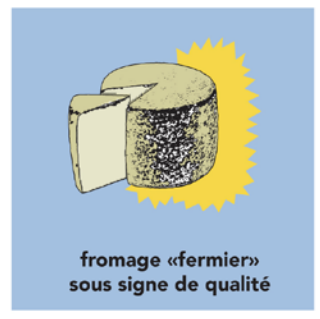

ferme produisant du fromage «fermier» sous signe de qualité

autre

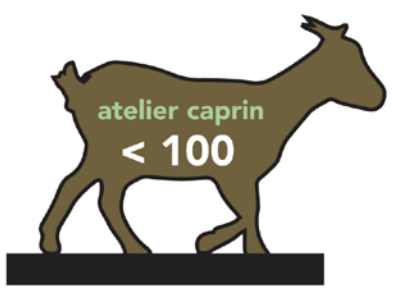

atelier caprin moins de 100

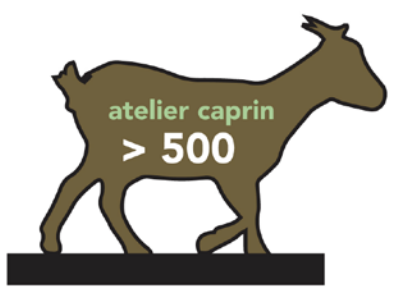

atelier caprin plus de 500

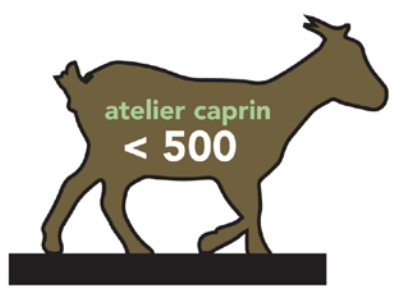

atelier caprin entre 100 et 500

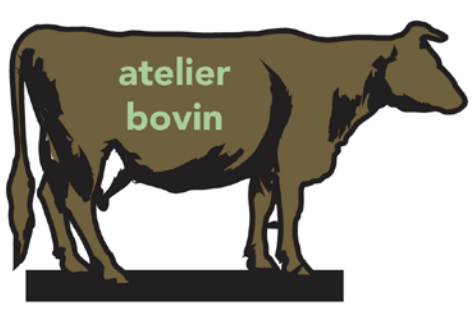

atelier bovin

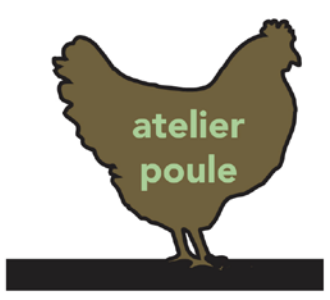

atelier poule (ou autres ateliers)

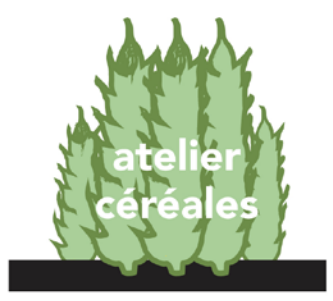

atelier céréales

Mode de production

accès à pâture

accès à aire extérieure d'exercice

HORS SOL

Type de marché

marché agro-industriel

marché sous signe de qualité

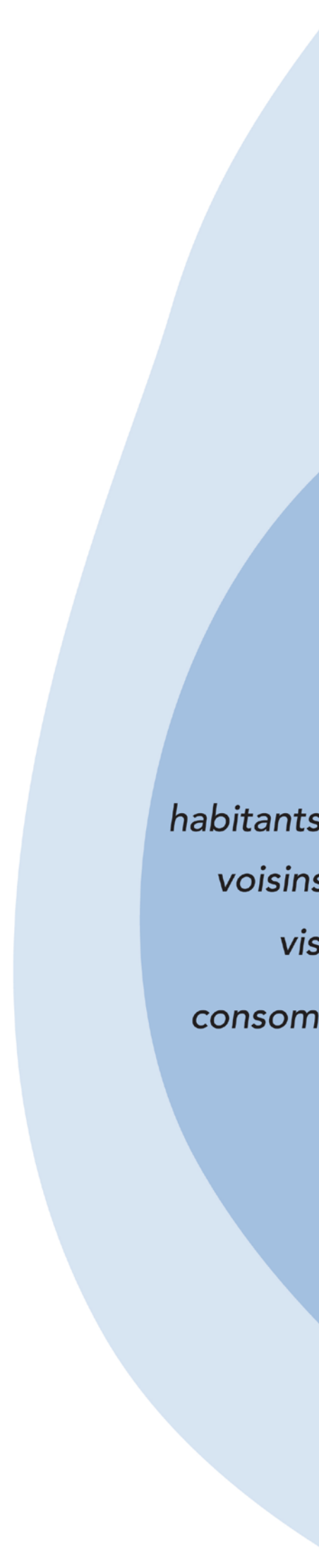

le contexte de la ferme

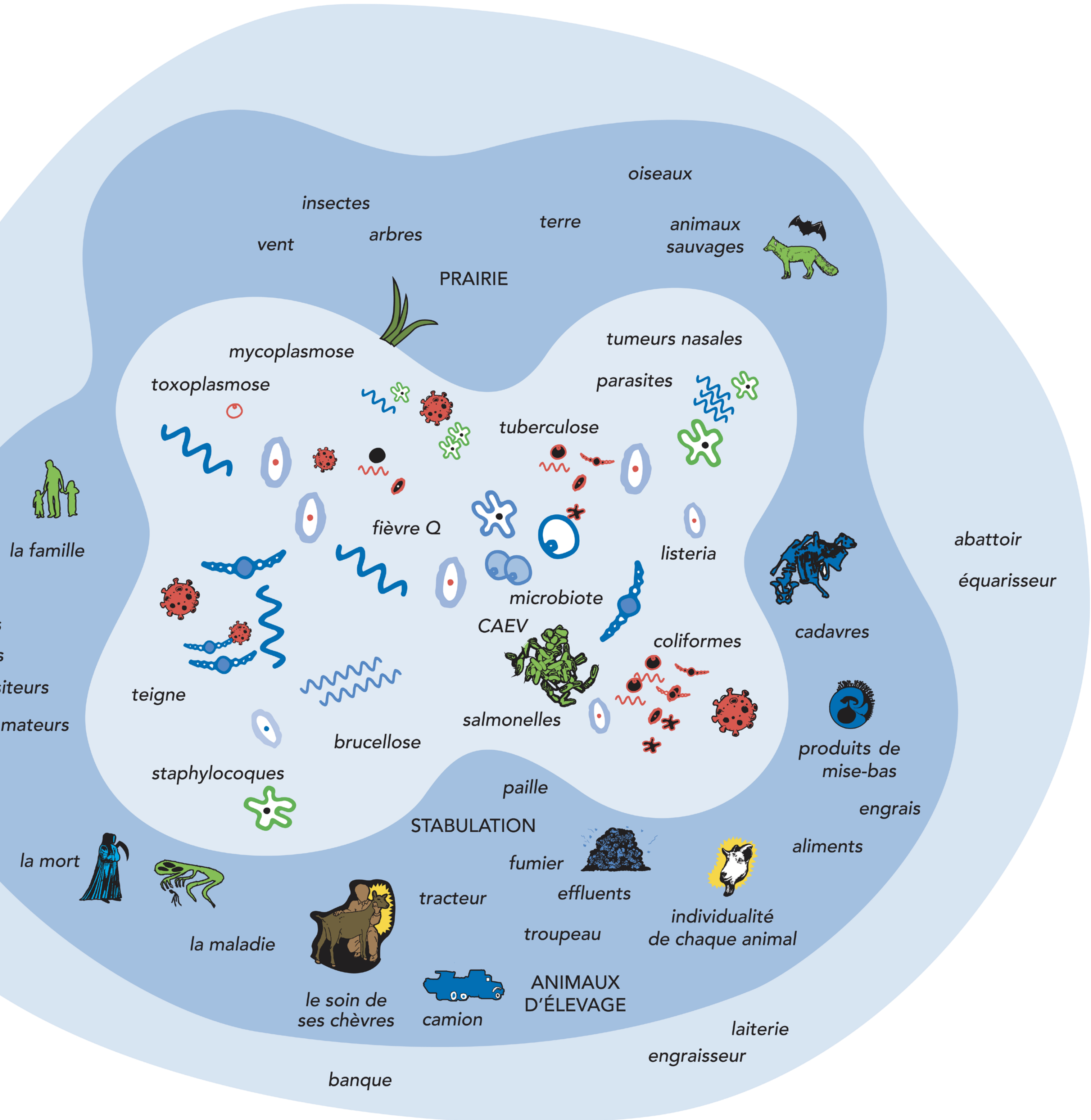

B ce qui surgit avec l'évènement

ce qui surgit avec l'évènement

mettre le carré sur le plateau et placer dans le carré tout ce qui surgit avec l'évènement

Qu'est-ce qui est affecté ou concerné ?
(sources de diffusion possibles)

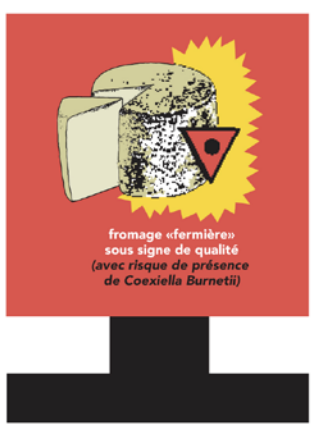

fromage «fermier» sous signe de qualité (avec risque de présence de C. burnetii)

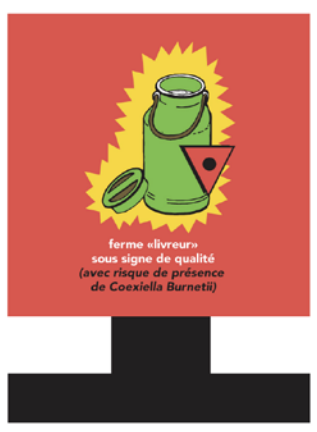

ferme «livreur» sous signe de qualité (avec risque de présence de C. burnetii)

autre

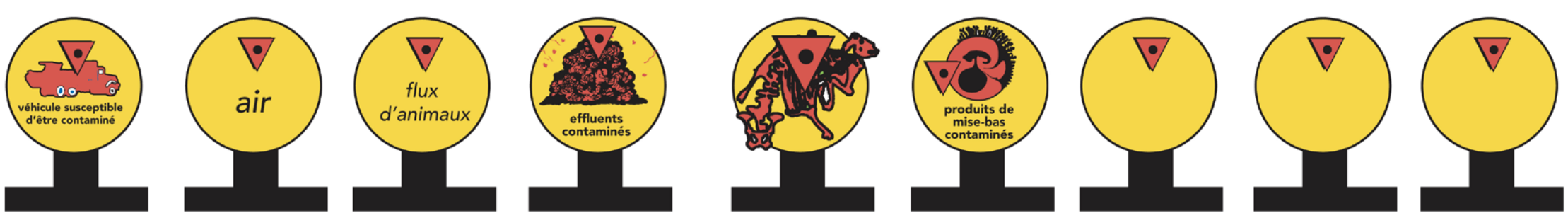

véhicule — *air* — *flux d'animaux contaminés* — *effluents contaminés* — *cadavres contaminés* — *produits de mise bas contaminés* — *autres* — *autres* — *autres*

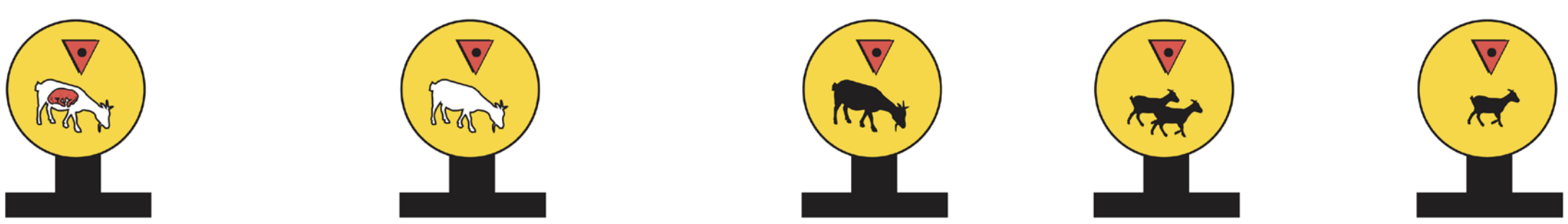

Chèvres gestantes — Chèvres en lactation — Chèvres taries — Chevrettes de renouvellement — Chevreaux

Qui fait irruption sur la ferme ?

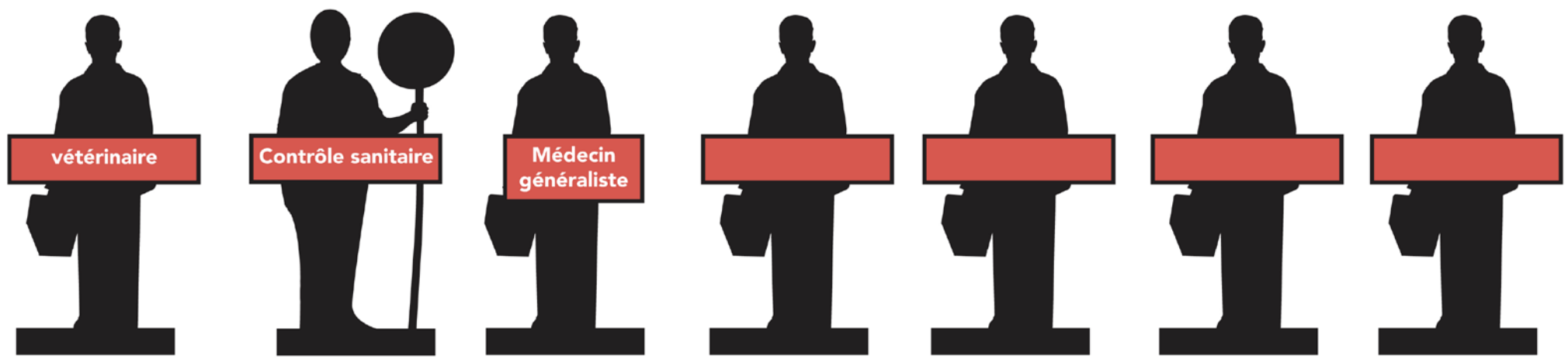

vétérinaire | contrôle sanitaire | médecin généraliste | autre | autre | autre | autre

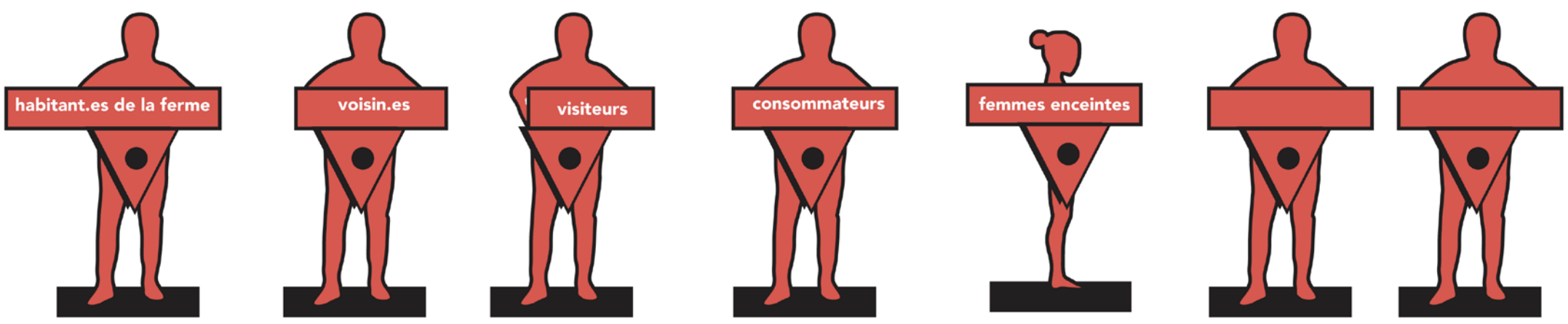

habitant.es | voisin.es | visiteurs/ visiteuses | consommateurs/ consommatrices | femmes enceintes | autre | autre

Que disent la loi et la réglementation ?

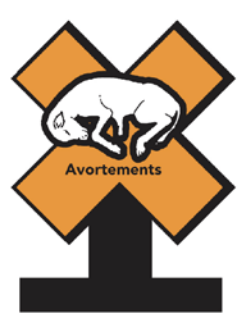

Déclaration obligatoire au vétérinaire sanitaire (surveillance de la brucellose)

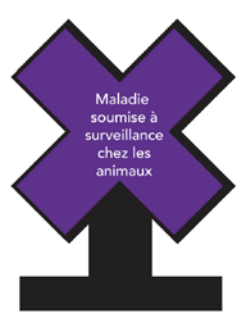

Maladie soumise à surveillance (catégorie E) chez les animaux

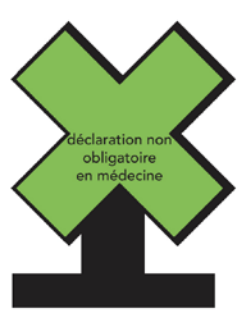

Maladie dont la déclaration n'est pas obligatoire chez les humains

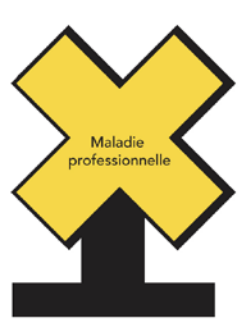

Maladie professionnelle indemnisable chez les humains

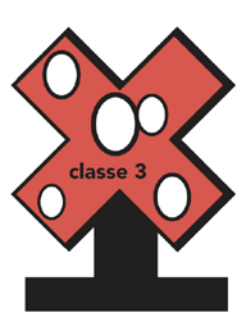

Agent pathogène de classe 3 (R. 4421-3 du Code du Travail)

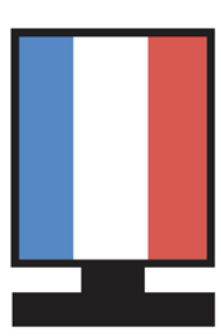

Note de service n°2011-8124 si survenue de cas humains groupés

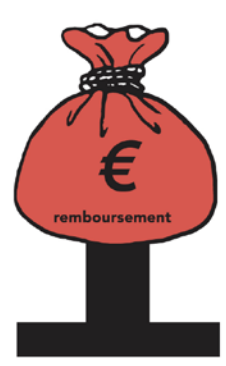

financement public d'une gestion du risque

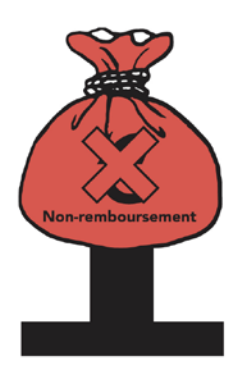

absence de financement public d'une gestion du risque

C ce qui suscite de la sécurité et des vul

MODE DE GESTION DES MICROBES PAR LA COHABITATION

Le travail thérapeutique de l'éleveur (ou du vétérinaire)

mise en oeuvre
de technique créée
par l'éleveur (ou
le vétérinaire)

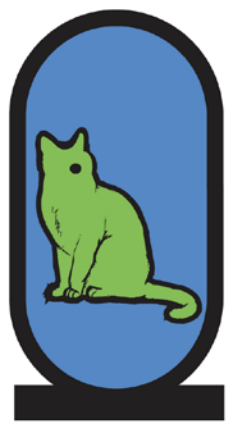

« J'ai mis des chats partout aussi. Parce que les chats, cela immunise tout le monde. Et on n'a jamais eu de fièvre Q »

diagnostics médicaux
sur les animaux
réalisés par
l'éleveur (ou le vétérinaire)

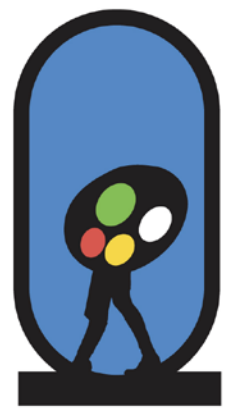

« l'outil majeur c'est internet pour identifier les symptômes »

fourniture de
traitements ou
médicaments par l'éleveur
(ou le vétérinaire)

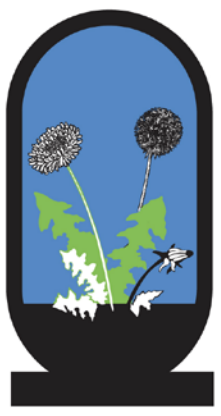

détention par l'éleveur
(ou le vétérinaire)
d'un savoir spécialisé
relatif à la santé

L'ambivalence des microbes préserve les éleveurs de problèmes sanitaires : « je fais très peu d'hygiène de traite et je me dis que mon système nous protège ».

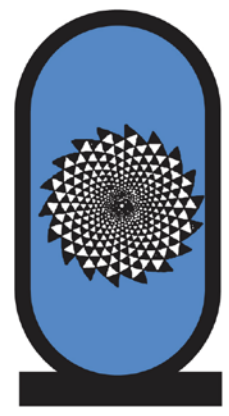

les méthodes et dispositifs

Obsalim®

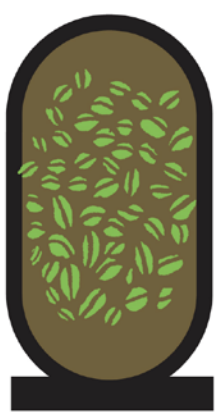

phyto-
thérapie

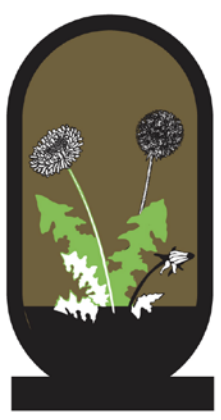

aroma-
thérapie

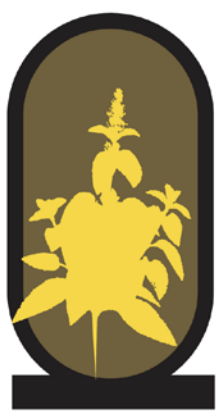

autre

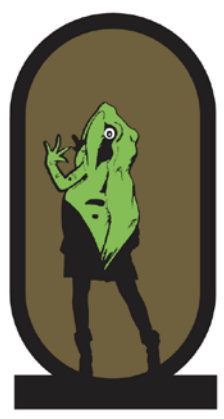

échanges entre éleveurs
(ou vétérinaires) à
propos des techniques
thérapeutiques et de soin

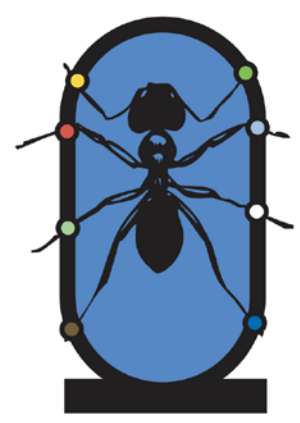

SUIVI MÉDICAL DE L'ÉLEVEUR ET DE SA FAMILLE

Connaissance de la fièvre Q ou non par le médecin

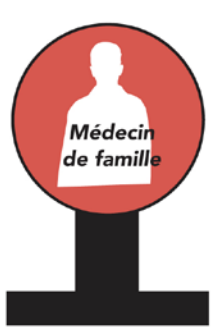

Médecin
de famille

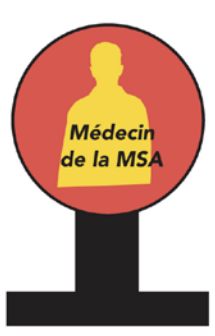

Médecin
de la MSA

Existence de facteurs de risques de gravité

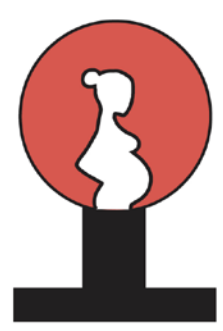

Femme
enceinte

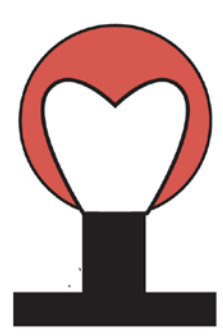

Lésion ou
prothèses
cardiaques ou
vasculaires

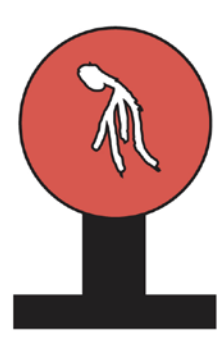

Personnes
immunodéprimées

nérabilités

DISPOSITIFS ET OUTILS DE POLITIQUE SANITAIRE

Mode de gestion des microbes par l'éradication ou la lutte ciblée contre certains agents pathogènes

BÂTIMENT

- Désinfectant sporicide
- Pratiques et protocoles sanitaires
- Fermeture au public
- Vide sanitaire
- Signalétique

PRODUITS BIOLOGIQUES

- Port de gants
- Pasteurisation du lait
- Equarrissage
- Port du masque
- Ramassage et destruction des produits de mise bas
- Container étanche pour le stockage des cadavres et produits d'avortement

ANIMAUX

- Abattage du troupeau
- Analyses de laboratoire
- Vaccination
- Antibiotiques
- Déclaration d'avortement
- Séparation des animaux

GESTION DU FUMIER

- Compostage du fumier
- Bâchage du fumier
- Utilisation d'un filet brise vent pour limiter l'aérosolisation
- Epandage avec enfouissement
- Epandage par temps calme, non pluvieux, à une période habituellement humide

- Autre
- Autre
- Autre
- Autre
- Autre
- Autre

D manières d'agir avec les microbes

DÉFAILLANCES DE POLITIQUE SANITAIRE

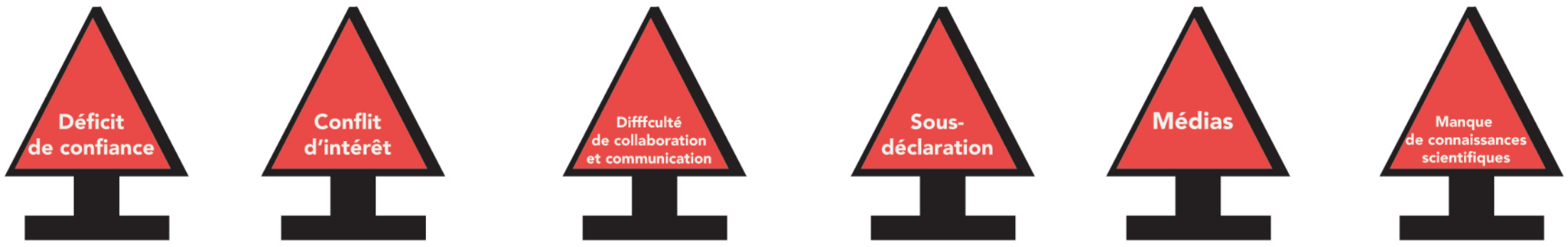

Déficit de confiance — Conflit d'intérêt — Difficulté de collaboration et de communication — Sous-déclaration — Médias — Manque de connaissances scientifiques

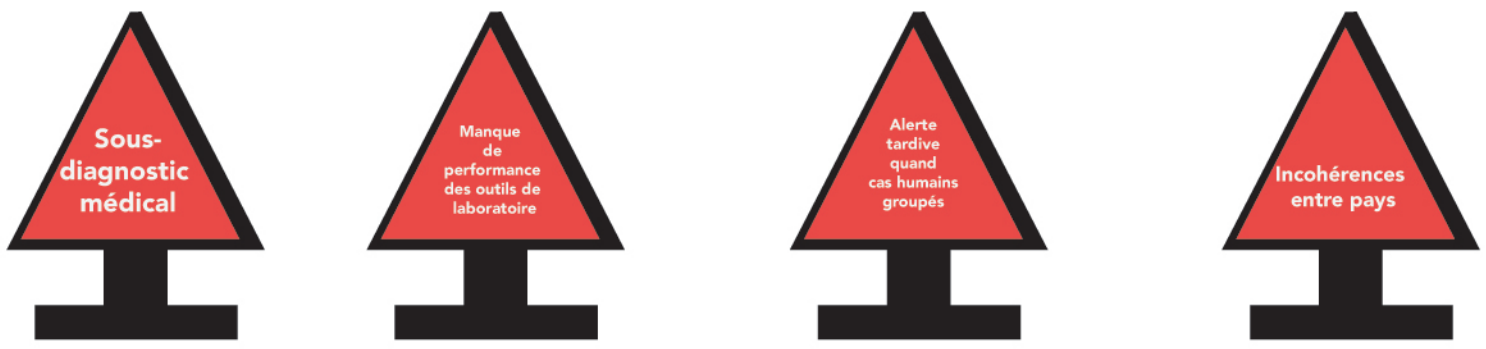

Sous-diagnostic médical — Manque de performance des outils de laboratoire — Alerte tardive quand cas humains groupés — Incohérences entre pays

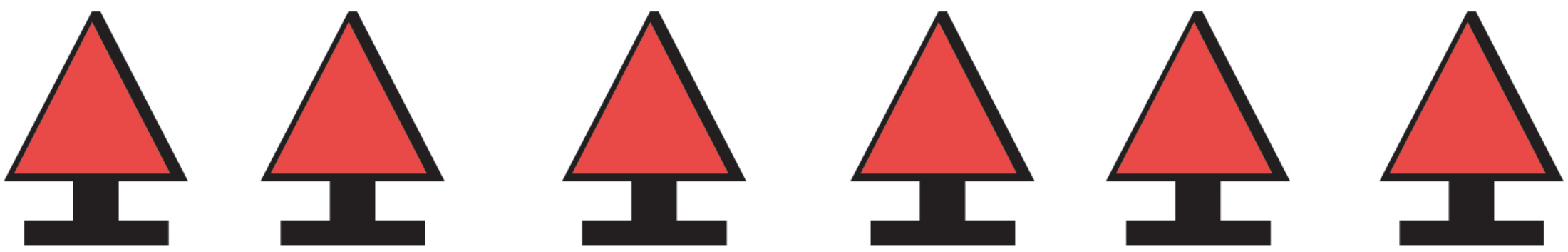

à définir — à définir — à définir — à définir — à définir — à définir

REMANIEMENT DE POLITIQUE SANITAIRE

gouverner l'action publique sanitaire (hypothèse)

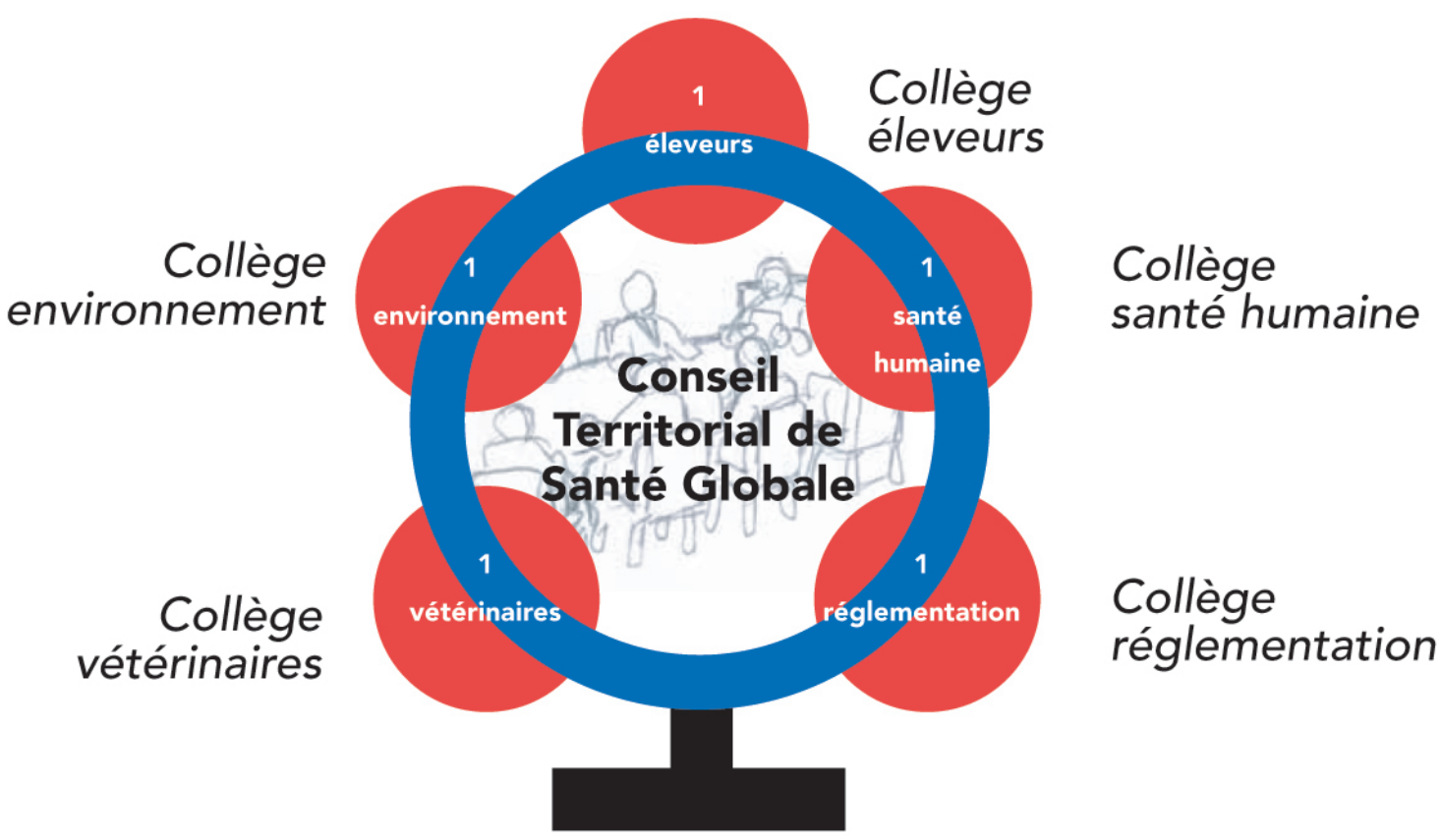

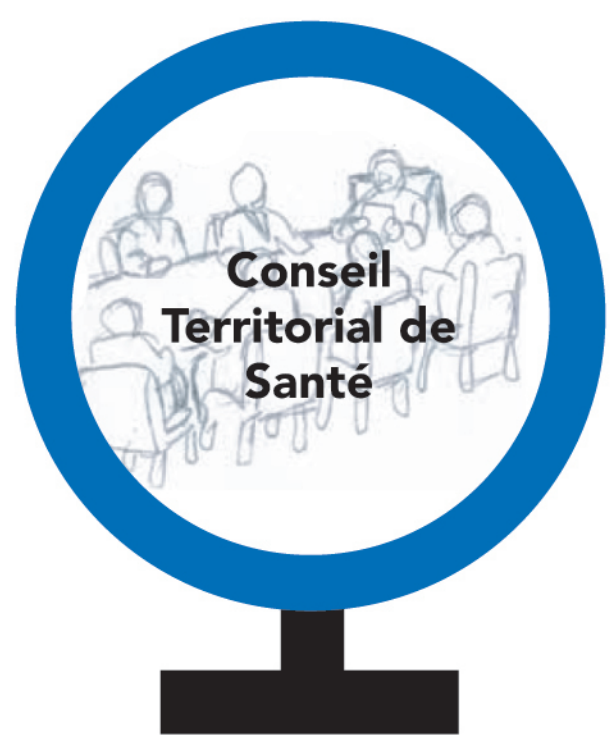

Patient expert

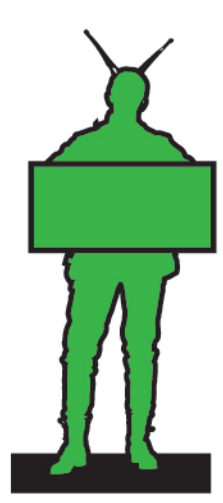

Autres

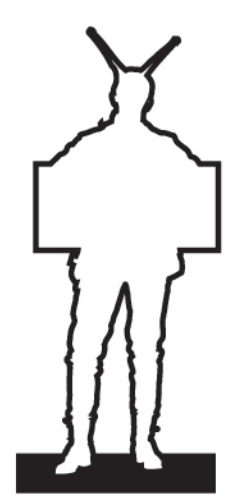

exemple de mise en place en quatre étapes

Tableau de bord

Ce que produit la ferme

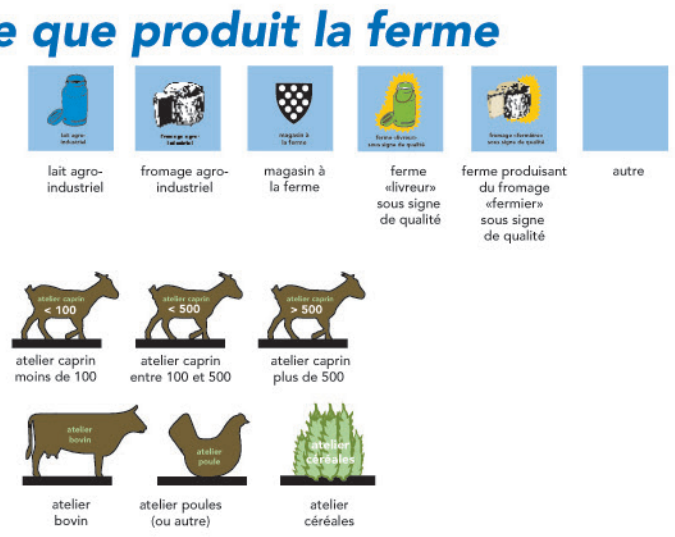

Mode de production

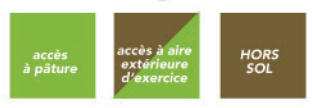

Type de marché

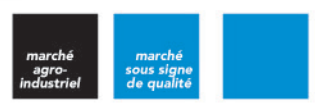

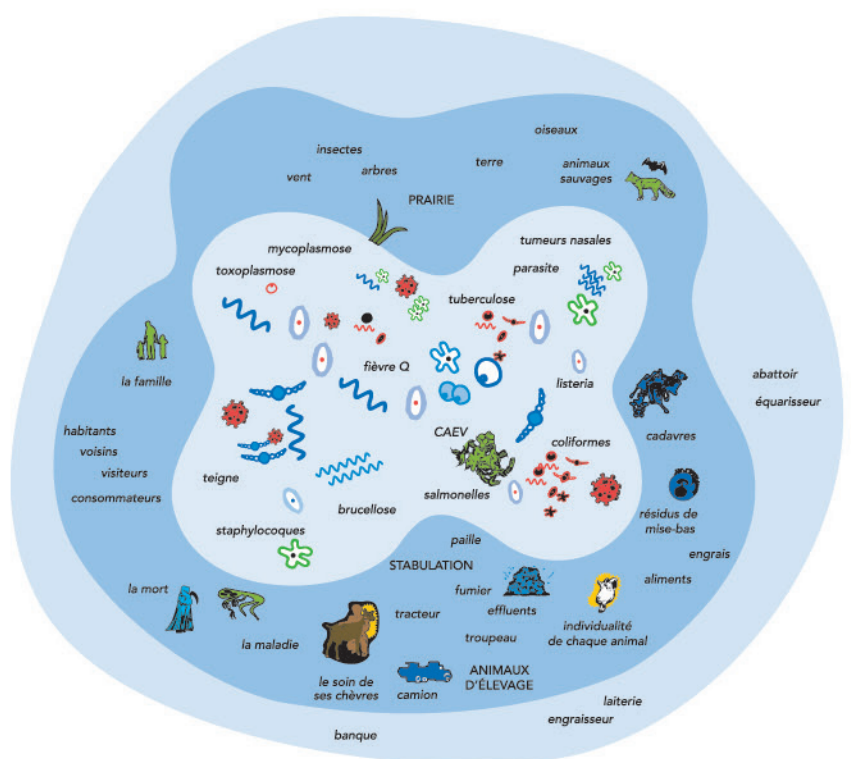

Contexte

A la ferme

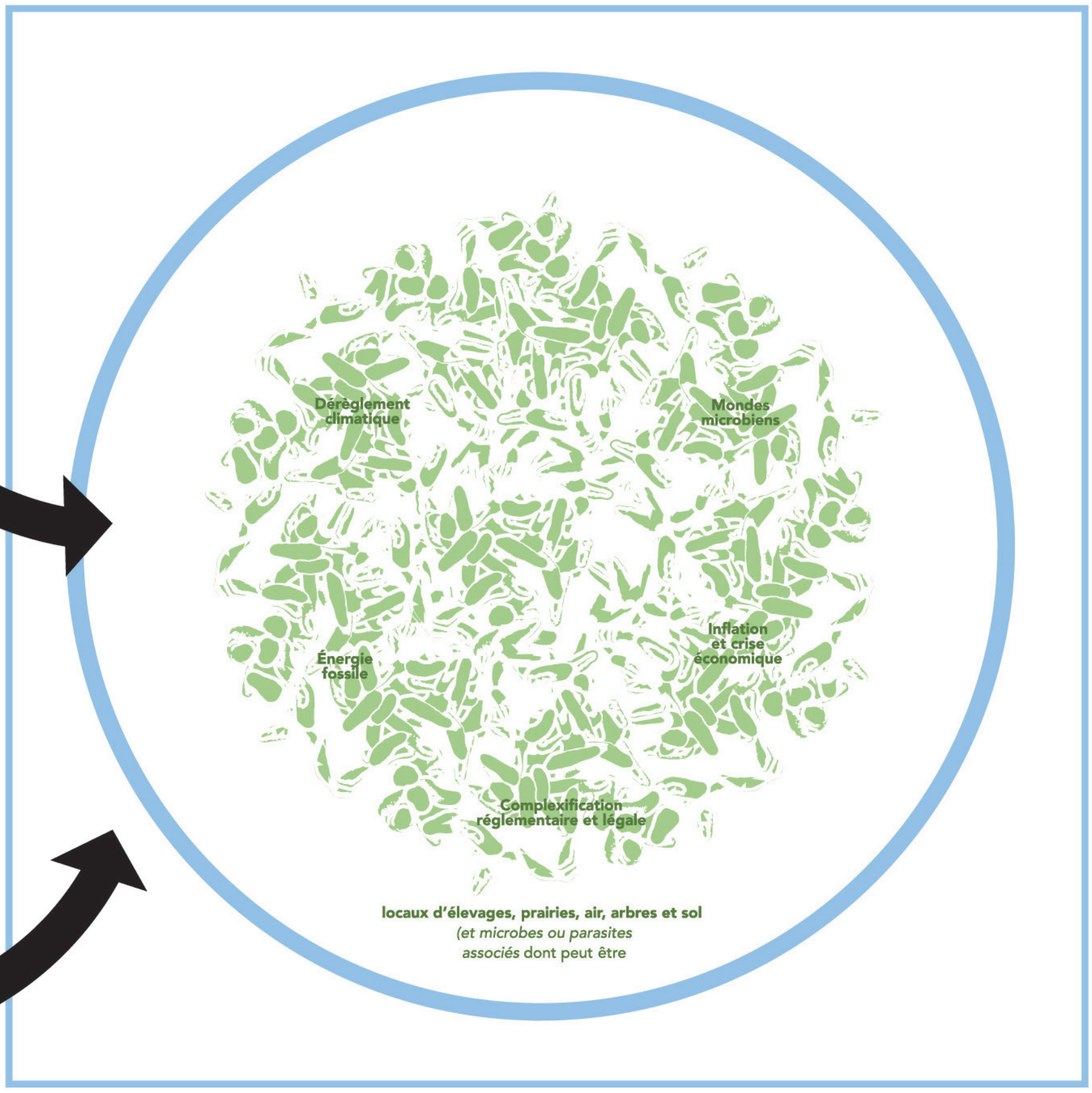

B irruption de Coxiella

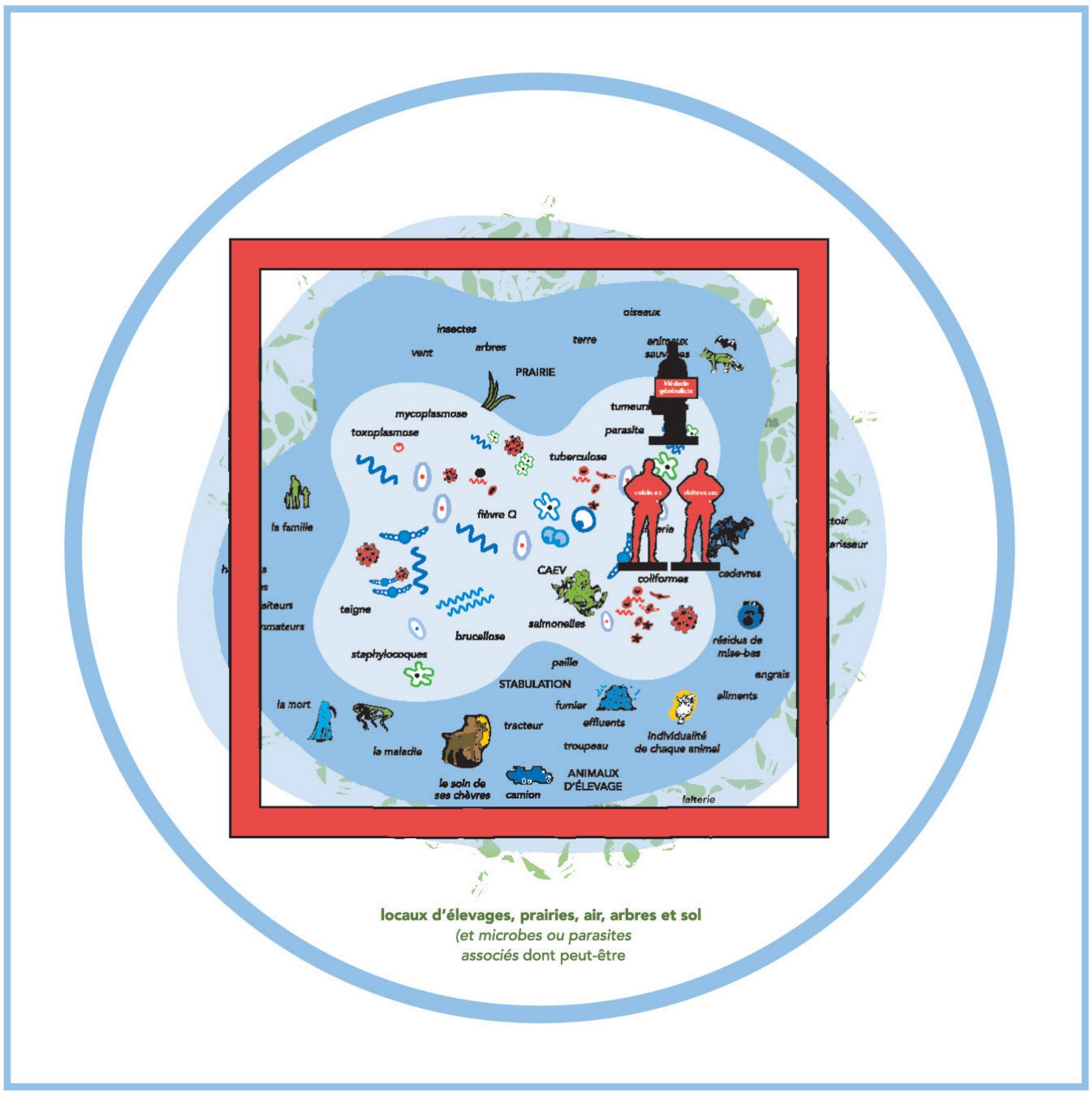

C

situation des risques
ce qui suscite
de la sécurité
ou des vulnérabilités

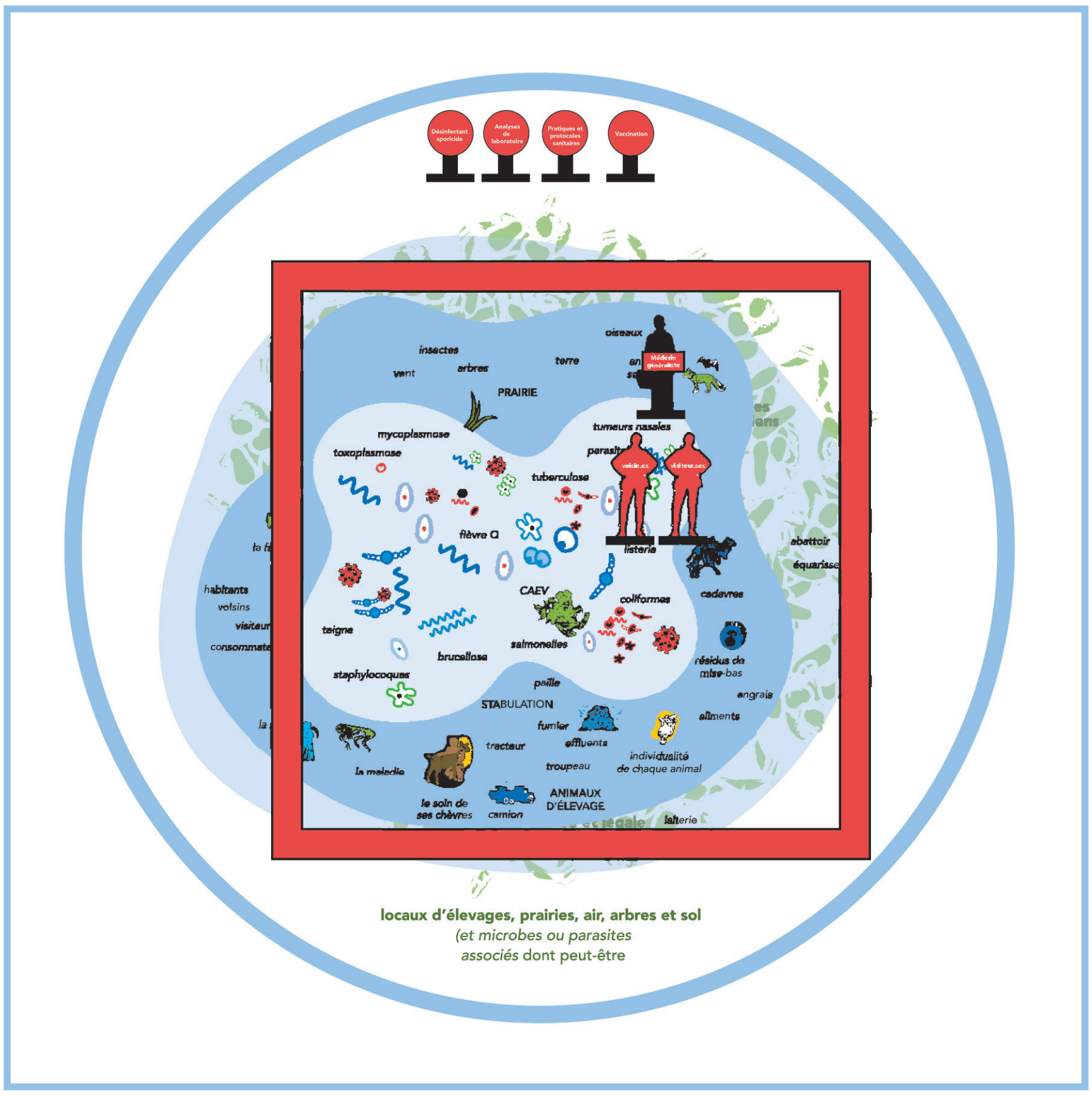

D

**manières d'agir
avec les microbes**

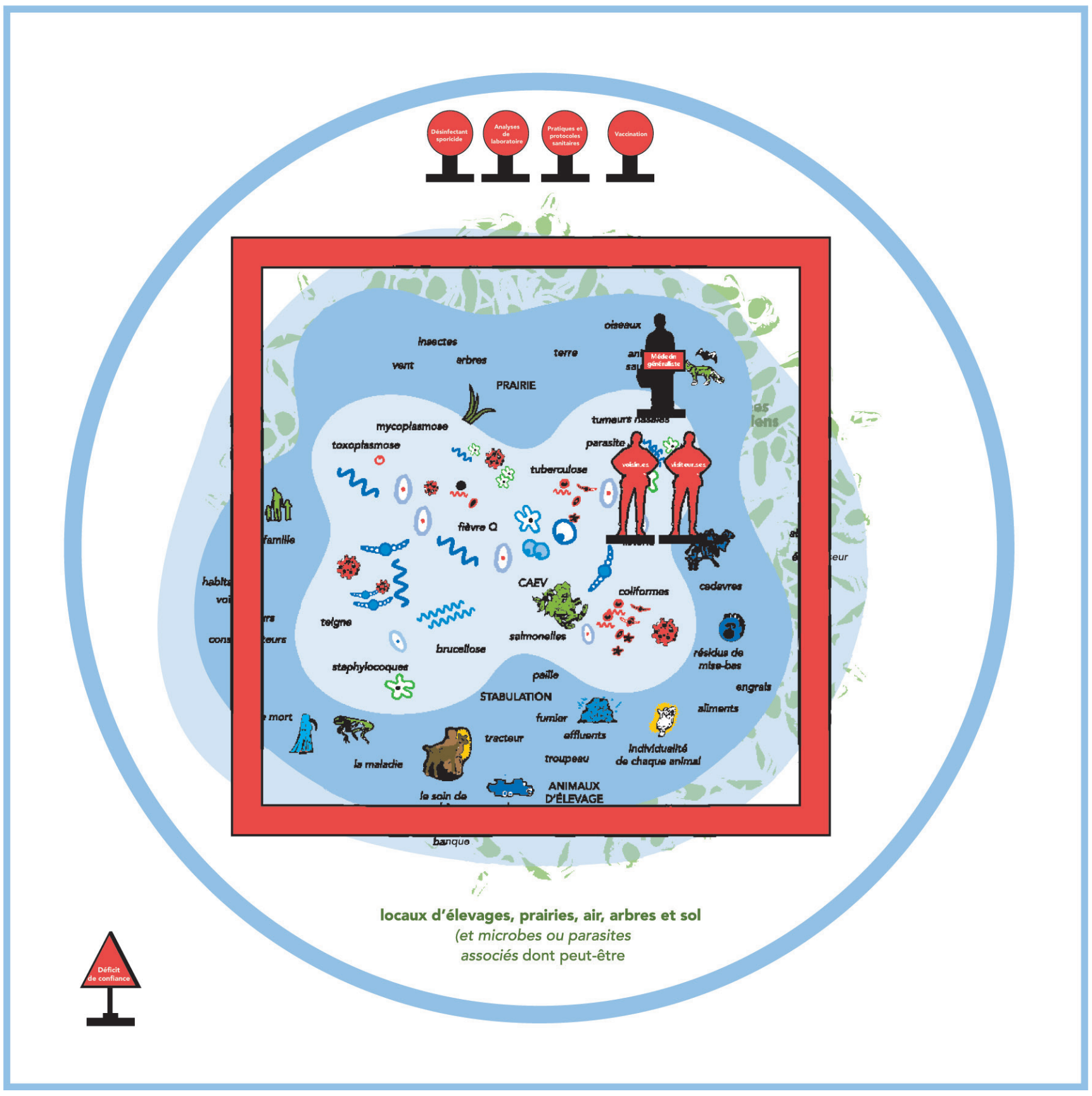

après-midi Zoojeu (2)

restitution du matin

Tous experts pour prévenir et gérer l'alerte

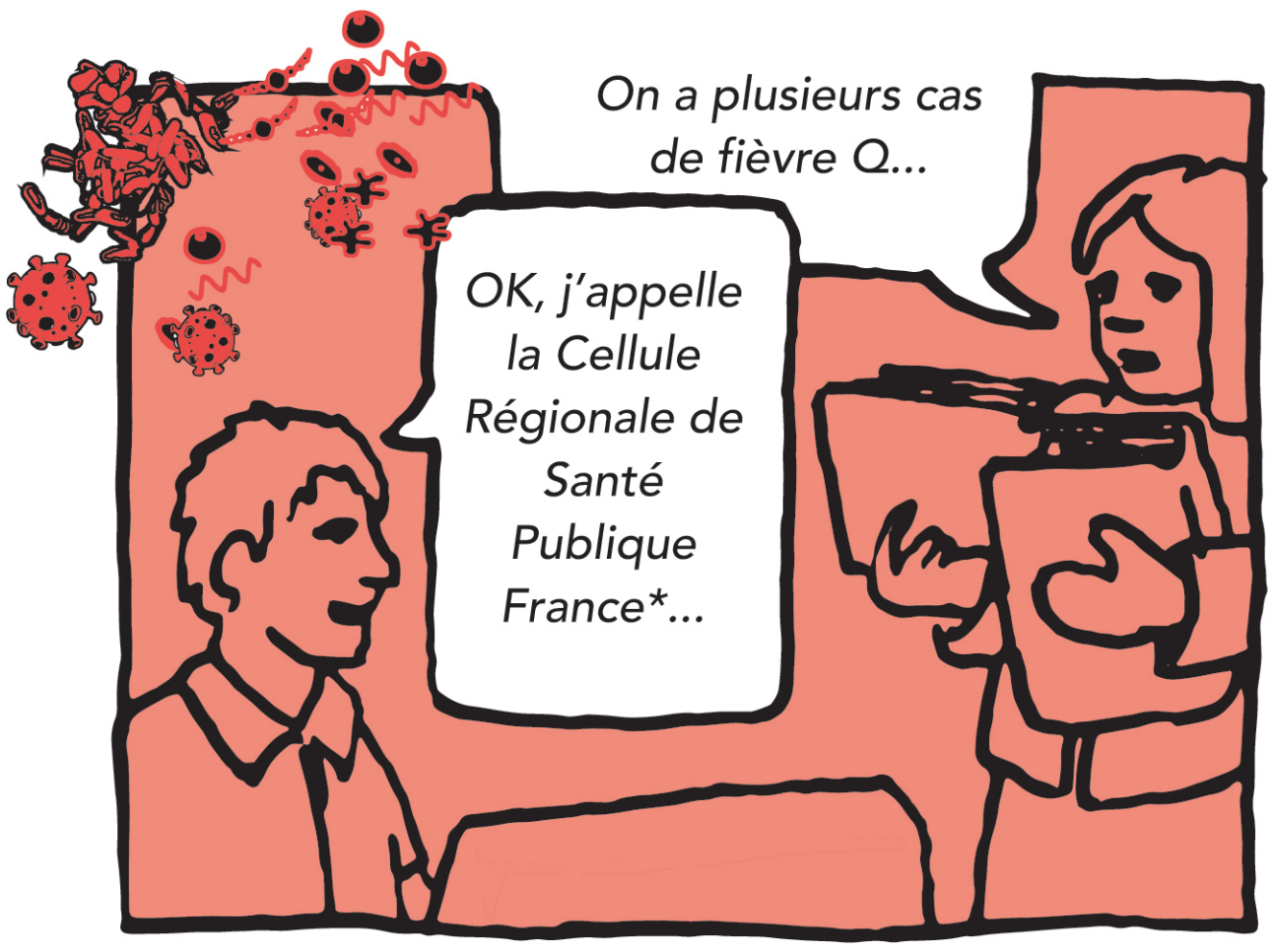

Le mardi 6 juin, l'infectiologue du Centre hospitalier fait un signalement

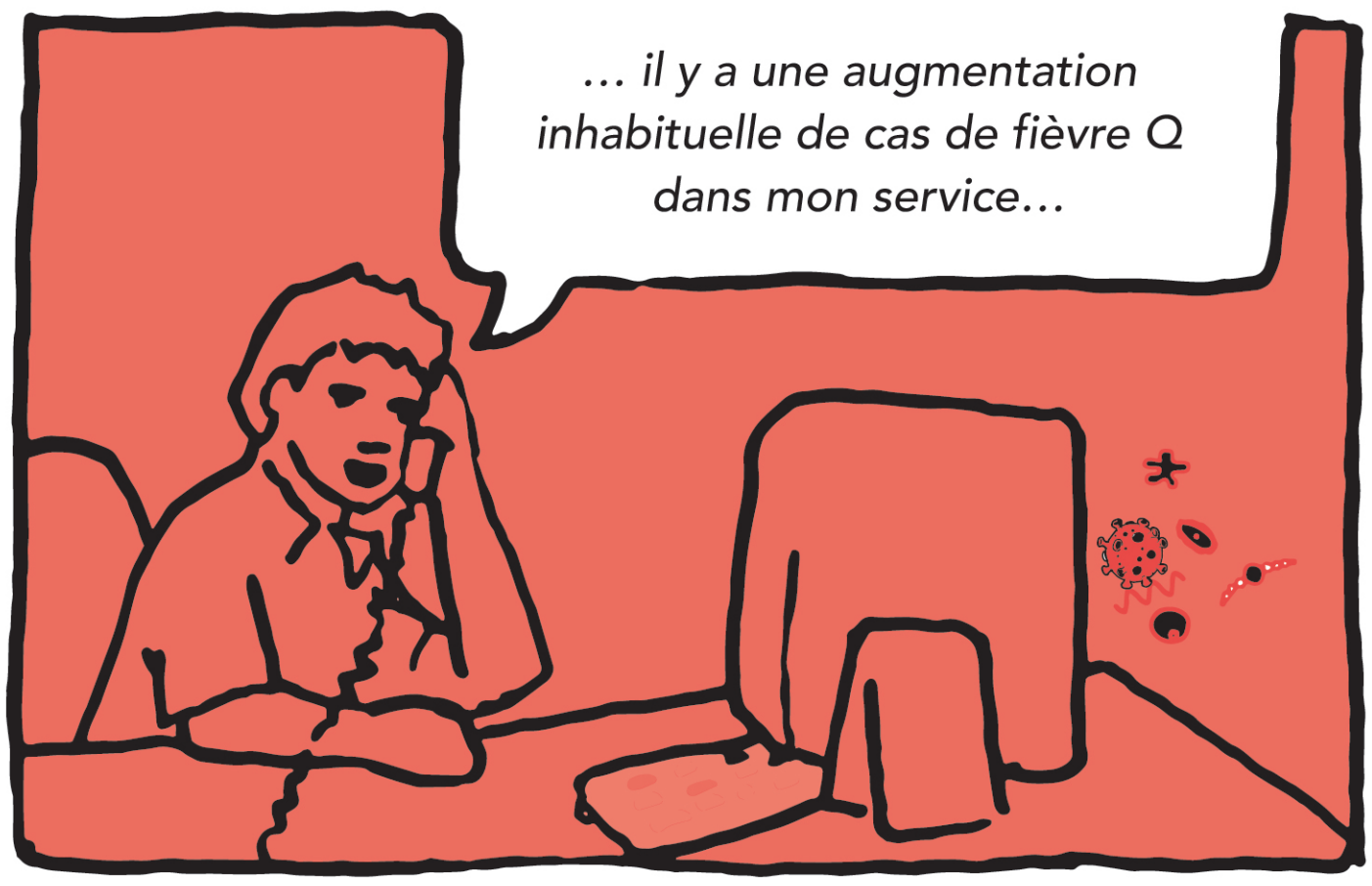

Partant de ce qui a été vu le matin, des groupes, par domaine professionnel, revisitent le moment d'alerte présenté dans la BD et proposent des scénarios alternatifs prenant en compte les échanges du matin (ce qu'on a appris des autres, ce qu'on a compris, les incertitudes mises à jour...)

mise en commun

clôture

remerciements

Nous tenons à remercier grandement les personnes enquêtées. Nous remercions également celles et ceux qui ont contribué de près ou de loin au projet EXPAIRCOX, à l'organisation des enquêtes anthropologiques et à la préparation de la journée ZOOJEU. Nous remercions en particulier, sans prétendre être exhaustifs :

• à l'ARS Nouvelle Aquitaine - Raquel Ceniceros, Renaud Pouget, Christine Ribreau, Maxime Robert

• à INRAE - David Abrial, Vanessa Boissery, Marie Brossier, Delphine Caray, Jocelyn De Goër De Hervé, Nathalie Gandon, Sabrina Gasser, François Johany, Roxane Jupin, Isabelle Lebert, Philippe Lecomte, Caroline Mignon-Falize, Didier Richard, Cyrille Rigolot

• aux GDS - Ghislain Audusseau, Manon Delalande, Nicolas Ehrhardt, Kristel Gache, Alice Jardin, Raphaël Lamothe, Karine Sommier

aux GTV - Thiéry Cazajous

• à l'Anses - Christophe Aubert, Marc Tabouret, Jaquemine Vialard

• à Qualyse - Michaël Treilles

• à l'IDELE - Renée de Crémoux

• les étudiants et encadrants du Master 2 Politiques publiques et gouvernements comparés impliqués dans les groupes de travail sur la fièvre Q l'année 2021-2022

• à l'Université Fédérale de Santa Catarina (Brésil) - Jeremy Deturche

• au tiers-lieu Le Mazier (03 Bourbon l'Archambault) et à l'association Champ des possibles (03 Saint Menoux), pour la confection des pièces de jeu.
