# Supplementary material for: ZOOJEU: Using Q fever to highlight a participatory science approach for fostering the co-design of management strategies for neglected zoonotic diseases
Source: PLoS Negl Trop Dis. 2026 Oct 15;20(10):e0014618. doi: 10.1371/journal.pntd.0014618 (PMC13671574; doi:10.1371/journal.pntd.0014618)
Supplement: Supplementary file 3 [file pntd.0014618.s007.pdf]

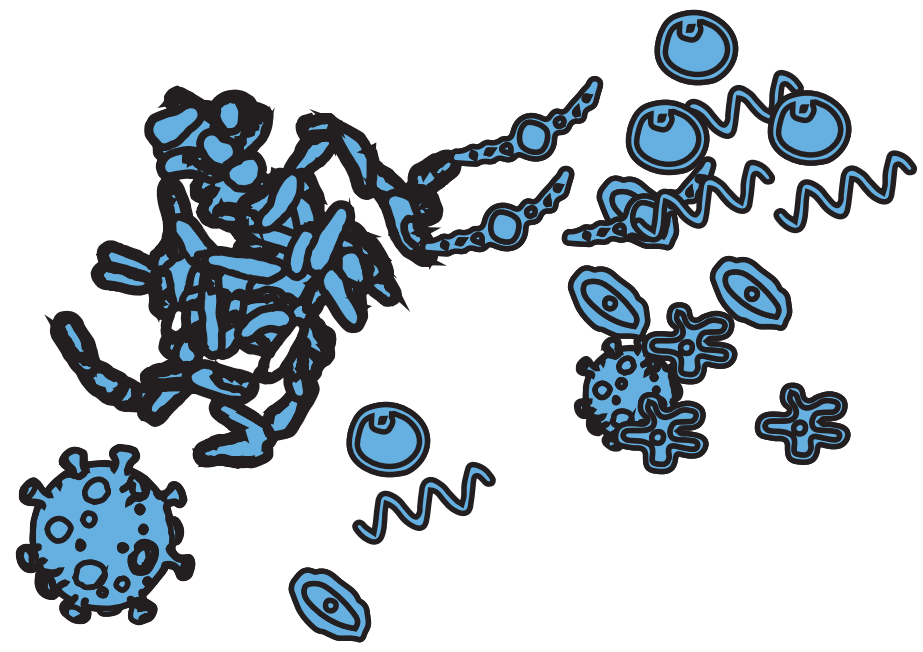

ZOOJEU

faire face aux zoonoses du point de vue des éleveurs

documents sur une expérimentation proposée par le collectif de recherche Zoojeu pour comprendre ce que fait chacun, réfléchir ensemble sur la possibilité d'une co-construction de politiques publiques sanitaires avec les éleveurs

en prenant pour exemple la prévention et la gestion de la fièvre Q dans un territoire concret (le territoire niortais) et en tenant compte de la diversité des points de vue des éleveurs

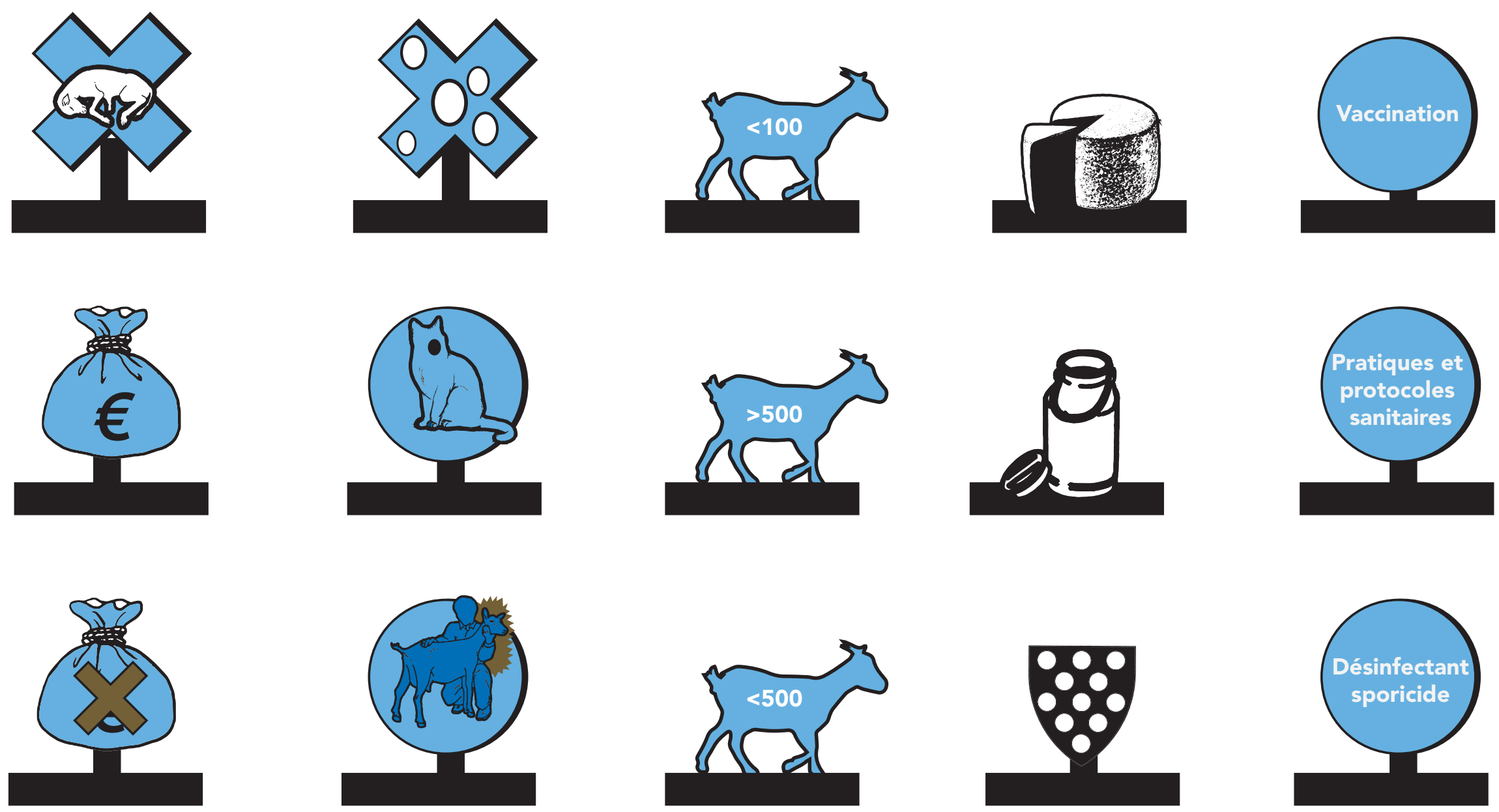

La présente brochure documente la journée d'expérimentation collective du jeudi 4 mai 2023**.** Pour cette journée, **le parti pris était de s'intéresser au point de vue des éleveurs,** directement affectés par la fièvre Q ; les autres acteurs concernés (action publique, réglementation, recherches,...) étaient pris en compte en partant de ce point de vue des éleveurs.

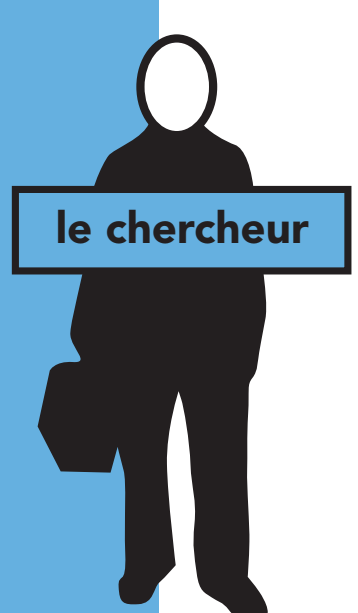

Le collectif de recherche Zoojeu est composé d'une anthropologue *(Emilie Ramillien, anthropologue au laboratoire de recherche associatif Origens Media Lab),* **d'un designer** *(Xavier Fourt, designer de recherche et d'action publique, membre du collectif d'artistes Bureau d'études),* **d'une épidémiologiste** *(Elsa Jourdain, vétérinaire épidémiologiste à INRAE),* **d'un sociologue** *(Patrice Cayre, sociologue membre du collectif Reso'them de la DGER, rattaché à l'UMR Territoires et à Origens Media Lab),* **d'une microbiologiste** *(Élodie Rousset, responsable du Laboratoire National de Référence de la fièvre Q animale à l'Anses).*
Les membres du collectif ZOOJEU n'ont aucun conflit d'intérêt.

ZOOJEU *prend pour exemple la fièvre Q, une zoonose considérée comme « orpheline » car son diagnostic et sa gestion sont complexes en santé humaine comme en santé animale en France (1). La fièvre Q ne fait pas partie des maladies réglementées mais sa surveillance et sa gestion sont considérées importantes par les institutions sanitaires pour protéger la santé animale et la santé publique (2). En l'absence de cadre réglementaire précis, lorsque des cas humains groupés surviennent, les pouvoirs publics font souvent appel à des « experts » pour analyser les risques zoonotiques, recommander des investigations et préconiser de mesures de gestion.*

En pratique, la préconisation de mesures de gestion par les «experts» peut s'avérer complexe. Cela est dû aux nombreuses incertitudes scientifiques qui subsistent sur cette maladie, pourtant connue depuis les années 1930. De plus, les mesures préconisées sont parfois trop éloignées des réalités du terrain, ce qui ajoute à la complexité de leur mise en œuvre.

Face aux difficultés récurrentes rencontrées par les autorités sanitaires « pour agir », malgré l'appui des experts, les chercheurs impliqués dans le projet EXPAIRCOX se sont tournés vers les sciences humaines et sociales pour comprendre comment cette maladie est appréhendée par les différentes parties prenantes. En effet, comment proposer des mesures de gestion adaptées aux situations de terrain si la façon dont celles-ci sont vécues par les personnes directement concernées n'est pas prise en compte dans les préconisations ? **Il est donc apparu essentiel de s'intéresser au point de vue des éleveurs, qui sont directement affectés par la fièvre Q et concernés par la mise en place de mesures de prévention et de gestion.**

ZOOJEU est un projet de sciences collaboratives *dont l'objectif est de faire émerger une démarche mobilisatrice au sein de laquelle les différents acteurs sont à l'initiative des options de gestion des maladies d'élevage transmissibles aux humains. Plus particulièrement, ZOOJEU vise à la concertation dans l'élaboration des scénarios de prévention et de gestion des zoonoses grâce à une méthodologie innovante de collaboration entre sciences biotechniques, sciences humaines et sociales et design de l'action publique.*

Le dispositif ZOOJEU a été testé le 4 mai 2023 à Prahecq à proximité de Niort. Cette journée a réuni 23 participants : 4 éleveurs de chèvres, 12 professionnels de la santé animale (Anses Niort, GDS 79, GDS 85, GDS Poitou, laboratoire Qualyse, vétérinaires praticiens, GDS France, Direction scientifique Anses, Institut de l'Elevage), 5 professionnels de la santé humaine (MSA Poitou, MSA national, SPF Nouvelle Aquitaine, ARS 79, CHU Niort) et 1 « patient-expert ».

(1) Voir les sites de l'Anses : https://www.anses.fr/fr/content/fievre-q-ruminants-humains
et de l'INRS : https://www.inrs.fr/media.html?refINRS=ZO%2011
(2) Loi connue sous l'expression de «Loi de Santé Animale» en application du
règlement d'exécution UE 2018/1882 de la commission du 3 décembre 2018
(lien : https://eur-lex.europa.eu/legal-content/FR/TXT/PDF/?uri=CELEX:32018R1882)

matinée introduction générale

Réfléchir ensemble à ce qui protège les chèvres et les humains de la fièvre Q à la ferme

Principes de la journée
Chacun était libre de se positionner comme il/elle le souhaitait et personne n'avait de compte à rendre sur ce qu'il/elle disait ou faisait au cours de la journée. L'objectif de cette mise en scène était de réfléchir collectivement à des scénarios et des positionnements possibles : il ne s'agissait donc pas nécessairement de défendre ses positions personnelles ni les obligations telles qu'elles sont mises en œuvre dans ses activités mais d'explorer un univers des possibles. En revanche, chacun conservait sa position professionnelle.

9h / accueil

9h30 > 10h15 / introduction

La bande-dessinée préalablement diffusée auprès des participants.es a été présentée sous forme de film réalisé par Élodie Rousset

• Elsa Jourdain a présenté les résultats de l'enquête épidémiologique

• Patrice Cayre a expliqué pourquoi le recours au sciences humaines et sociales était essentiel dans ce projet

• Émilie Ramillien a présenté les résultat des enquêtes anthropologiques

• Xavier Fourt a présenté les objets et le plateau de jeu

11h30 > 13h00 / jeux de plateau par groupes

//

14h00 > 15h00 / restitutions des jeux du matin

15h00 > 16h00 / réflexions sur les améliorations possibles (mêmes groupes)

16h00 > 16h45 / échanges sur les scénarios joués

16h45 > 17h00 / clôture de la journée ZOOJEU

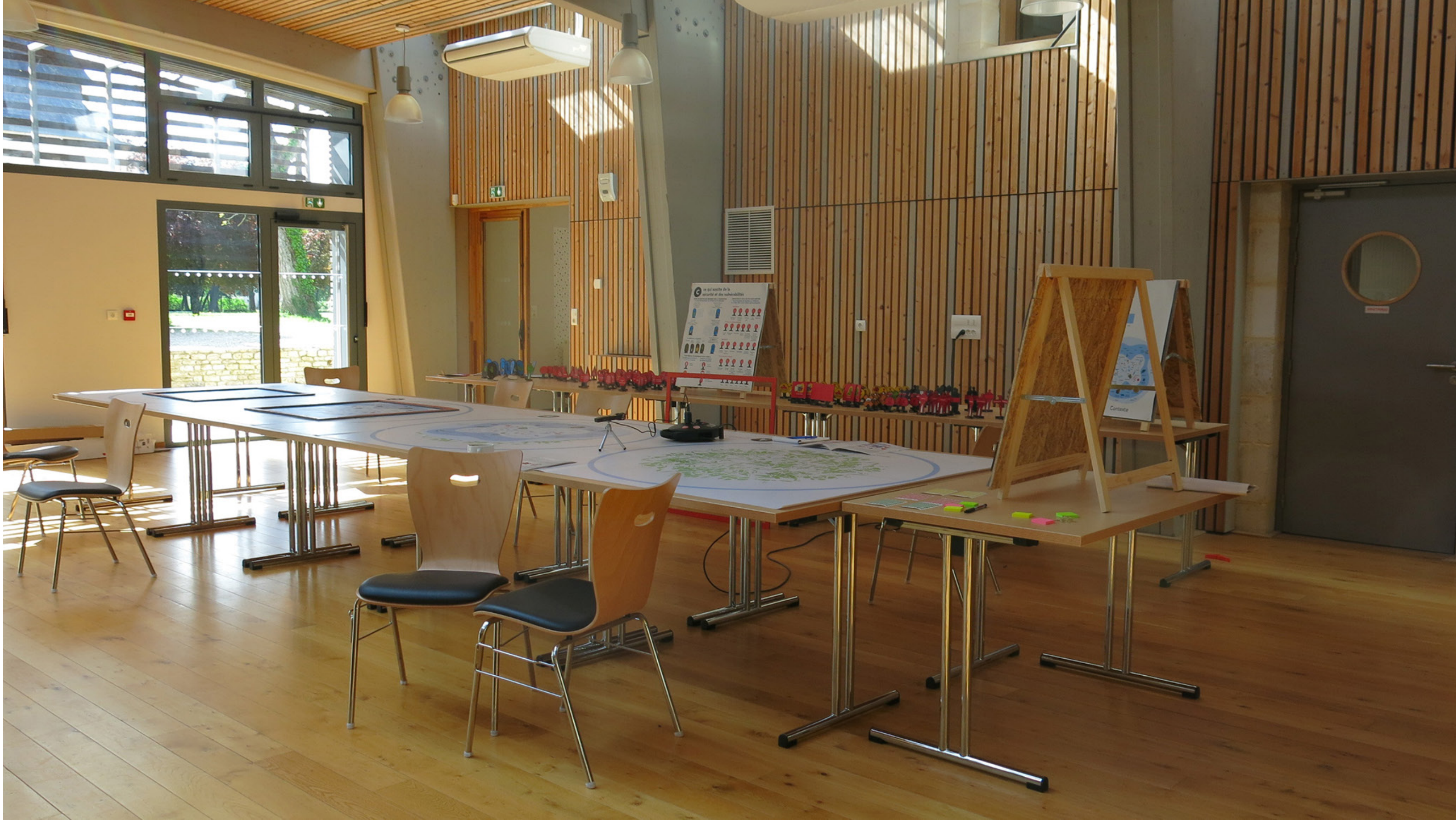

de l'enquête à l'expérimentation…
Introduction à la journée de travail

Les enquêtes anthropologiques
Sur un plan méthodologique, l'anthropologie est apparue comme la discipline la plus appropriée pour « écouter » tous les points de vue, tenir compte « des différentes réalités », sans rapport de pouvoir *a priori*. Des enquêtes anthropologiques, « prenant au sérieux » le point de vue des éleveurs, ont donc été réalisées en amont de cette journée pour révéler les modes de relations que ces derniers entretiennent avec les microbes, et notamment *Coxiella burnetii*, à travers leurs pratiques. Ces enquêtes ont été conduites au sein d'élevages de petits ruminants, de statut infecté par la fièvre Q et/ou ayant des contacts avec du public ; elles ont par ailleurs été contextualisées par des entretiens complémentaires auprès de professionnels de l'élevage, la santé animale et la santé humaine et par une « observation participante » dans une réunion sur la fièvre Q organisée par un groupement technique vétérinaire en contexte de cas humains groupés.

Rendre compte des enquêtes : une bande dessinée
Les résultats d'enquêtes anthropologiques ont permis de révéler que plusieurs savoirs et expertises étaient en présence et travaillaient ensemble. Ces résultats n'étant pas nécessairement compréhensibles pour tous quand ils sont présentés dans un rapport ou un article scientifique, nous avons appliqué l'adage « un dessin vaut mille mots » et fait le choix de résumer les principaux résultats d'enquêtes sous la forme d'une bande-dessinée qui a été diffusée aux participants avant la journée du 4 mai 2023.

Expérimenter : un espace commun de travail et de réflexion
Un « espace de travail et réflexion commun » a été expérimenté le 4 mai 2023 permettant de passer des résultats d'enquêtes anthropologiques à la mise en travail entre les parties prenantes.
Le dispositif était conçu pour que chaque acteur puisse rendre compte des enjeux, des pratiques et des objets avec lesquels il est en prise, de façon éventuellement contradictoire voire incompatible avec les autres. Ce dispositif avait pour visée de vivre une expérience permettant de dégager ensemble les conditions d'une gestion commune.

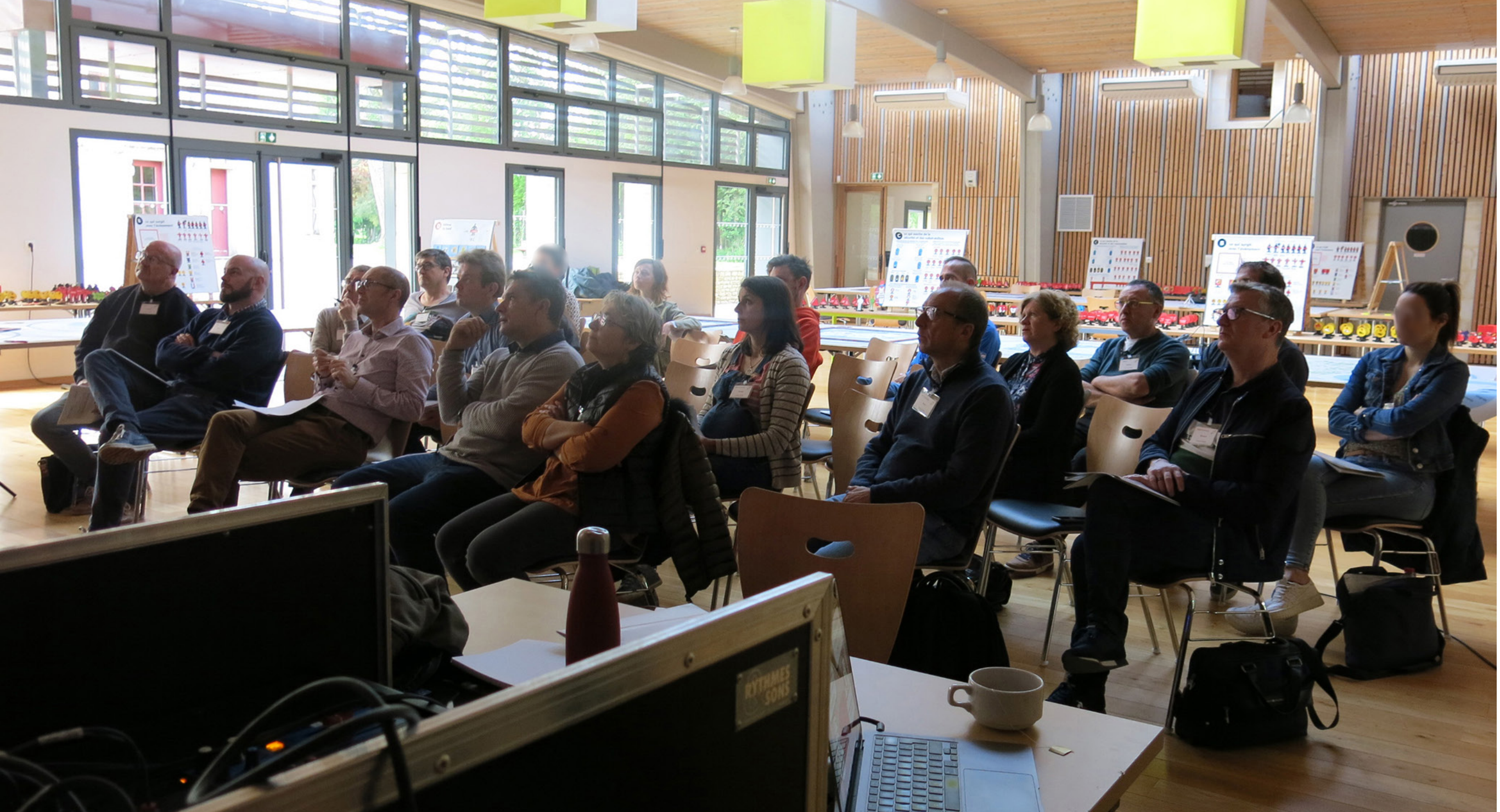

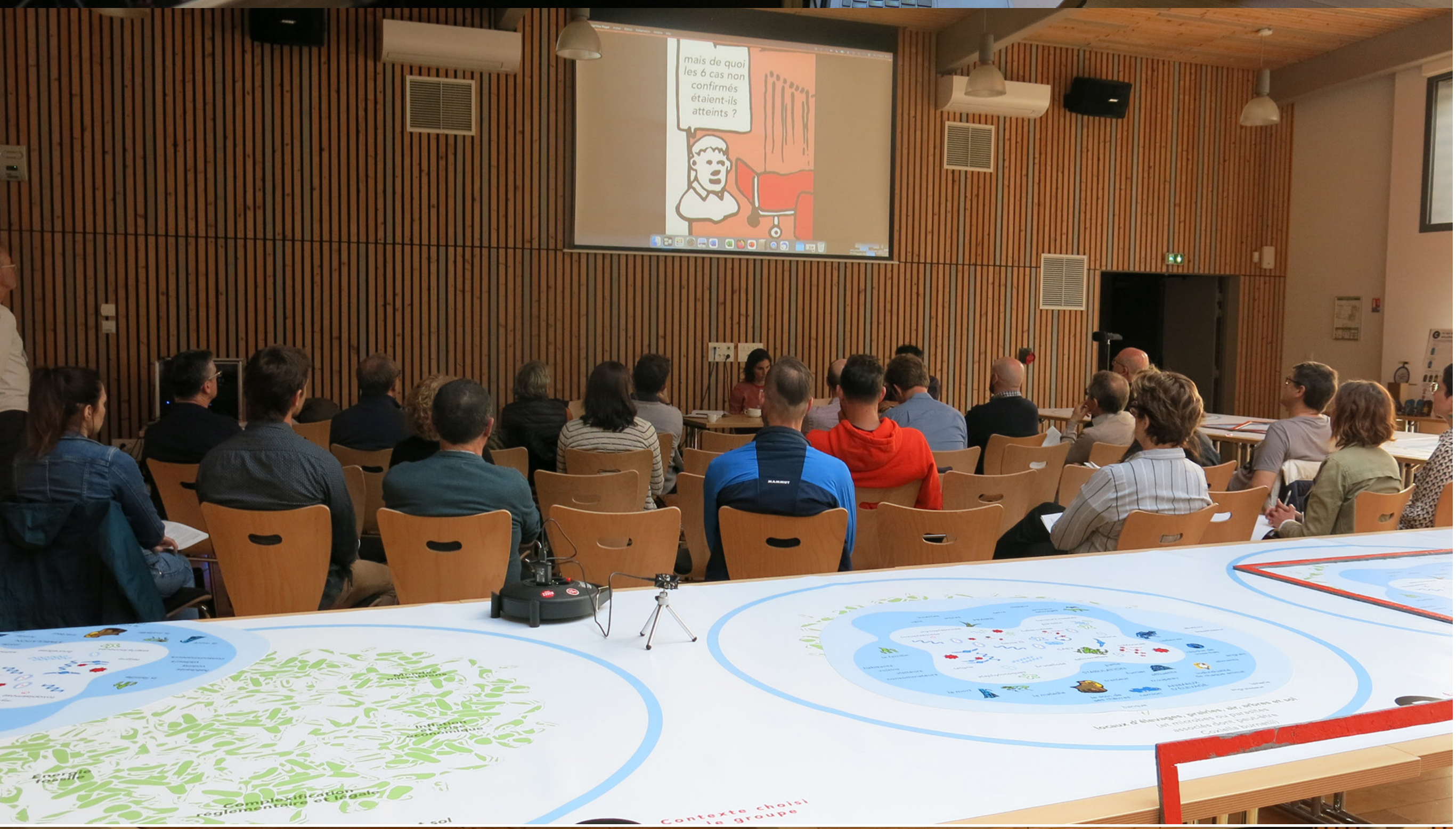
mais de quoi les 6 cas non confirmés étaient-ils atteints ?

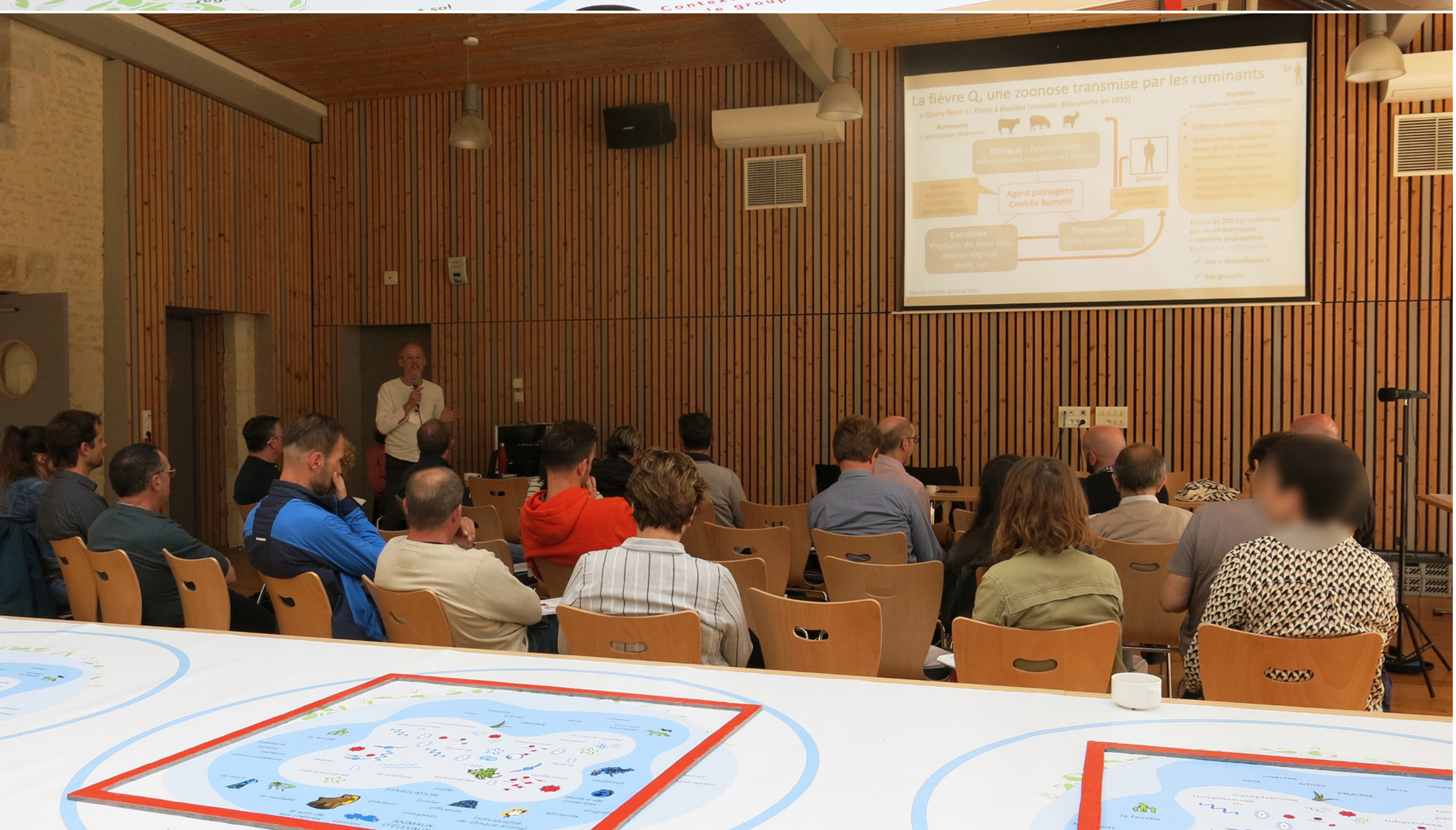
La fièvre Q, une zoonose transmise par les ruminants

trois équipes

Trois équipes, regroupant chacune des acteurs de santé humaine, de santé animale et des éleveurs, avaient pour objectif de dépasser une situation de crise liée à la fièvre Q et de trouver une voie commune pour en sortir. Au sein de chaque groupe, une suite de plateaux de jeu permettait à l'équipe d'évoluer autour de l'irruption d'avortements dans le troupeau.

Dans un premier temps, il s'agissait de poser le cadre de la ferme, fictive ou non, de l'éleveur ou des éleveurs présents dans l'équipe, sur un premier plateau. Des figurines indiquaient ses caractéristiques : type de cheptel, mode de production, type de production, type de marché, etc. Ensuite, l'irruption de l'évènement « survenue d'avortements » engendrait une modification des entités présentes dans la ferme : de nouveaux acteurs interviennent, certaines entités de la ferme sont affectées, la survenue de la fièvre Q perturbe la ferme et redistribue les entités d'une autre manière.

Chaque équipe mobilisait ou non ces nouvelles entités, ce qui donnait lieu à d'intenses discussions et négociations entre les acteurs présents : chacun à partir de sa propre perspective, les acteurs devaient échanger sur leur manière de voir et de faire pour établir ou non une vision commune de ce qui se déroulait dans la ferme et parvenir à proposer des pistes pour une meilleure gestion de la maladie, réellement efficace car tenant compte des perspectives et expertise de chacun.

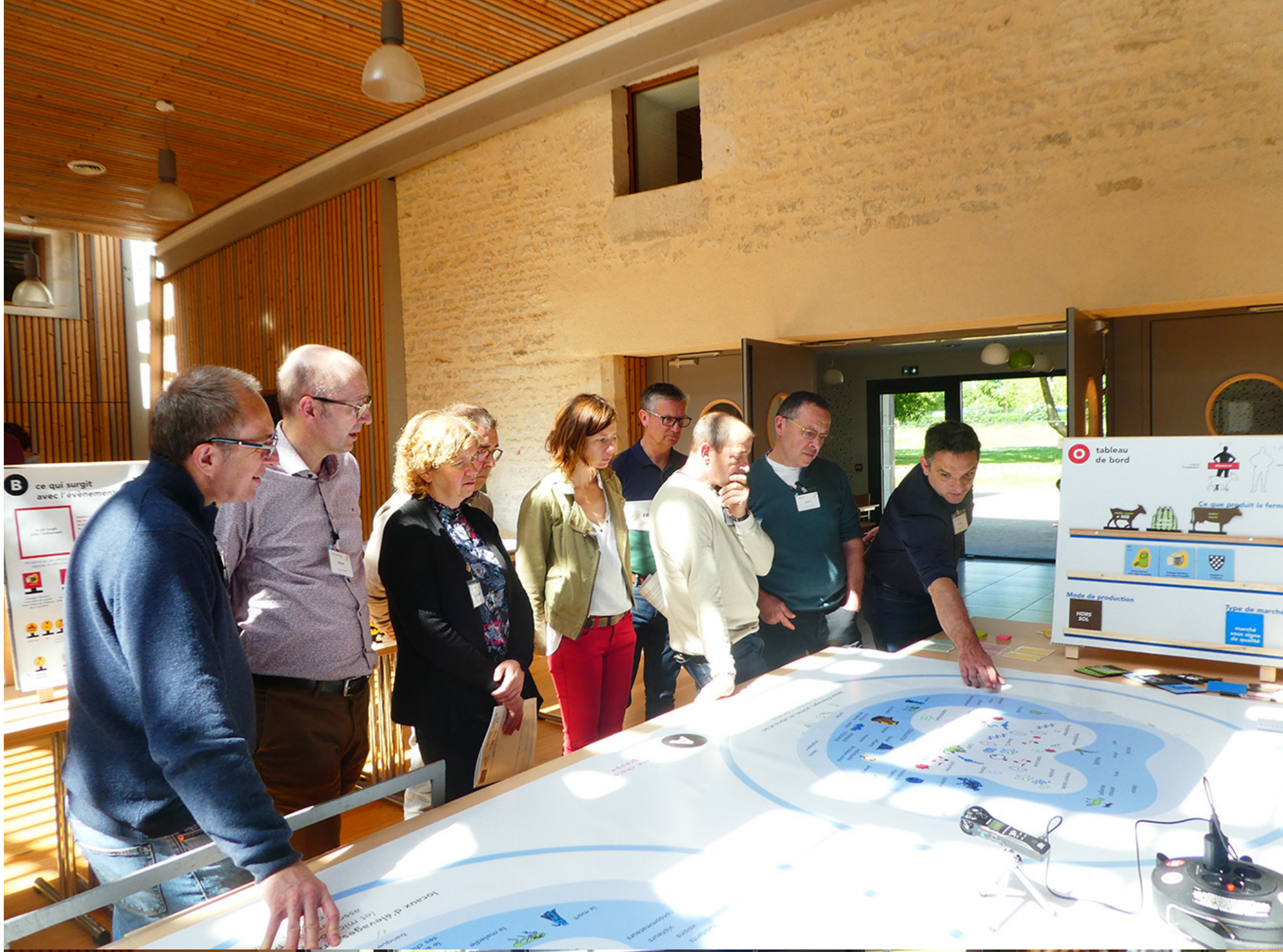

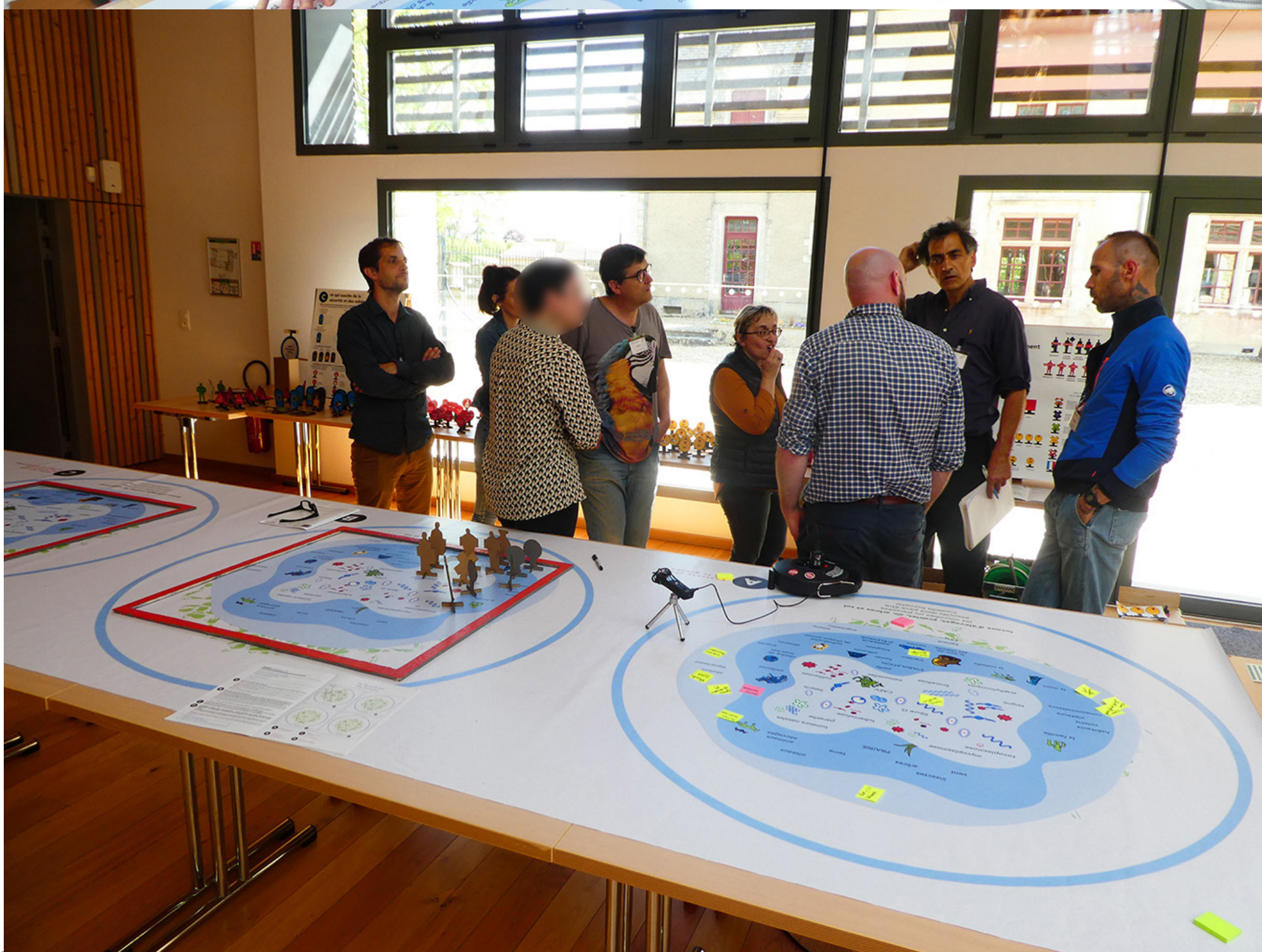

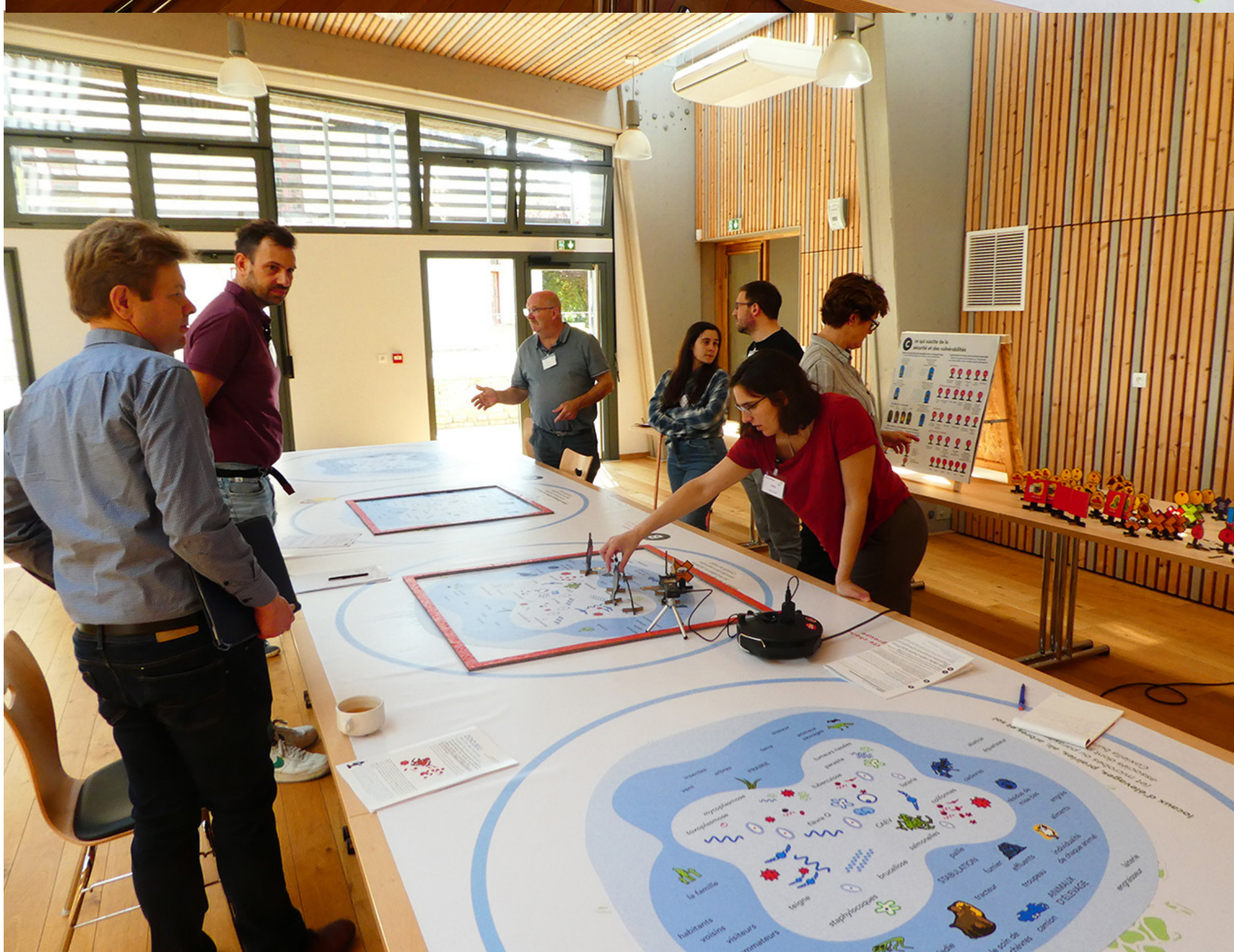

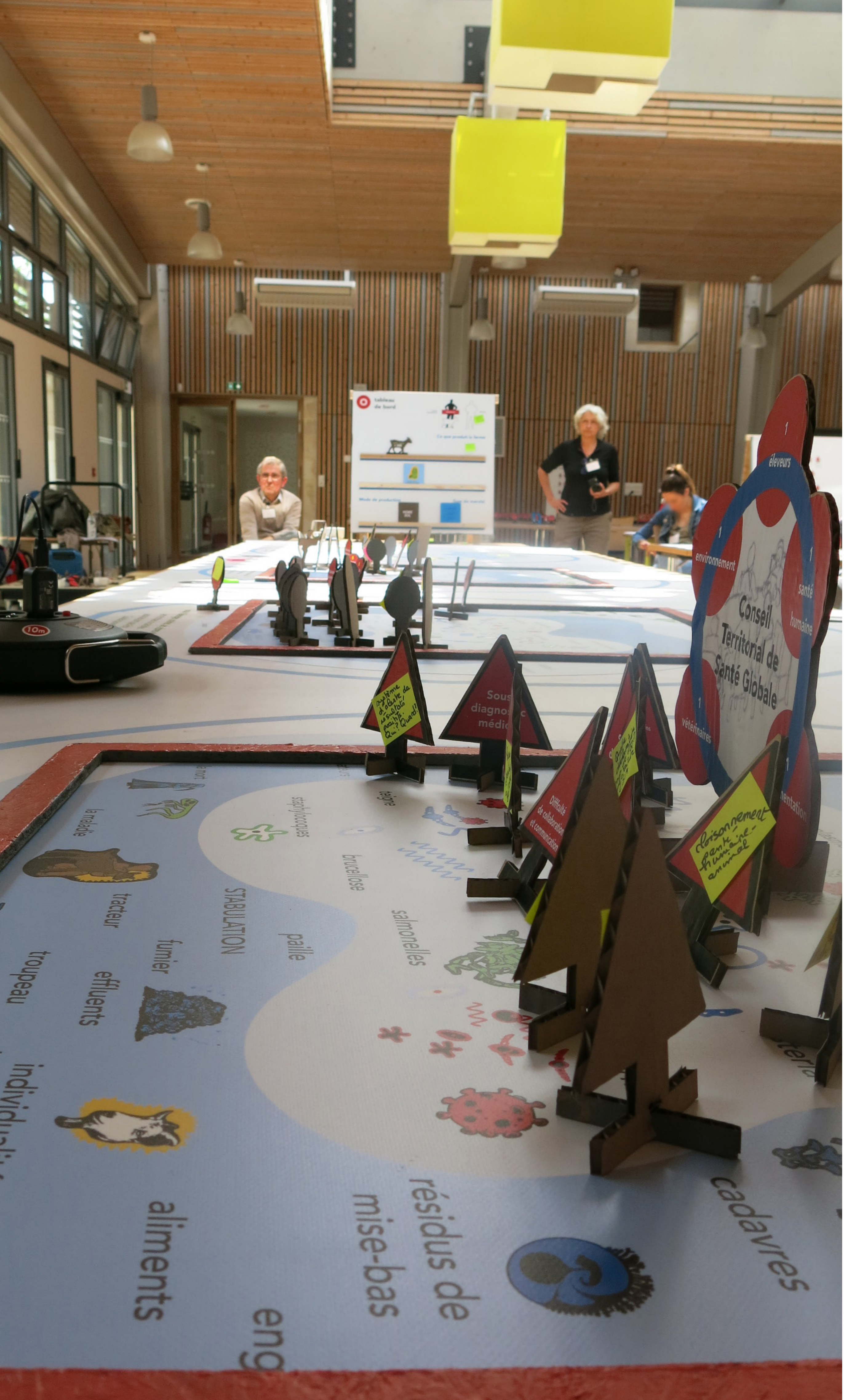

Conseil Territorial de Santé Globale
éleveurs
environnement
santé humaine
vétérinaires
Difficultés de collaboration et communication
tableau de bord
10m
la maladie
STABULATION
brucellose
salmonelles
paille
fumier
tracteur
effluents
troupeau
aliments
résidus de
mise-bas
cadavres

groupe "bleu"

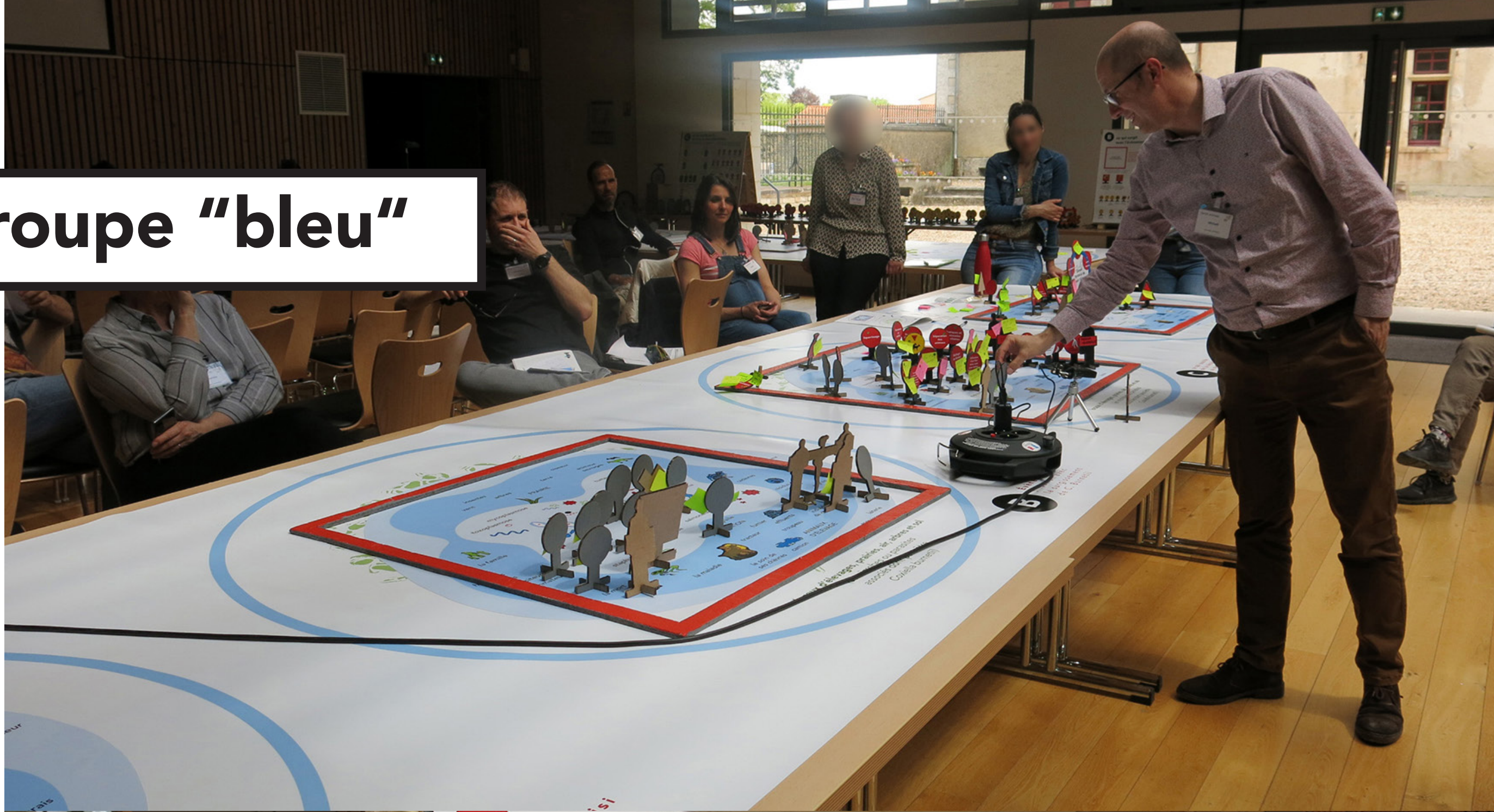

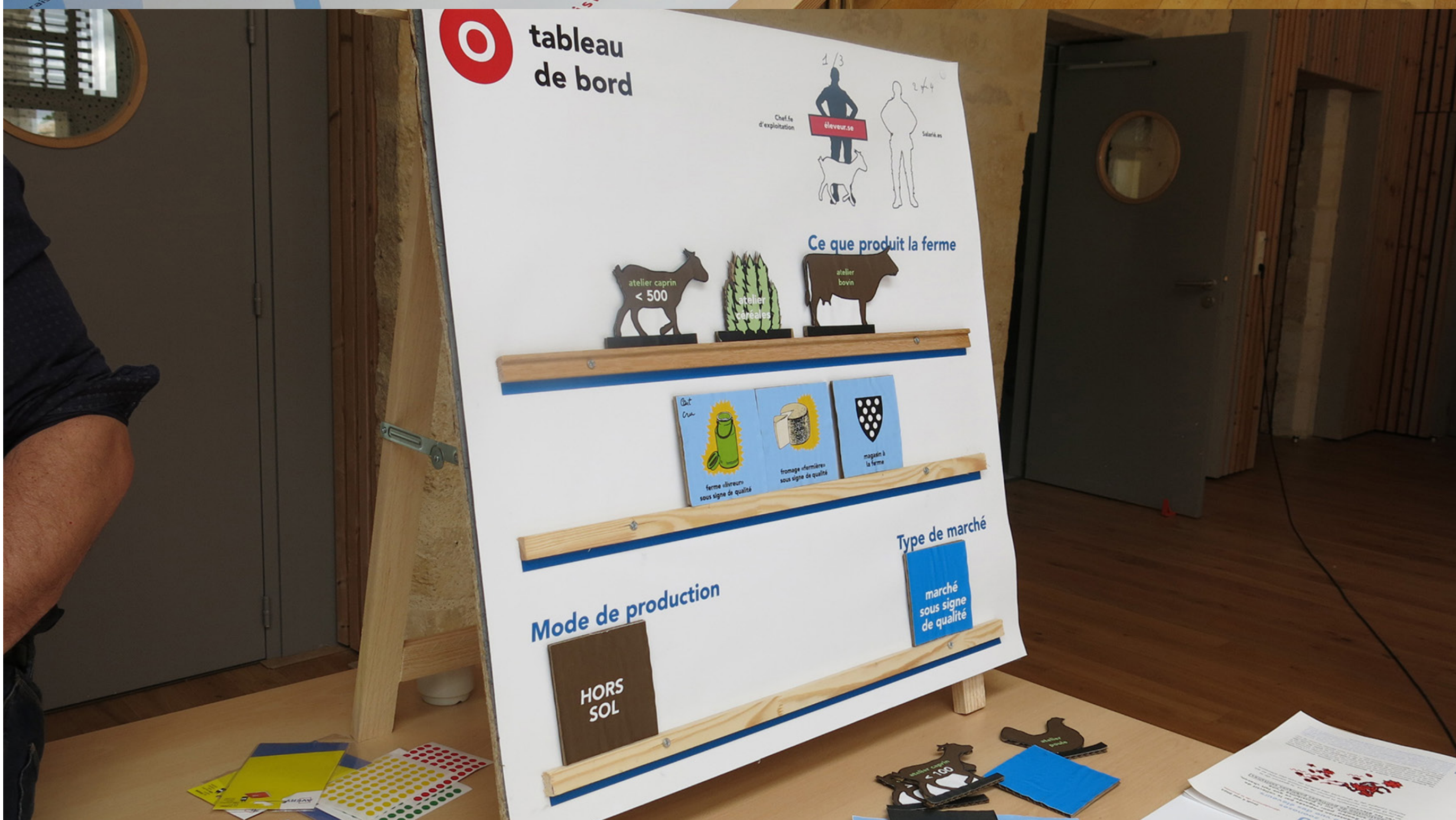
tableau de bord
Ce que produit la ferme
atelier caprin
< 500
Type de marché
marché sous signe de qualité
Mode de production
HORS SOL

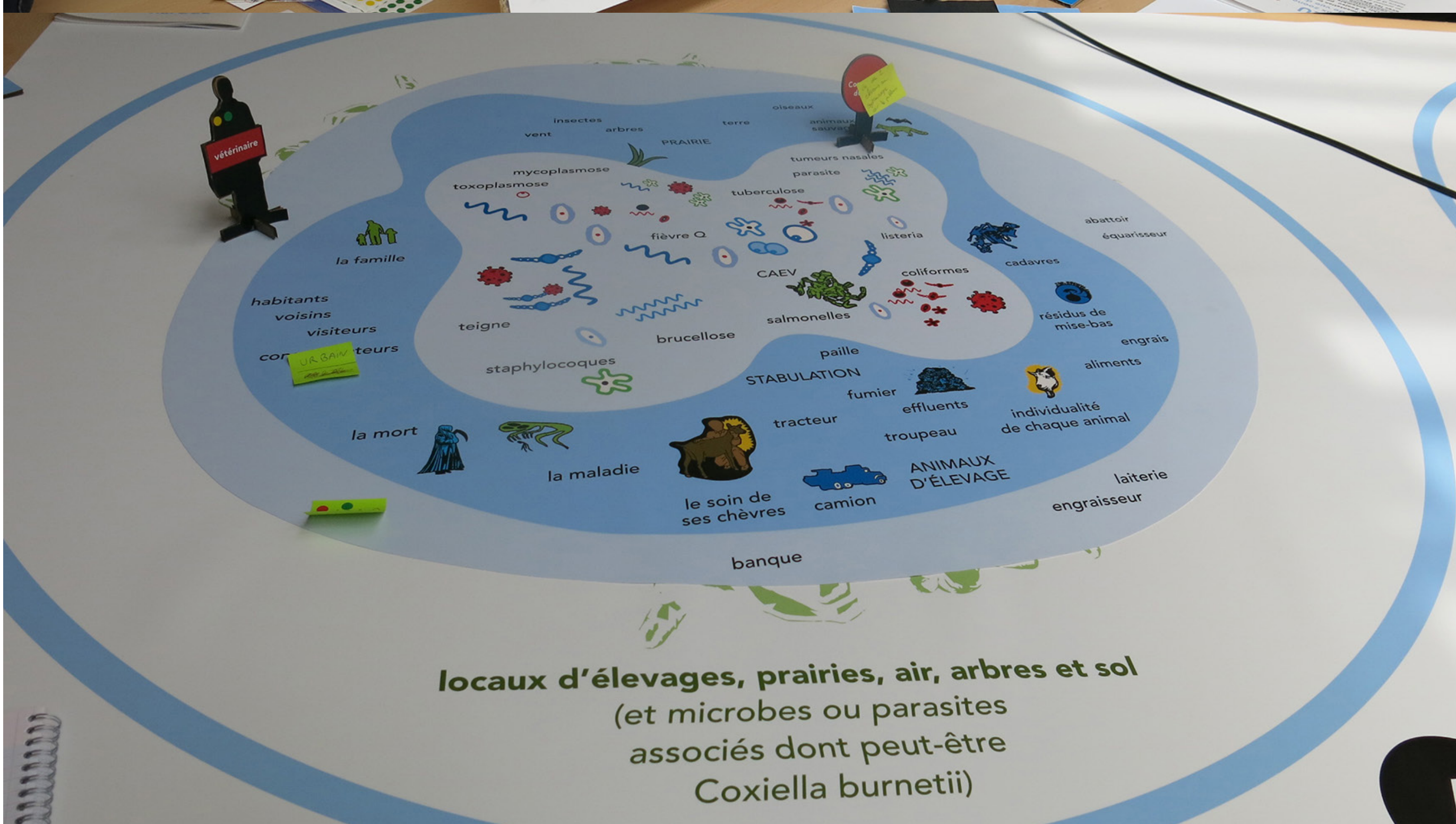
vétérinaire
insectes
vent
arbres
terre
PRAIRIE
mycoplasmose
toxoplasmose
tumeurs nasales
parasite
tuberculose
fièvre Q
listeria
abattoir
équarisseur
la famille
CAEV
coliformes
cadavres
habitants
voisins
visiteurs
teigne
salmonelles
résidus de mise-bas
engrais
brucellose
aliments
URBAIN
staphylocoques
paille
STABULATION
fumier
effluents
individualité de chaque animal
tracteur
troupeau
la mort
la maladie
ANIMAUX D'ÉLEVAGE
laiterie
engraisseur
le soin de ses chèvres
camion
banque
locaux d'élevages, prairies, air, arbres et sol
(et microbes ou parasites
associés dont peut-être
Coxiella burnetii)

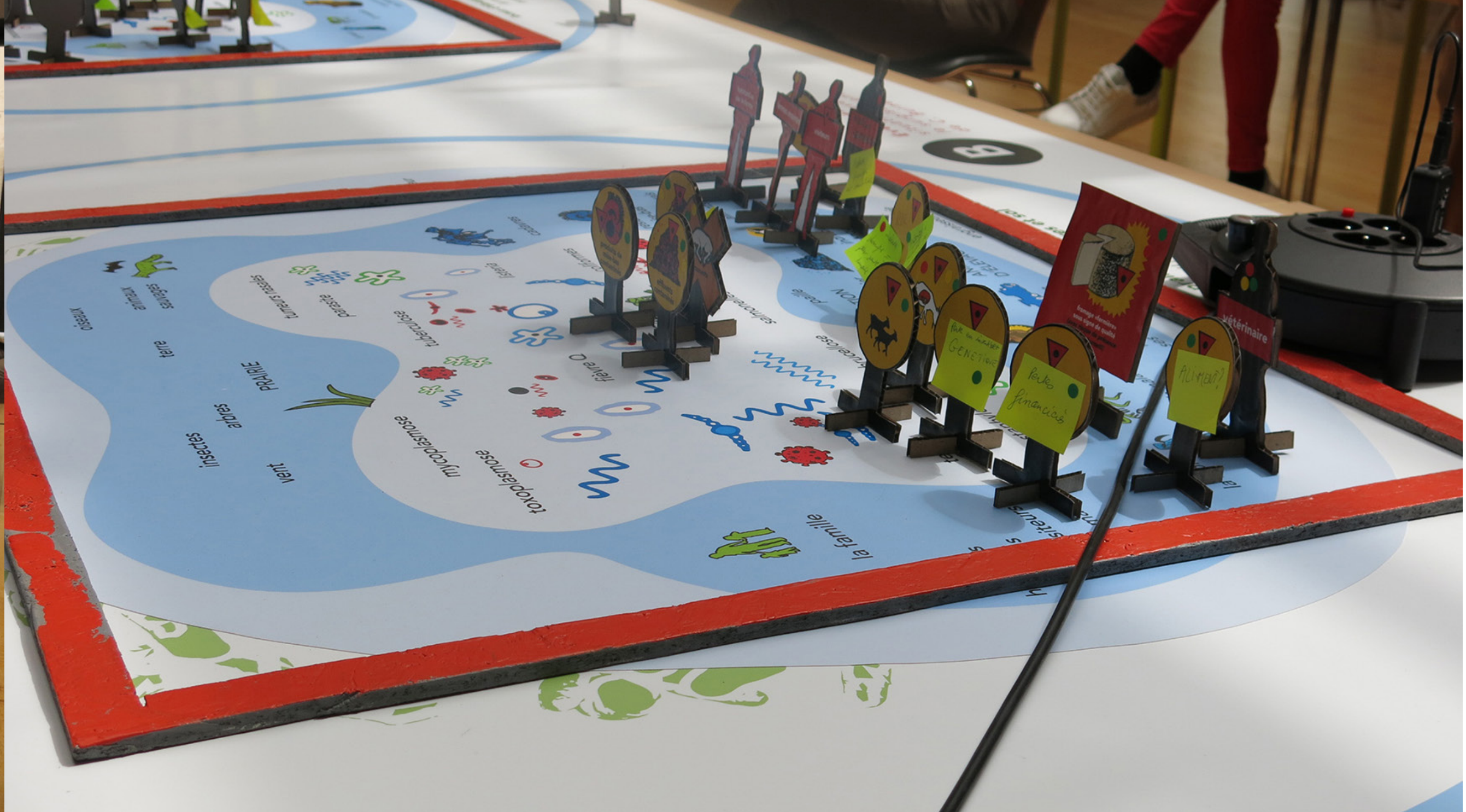
vétérinaire
la famille
mycoplasmose
toxoplasmose
fièvre Q
PRAIRIE
arbres
insectes
vent
terre
oiseaux

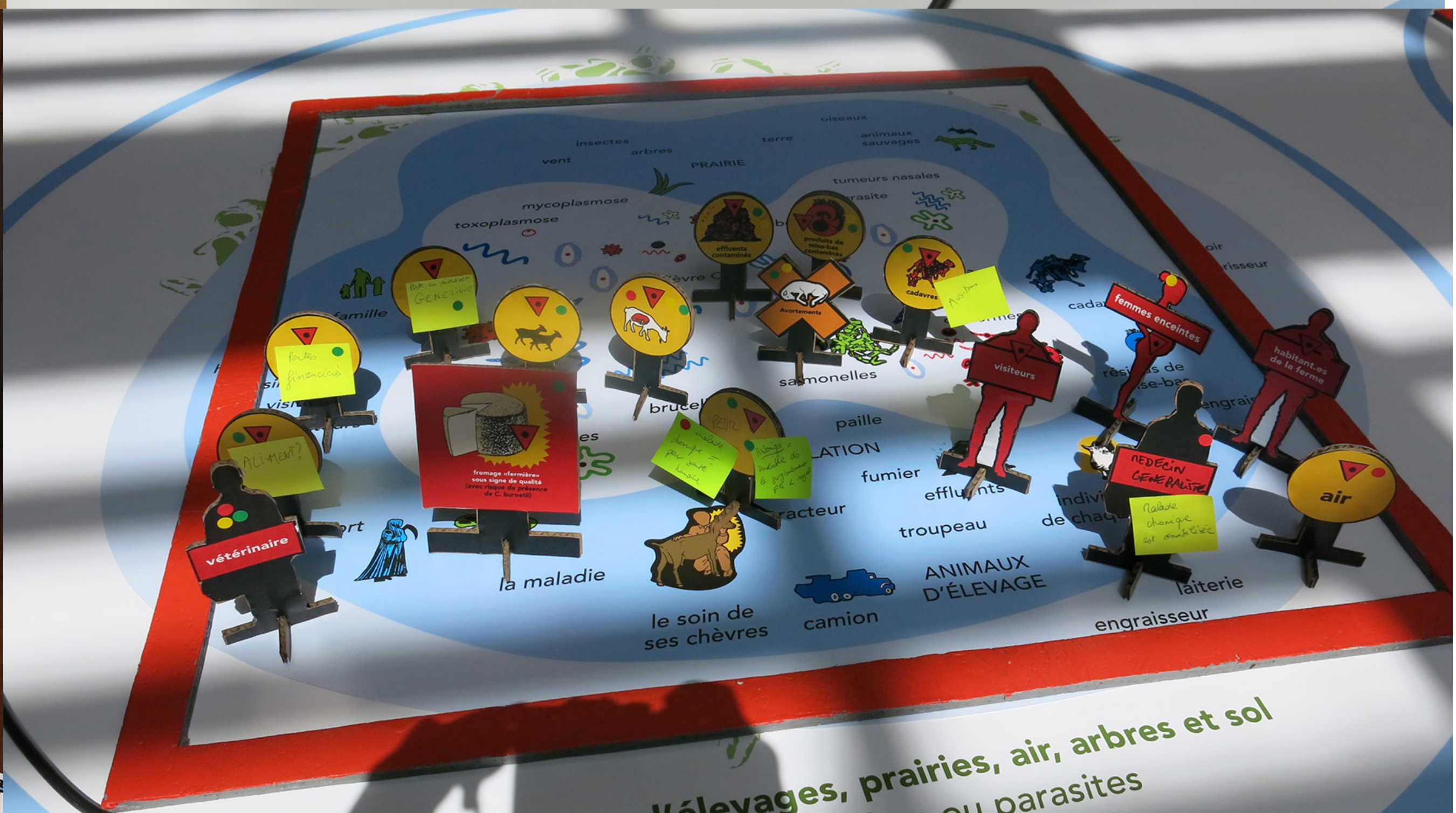
insectes
arbres
terre
oiseaux
animaux sauvages
vent
PRAIRIE
tumeurs nasales
mycoplasmose
toxoplasmose
famille
effluents contaminés
Avortements
cadavres
femmes enceintes
visiteurs
habitant.es de la ferme
samonelles
paille
fumier
troupeau
ANIMAUX D'ÉLEVAGE
laiterie
engraisseur
camion
le soin de ses chèvres
la maladie
vétérinaire
air
MEDECIN GENERALISTE
fromage «fermier» sous signe de qualité
d'élevages, prairies, air, arbres et sol
ou parasites

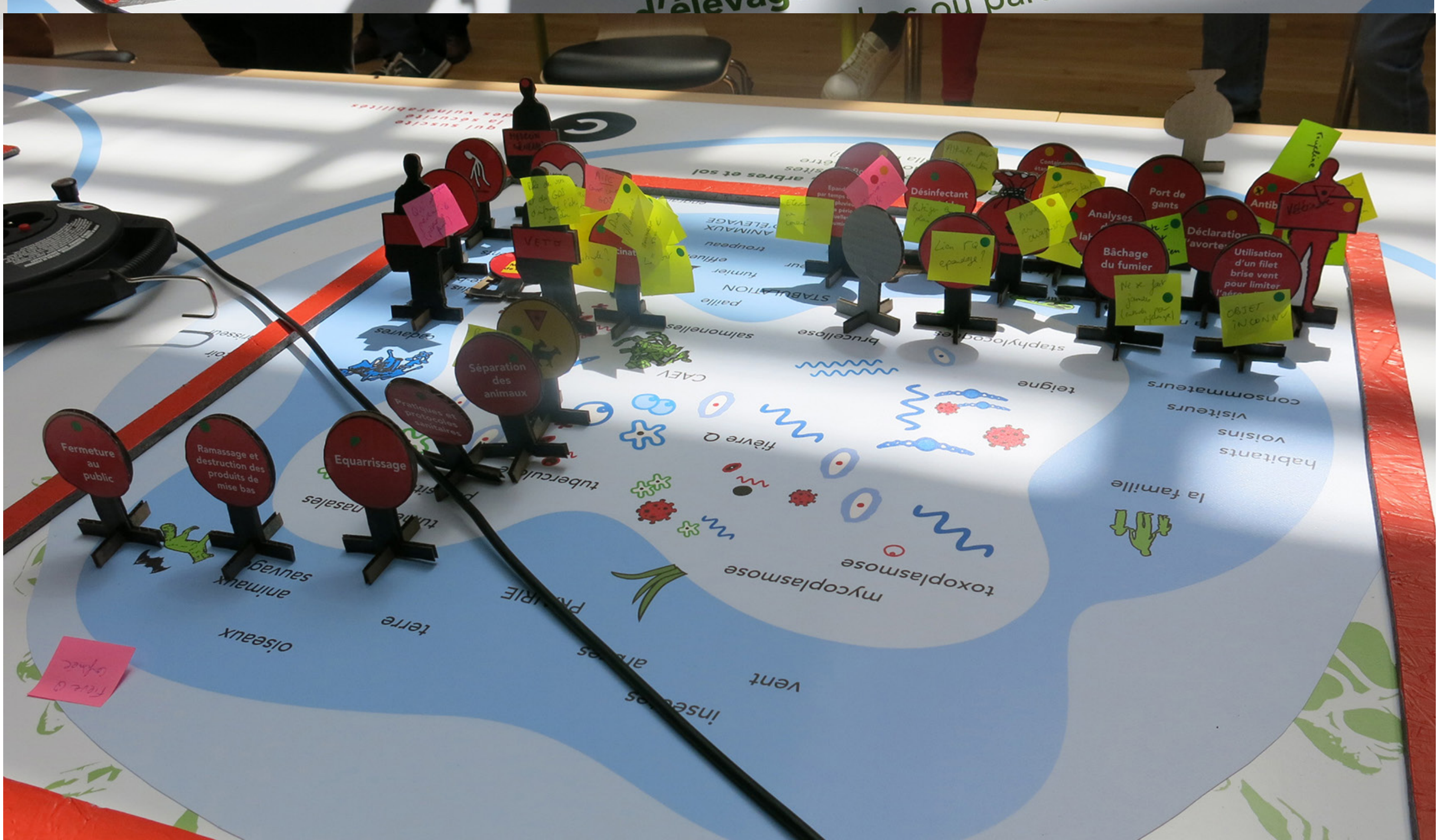
Fermeture au public
Ramassage et destruction des produits de mise bas
Equarrissage
Séparation des animaux
Désinfectant
Port de gants
Analyses
Bâchage du fumier
Déclaration avortement
Utilisation d'un filet brise vent pour limiter
VETO
tuberculose
fièvre Q
teigne
staphylocoques
brucellose
salmonelles
paille
mycoplasmose
toxoplasmose
la famille
habitants
voisins
visiteurs
consommateurs
PRAIRIE
terre
oiseaux
animaux sauvages
vent
insectes
arbres

groupe "rouge"

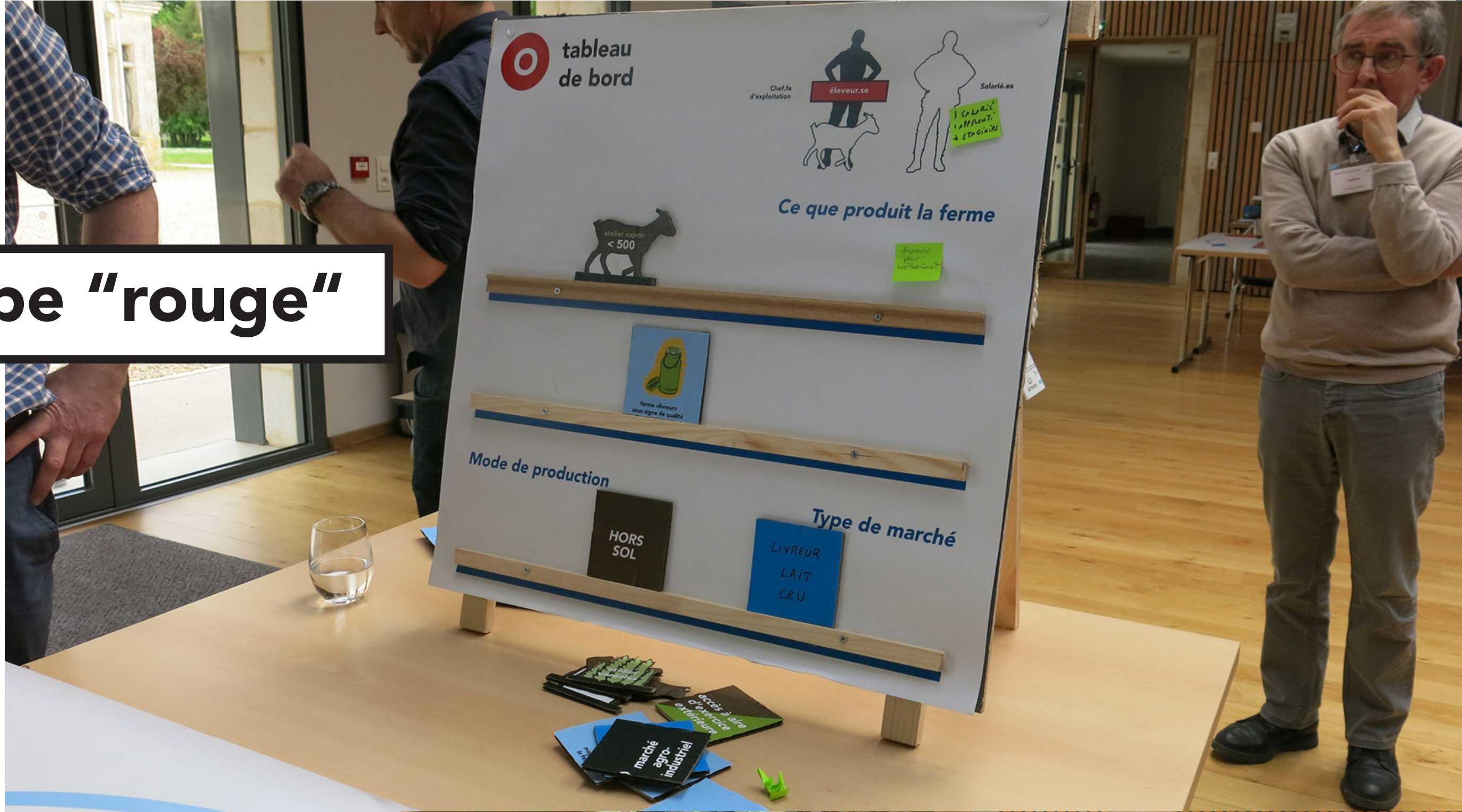

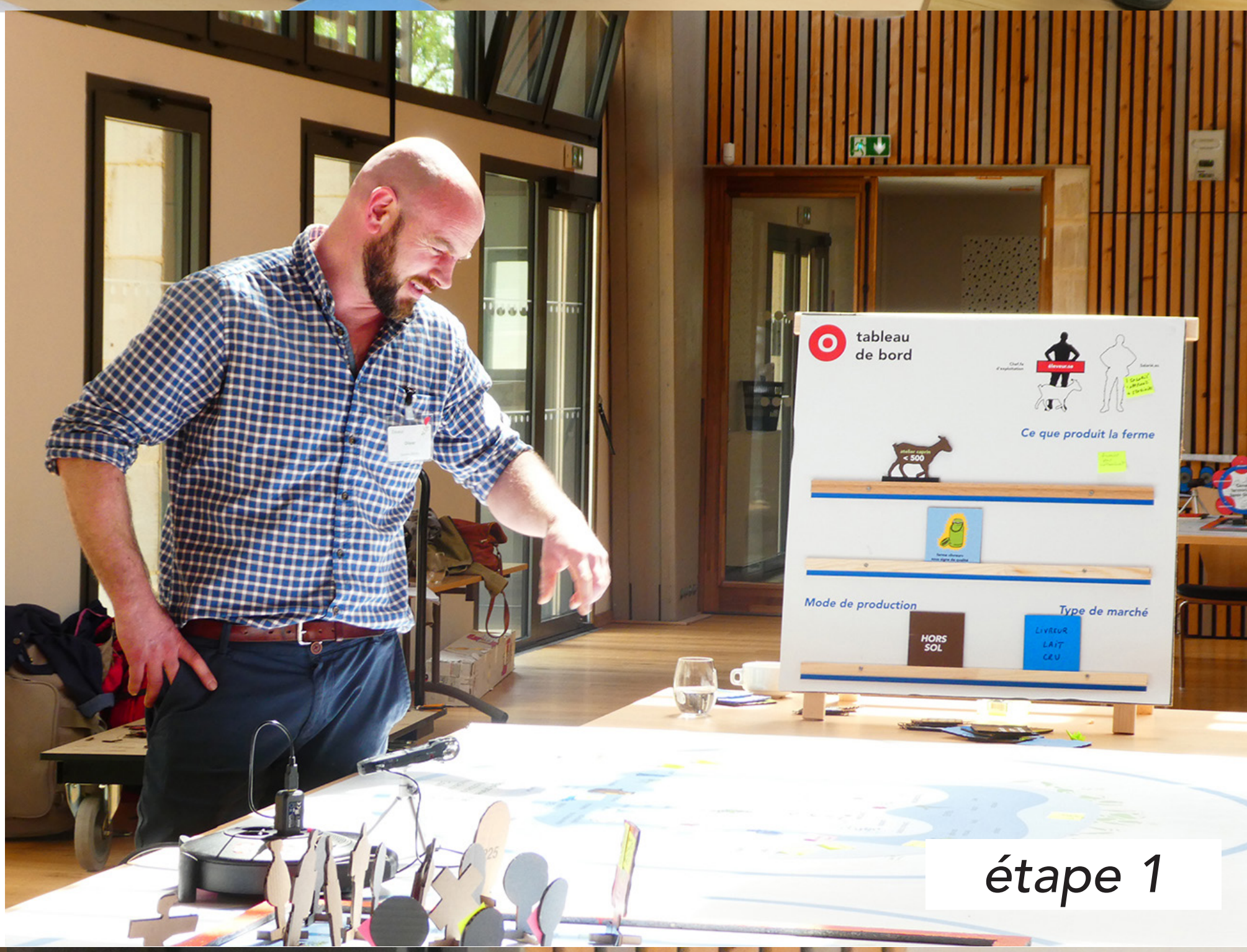

étape 1

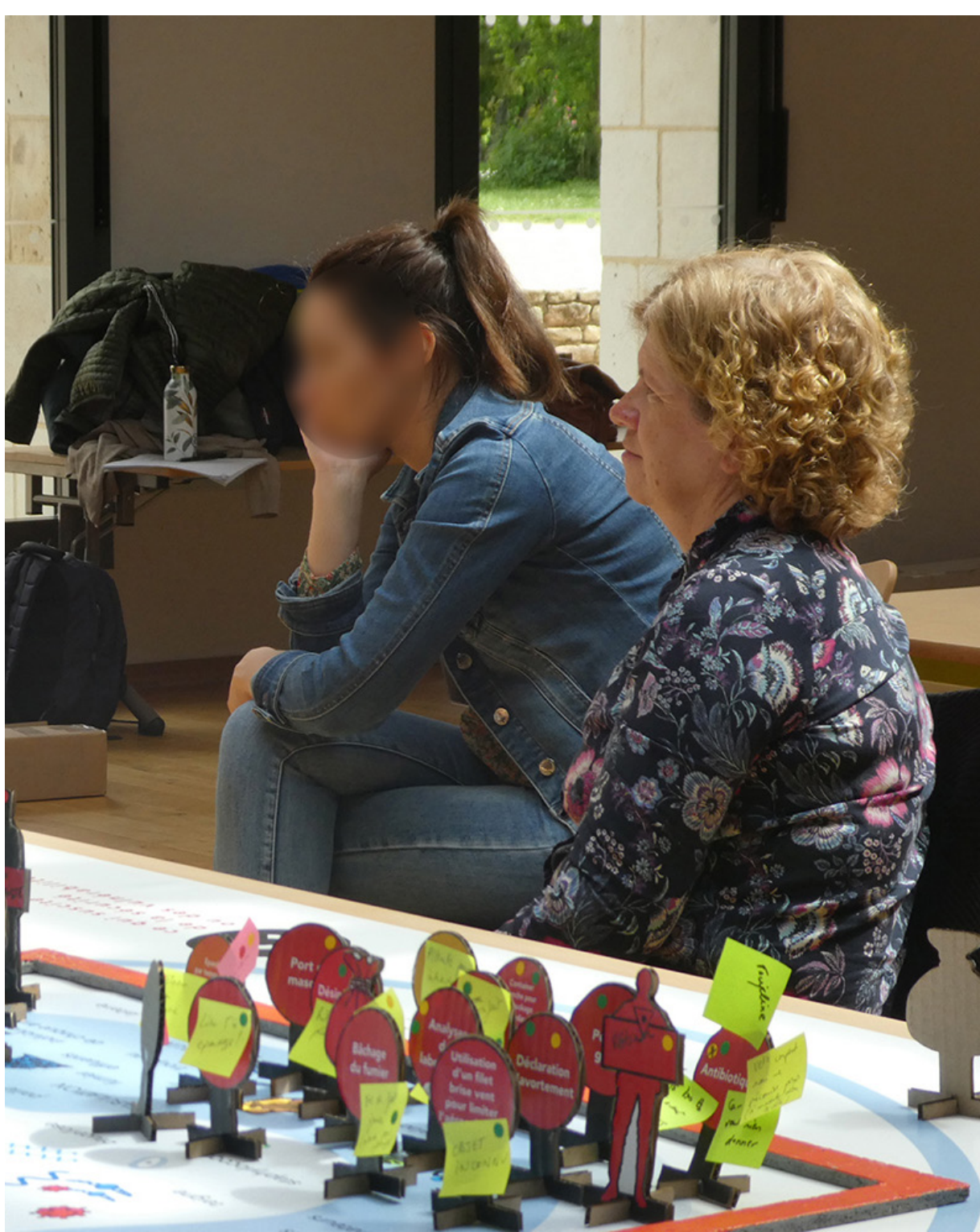

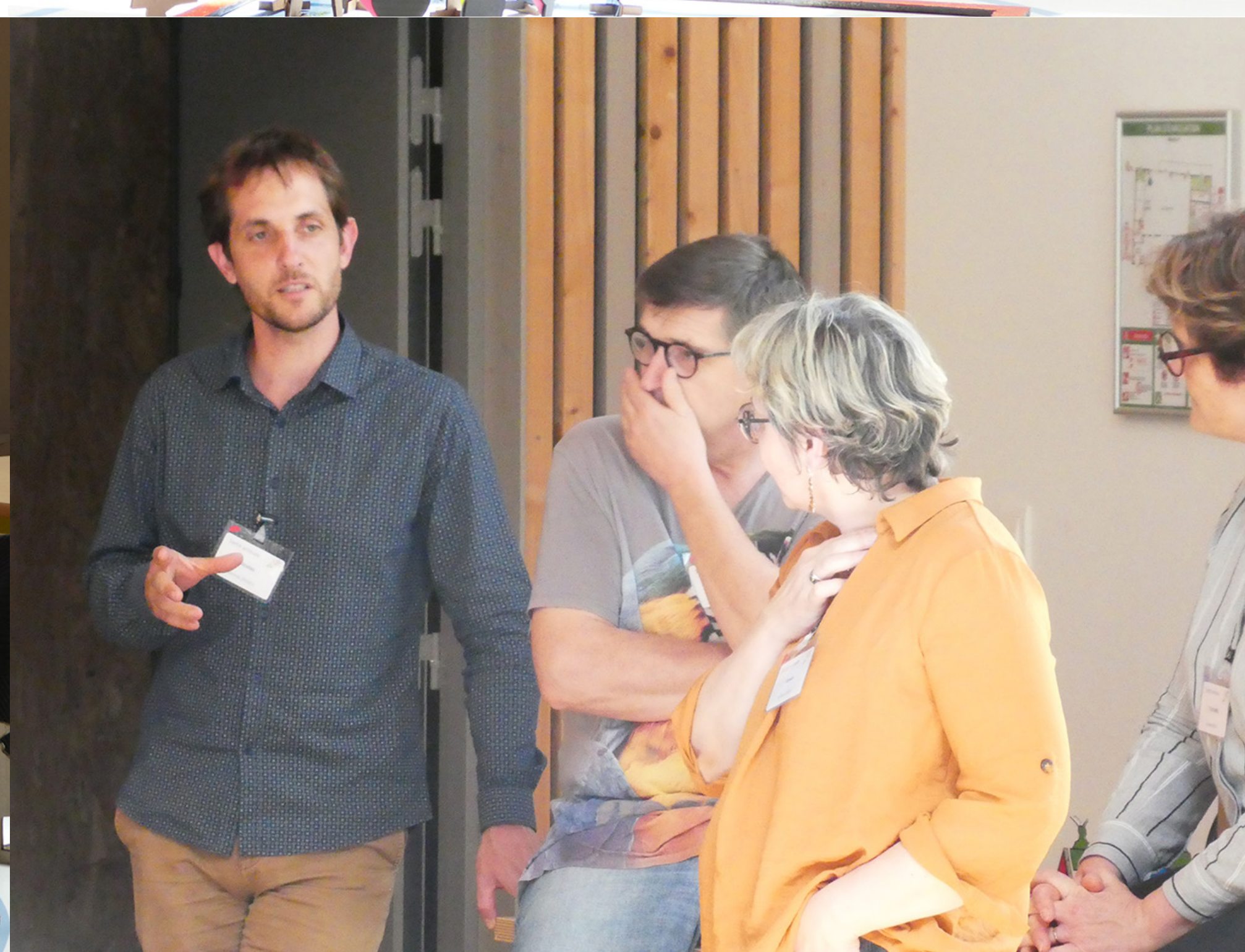

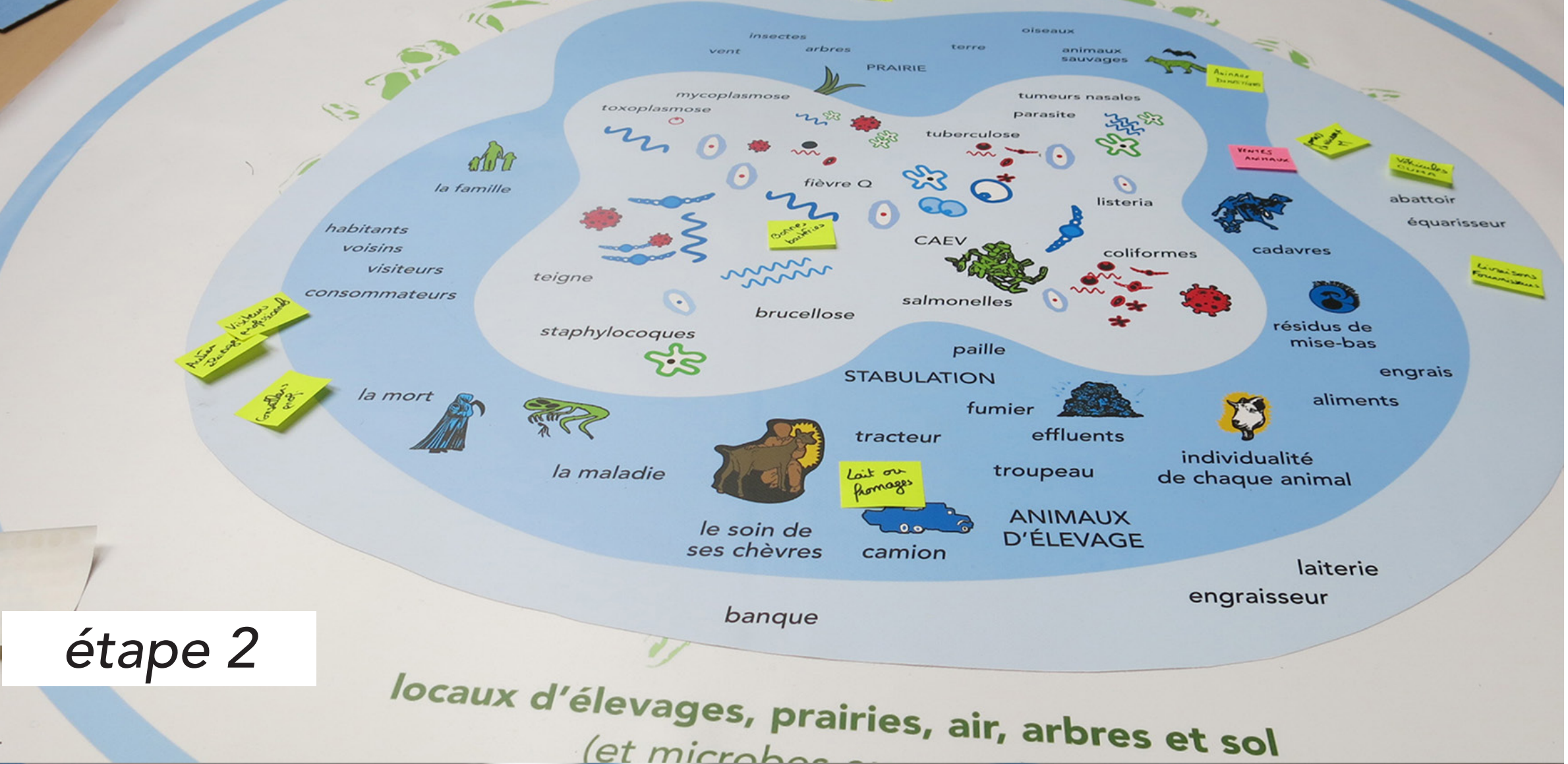

étape 2

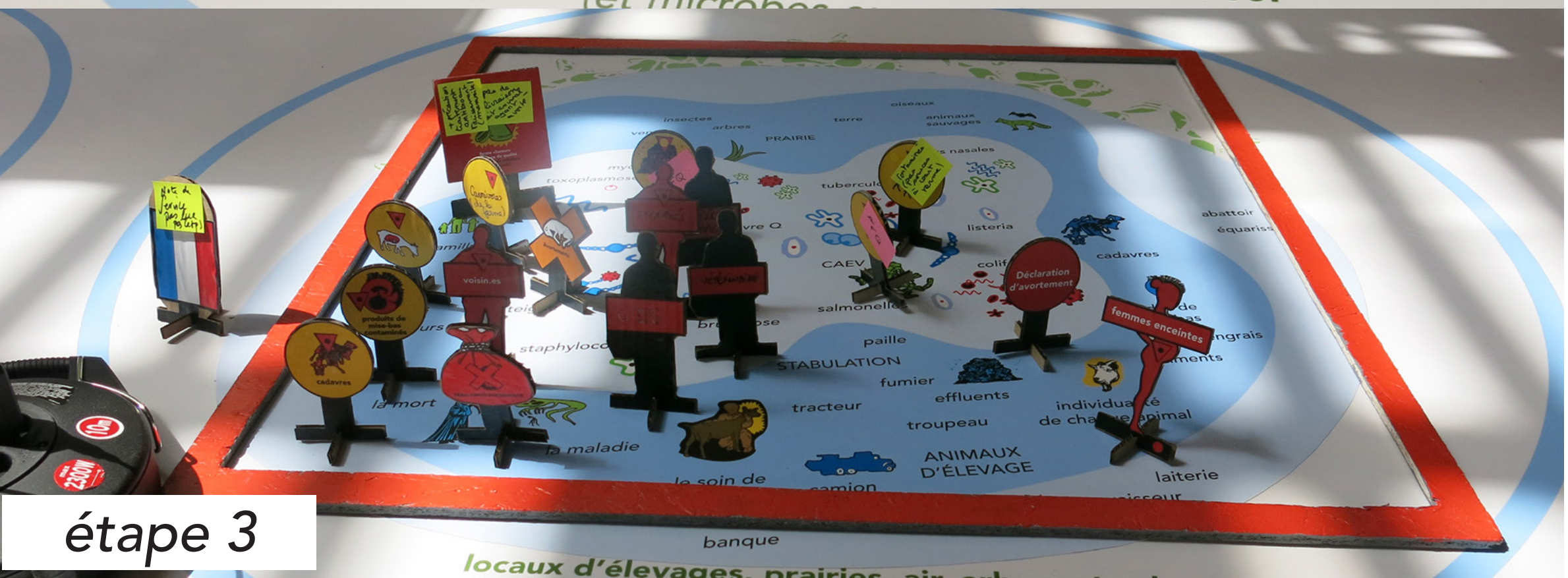

étape 3

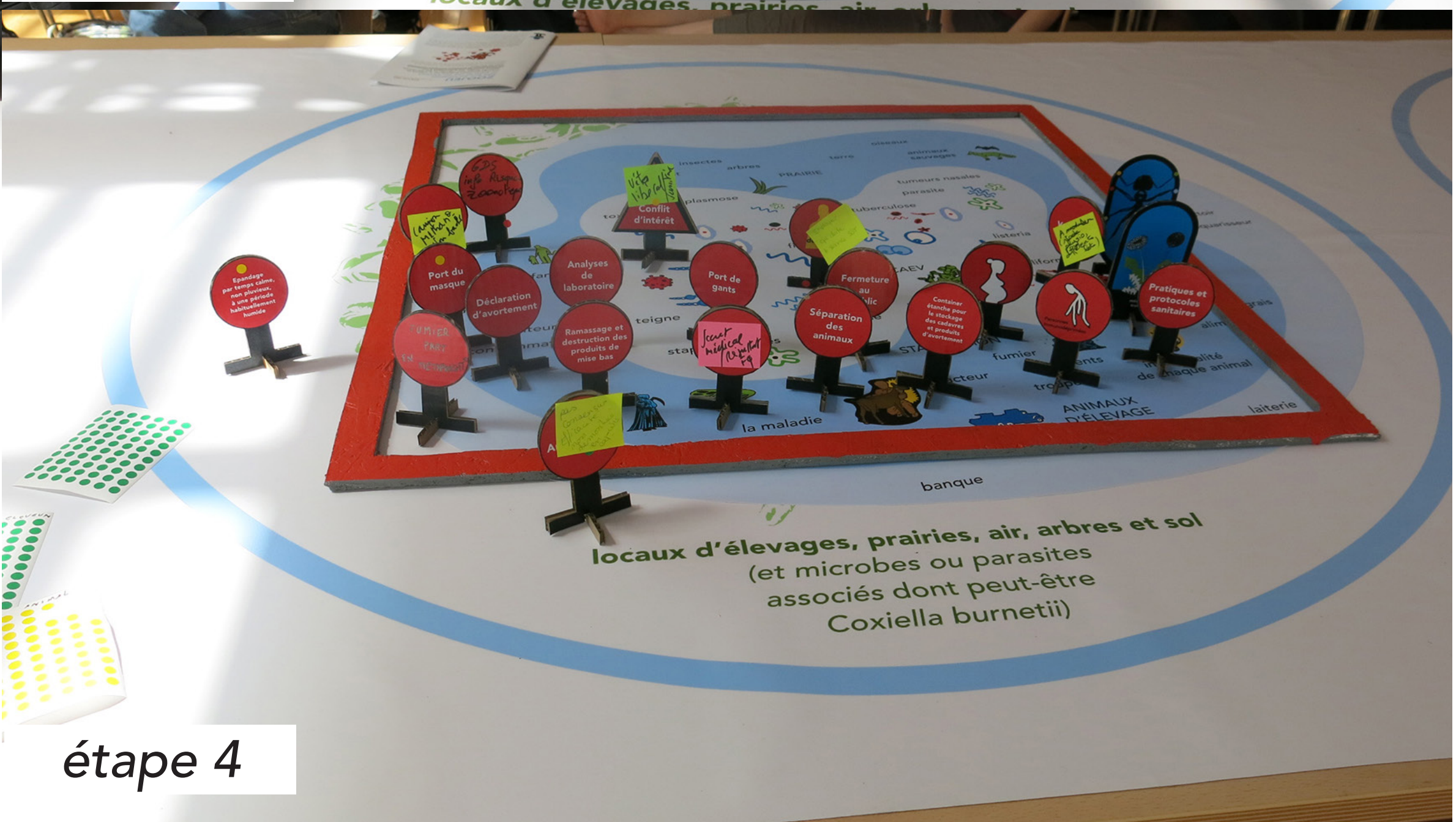

étape 4

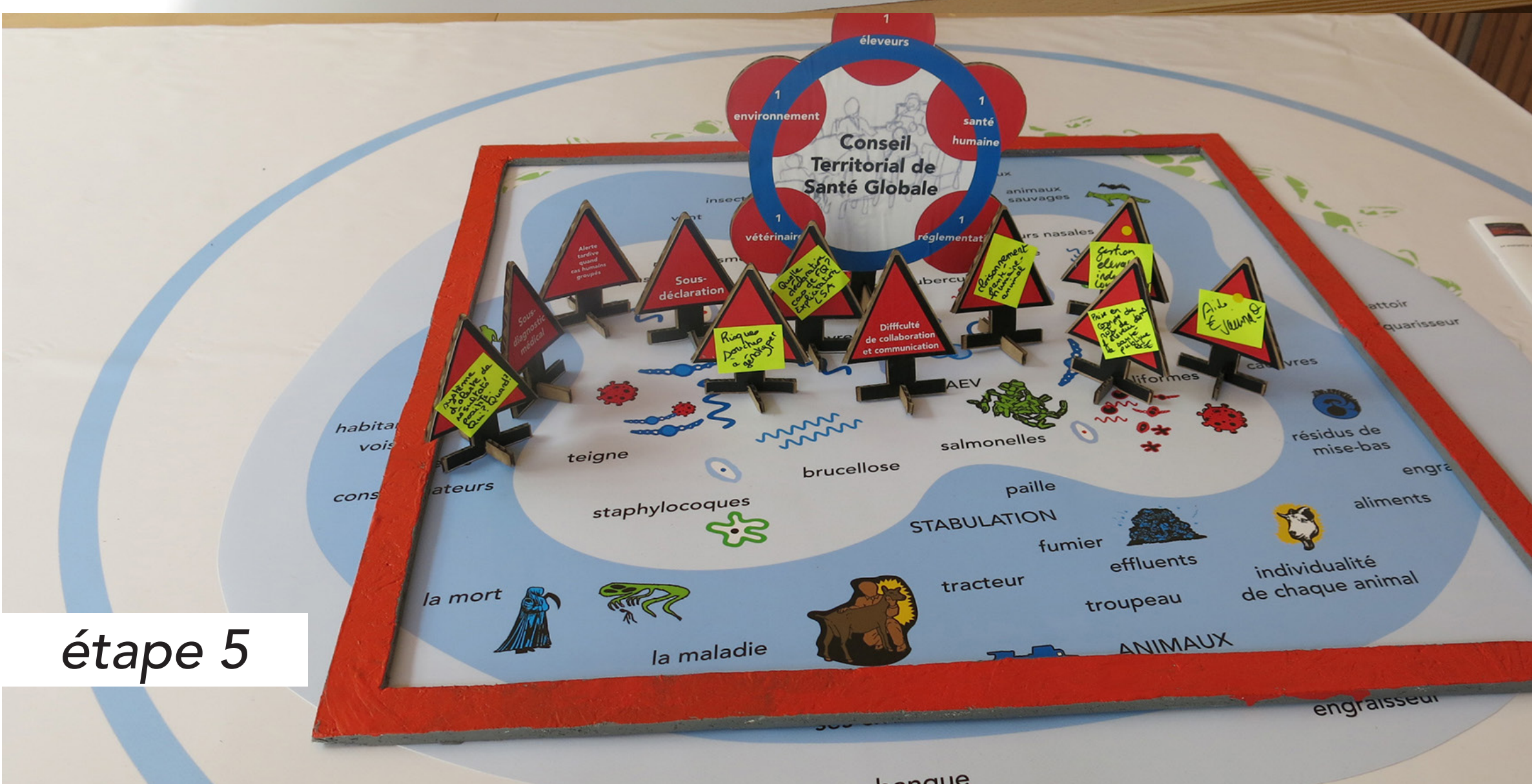

étape 5

Les échanges lors de cette journée ont été passionnants et nourris. Ils ont permis des prises de conscience au sein des équipes : comprendre la perspective d'un malade ou d'un éleveur par exemple peut permettre de repenser la manière de concevoir les options de gestion de la maladie. De la même façon, échanger autour de la question du risque permet de comprendre pourquoi les actions engagées par les uns et les autres diffèrent, et de repenser une notion de risque qui soit partagée.

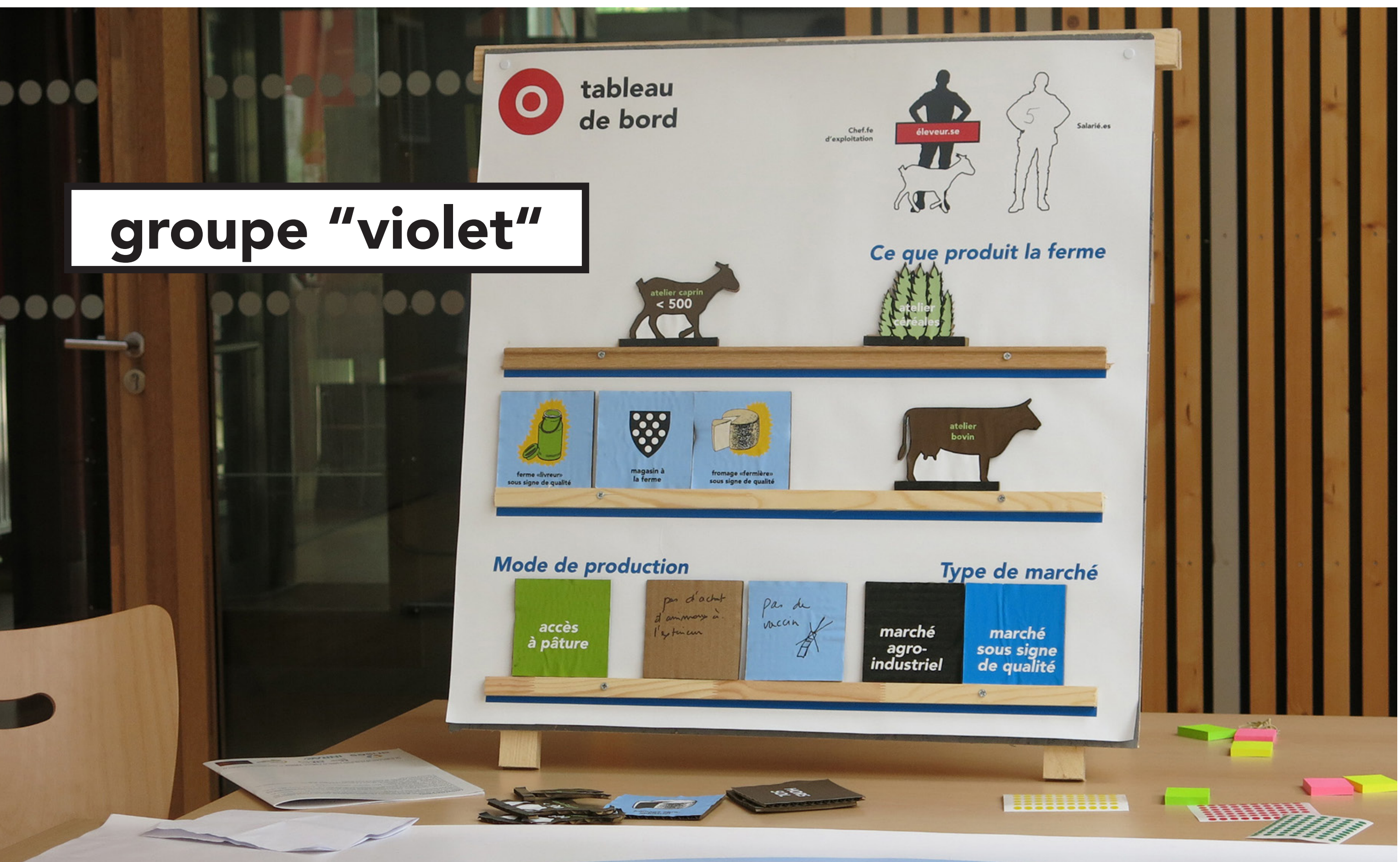

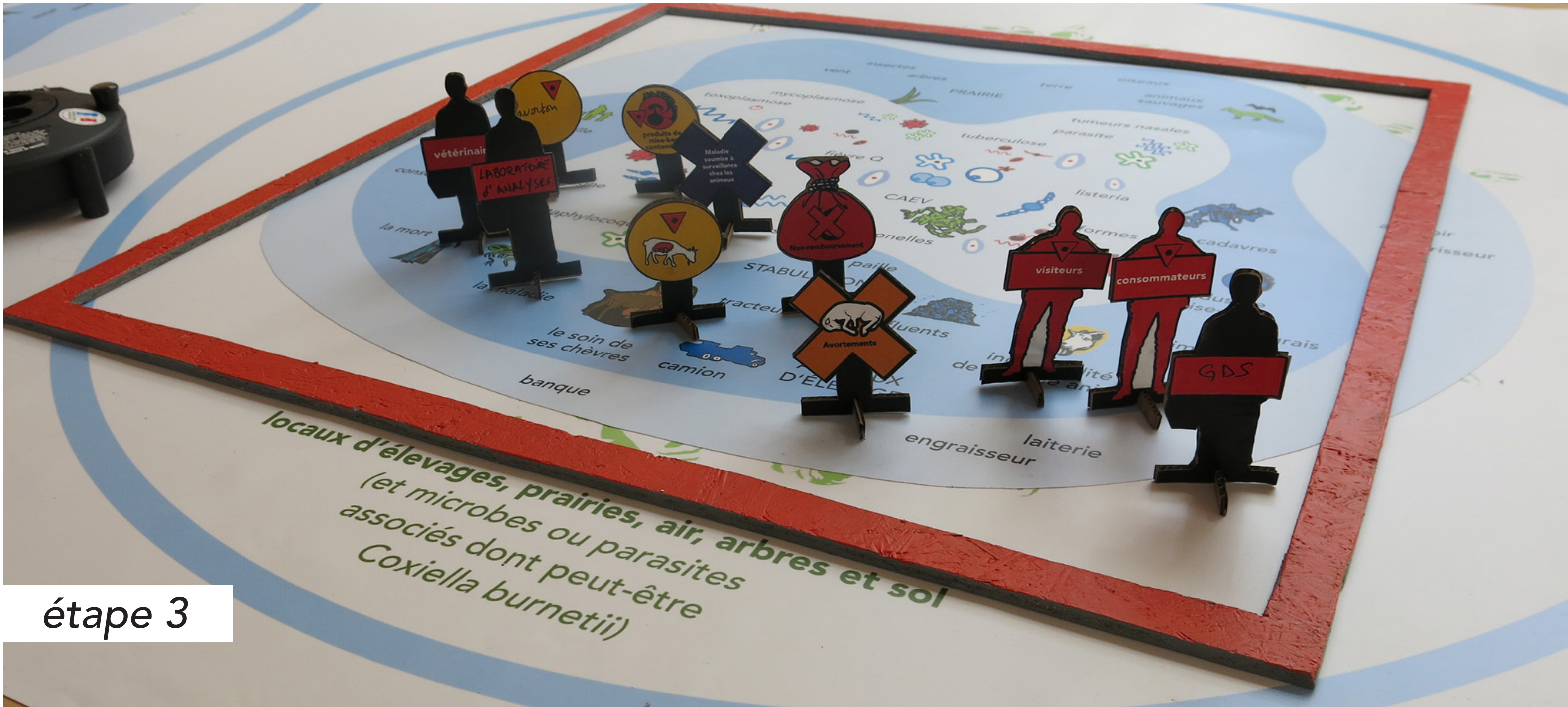

étape 3

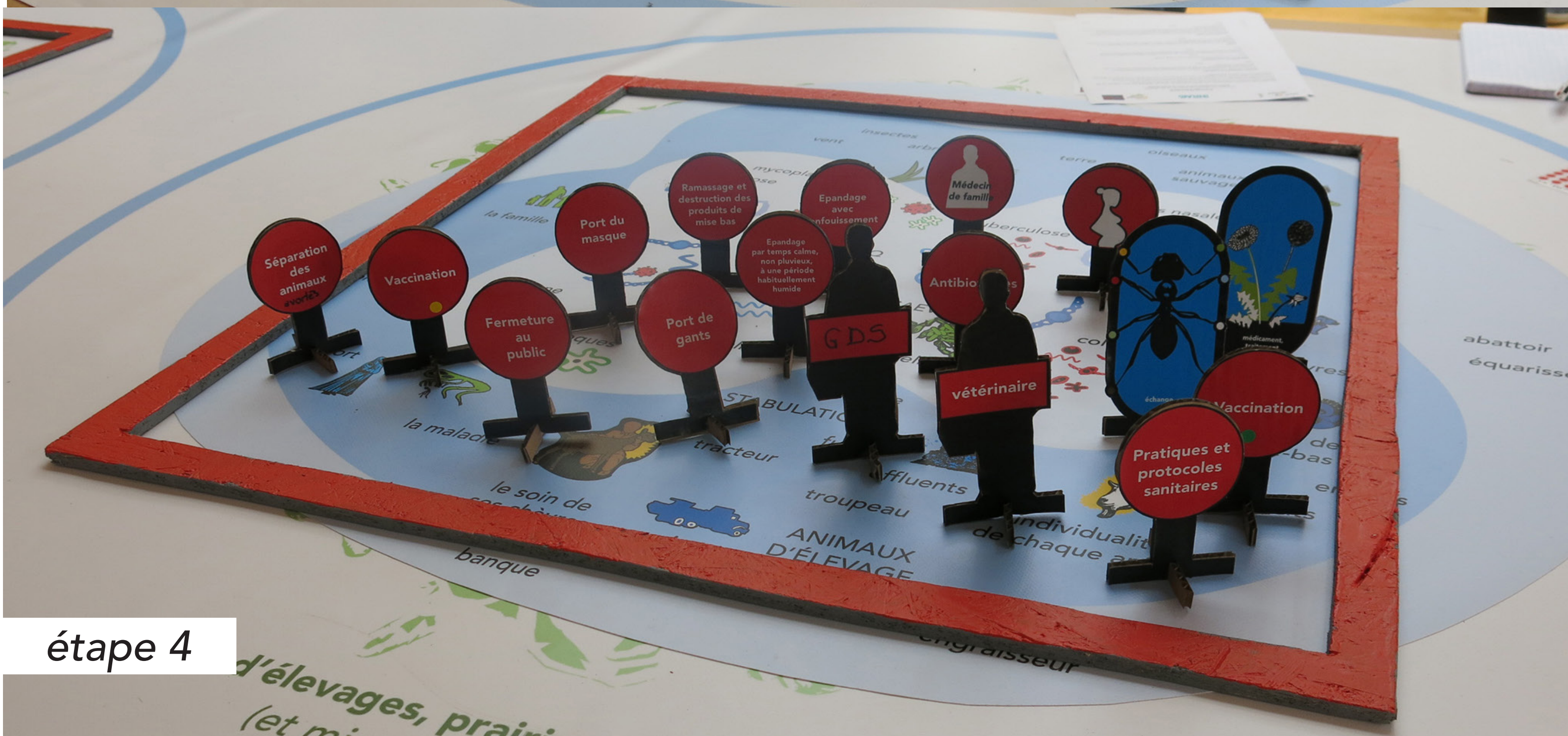

étape 4

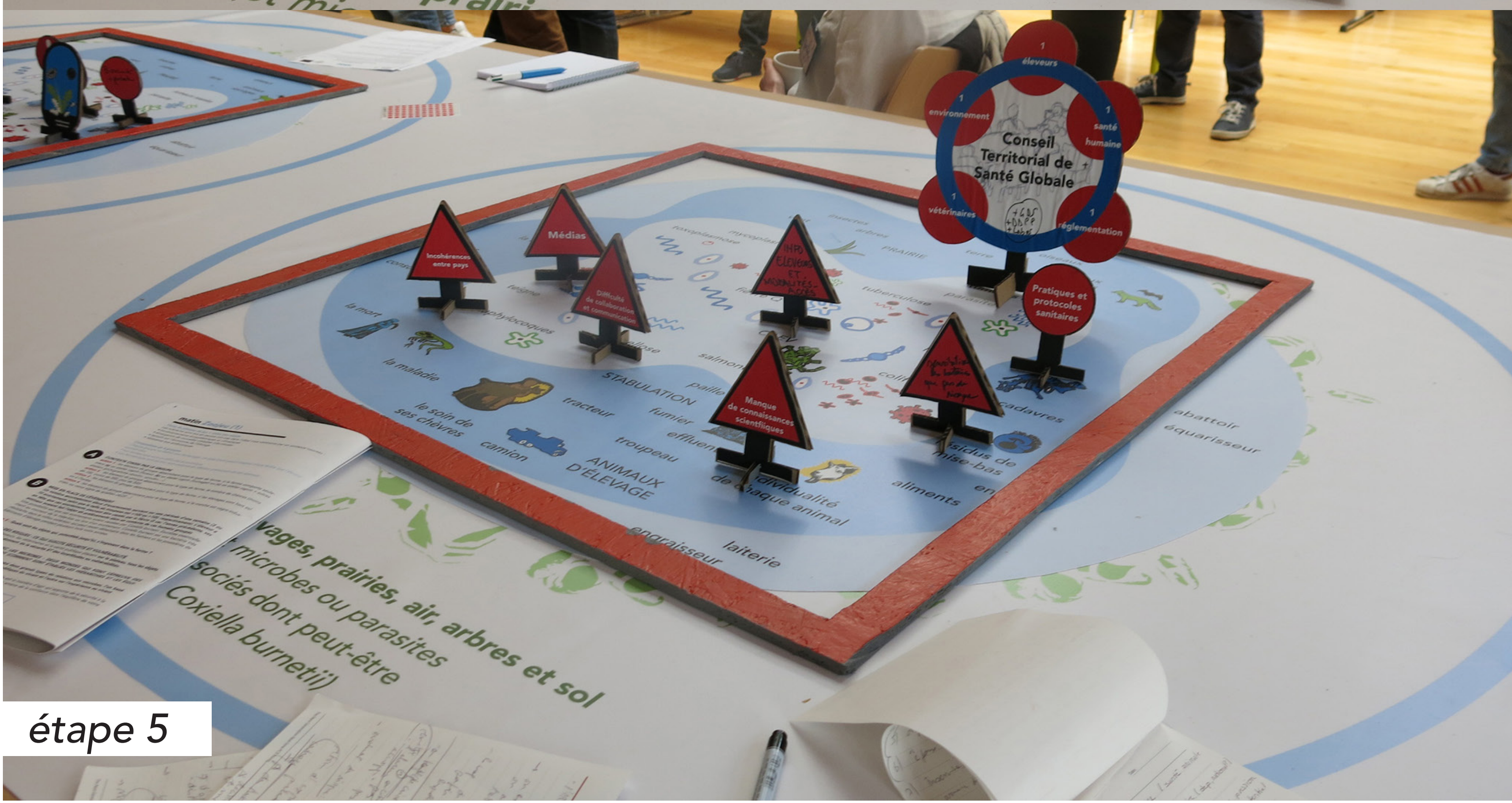

étape 5

les restitutions des jeux du matin

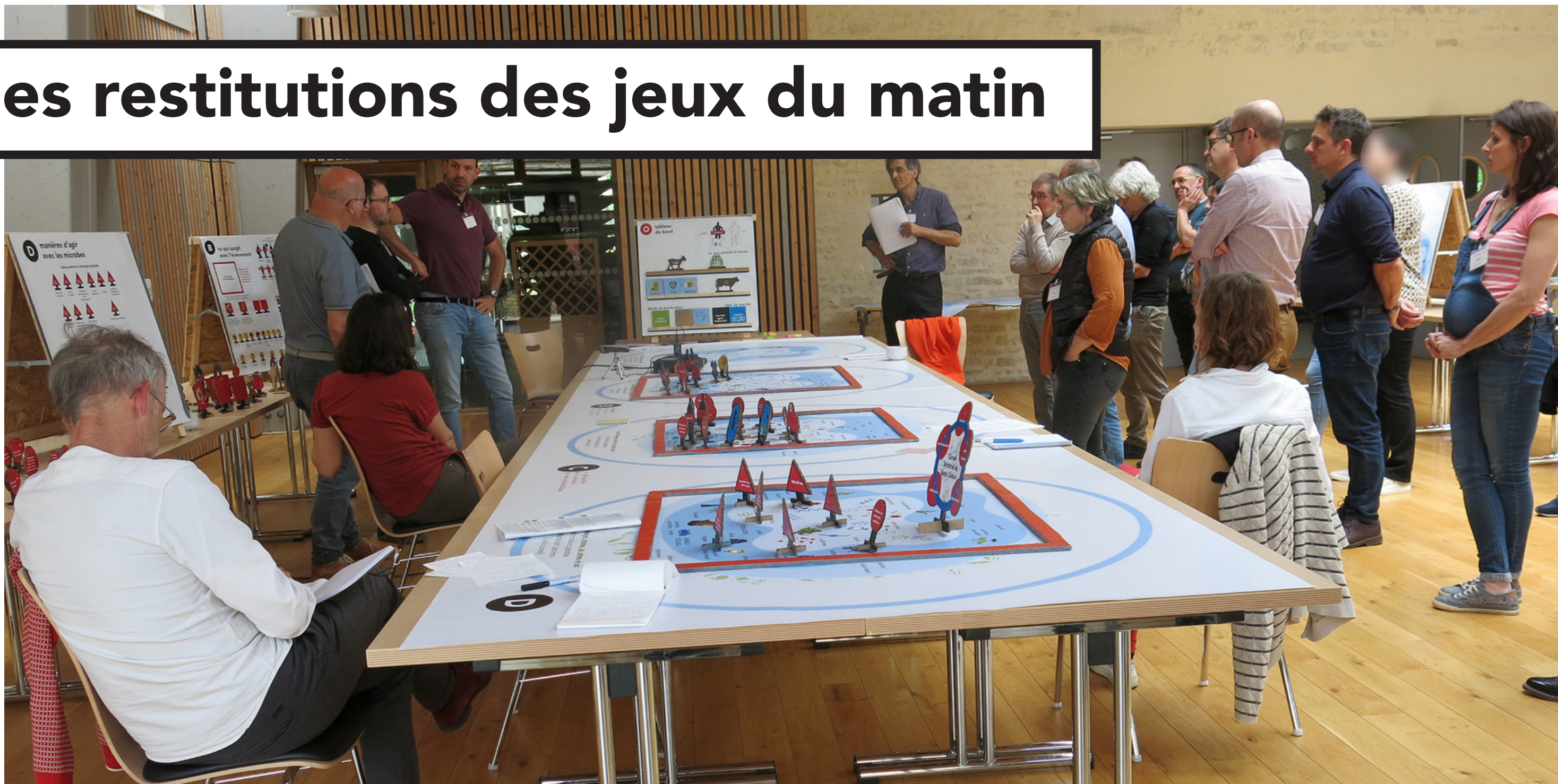

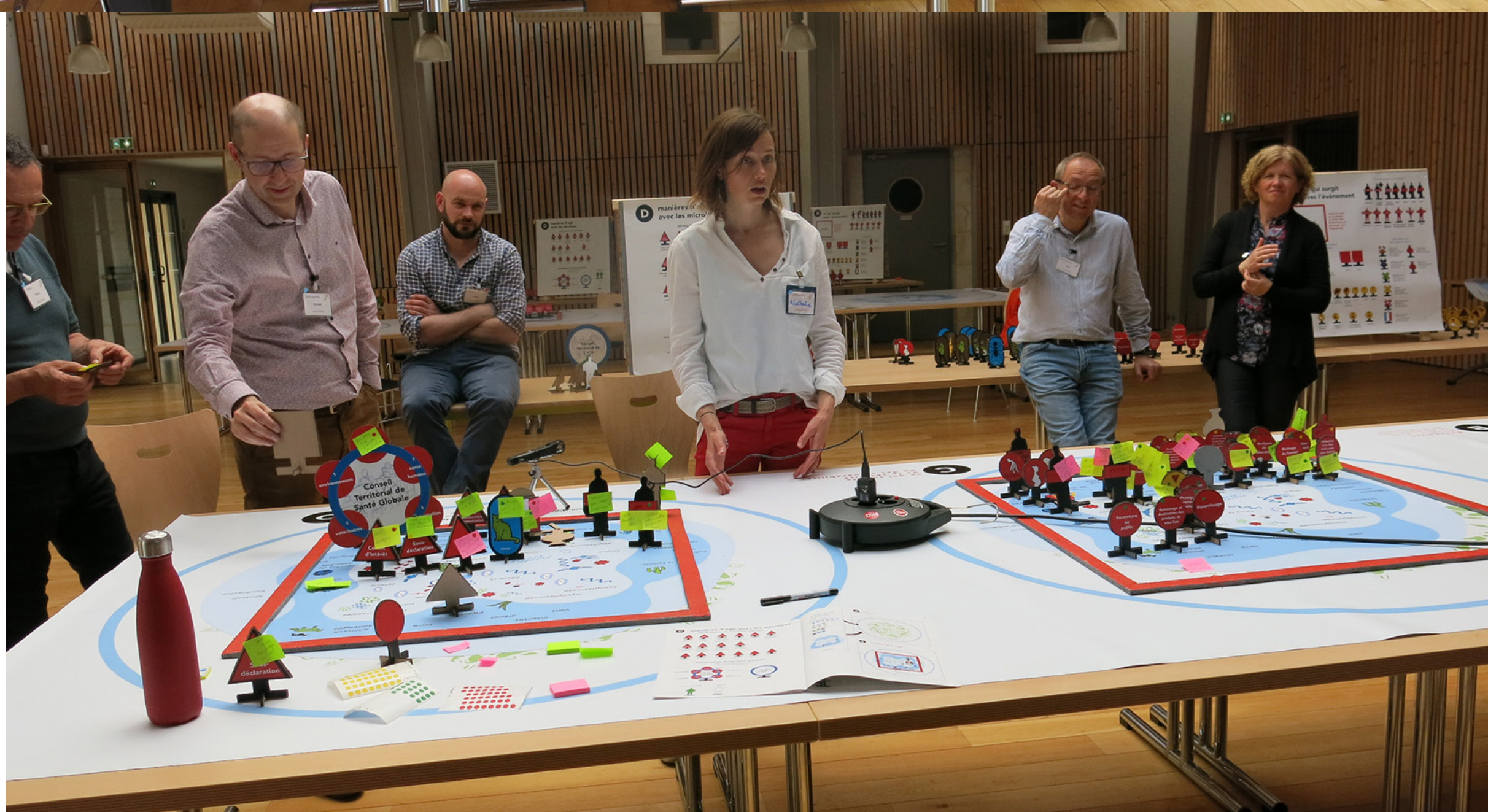

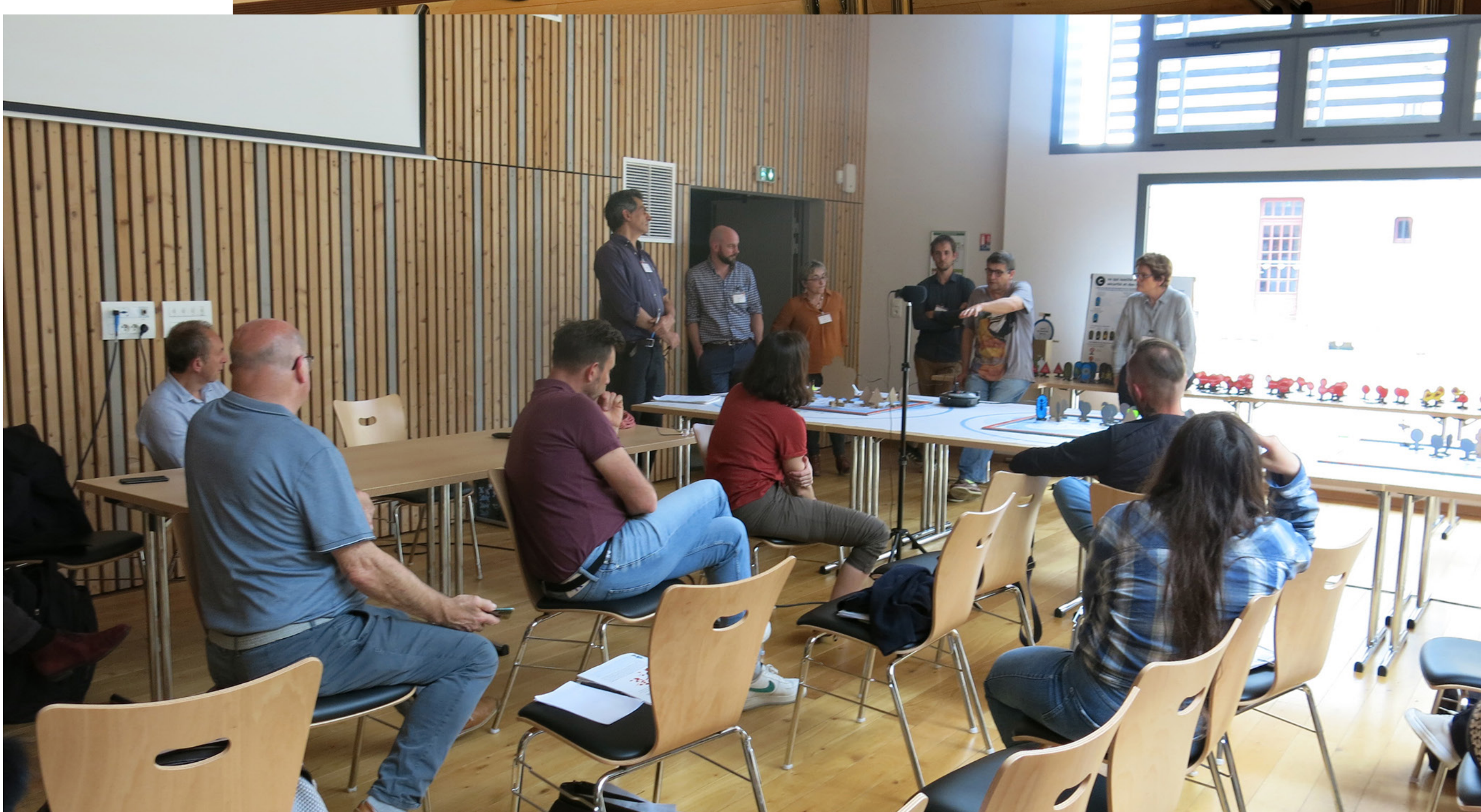

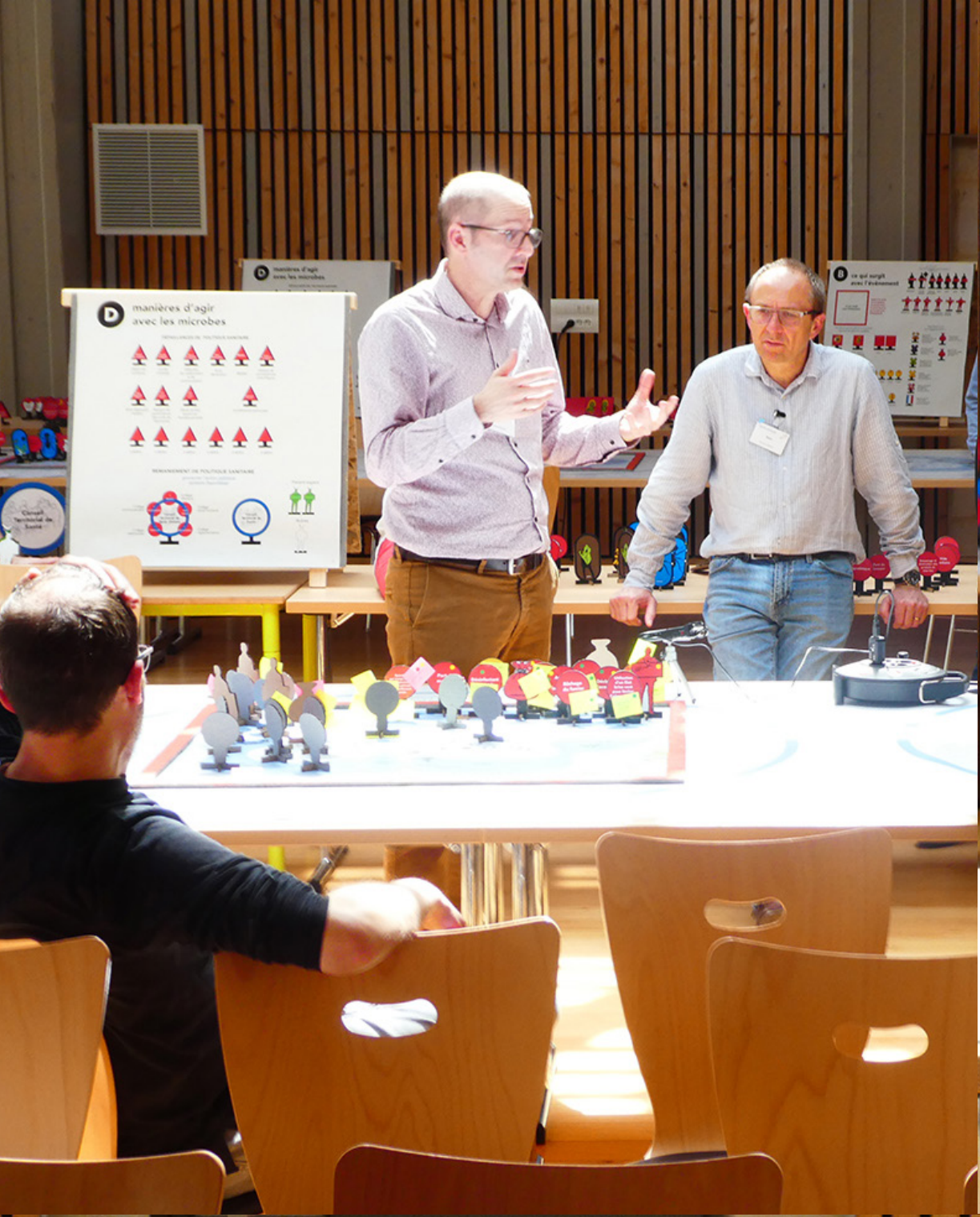
manières d'agir
avec les microbes
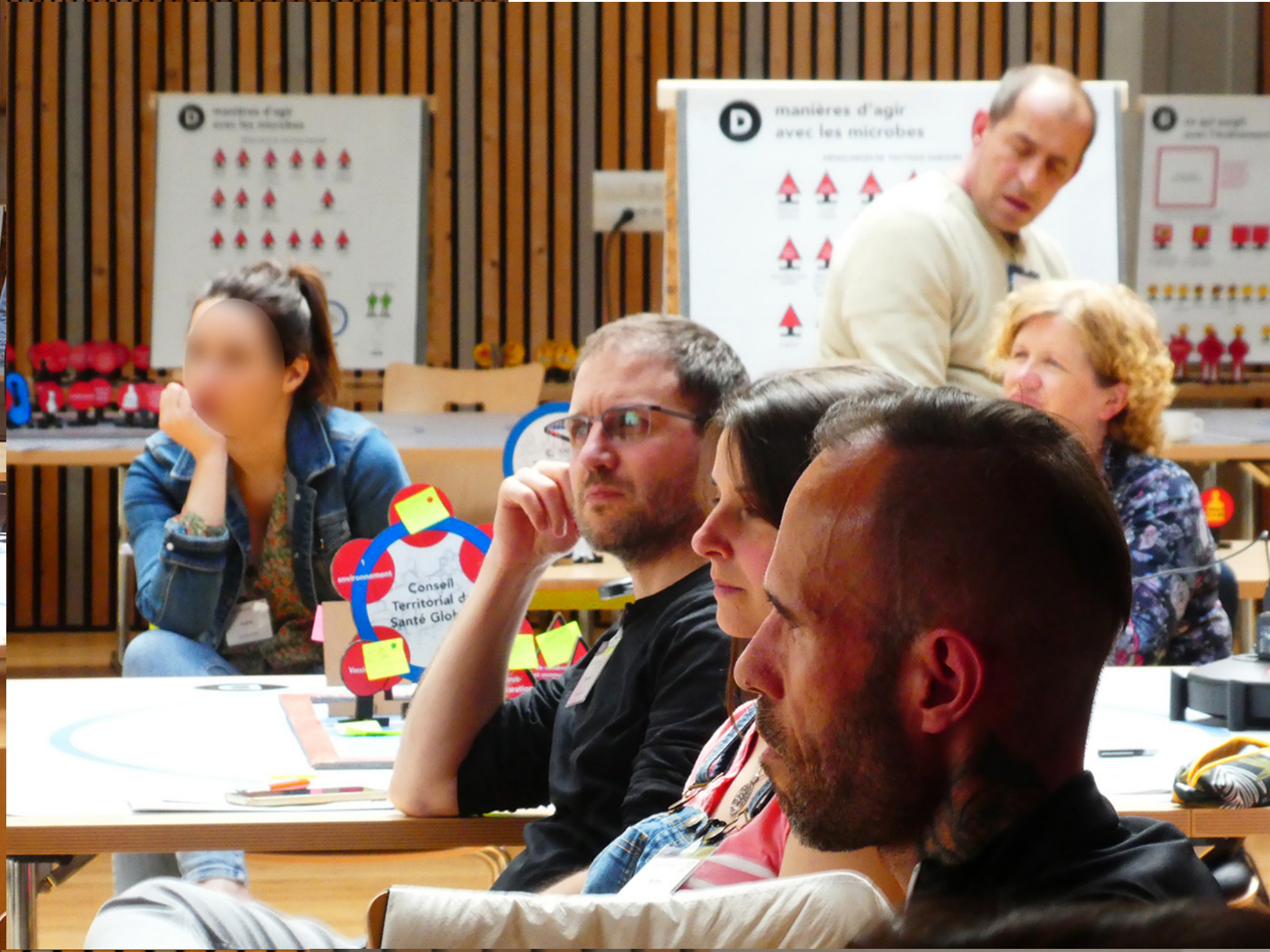
manières d'agir
avec les microbes
Conseil
Territorial d
Santé Glob
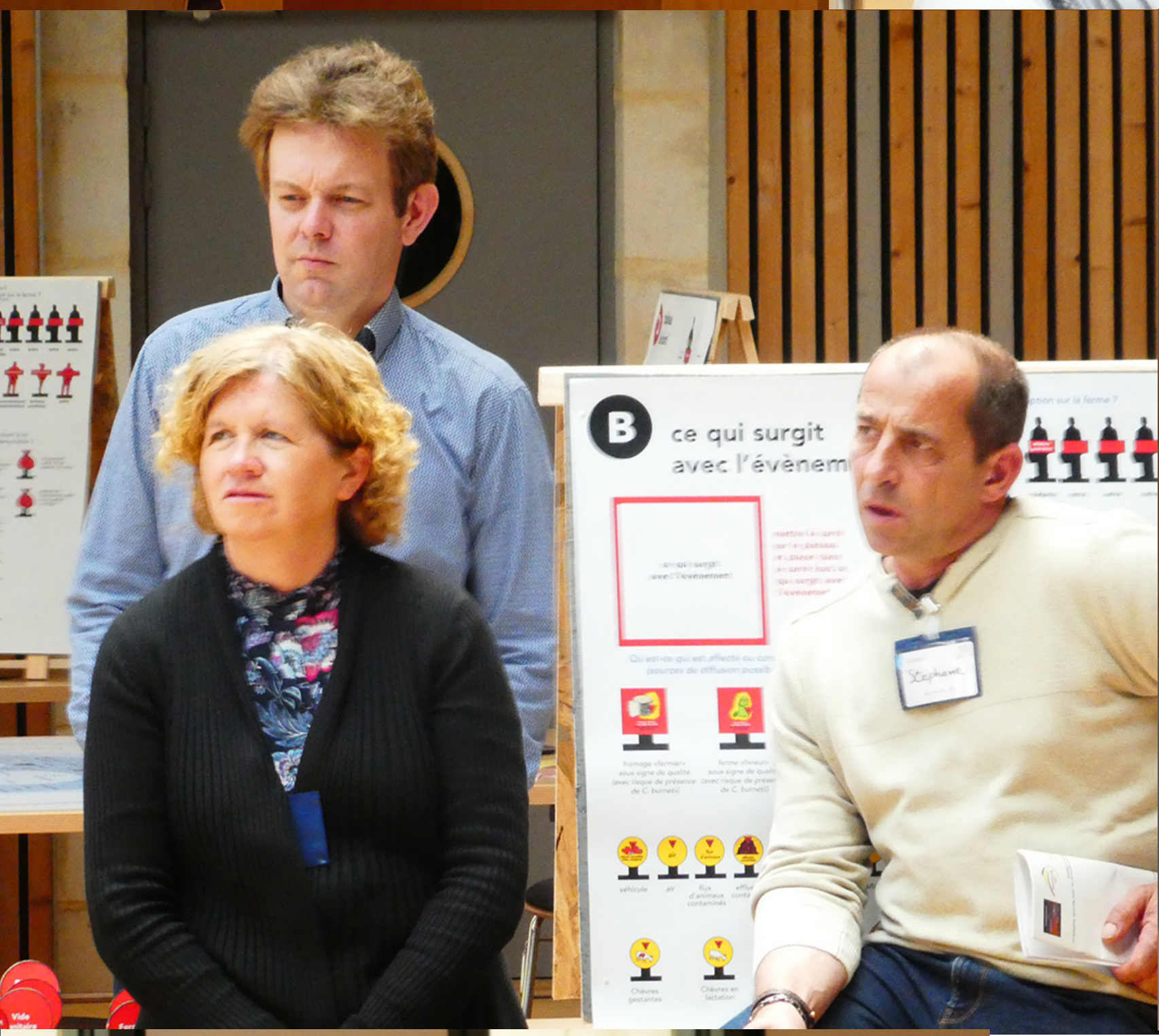
ce qui surgit
avec l'évènem
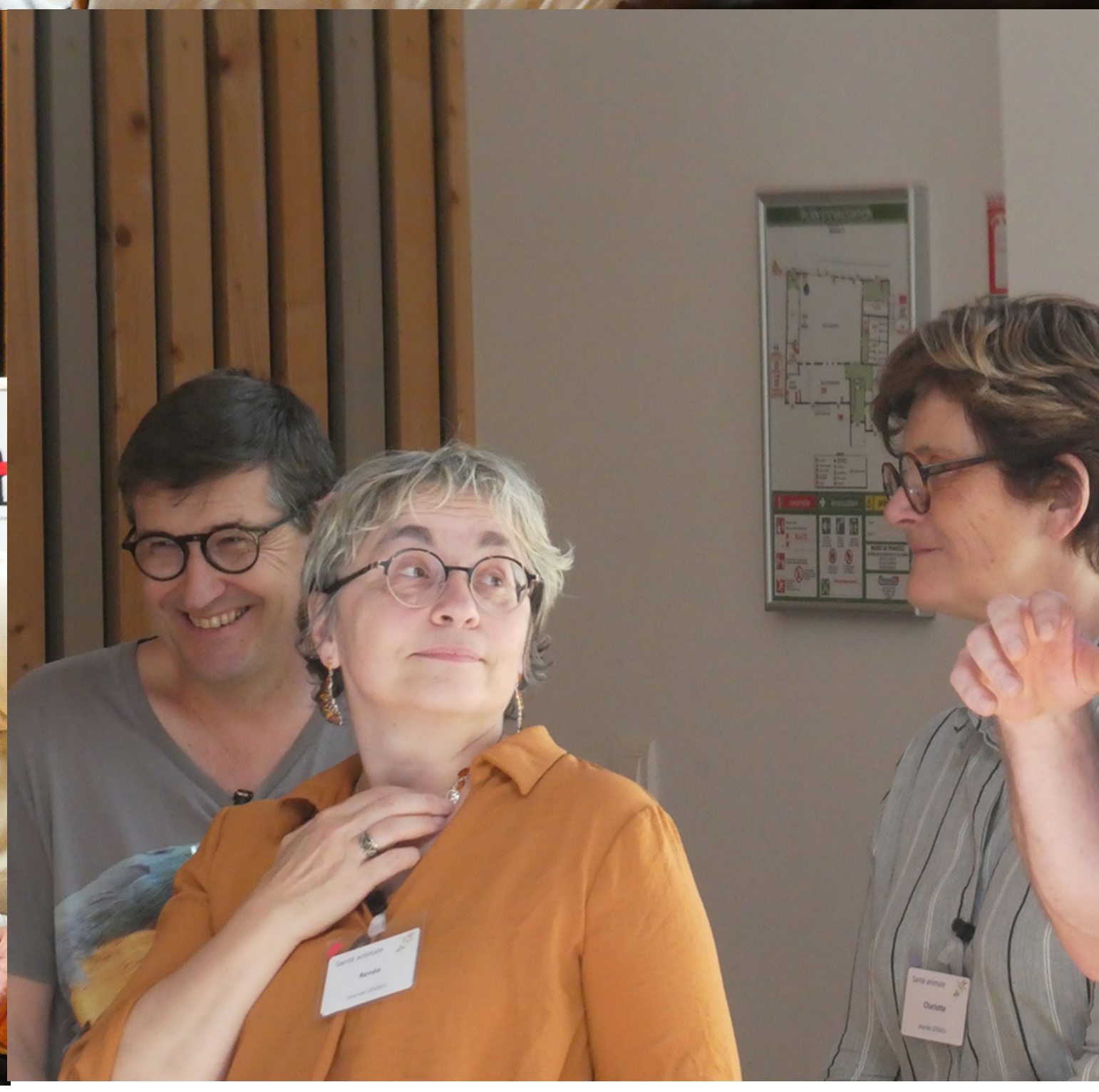
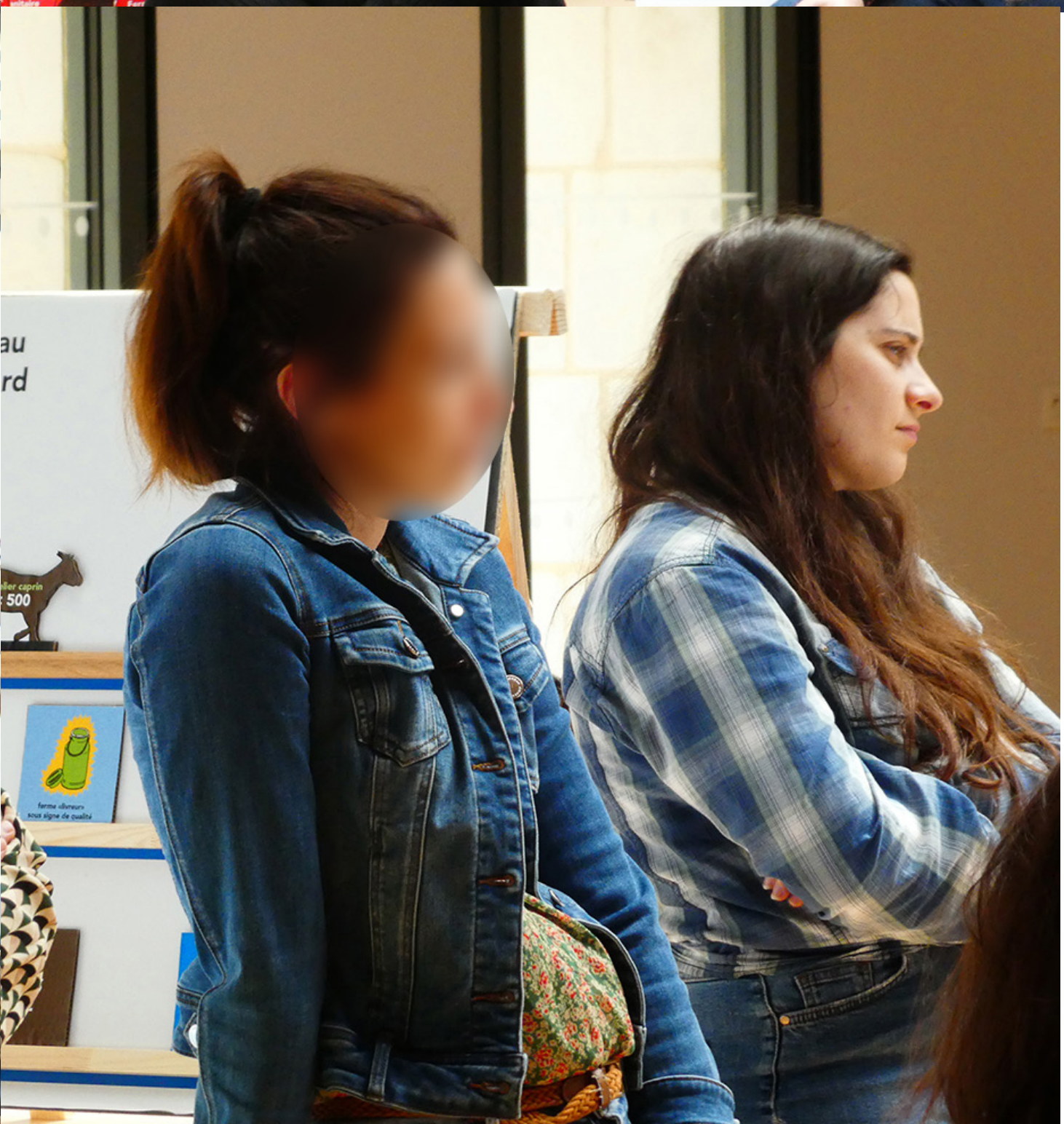

l'après-midi

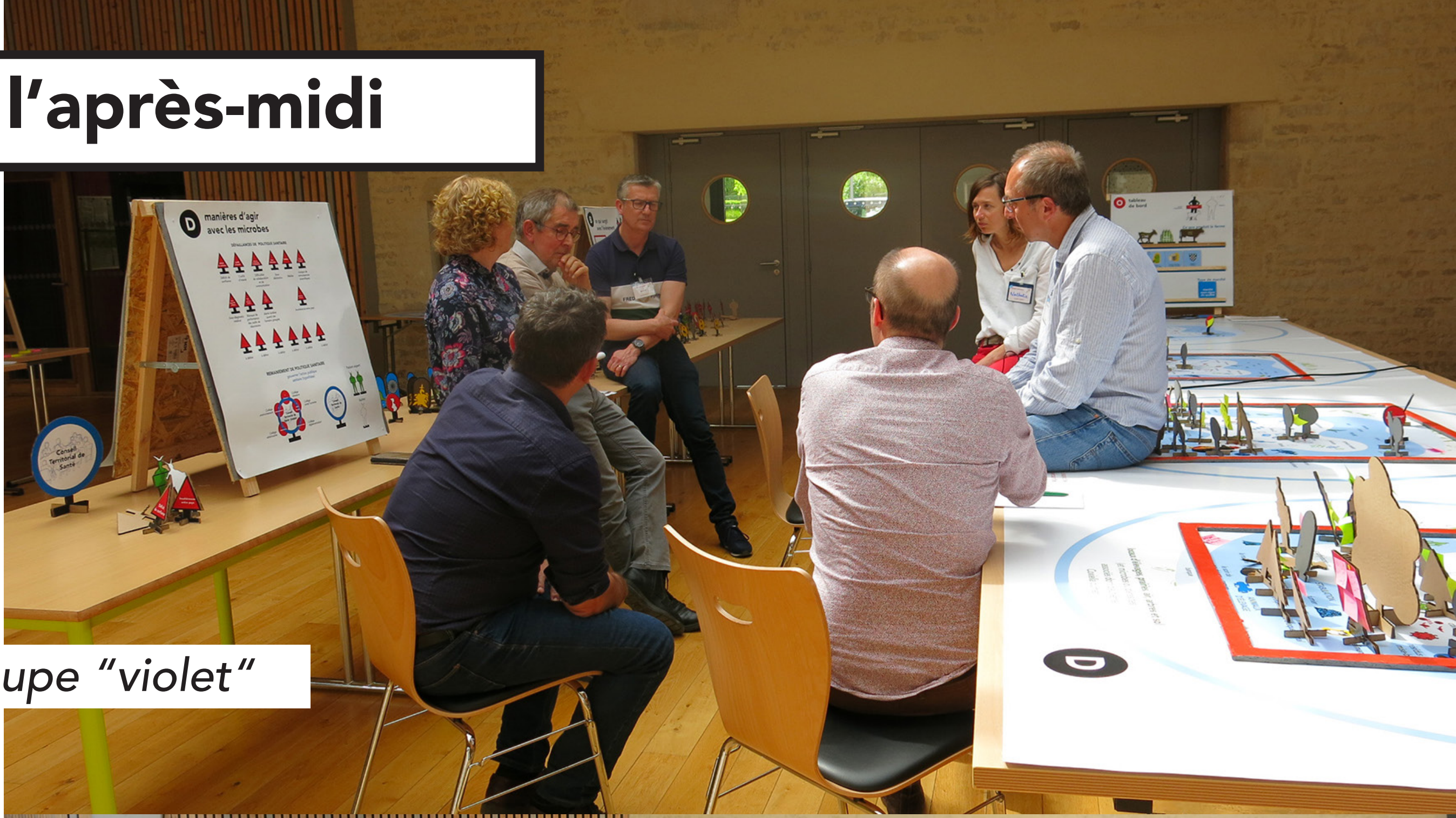

groupe "violet"

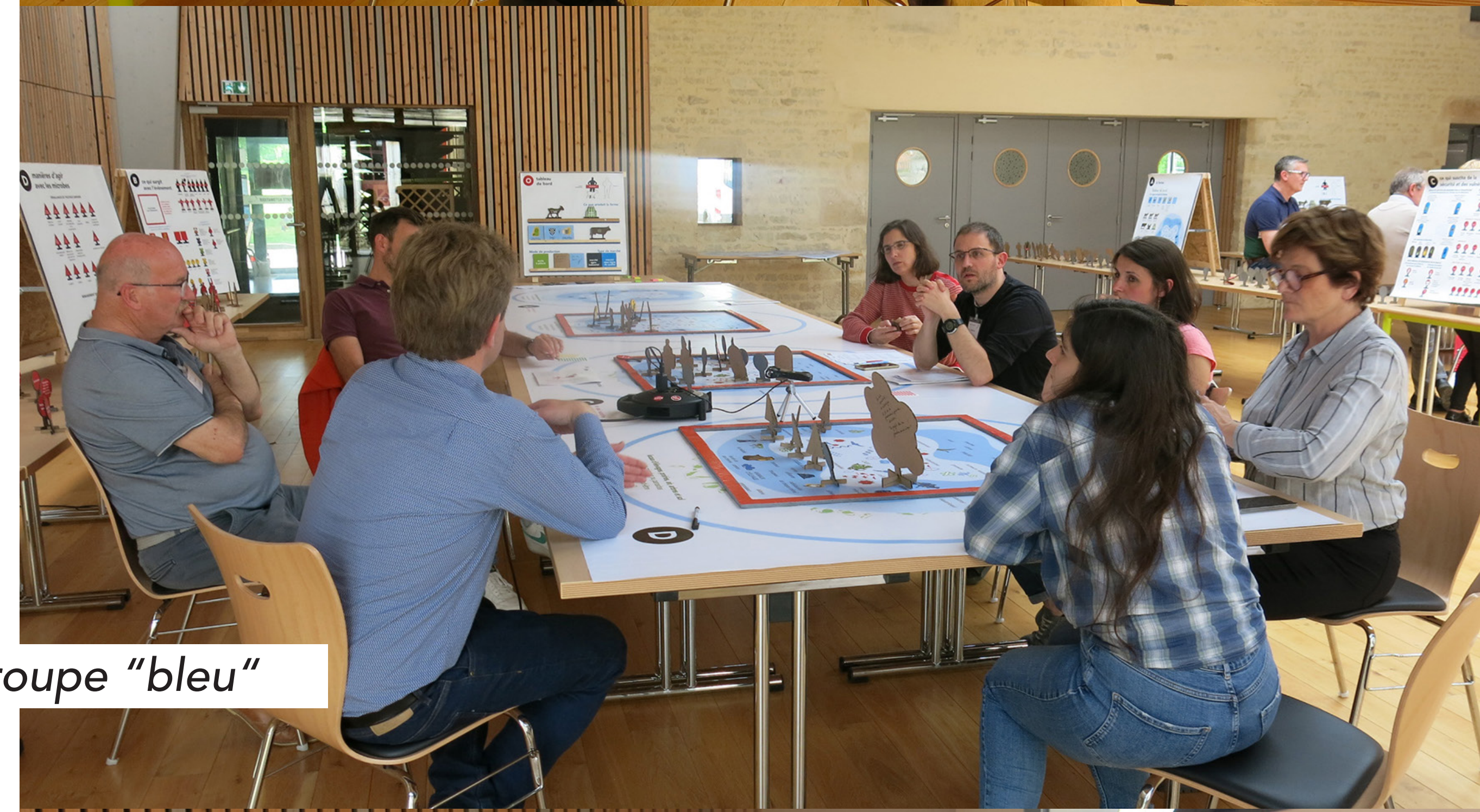

groupe "bleu"

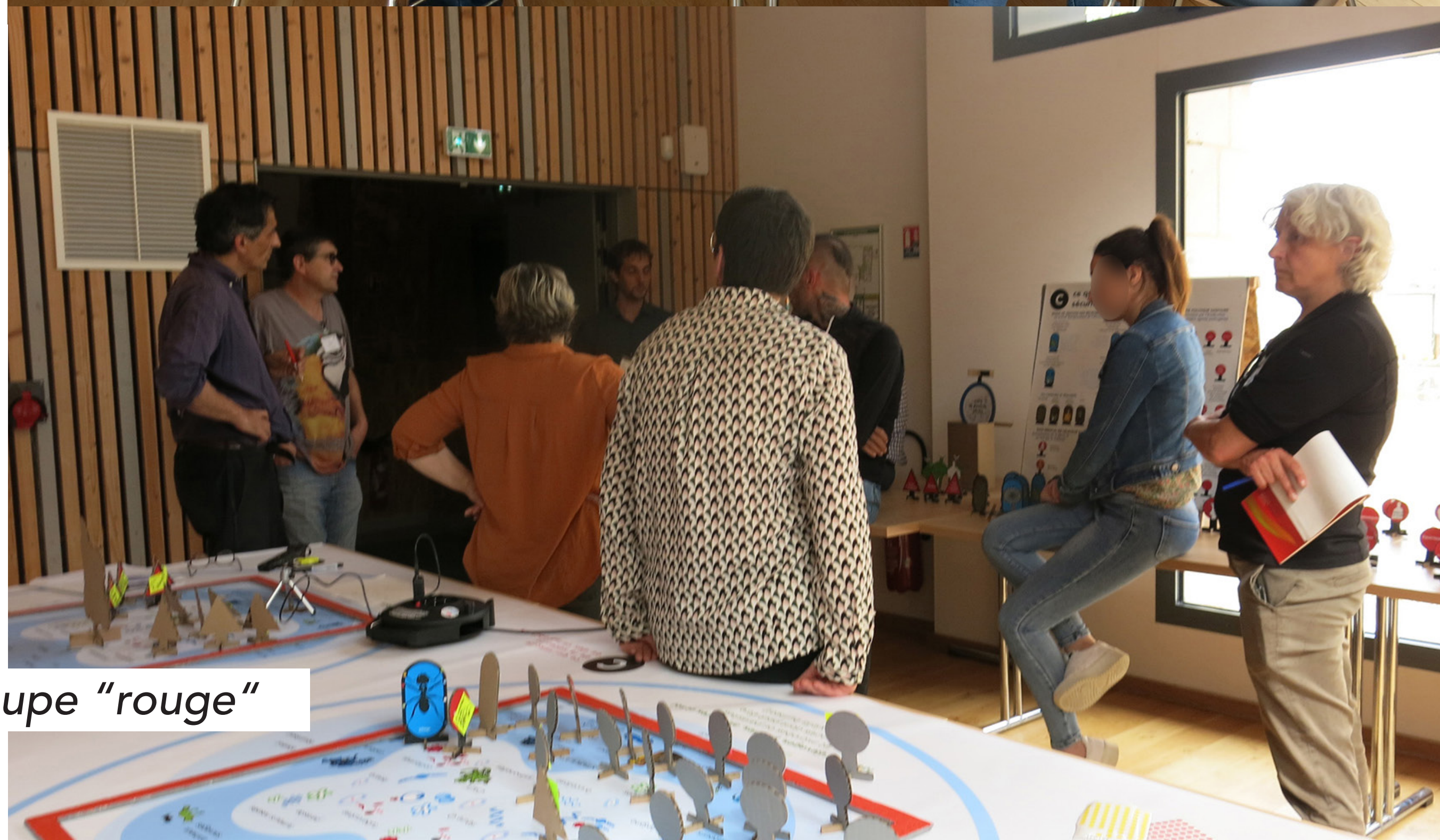

groupe "rouge"

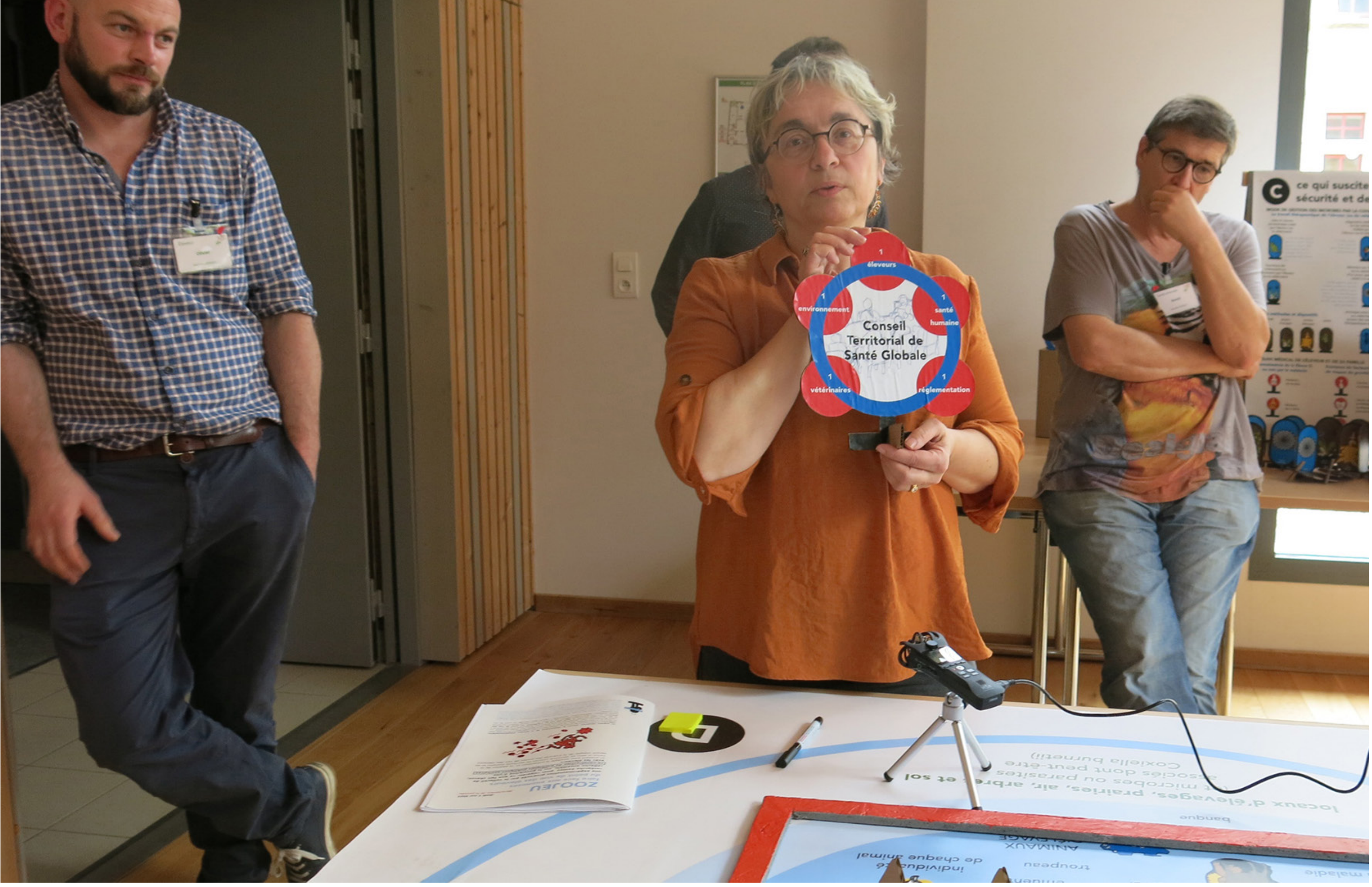
éleveurs
environnement
santé humaine
Conseil Territorial de Santé Globale
vétérinaires
réglementation
ce qui suscite
sécurité et de
ZOOJEU
banque
troupeau
maladie

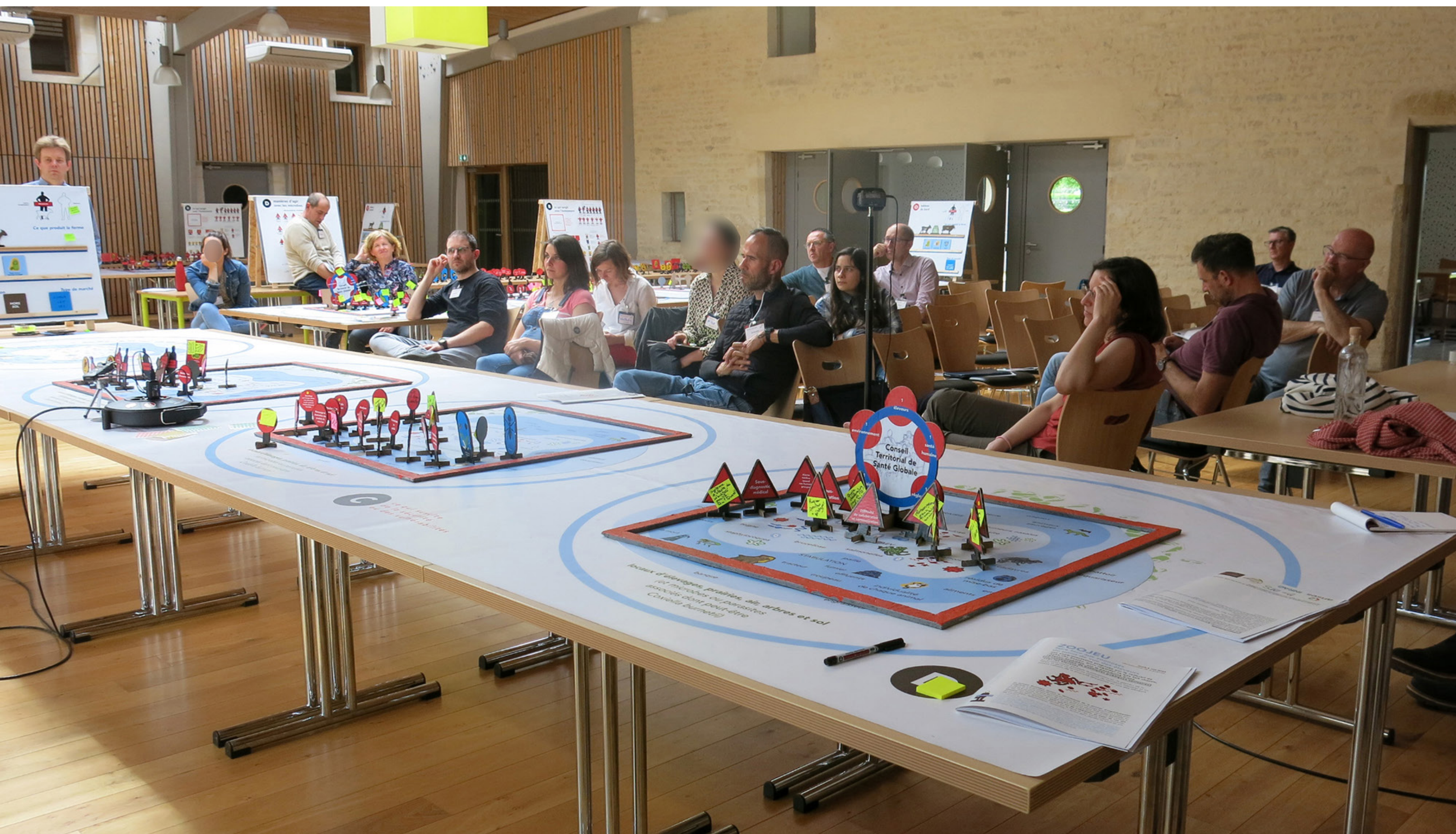
Conseil Territorial de Santé Globale
ZOOJEU

l'après-midi

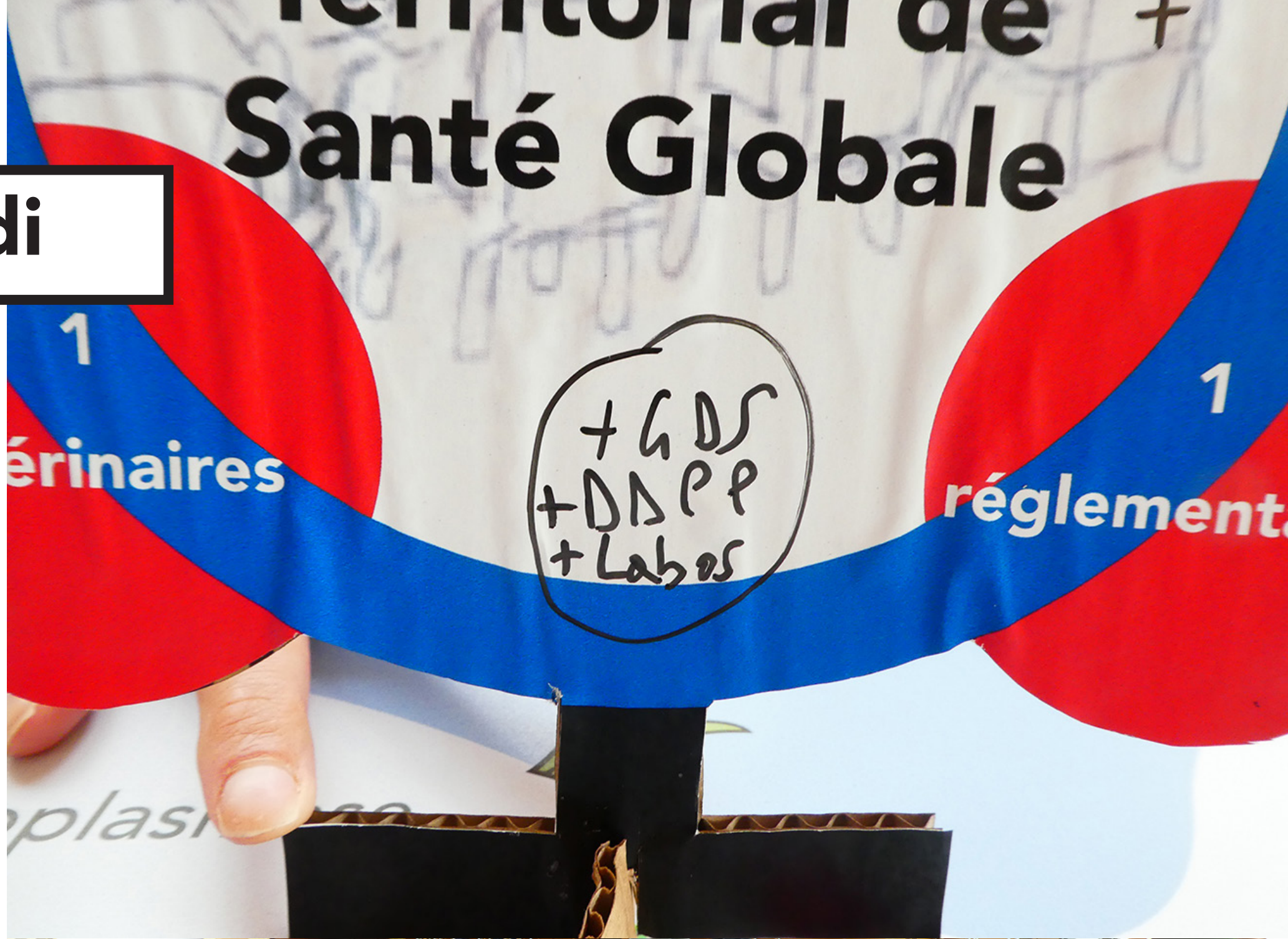
Santé Globale
+GDS
+DDPP
+Labos

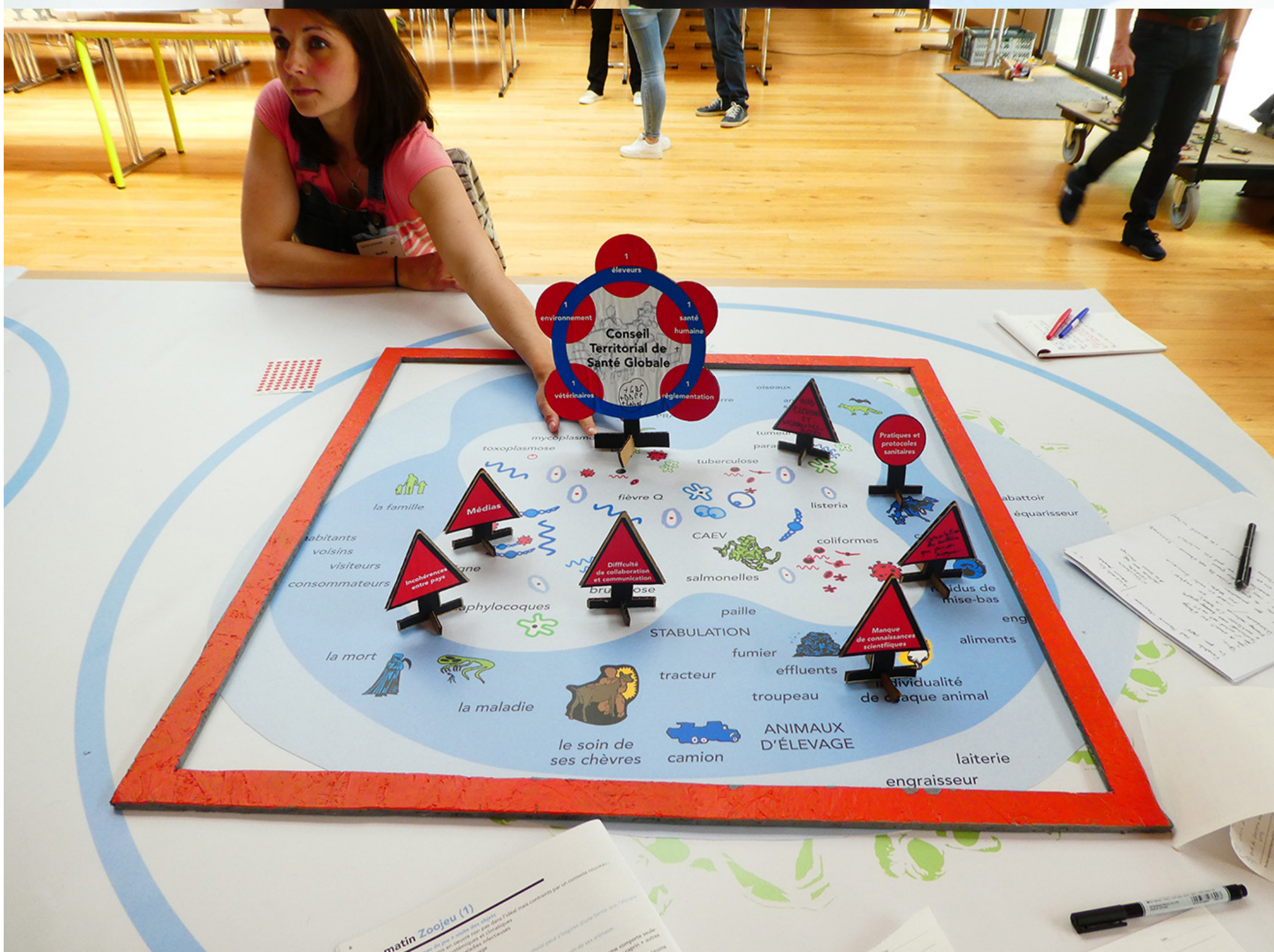
Conseil Territorial de Santé Globale
Médias
toxoplasmose
tuberculose
la famille
fièvre Q
listeria
CAEV
coliformes
salmonelles
paille
STABULATION
fumier
effluents
tracteur
troupeau
la mort
la maladie
le soin de ses chèvres
camion
ANIMAUX D'ÉLEVAGE
aliments
abattoir
équarisseur
laiterie
engraisseur

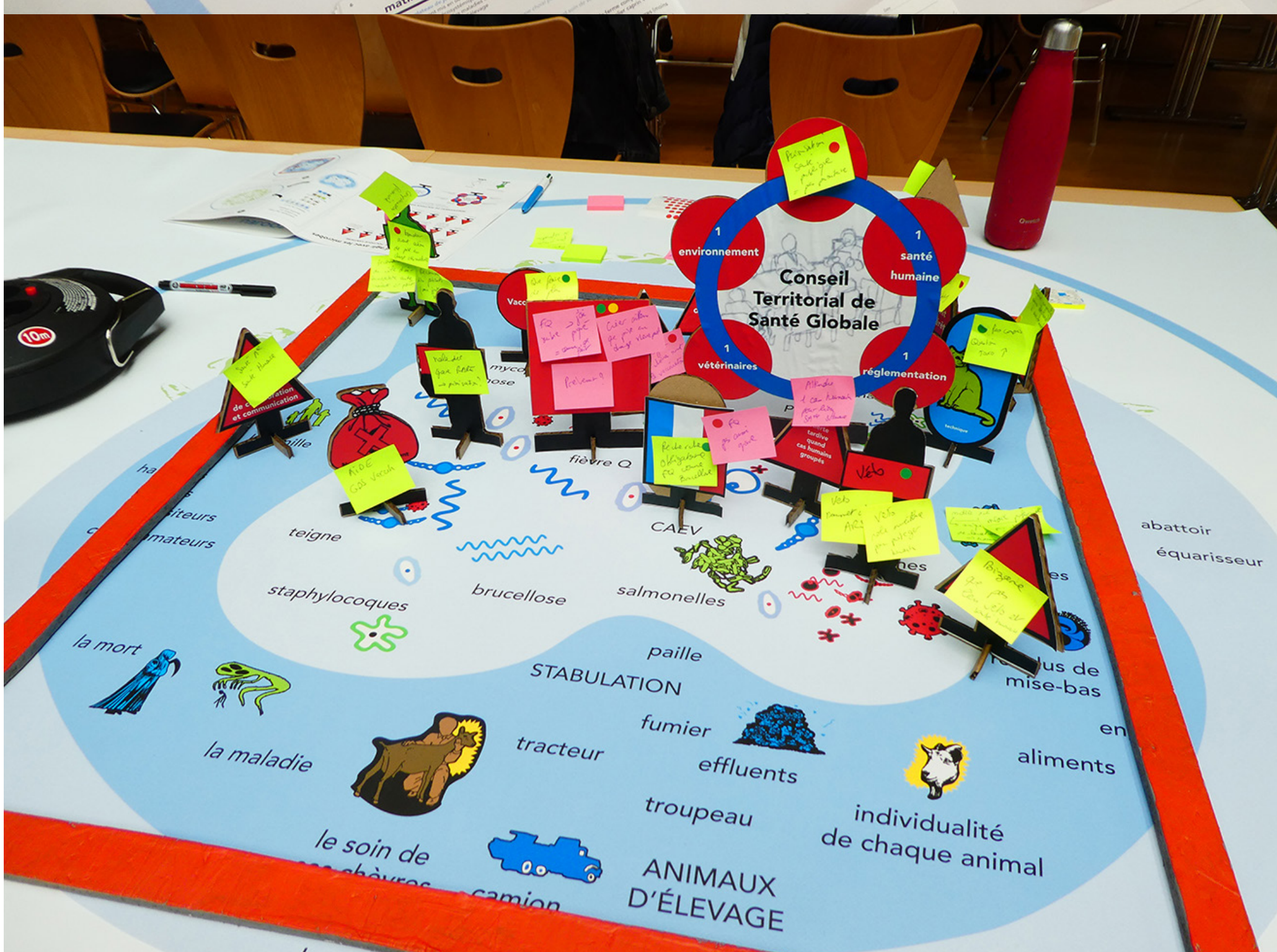
Conseil Territorial de Santé Globale
environnement
santé humaine
vétérinaires
réglementation
fièvre Q
CAEV
teigne
brucellose
salmonelles
staphylocoques
la mort
la maladie
paille
STABULATION
fumier
tracteur
effluents
troupeau
individualité de chaque animal
aliments
abattoir
équarisseur
ANIMAUX D'ÉLEVAGE

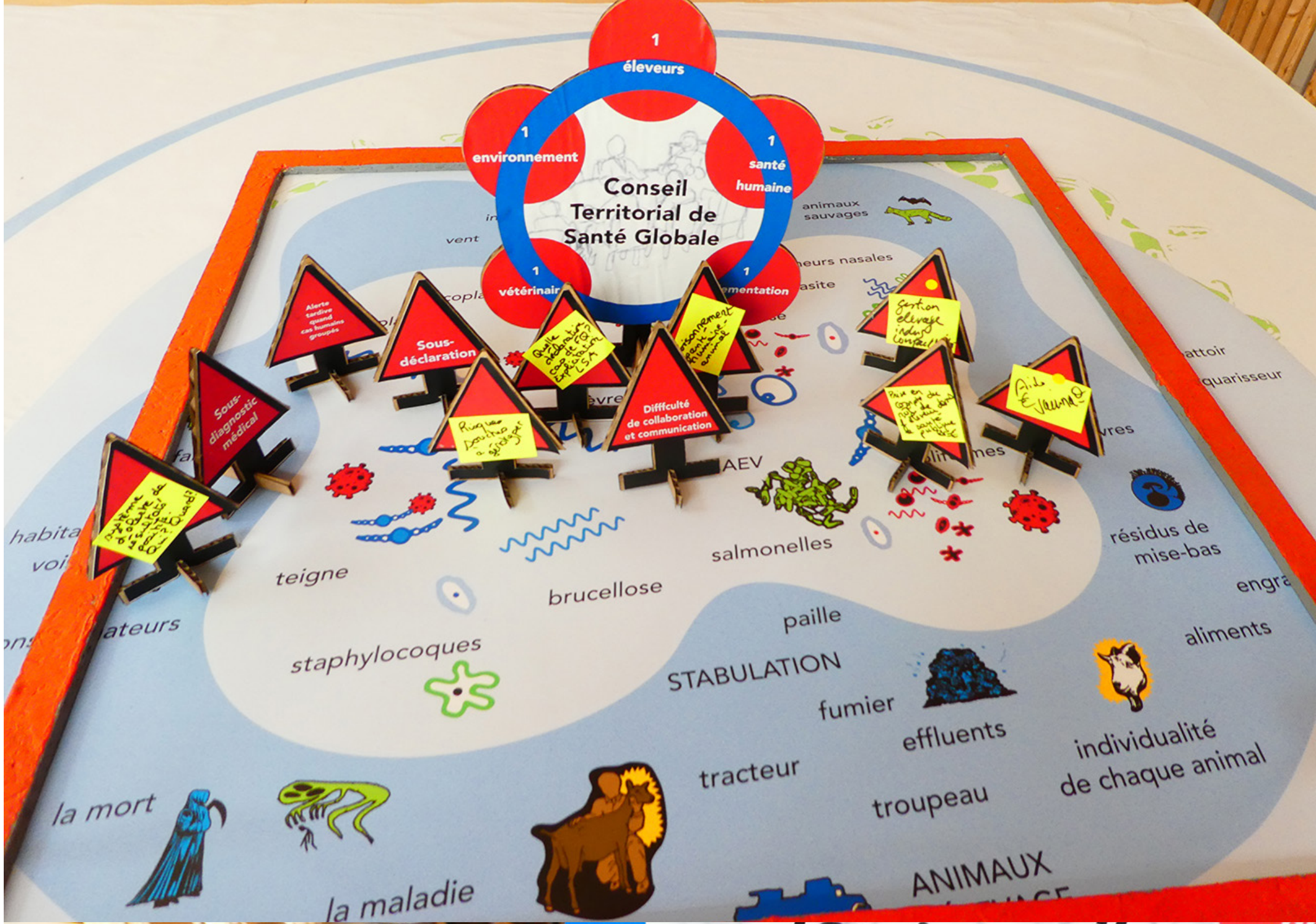
éleveurs
environnement
santé humaine
Conseil Territorial de Santé Globale
Sous-déclaration
Sous-diagnostic médical
Difffculté de collaboration et communication
animaux sauvages
vent
AEV
teigne
brucellose
salmonelles
staphylocoques
paille
STABULATION
fumier
effluents
tracteur
troupeau
aliments
résidus de mise-bas
individualité de chaque animal
la mort
la maladie
ANIMAUX
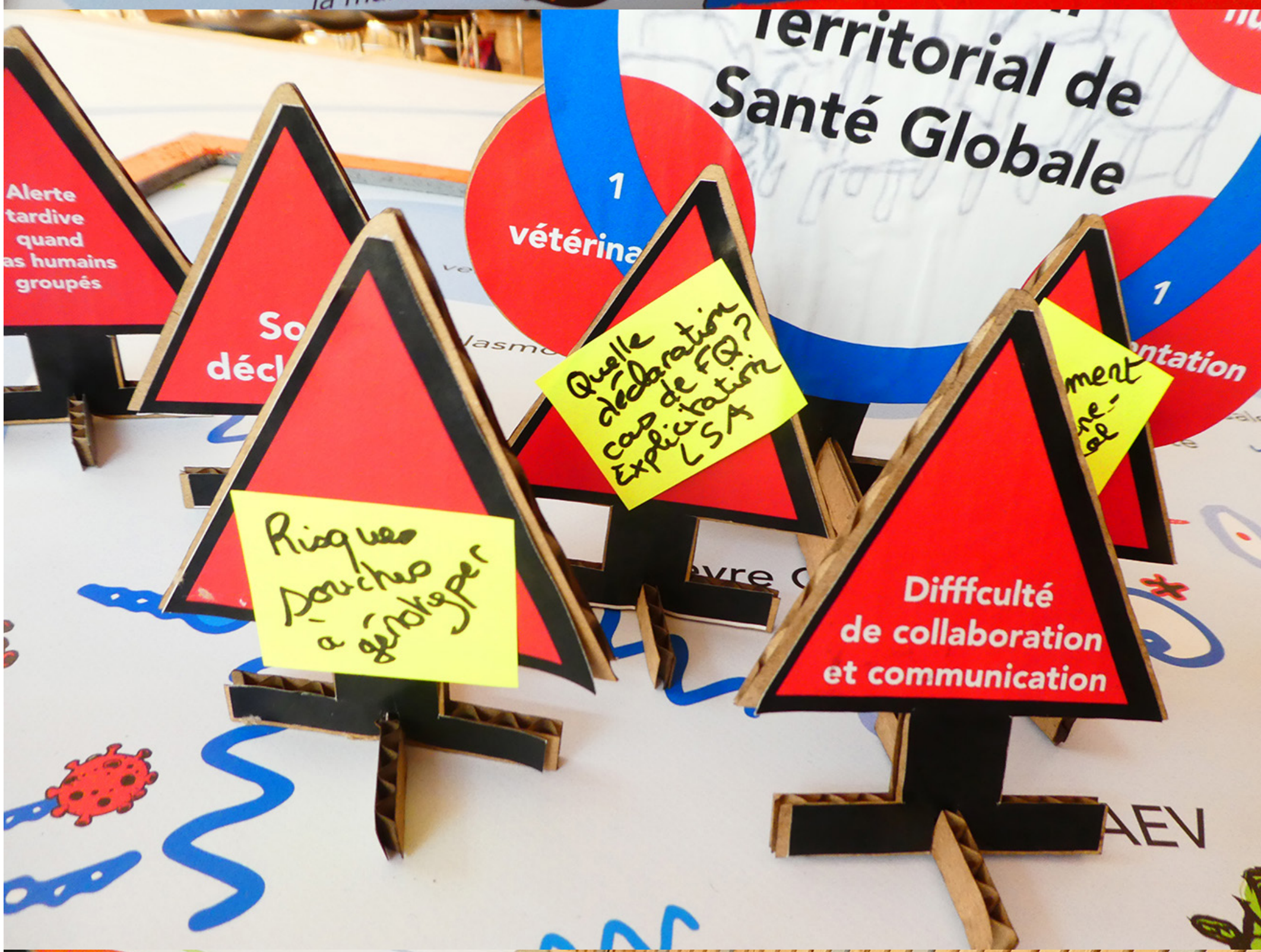
Territorial de Santé Globale
Alerte tardive quand
groupés
Risques souches à génotyper
Quelle déclaration cas de FQ? Explicitation LSA
Difffculté de collaboration et communication
AEV
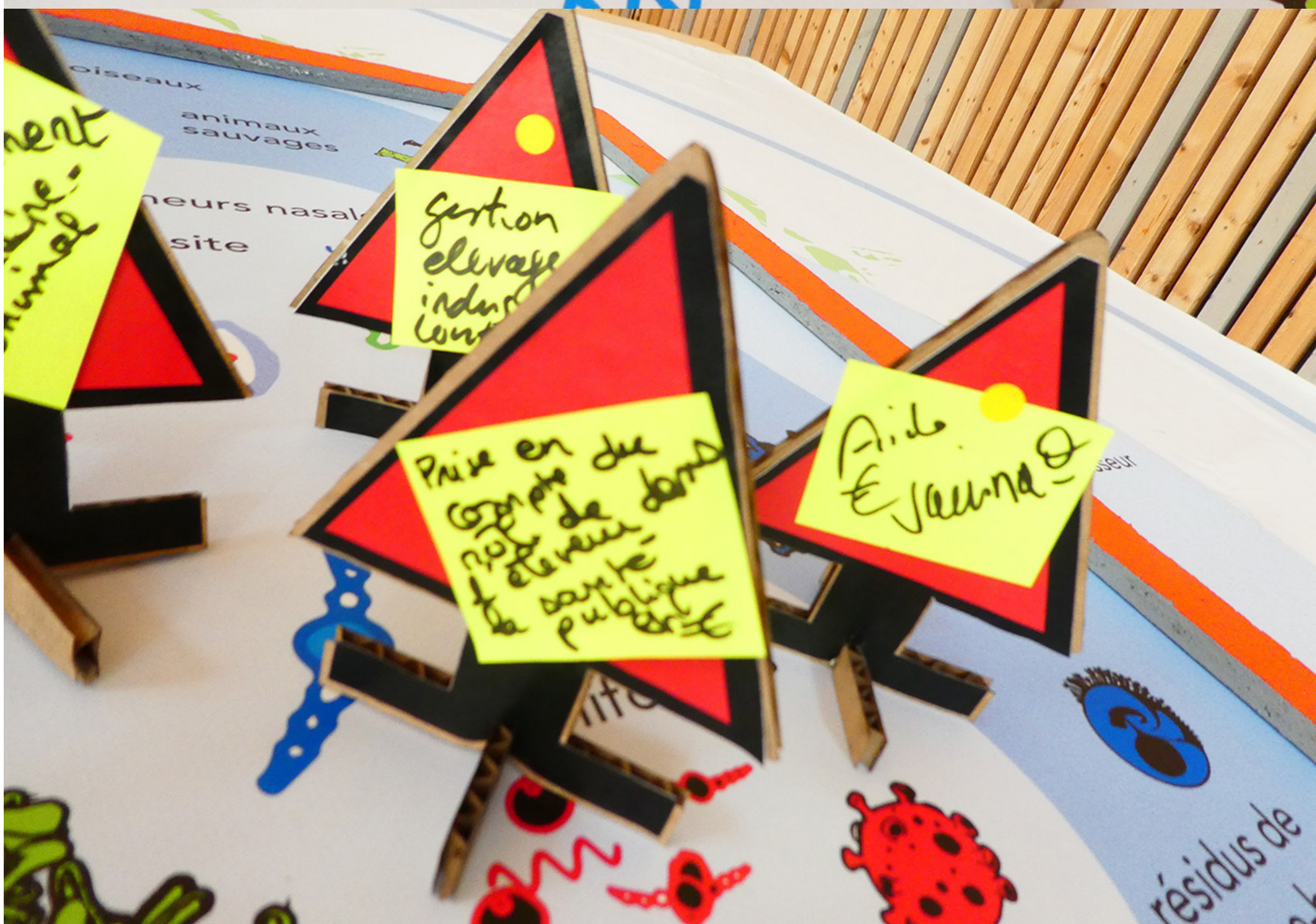
oiseaux
animaux sauvages
résidus de

quelques commentaires

Échanges intéressants
et constructifs

Oui c'est très intéressant de parler avec
des gens qui ne sont pas du milieu agricole pour
leur exposer nos problèmes

Belle rencontre. Échanges fructueux. Profil éleveur
pertinent

Importance de la présence concomitante et non hiérarchique
des différents acteurs d'une zoonose, avec écoute bienveillante
et partage / travail en commun.

J'apprécie tout particulièrement aussi le croisement des
disciplines biologie - anthropologie - représentation graphique
→ richesse +++

Bravo pour le travail réalisé !

… et la suite ? Quelques unes de vos propositions à chaud

propositions données à l'issue de la journée du 4 mai 2023

Un groupe de travail ou d'échange réunissant tous les acteurs.

Réseau avec la santé humaine.

Concrétiser les échanges par la mise en place d'un réseau local (territorial)

La mise en place des conseils territoriaux

- Garder le contact avec les acteurs pour expérimenter le conseil de santé globale

Un groupe de travail ou d'échange réunissant tous les acteurs.

Tester le jeu dans d'autres départements.

Il faut passer à l'action, et donc créer un dispositif permettant d'améliorer la prévention et la gestion des cas de fièvre Q en élevage.

… ce que nous vous proposons

Nous avons obtenu auprès du métaprogramme INRAE Sanba (https://sanba.hub.inrae.fr/) un financement de deux ans pour prolonger et valoriser ZOOJEU dans le cadre d'un projet exploratoire appelé **« Transition ZOOJEU ».**

ZOOJEU partait d'un exemple de zoonose, la fièvre Q, point de départ à la fois des enquêtes anthropologiques et de la journée d'expérimentation collective du jeudi 4 mai 2023. Dans ces deux contextes, nous avons assisté à un déplacement de l'objet d'attention et des ambitions du projet, **passant de l'action publique sanitaire pour faire face à une zoonose, à l'action publique de « santé globale ».** Ainsi, alors que la question de la santé est généralement abordée par les institutions sanitaires sous l'angle des maladies, des agents pathogènes et des risques, nous avons fait que le constat dans nos travaux que les éleveurs parlent aussi et surtout de « santé ».

De façon intéressante, ce positionnement fait écho au concept de **"tournant microbien"** décrit notamment par la chercheuse Charlotte Brives (1) : le XXIème siècle marque un glissement du savoir sur les « microbes », de la notion de risque à celle de santé, partant du constat que la bonne santé et le bien-être des êtres humains, des animaux et des écosystèmes tient beaucoup à celle des microbiotes avec lesquels ils cohabitent en permanence et desquels ils dépendent (2). Les microbes sont donc nécessaires à la santé et au bien-être tout en étant parfois pathogènes et donc nuisibles à la santé et au bien-être.

Cette **ambivalence entre « menaces & risques »** et **« santé & bien être »** donne lieu à des points de vue variés, qui dépendent des expériences de chacun. En d'autres termes, nous n'éprouvons pas les mêmes choses et ne comprenons pas tout à fait de la même manière les microbes selon que nous sommes éleveur, acteur de la réglementation ou chercheur.

Dans **« Transition ZOOJEU »**, nous proposons de travailler collectivement autour de cette ambivalence des microbes entre « menaces & risques » et « santé & bien-être » et d'expérimenter comment elle pourrait être prise en considération, par exemple dans le cadre d'un Conseil Territorial de Santé Globale.

Dans ce contexte, **nous vous invitons à participer le 4 avril 2024** à une **session de partage** intitulée **« L'élevage caprin et les microbes : entre risques et santé »** organisée sur le campus Terres & Paysages (lycée agricole de Melle).

(1) Brive C. et Zimmer A. *Écologie et promesses du tournant microbien* in Revue d'Anthropologie des connaissances, dossier thématique. 2021, 15-3 - https://journals.openedition.org/rac/24895

(2) Selosse M.-A. *Jamais seul. Ces microbes qui construisent les plantes, les animaux et les civilisations.* Éditions Actes Sud, 2017, 368 pages

remerciements

Nous tenons à remercier grandement les personnes enquêtées. Nous remercions également celles et ceux qui ont contribué de près ou de loin au projet EXPAIRCOX, à l'organisation des enquêtes anthropologiques et à la préparation de la journée ZOOJEU. Nous remercions en particulier, sans prétendre être exhaustifs :

• à l'ARS Nouvelle Aquitaine : Raquel Ceniceros, Renaud Pouget, Christine Ribreau, Maxime Robert

• à INRAE : David Abrial, Vanessa Boissery, Marie Brossier, Delphine Caray, Jocelyn De Goër De Hervé, Nathalie Gandon, Sabrina Gasser, François Johany, Roxane Jupin, Isabelle Lebert, Philippe Lecomte, Caroline Mignon-Falize, Didier Richard, Cyrille Rigolot

• aux GDS : Ghislain Audusseau, Manon Delalande, Nicolas Ehrhardt, Kristel Gache, Alice Jardin, Raphaël Lamothe, Karine Sommier

aux GTV : Thiéry Cazajous

• à l'Anses : Christophe Aubert, Marc Tabouret, Jaquemine Vialard

• à Qualyse : Michaël Treilles

• à l'IDELE : Renée de Crémoux

• les étudiants et encadrants du Master 2 Politiques publiques et gouvernements comparés impliqués dans les groupes de travail sur la fièvre Q l'année 2021-2022

• à l'Université Fédérale de Santa Catarina (Brésil) : Jeremy Deturche

• au tiers-lieu Le Mazier (03 Bourbon l'Archambault) et à l'association Champ des possibles (03 Saint Menoux), pour la confection des pièces de jeu
