# Supplementary material for: ZOOJEU: Using Q fever to highlight a participatory science approach for fostering the co-design of management strategies for neglected zoonotic diseases
Source: PLoS Negl Trop Dis. 2026 Oct 15;20(10):e0014618. doi: 10.1371/journal.pntd.0014618 (PMC13671574; doi:10.1371/journal.pntd.0014618)
Supplement: Supplementary file 4 [file pntd.0014618.s003.docx]

**S2. Game description**

***S2.1 Game facilitation***

ZOOJEU is designed to address disease-related issues, while simultaneously spurring the engagement of farmers. The latter is fostered by facilitators, who ensure that farmers' input receives specific attention. The role of facilitators is crucial in the success of the approach, particularly when there is a wide diversity of stakeholders, some of whom feel less legitimate than others to express themselves. During the game, players are asked to deal with a cluster of Q-fever-induced abortions on a goat farm; they must collaborate to overcome the associated challenges. The facilitator leading the game explains that the goal is not to impose a single 'truth' on everyone, but rather to start from each player's own perspective without judging the truth or appropriateness of what is said. The facilitator also explains that the farmer speaks first because they are most directly involved in what happens during the game. The farmer should describe what they see, think, and feel, as well as what they believe is right. The facilitator’s statement aims to limit potential hierarchical dynamics among players, given that veterinarians or researchers may possess a health mandate.

***S2.2 Game stages***

The game is played over four successive stages, which are detailed below as well as in **Figs 3 and 4**.

- First (Board A), the facilitator asks the players to list the potential factors that can affect a farm, considering more than economics and agronomics; then, the facilitator invites the farmer playing the game to describe their farm, which will provide the backdrop for the rest of the game stages (see **Fig 3**).
- During the second stage (Board B), there is a cluster of abortions among the farm’s goats. The facilitator places a red square 70 cm wide (see **Fig 4**) at top of the board to symbolise this event, which marks a shift away from routine farming practices. The facilitator then asks the farmer to describe, using the cardboard game figures, how they would handle such a situation: would it be a significant event to deal with and/or a cognitive burden? The figures are placed on the board to represent what arises from the event. The game’s other players are then invited to add other figures that illustrate the elements they find to be key in such situations (see **Fig 5**). Commonalities or differences in opinions can be noted by placing notes on the figures. During this stage, the objectives are to (1) provide the farmer with the opportunity to share how they cope with and respond to this health issue, without passing judgment or rendering an evaluation and (2) reveal the interdependencies and the intertwining of worldviews and knowledge of the different stakeholders.
- During the third stage (Board C), the focus is placed on what creates feelings of security or vulnerability in the situation. The farmer and the other players are asked to discuss their general relationship with health and with microbes. To this end, they are invited to carefully consider a range of figures representing standard biosafety recommendations and first-hand accounts of alternative therapeutic or healthcare practices. The physical act of picking up the figures and placing them on the board is viewed as essential at this stage of the game, as it helps players to open up and share their fears and feelings.
- During the final stage (Board D), the facilitator invites the players to collectively reflect on current health policy failures by selecting from a range of figures or by creating new ones (see **Fig 5**). Then, the facilitator proposes a creative exercise and asks the players to imagine redesigning Q fever management in such a way as to address the issues, uncertainties, and suboptimal approaches identified over the course of the game stages. For support, they may use figures that represent hypothetical health governance bodies or stakeholders that should be involved (see **Fig 5**).
