# Supplementary material for: ZOOJEU: Using Q fever to highlight a participatory science approach for fostering the co-design of management strategies for neglected zoonotic diseases
Source: PLoS Negl Trop Dis. 2026 Oct 15;20(10):e0014618. doi: 10.1371/journal.pntd.0014618 (PMC13671574; doi:10.1371/journal.pntd.0014618)
Supplement: Supplementary file 5 [file pntd.0014618.s004.docx]

**S3 Workshop preparation and implementation**

***S3.1 Recruitment of participants***

Our goal was to recruit 24 participants (8 in each set of players). We took great care when selecting the participants because their diversity and mutual complementarity would determine the richness and relevance of the collective experiment. Potential participants were identified in collaboration with local stakeholders involved in goat industry (REXCAP network), regional livestock health associations (GDS des Deux-Sèvres and FRGDS Poitou-Charentes), and a regional human health agency (ARS des Deux-Sèvres). We created a list of these potential participants; many had already been interviewed as part of the EXPAIRCOX project [Ramillien et al. 2025] and had stated that they agreed to be involved in future research. We defined broad categories of stakeholders to ensure that each set of players contained at least a farmer, a veterinary health practitioner, and local and national public health authorities from the veterinary and human health sectors. Farmers were contacted by phone, whereas veterinary health practitioners and public health authorities were contacted by email and invited to call if they wanted more information about the event. We offered financial compensation to farmers and veterinarians at rates aligned with those established by the national livestock health association (GDS France). Additionally, we covered travel expenses for participants coming from more than 200 kilometres away. The comic strip was mailed to each confirmed participant, along with a letter of invitation, on April 24, 2023.

***S3.2 Event preparation***

We chose a neutral location to balance power dynamics among the participants. The room was well-lit and spacious enough to allow three sets of players to play the game simultaneously, and it had a specific area dedicated to presenting the project’s framework. All necessary materials were prepared the day before the event. We set up the three game sets; each was equipped with a Zoom H1N audio recorder. We also arranged chairs around a large screen where participants could sit during presentations and video projections. We set up tables to welcome participants as they arrive, collect their signed GDPR documents, and give them the informational brochure and a feedback form to be completed at the end of the day. Name tags were prepared for each participant with their first name and their specific area of expertise: goat farming, animal health, or human health.

***S3.3 Practical organization***

The event began with the screening of the animated video. Next, the presentation was given to provide context for the research underway and to share preliminary results from local epidemiological research conducted as part of the EXPAIRCOX project. The participants were then divided into three sets of players, each with a facilitator and seven to nine participants with expertise in human health, animal health, or goat farming. During the workshop, photos were taken and the recorders were launched. The recorded voices were transcribed and archived using the Sygade platform (<https://sygade.inrae.fr/>) after the event.

***S3.4 Serious game implementation***

During the workshop, overall, players were energetic and committed, recognising the need to discuss Q fever and its management.

- During the first stage (Board A), the players were asked to provide a comprehensive description of the farm’s operational environment, drawing on the practical experience of the farmer(s) within the set of players. The 4 farmers at the event had between 100 and 500 goats, but their farming conditions differed markedly (see **Fig 6a** for details).
- During the second stage (Board B), for all sets of players, the cluster of abortions among the goats led to the emergence of diverse entities that had not previously existed on the farm. New stakeholders intervened, and certain farm entities were affected. Overall, the presence of Q fever disrupted the farm and redistributed its entities. Each player chose whether or not to mobilise these new entities, which led to intense discussions and negotiations. The players explained their perspectives and actions in an effort to establish a shared vision of what was happening on the farm. Tensions occasionally emerged regarding the handling of the abortions specifically and of diseases more generally. For instance, a veterinarian lectured a farmer on the importance of testing his goats, but the farmer considered that it was unnecessary and too expensive (see verbatims in **Fig 7**).
- The third stage (Board C) provided an opportunity to openly discuss fears and uncertainties. Intense discussions took place around the wide range of recommended biosecurity actions. The use of the serious game clearly helped create a bond among the players. It allowed them to discuss their emotions without feeling uncomfortable, which would have been an unlikely outcome during a traditional round table. There was a debate within one set of players regarding the transmission of Q fever via raw milk, which ended with the conclusion that no one seemed to be certain about the subject. There was a consensus that actions were guided by human health but that, in practice, 'these people were rarely at the table to discuss management decisions'. There was also a shared fear that 'the consequences of what we implement are sometimes more serious than the reasons behind the implementation'. This result underscores the importance of consulting with those who will be affected before taking action.
- During the fourth stage (Board D), the players were asked to propose ways to improve management strategies based on the discussions that had taken place during the earlier game stages. The figure representing a hypothetical Regional Council for Global Health was put to work by all three sets of players (see **S6 File**).

Finally, each set of players was given time to present their work, as shown in **Fig 6b**.

***S3.5 Handling of potential power imbalances among players***

Potential disparities and power imbalances among players in groups were handled by:

- using a nametag specifying only their first name and their specific area of expertise (goat farming, animal health, or human health); this choice clearly facilitated the interaction between participants; for example, the expert patient said to the authors that she would not have spoken in the same way had she known she was speaking to the hospital doctor who had raised the alarm about the cluster of cases;

- by facilitating the session in a way that ensures everyone, including the famer(s) and expert-patient, has a chance to speak. Special attention was essentially given to ensuring that the farmer(s) could speak first at each stage of the game, to counterbalance the implicit authority of veterinarians and healthcare professionals.

The effort made to address potential disparities clearly influenced the outcomes of the experiment.

Reference cited:

Ramillien E, Cayre P, Fourt X, Rousset E, Jourdain E. Q fever as an anthropological prism for revealing how farmers live with microbes Agriculture and Human Values 2025;42(1):527-43
