# Supplementary material for: ZOOJEU: Using Q fever to highlight a participatory science approach for fostering the co-design of management strategies for neglected zoonotic diseases
Source: PLoS Negl Trop Dis. 2026 Oct 15;20(10):e0014618. doi: 10.1371/journal.pntd.0014618 (PMC13671574; doi:10.1371/journal.pntd.0014618)
Supplement: Supplementary file 6 [file pntd.0014618.s005.docx]

**ZOOJEU self-evaluation document**

**We chose to conduct this evaluation document based on the self-evaluation guide for participatory science programmes developed by the *Collectif Vigie Mer:***

***Guide d'autoévaluation pour des programmes de sciences participatives mer et littoral. Groupe de travail « Indicateurs » - Collectif Vigie Mer*, version dated January 7, 2025. Available online (accessed on September 7, 2026):** [**https://collectif.vigiemer.fr/wp-content/uploads/2025/04/VF_Guide-autoevaluation-01.2025.pdf**](https://collectif.vigiemer.fr/wp-content/uploads/2025/04/VF_Guide-autoevaluation-01.2025.pdf)

The guide encourages the evaluation to focus on four project characteristics: (1) project relevance (alignment between objectives and field-related issues); (2) project effectiveness (alignment between objectives and results); (3) project usefulness (effects of actions in the field); and (4) project efficiency (achieving effects at a reasonable cost). The guide further suggests using a combination of indicators related to project monitoring, performance, audience characterisation, and impact that fit with the project’s objectives and context. Therefore, we used a subset of indicators consistent with the nature and context of our project. Below is the list of these indicators: the letters and numbers correspond to the indicator classification scheme in the *Collectif Vigie Mer* self-evaluation guide; the questions are written in black, and the answers are written in blue.

A. **Collaborative and participatory structure**

**A1. Stakeholder diversity**

- Number of participants: 23
- Number of professional profiles: 11 (farmer; veterinary practitioner; member of association for the chronically ill; veterinarian working in a veterinary analysis laboratory; and employees at 7 national and local institutions—see below); in addition, one participant was an expert-patient (also referred to as a partner-patient or resource-patient)—expert-patients are people who have a chronic illness and who have developed a detailed understanding of their condition, meaning they possess expertise in the daily experience of a pathology or a physical limitation related to their condition^[1]^.
- Number of institutions represented by the participants: 7; related to human health: **SPF** – *Santé Publique France* (French National Public Health Agency); **MSA** – *Mutuelle Santé Agricole* (Health Insurance Company for Agricultural Professionals); **ARS** – *Agence Régionale de Santé* (Regional Health Agency); **CHU** – University Hospital Centre); related to animal health: **Anses** – *Agence nationale de sécurité sanitaire de l'alimentation, de l'environnement et du travail* (French Agency for Food, Environmental and Occupational Health & Safety); **GDS** – *Groupements de Défense Sanitaire* (national livestock health association); related to farming: **IDELE** – *Institut de l’Elevage* (French Livestock Institute).
- Number of funding organisations supporting the programme: 2

**A2. Level of stakeholder involvement**

- Differentiate between the types of participants by categorising their level of engagement: in ZOOJEU, all participants were equally involved.
- How long do participants receive support from the research team? ZOUJEU was the first step in a collaboration between researchers and stakeholders. When this article was written, the collaboration was still ongoing as part of the *Transition ZOOJEU* project, a continuation of ZOOJEU.

**A3. Regional issues**

- Is the regional scale appropriate for the scientific question being asked? Yes. The project was designed to specifically address regional issues.

**A4. Networking**

- How many citizen science platforms include the programme? One—the project can be found on the Gamae platform: <https://ludotheque.gamae.fr/game/117>

**A5. Governance**

- Please describe whether or not governance is shared. Governance was shared by three researchers from three different scientific disciplines (epidemiology, sociology, public action design).
- Is a third-party monitor^[2]^ involved? Yes. A third-party monitor from the Directorate for Policy Support (DAPP) at the French National Research Institute for Agriculture, Food, and Environment (INRAE) helped guide all stages of the research process and provided external feedback on project design and implementation.

**A6. Funding**

- What is the source of programme funding? Public funding from the state (the DAPP at INRAE) and regional health authorities (ARS of Deux-Sèvres)
- Is the programme dependent on this funding? The programme is dependent on this funding.
- What is the average duration of support per funding source? DAPP: 2 years, ARS: 1 year
- Is the funding renewable? Yes
- Do the funding sources share the values promoted by the programme and the project leader? Yes

B. **Objective and scientific question**

**B1. Answering a scientific question**

- Did non-scientific participants contribute to formulating and refining the scientific question? If so, how? Yes. The stakeholders were interviewed as part of an earlier anthropological study, which led to the formulation of the research question for this study.
- Are the proposed methods appropriate for answering the question? Yes. We provided feedback on the results of the anthropological study and developed a tool that facilitates mutual recognition and understanding.

**B2. Objective that is not purely scientific**

- Is the objective clearly stated? Is the wording understandable to everyone? Yes. The objective was clearly explained on the last page of the comic strip, and it was reiterated during the introductory presentation at the start of the workshop.
- Are the methods, including the scientific aspects, appropriate for achieving the objective? Yes. Public policy design is relevant for establishing a common narrative for stakeholders and facilitating balance among them.

**B3. Origin of the question or the objective**

- Has the question or objective initially been raised by citizens via an association, which then reaches out to scientists? Yes. Initially, the scientists were approached by farmers, either individually or collectively through a livestock health protection association; the farmers were seeking recommendations for effectively managing Q fever outbreaks.
- Have non-scientific participants contributed to formulating and refining the scientific question? If so, how? They contributed through their interviews during the earlier anthropological study.

C. **Implementation and methodology**

**C1. Co-construction of protocol implementation**

- Were associative structures and participants involved in developing the protocol? Yes. The stakeholders were interviewed as part of an earlier anthropological study, which led to the *ad hoc* creation of the comic strip and serious game. Some stakeholders also played a key role in identifying potentially relevant participants. Local stakeholders (FRGDS, REXCAP) also communicated about the project before the workshop took place, giving stakeholders the opportunity to volunteer. A farmer contacted us spontaneously to participate.
- Are the proposed and implemented protocols consistent with the objective or scientific question? Yes. The comic strip and serious game were designed to promote discussion about disease management issues, taking into account both research findings and the perspectives of the relevant stakeholders.
- Have the main limitations, risks, and solutions for addressing these limitations and risks been identified?

The main limitation was the unavailability of stakeholders for a timeframe longer than one day, whereas our objectives were ambitious. The workshop schedule was optimised and adapted throughout the day to achieve the objectives within the available time. The main risk was that stakeholders would drop out of the workshop at the last minute (e.g., cancel with little notice or fail to show up with no notice). This risk was particularly high for farmers, whose participation was crucial but whose professional activities (e.g., crop farming) are strongly dependent on the weather (e.g., storms), the timing of goat kidding, and other variable events (e.g., sick farm employees). To address this risk, we invited more farmers than we needed. Of the 22 farmers we invited, 6 accepted the invitation, 4 participated in the workshop (2 were assigned to the same set of players), and 2 cancelled the day or the week before the event. In addition, to further encourage farmers to participate in the workshop, we offered them financial compensation at rates aligned with those established by the national livestock health association (GDS France).

- Have the protocols been adapted to account for specific regional characteristics? Yes. The protocol was designed to fit with the specific regional characteristics of Deux-Sèvres.
- When participants are required to have prior knowledge, are educational tools (including training) provided? Prior knowledge was not necessary, but we thought that, before the workshop, it would be helpful for workshop participants to be familiar with our research approach and the results of our earlier anthropological study. For this reason, we mailed the comic strip to the participants along with a formal invitation to the workshop one week before the workshop.

**C2. Shared contributions to data management (e.g., database design, data organisation) and analysis**

- How many participants are involved in data management and analysis? None

- How many support structures are involved in these stages? None

- Are the data subject to a validation protocol? No

- Do the data collected allow the scientific question to be answered? Yes

- Does the lead organisation or its partners have the necessary engineering, analysis, and

interpretation capabilities? Yes

**C3. Shared participation in the dissemination and communication of results**

- How many participants are taking part in these processes? None

- How many support structures are involved in these processes? None

- Has an information and communication network been set up? Are existing networks being exploited? Existing networks have been used (e.g., REXCAP, the goat commission of FRGDS, agricultural high school, DAPP at INRAE).

D. **Mutual benefits for stakeholders (project leaders and participants)**

**D1. Improvements in participant learning, skills, and knowledge (i.e., literacy)**

Does the project help strengthen literacy in relation to the following?

- Ecocitizenship: The participants acquired new knowledge/skills related to the current governance of zoonotic disease management and to Q fever pathophysiology and epidemiology.
- Research methodologies and instruments: The participants discovered that public policy design is a methodological approach for developing material forms of support (e.g., dispositives), such as comic strips and serious games. By symbolically representing ‘objects’, ‘stakeholders’, and ‘practices’, these materials encourage mutual recognition and dialogue among stakeholders.
- Collaborations: The participants gained a new understanding of the importance of establishing transdisciplinary collaborations among the biological sciences, humanities, and social sciences.
- Taking action: The participants explored a participatory research process in which different forms of knowledge were equally respected, which we postulate is a necessary prerequisite for efficient public health policies in livestock farming.

**D2. Recognition and support of stakeholder participation**

- Are there any means of honouring participant engagement and involvement? We offered financial compensation to farmers and veterinarians at rates aligned with those established by the national livestock health association (GDS France). We paid for lunch and reimbursed travel expenses for participants who travelled more than 100 km.
- Is an evaluation conducted of the participatory setting (e.g., evaluation of methodologies, adherence/understanding of co-constructed research phases, type of facilitation/workshops with stakeholders)? We did not conduct any type of formal evaluation, but the participants were given feedback forms to fill out, and the recordings obtained during the workshop and the subsequent discussion were transcribed.
- Is an evaluation conducted of participation intensity and related social benefits (e.g., degree of participant activity, interactions)? Participation during the workshop was fostered by the facilitators, who were responsible for encouraging the players to engage in group discussions.

With regards to the related social benefits, a significant outcome of the study was the strengthening of relationships between the MSA and stakeholders involved in goat farming or animal health. Indeed, the ZOOJEU workshop helped establish these relationships, and the exchanges that followed have built greater ties between the MSA and the regional goat industry. At present, these stakeholders are organising regular meetings and have launched an awareness campaign focused on zoonotic diseases (including Q fever) that uses posters and short videos. The aims are (1) to communicate with healthcare professionals so that they encourage their patients to mention that they work in livestock farming (through a poster placed in the waiting room); (2) communicate with healthcare professionals so that they think to ask their patients whether they work with livestock (via letters and flyers); and (3) communicate with farmers and their employees (by using a poster describing best practices for preventing zoonoses and short videos).

Another major outcome was the possibility of using ZOOJEU as a teaching support tool in diverse pedagogical situations (see **S7 File** for details). We have organised educational workshops with (1) 24 future farmers and agricultural advisors at the agricultural high school of Melle, which has a goat farm; (2) 24 third-year agricultural engineering students at VetAgro Sup in Clermont-Ferrand; and (3) 6 international students with professional experience as veterinarians, medical doctors, or health officials in their home countries, who were enrolled in the master’s programme One Health - Managing the Health of Populations at the University of Lyon. We have also been contacted by many other teachers (particularly those at agricultural high schools), who would like to play the game with their students. We are currently adapting the dispositive so that it can be used without our research team needing to be present.

Overall, the project’s main outcome is its continuation via *Transition* *ZOOJEU*, during which we have organised other participatory science events. The latter have allowed stakeholders involved in goat farming or animal health to discover regional health organisations with democratic structures, such as the Regional Congress for Health and Autonomy (*Conférence Régionale de la Santé et de l’Autonomie* [CRSA]) and the Regional Health Councils (*Conseils Territoriaux de Santé* [CTS]) of Nouvelle-Aquitaine^[3]^; stakeholders have also been able to engage in exchanges with some of their members. For further details, please see the video produced after the participatory science event that took place in Melle on October 2, 2025^[4]^.

- Are the results disseminated in a way that involves or acknowledges the participants? The participants have been systematically mentioned in the acknowledgements when the results have been shared at conferences or in publications, for example (see section D4 for details). Some of the participants were specifically named, depending on their level of involvement. In addition, one of the participants, a farmer, was interviewed by a journalist about the workshop, and the journalist subsequently published an article in the professional agricultural press (translated title: *Farmers are public health stakeholders without realising it*, published in *Réussir La Chèvre* magazine, March-April 2025, no. 387, page 30^[5]^). Two teachers at the agricultural high school of Melle also shared their experience of using ZOOJEU in an educational context in the same press outlet (translated title: *How the agricultural high school of Melle is turning Q fever into a teaching tool*, published in *Réussir La Chèvre* magazine, December 2025^[6]^).
- Is there any other form of recognition (e.g., certificate of participation, mention in official documents related to the project)? A summary of the workshop's events was mailed to individual participants three months after the workshop (see **S6 File**). The participants have been systematically mentioned in the acknowledgements of all documents related to the project.

**D3. Transformation of scientific methods and the work itself**

- Have any changes occurred in the relationships between the participants and researchers or the positions of either (provide details on any type of change)? The relationships between the participants and the researchers have become more friendly, reflecting a sense of equality (e.g., use of informal language in French). Participants helped and encouraged the researchers to continue the project in 2024–26, particularly by identifying people to survey (during master's research) and by facilitating participatory science days.
- Are there interdisciplinary facets to the project (e.g., collaboration among social sciences, humanities, and other disciplines)? Transdisciplinarity is at the core of the project, which seeks to bring together scientific, empirical, and practical knowledge and draw upon the social and biological sciences.
- Have the research questions evolved? The research questions have significantly evolved. We began the project with a focus on a specific zoonotic disease (Q fever), but the participants encouraged us to expand our research scope to address issues related to the interplay between health and welfare more generally. They also urged us to consider the governance of human and animal health at a regional scale. We have dedicated our next research programme, named *Transition ZOOJEU*, to exploring these issues^[7]^.
- Have there been shifts in the protocol or co-construction approach? We are constantly learning from our interactions with project partners and colleagues who have experience with participatory projects. While the overall research project has evolved to address broader scientific challenges, the ZOOJEU framework remains relevant for addressing Q fever-related issues with stakeholders (provided that the latter includes farmers) and in educational contexts.

**D4. Creation of scientific value**

- Has the programme resulted in scientific publications? Yes. This manuscript is one such publication. In addition, a manuscript will be published in French in the *Bulletin de l'Académie Vétérinaire de France*.
- How many scientific articles have been published as a result of the programme? There will be two scientific articles published specifically as a result of the ZOOJEU project (one in French, one in English).
- Has the program resulted in participation in symposiums or conferences? ZOOJEU has been presented at many professional and scientific events: a meeting with the General Directorate of Anses (Sophia-Antipolis, May 2023), a round-table discussion at the annual conference of the French Anthropological Association (Paris, November 2023), a presentation at the DAPP (INRAE) seminar (Paris, November 2023), posters and an oral presentation during the Goat Technical Days (Dienné, March 2024), a presentation to the representatives of the General Directorate for Research and Education (DGER) (Aubière, June 2024), a workshop session during the 3R Ruminant Research Meetings (Paris, December 2024), a brief online presentation during the Q Fever International Group Symposium (Byron Bay, Australia, June 2025), a presentation at the Q fever online webinar for French professionals (December 2025), a presentation to the French Veterinary Academy (visioconference, March 2026), and a presentation at the colloquium "Cartography Trouble. Counter-cartographies and paradigm shifts" (Paris, September 2026).

**D5. Other outcomes and forms of communication**

- How have the results been presented to the participants (e.g., in the form of posters, conferences)? A summary of the workshop's events was mailed to individual participants three months after the workshop (see **S6 File**). Oral feedback was made available to stakeholders in April and September 2024 during two participatory science days organised as part of the new project, *Transition ZOOJEU*.
- Have any articles for the general public been published in journals produced by associations, professional or academic societies, or regional authorities (etc.)? ZOOJEU was presented during the Open Days at the INRAE Research Centre of Theix (May 2024). In November 2025, Elsa Jourdain was interviewed by a journalist from the general press (*Libération*) who wanted to know more about the ZOOJEU approach (translated title: *After the ‘trauma’ of avian flu, farmers are preparing to face recurring epidemics*).
- Has a dedicated website or web page been developed for sharing the results? Yes. The project has a dedicated web page (<https://epia.clermont.hub.inrae.fr/pages-web/zoojeu>); a separate web page describes the project’s outcomes (<https://epia.clermont.hub.inrae.fr/pages-web/valorisations-zoojeu>). Both are associated with the website for the EPIA Research Unit. Information on ZOOJEU is also available from the INRAE DAPP website (https://app.inrae.fr/breves/zoojeu/). In addition, ZOOJEU is included in the Gamae serious game library: https://ludotheque.gamae.fr/game/117.

E. **Sociopolitical and environmental impacts**

**E1. Impacts on the environment and environmental challenges**

- Are the programme's environmental objectives being achieved? The project did not have any short-term environmental objectives.

**E2. Impacts on civil society and outreach**

- Has the programme increased levels of interest in target issues by non-participants? The discussions we had with members of the public at the INRAE Centre of Theix during the Open Days (May 2024) suggested that they were interested in the ZOOJEU approach. The approach was particularly relevant at the time, given that French farmers were facing issues related to the culling of livestock due to contagious nodular dermatosis. Elsa Jourdain was contacted in this context by a journalist at *Libération* in November 2025, who was interested in her experience with using mediation approaches with farmers. She was also contacted by a farmer with chronic Q fever symptoms who was seeking medical support.
- Have the programme’s results been integrated into school and/or university curricula? Are its resources used in educational contexts? ZOOJEU has already been used in a few high school and university courses. One of the current goals wis to make ZOOJEU available easily in educational contexts. The aim is to produce a version that can be used without the research team needing to be present to provide expertise and facilitation. This version of ZOOJEU should give equal importance to the various public health stakeholders (e.g., doctors, veterinarians, regulatory authorities, farmers, researchers) while also holding space for the friction and tensions that usually arise from differences in stakeholder approaches.

**E3. Impacts on local stakeholders and public decision-making**

- Has the participatory science programme received institutional recognition? Yes. ZOOJEU is promoted by INRAE, in particular the DAPP and the Animal Health Division. It has been included in the Gamae serious game library.
- Have the programme's results or data been taken into account and integrated into public policy? Not to date.
- Have the data collected by the project been integrated into public databases? The detailed personal data have not been integrated into public databases because such is not allowed by the GDPR.
- Has the programme led to changes in regulations or standards? No
- What other impacts has the programme had on regional stakeholders, particularly those in the private sector? The project initiated networking between stakeholders involved in goat farming, animal health, and human health, which we hope will last over the longer term. The project has met the expectations of at least some goat farmers: they felt listened to with regards to how they manage health issues. One of the farmers who participated in the workshop said: *'I've been waiting for this day my whole life'*.

F. **Consideration of ethical issues**

**F1. Copyright protection**

- Has a copyright policy been developed for the programme? The programme has followed the recommendations of INRAE’s Directorate for Open Science. A copyright policy development is underway.
- Are specific licences, such as Creative Commons licences, defined and available to stakeholders? Ultimately, our plan is to use a Creative Commons licence for ZOOJEU.

**F2. Intellectual property**

- Has an intellectual property management policy or charter been developed for the programme? An intellectual property agreement was collaboratively established by the researchers involved in the project.
- Is this policy/charter made available to stakeholders? This agreement has not been made available to stakeholders to date. It would be upon request.

**F3. Data sharing and confidentiality**

- Has a clear privacy policy been made available to stakeholders, in which data collection, storage, sharing, and protection are described? The data collected during this study are part of EPIA's data structure plan, which specifies methods for storing, sharing, and reusing data in accordance with FAIR (Findable, Accessible, Interoperable, Reusable) principles as well as ethical and legal constraints. Before the workshop began, participants were given a document stating that the information that they provided during the day would be used by the researchers for the purposes of the project and could only be communicated to INRAE staff involved in analysing the ZOOJEU data.
- Are sensitive data protected to ensure their confidentiality? Yes. All sensitive data are stored on secure INRAE-owned servers.
- Are personal data protected to ensure their confidentiality? Do the terms and conditions mention the GDPR and explain how compliance will be ensured? Can participants use pseudonyms to avoid disclosing their identity? During all of the project’s surveys and workshops, participants were asked to sign informed consent forms. Participants can withdraw at any time and request anonymity. The protocol was approved by the INRAE Data Protection Board with the support of the INRAE Data Protection Officer, thus ensuring that the study was carried out in accordance with the French GDPR.

**F4. The potential environmental impact of the (programme) activities**

- Has the programme carried out a carbon assessment? No
- Are there any proposed measures for reducing the programme's carbon footprint (e.g. carpooling platform, vegetarian meals, reusable tableware)? Carpooling to the workshop was organised. For people travelling alone from far away, we encouraged travel by train versus by individual car. After the workshop, leftovers from the meal were given to locals. The game materials were selected to have a minimal environmental impact.

**F5. Ethical aspects of the research question being addressed**

- Has the research question undergone ethical review to ensure social relevance, non-discrimination, and respect for human rights? The project promoted inclusivity, gender equality, and a diverse range of backgrounds (institutions, the agricultural world, teachers, users, researchers, artists). Particular attention was paid to making ZOOJEU accessible and limiting the degree of exclusion linked to institutional frameworks or the specialised nature of knowledge. Indeed, the ZOOJEU approach aims to limit asymmetrical power dynamics and promotes respect for different forms of knowledge, including experience-based knowledge.

**F6. Ethical aspects of programme funding**

- Are the sources of funding clearly identified and transparent? Yes
- Are the programme's funding sources ethical? Yes
- Does programme funding influence decisions and outcomes? No
- Are the motivations and interests of the funding sources consistent with the programme's objectives? Yes

1. *As* d*efined by the French National Authority for Health - Haute Autorité de Santé HAS: https://www.has-sante.fr/upload/docs/application/pdf/2020-11/fiche_pedagogique_engagement_patient_certification.pdf*
2. *Definition taken from the CO3 framework: a third-party monitor is a person chosen from outside the research group, whose mission is to support each stage of project implementation; particular attention is paid to the project’s co-construction and reflexivity—this balance must be defined according to the specific needs of each group.*
3. https://www.crsa-cts-na.fr/
4. https://mediatheque.inrae.fr/Clermont/embed/public/297247
5. https://www.reussir.fr/chevre/leleveur-est-un-acteur-de-la-sante-publique-qui-signore
6. https://www.reussir.fr/chevre/comment-le-lycee-agricole-de-melle-transforme-la-fievre-q-en-outil-pedagogique
7. See the following websites for more information:

   - https://sanba.hub.inrae.fr/rubriques-verticales2/nos-actions/projets-exploratoires/transition-zoojeu-projet-exploratoire-2024-2025

   - https://epia.clermont.hub.inrae.fr/pages-web/transition-zoojeu
