# Supplementary material for: ZOOJEU: Using Q fever to highlight a participatory science approach for fostering the co-design of management strategies for neglected zoonotic diseases
Source: PLoS Negl Trop Dis. 2026 Oct 15;20(10):e0014618. doi: 10.1371/journal.pntd.0014618 (PMC13671574; doi:10.1371/journal.pntd.0014618)
Supplement: Supplementary file 7 [file pntd.0014618.s008.docx]

**S7 Educational applications**: brief description of the use of ZOOJEU in educational contexts

We have used ZOOJEU as a teaching support tool in diverse pedagogical situations. We have organised educational workshops with (1) 24 future farmers and agricultural advisors at the agricultural high school of Melle, which has a goat farm; (2) 24 third-year agricultural engineering students at VetAgro Sup in Clermont-Ferrand; and (3) 6 international students with professional experience as veterinarians, medical doctors, or health officials in their home countries, who were enrolled in the master’s programme One Health - Managing the Health of Populations at the University of Lyon.

We found that ZOOJEU was particularly helpful in encouraging students with veterinary or medical backgrounds to explore field-based situations; they became more aware of the importance of considering all stakeholders when managing health issues related to zoonotic diseases. ZOOJEU also served to highlight the social issues that may arise from the implementation of management strategies. Because of its practicality, ZOOJEU was successfully implemented by the students at the agricultural high school of Melle, which has a goat farm. Their school farm served as the case study for the game, and the farmer was the school farm manager. The game’s representation of diverse health and biosecurity objects promoted greater reflection around the range of diseases that could occur on a farm. ZOOJEU was useful in highlighting the various approaches to health on a farm that may be mutually incompatible in terms of acceptability criteria.

When ZOOJEU was employed in educational contexts, it was essential to emphasise that the serious game does not involve role-playing. Role-playing would negate the approach’s emphasis on respecting the expertise of stakeholders and particularly that of farmers. Indeed, when the serious game was played by the agricultural engineering students, it became clear that ZOOJEU could not serve its intended role unless it could draw upon the practical experience of a farm as presented by its farmer. ZOOJEU requires a first-hand connection to the situation under consideration, not second-hand expertise without a concrete link to the context and in the absence of the relevant stakeholders. The feedback we received at the end of the educational workshops highlighted this point: the agricultural engineering students struggled to engage with the serious game, whereas the agricultural high school students readily navigated the gameplay, drawing on their experience.
