# Supplementary material for: ZOOJEU: Using Q fever to highlight a participatory science approach for fostering the co-design of management strategies for neglected zoonotic diseases
Source: PLoS Negl Trop Dis. 2026 Oct 15;20(10):e0014618. doi: 10.1371/journal.pntd.0014618 (PMC13671574; doi:10.1371/journal.pntd.0014618)
Supplement: Supplementary file 8 [file pntd.0014618.s010.docx]

**PNTD-D-25-00972
ZOOJEU: a public policy design approach to facilitate the co-conception of management scenarios for neglected zoonotic diseases, based on the example of Q fever
PLOS Neglected Tropical Diseases**

**Responses to Reviewers**

We want to warmly thank both reviewers for their highly constructive comments, which forced us to significantly improve the manuscript. We did our best to address all the comments and provide extensive information on the game, as required.

**Journal Requirements:**

1) Please provide an Author Summary. This should appear in your manuscript between the Abstract (if applicable) and the Introduction, and should be 150-200 words long. The aim should be to make your findings accessible to a wide audience that includes both scientists and non-scientists. Sample summaries can be found on our website under Submission Guidelines:
https://journals.plos.org/plosntds/s/submission-guidelines#loc-parts-of-a-submission

Done

2) Some material included in your submission may be copyrighted. According to PLOSu2019s copyright policy, authors who use figures or other material (e.g., graphics, clipart, maps) from another author or copyright holder must demonstrate or obtain permission to publish this material under the Creative Commons Attribution 4.0 International (CC BY 4.0) License used by PLOS journals. Please closely review the details of PLOSu2019s copyright requirements here: PLOS Licenses and Copyright. If you need to request permissions from a copyright holder, you may use PLOS's Copyright Content Permission form.
Please respond directly to this email and provide any known details concerning your material's license terms and permissions required for reuse, even if you have not yet obtained copyright permissions or are unsure of your material's copyright compatibility. Once you have responded and addressed all other outstanding technical requirements, you may resubmit your manuscript within Editorial Manager. 
Potential Copyright Issues:

- Please confirm (a) that you are the photographer of Figure 3., or (b) provide written permission from the photographer to publish the photo(s) under our CC BY 4.0 license.

- Figures 1, 2A, 2B, 2C, and and 2D.. Please confirm whether you drew the images / clip-art within the figure panels by hand. If you did not draw the images, please provide (a) a link to the source of the images or icons and their license / terms of use; or (b) written permission from the copyright holder to publish the images or icons under our CC BY 4.0 license. Alternatively, you may replace the images with open source alternatives. See these open source resources you may use to replace images / clip-art:
- https://commons.wikimedia.org
- <https://openclipart.org/>.

The photographs presented in the current Figures 3 and 5 and in the supplementary materials have been taken by two of the authors (Elsa Jourdain & Elodie Rousset). We have the consent of the participants to be visible in photographs, except for one participant whose face has therefore been blurred.

All images and icons have been drawn by one of the authors (Xavier Fourt)

The video (S2) has been prepared one of the authors (Elodie Rousset) based on the text and pictures of the comic strip (which was created by the authors).

**Methods:**
**General comments, key criteria for acceptance**

-Are the objectives of the study clearly articulated with a clear testable hypothesis stated?

Yes. (lines 196-198) *Our aim was to foster mutual understanding and respect among stakeholders and examine how Q fever management strategies could benefit from being co-designed with farmers*.

-Is the study design appropriate to address the stated objectives?
Yes. (lines 198-199) *We thus developed a mediation approach that we successfully tested with the relevant stakeholders.*

-Is the population clearly described and appropriate for the hypothesis being tested?
Yes. (lines 204-206) *Recruitment of participants for the multistakeholder workshop of the serious game started on April 17, 2019 and ended on April 24, 2023*. - (lines 370-380) *Our goal was to recruit 24 participants (8 in each set of players). We took great care when selecting the participants because their diversity and mutual complementarity would determine the richness and relevance of the collective experiment. Potential participants were identified in collaboration with local stakeholders involved in goat industry (REXCAP network), regional livestock health associations (GDS des Deux-Sèvres and FRGDS Poitou-Charentes), and a regional human health agency (ARS des Deux-Sèvres). We created a list of these potential participants; many had already been interviewed as part of the EXPAIRCOX project [25] and had stated that they agreed to be involved in future research. We defined broad categories of stakeholders to ensure that each set of players contained at least a farmer, a veterinary health practitioner, and local and national public health authorities from the veterinary and human health sectors.*

-Is the sample size sufficient to ensure adequate power to address the hypothesis being tested?
No, but this is not an issue for this study. There is no question here of claiming that the participants are ‘representative’ of their socio-professional category: our sampling efforts were neither exhaustive nor representative. Rather, the approach adopted aims at capturing the specific characteristics and features of the people recruited.

-Were correct statistical analysis used to support conclusions?
N/A

-Are there concerns about ethical or regulatory requirements being met?
Yes. The protocol was approved by the INRAE Data Protection Board, which ensured that the study was carried out in accordance with French General Data Protection Regulation (GDPR) principles. All study participants provided written informed consent.

**Reviewer #1:**

‘Anthropological analysis’ as a method is vague. Describe exactly how analysis of the results of interviews and observations carried out and what qualitative methods were used.

We rewrote the manuscript, as suggested below, to clarify that the anthropological data had been collected and analysed prior to the start of the current study. We therefore directly referred to the manuscript (i.e. Ramillien AHV) in which the methodology is described.

Clarify how the citing of reference 25 (i.e. Ramillien AHV) relates to the current study methods and specify what was done in this study and what was previously done.

See above. We hope that this is not clear in the manuscript. We dedicated a whole paragraph to the description of these data (see § 2.4.1, lines 267-288).

Explain by what process and by whom were the ‘anthropological’ results developed into the Worlds, whether they were validated by any of the participants, and if so at what step, which participants and by what process

As mentioned above, these data were analysed and published prior to this study. However, the "translation" of the results of the anthropological study into the comic strip and the design device is the result of a dialogue between the designer and the anthropologist (and more generally the authors as a whole). This work was notably essential in the selection process of the objects included in the serious game and the identification of potential boundary objects. (see lines 328-331) *We first created maps of the many objects identified. Then, through repeated exchanges, we selected the objects to incorporate into the serious game and built the narrative that would guide object use by participants.*

Specify the different groups of stakeholders involved in the anthropological part, who was involved, how many, how were they identified

This information is provided in Ramillien *et al*. (AHV), which is freely available online. Therefore, we did not feel the need to repeat this information, now that we clarified that this anthropological research was done prior to the current study.

More clearly contextualize and describe the worlds and clarify their relationship to each other e.g. do they represent co-existing realities? Separate ways of looking at the same reality?

We created a Figure (Fig. 1) to illustrate the concept of the worlds in a different way as in the original manuscript. Indeed, the concept of the worlds was decisive in the manner to design the comic strip and the serious game. These worlds are ideal types, that represent co-existing realities. We clarified this point :

- in the main text (lines 275-278): *A main finding was that livestock farmers viewed themselves as straddling two “worlds” that represent co-existing realities: in World A, cooperation and care for living organisms predominate whereas, in World B, management and control of living organisms predominate [25]*.

- in Figure 1's legend (lines 781-801): *World A and World B correspond to the ideal types described by Ramillien et al. [25] that represent co-existing realities [...]*.

**Clarify whether ‘public policy design’ always include scenarios as implied or was this a choice**, and clarify what parts of your description are established in PP design vs your own concepts of this design i.e. line 244, whether ‘this’ approach or ‘your’ approach

Simulation is one of the important applications of public policy design but PP design does not necessary implies simulations. We reworded the corresponding sentence as follows (lines 234-236): *we used public policy design, a scientific approach that facilitates the preparation, simulation, conception, and prototyping of novel forms of public action; it draws upon the findings of researchers while accounting for the perspectives of relevant stakeholders [57-60].*

**Include complete information on the nature of the device, who developed it**, etc. Much of the generic/objectives/benefits text in step 2 should be in the introduction.

We have totally revised the manuscript (as suggested) and included an extensive description of the dispositive*.* See § 2.5, lines 300-349. The dispositive was design by one the author, Xavier Fourt, who is an artist designer. This work was done through iterative exchanges with the other researchers, especially Elsa Jourdain.

Reviewer #2:

The methodology should be presented differently and should not include results. It should confine itself to defining the different steps followed, the conceptual and methodological frameworks in which they are rooted, explaining how the different steps relate to each other and what results are expected to meet the study's objective.

The has been entirely re-written according to these (highly relevant) recommendations.

In addition, a certain amount of information and detail is lacking to enable the reader to fully understand the methodological approach followed.

Extensive descriptions of the methodological approach has been added - See §2 Method section from line 200 to line 389

The results are partially presented in the methodology section (anthropological analysis) and lack detail.

Extensive details about the results have been added - See §3 Result section from line 390 to line 528. The anthropological data, which result from previous research, are presented in the Material and Method section - See § 2.4.1 from line 267 to line 288

It is difficult to understand whether Figure 1 is part of the material and method or of the results, as it has already been published in another study. In any case, it is presented somewhat disconnected from the text, and it's hard to understand what it represents and how it was used in this study. Similarly, we understand the rationale for using narrative graphs to communicate the results of an anthropological analysis to a wide audience; but in the context of a scientific article we'd like to know more about the raw data and methodology used to develop the messages. If this is presented elsewhere in another publication, then reference should be made to it and the narrative graphics should be presented in this study as materials used to facilitate the game (and not as results if this study per se).

We have created a new Fig 1 to present the results of Ramillien et al. in a different way as in the original study, in line with the graphic design used in the comic strip. We hope that this revision of the structure of the manuscript and of the figure make the role that these anthropological data had in the current study clearer.

In the interests of open science, raw data should be made available to the reader.
Unfortunately, the raw data cannot be made directly available because they are protected by the French GDPR regulation

It seems that the words control, management, surveillance and prevention are used interchangeably when they do not mean the same thing. it would be desirable to always use the same words to refer to the same thing. “its surveillance and control » ; « management and surveillance options » ; « the prevention and management of”, etc.

Thank you. We have chosen to focus the manuscript on the “management” of infectious diseases

Authors need to better explain why participation in decision-making for disease surveillance and control is an issue specific to emerging and neglected zoonotic diseases. why doesn't this apply to diseases in general?

We have addressed this point in the manuscript

- lines 84-87: *It is challenging yet crucial for public health strategies to account for all these perspectives while also addressing the unbalanced power dynamics that occur among stakeholders. Such is particularly important in the case of neglected and emerging diseases for which there are no specific regulations or dedicated sources of funding*.

- lines 530-532: *Unlike highly regulated diseases, neglected and emerging diseases allow for greater collaboration among stakeholders when it comes to the possibility of developing comprehensive public health strategies*.

Lines 88-91: Reading this sentence, one gets the impression that the need for harmonization is necessary not only between sectors, but also between countries within the same sector; if this is the case, we would need to be more explicit on this point, emphasizing that the need for harmonization is not only between sectors, but also between geographical areas.

This is the case. The sentence was rewritten as follows (lines 117-119): *There is also a need to harmonise human and animal Q fever surveillance data [22] given the considerable heterogeneity in Q fever surveillance protocols across Europe [13, 23, 24].*

Lines 91-93: I'm a bit confused reading this sentence - I have the impression that there are two ideas here: on the one hand, the lack of comparability between data to get a robust overview of the international situation, and on the other, the difference in quality of surveillance data which gives a biased representation of the relative importance of the disease between countries.

The sentence was rewritten as follows (lines 119-121): *Because the data vary in quality, it is impossible to conduct international comparisons, and there is a greater likelihood of misinterpretation, such as overestimating prevalence in countries with robust surveillance systems*.

Lines 119-123: This part of the text makes reference to recommendations for the management of Q fever in ruminant farms that are used to manage clusters of human cases occur. Can you clarify why recommendations dedicated to the livestock sector are used in case of human cases?

You put your finger on one of the main issues associated with the funding of human preventive measures: human authorities refuse to pay because the measures are implemented on farms and farmers refuse to pay to prevent the public. Because Q fever is aerially transmitted, preventive measure to protect human are essentially focused on limiting the contact between ruminants and humans. The fact that Q fever transmission is airborne is written on line 93: *The causative agent is the bacterium Coxiella burnetii, whose transmission is primarily airborne; it is a biosafety level 3 (BSL 3) pathogen.*

Then lines 129-130: if recommendations are used to deal with human cases, don’t we also need human health specialist around the table?

Yes, indeed. We added this professional profile in the sentence. See lines 157-159: *The stakeholders include not only veterinarians, human health specialists and risk managers, but also and above all, livestock farmers.*

Lines 128-129: Can we have a reference here? *expertise is only relevant if it is constantly evaluated, challenged, and adapted to align with the realities of the stakeholders involved*

We have inserted 3 references. (See lines 156-157): *expertise gains greatly in relevance if it is constantly evaluated, challenged, and tailored to align with stakeholder realities and if it is not biased by conflicts of interest [41-43].*

*41. Stengers I. Another science is possible. A manifesto for Slow Science. Press P, editor. Medford2018. 163 p.*

*42. Oreskes N, Conway EM. Merchants of doubt: how a handful of scientists obscured the truth on issues from tobacco smoke to global warming. Press B, editor. New York2010. 355 p.*

*43. Gardon S, Gautier A, Le Naour G. La santé globale au prisme de l'analyse des politiques publiques Versailles: Quae editions; 2020. 250 p*

Lines 133-134: Putting the sentence this way gives the impression that the recommendations are a priori lacking in pragmatism and relevance; is it really what we mean? I rather expected that their pragmatism and relevance should be questioned with field actors, but not judged unsuitable in the first instance.

We have rewritten this sentence as follows (lines 172-173): *The fact is that farmers are rarely represented in discussions between "experts" and health authorities, meaning the resulting recommendations may not be pragmatic or relevant.*

Line 136: “a perceived lack of knowledge”. Stated like this, it sounds like the lack of knowledge is perceived by farmers and not like public health authorities perceive a lack of knowledge among farmers
We have rewritten this sentence as follows (lines 170-173): *When public authorities see the lack of compliance with their recommendations, they tend to assume that farmers lack the requisite knowledge or have a poor understanding of the health risks,*

Lines 134-138: can we add a reference here? *When faced with a lack of compliance with recommended sanitary measures to control an infectious disease, public authorities most often attribute this to a perceived lack of knowledge or poor risk perception on the part of farmers, thus motivating communication campaigns aimed at "correcting" these behaviors*
We have rewritten this sentence as follows (lines 170-173): *When public authorities see the lack of compliance with their recommendations, (...) their response is to create communication campaigns aimed at "correcting" these issues [44].*

*44- Figuié M, Eduardo-Cuinhane C. Intégrer les enjeux sociaux et dépassr le cadre des études « connaissances, attitudes, pratiques ». Un défit majeur pour les approches interdisciplinaires. Approches interdisciplinaires en santé animale Dialogue entre sciences sociales et vétérinaires. Versailles: Quae editions; 2024. p. 171-82.*

Lines 138-142: expertise from farmers are not only about constraints - it's a bit reductionist and in contradiction to the rest of the paragraph, which describes the full extent of their expertise

Indeed. We have rewritten the § as follows (See lines 177-184): *Livestock farmers possess unique expertise [45] that they have cultivated through their daily interactions with communities of diverse species, including plant, animal [46], and microbial species [47]. They have gathered practical empirical knowledge about their animals and their working environment [48], which is critical to understanding the transmission dynamics of diseases such as Q fever [25]. Their daily observations, their experience with animal behaviour, and their awareness of subtle changes in farming conditions can provide valuable insights that are likely to be overlooked by academics or medical experts who are less familiar with realities in the field.*

Lines 167-170. In this introduction to the methodology, it would be interesting to specify how the two steps are articulated and their respective outputs to contribute to the overall objective of the study

We have now dedicated a full section on the methodological framework (§2.3) and specific steps (§2.5).

Lines 171. what investigations are we talking about? just before, we're talking about investigation, but in the singular. is it the same thing? we need to be more precise.

Thanks for this comment. The paragraph was modified to gain in clarity. See lines 209-210: *Our research took place after a Q fever outbreak*

Lines 171-173. when did this outbreak take place? how many farms, animals, human cases? please, briefly describe this outbreak.
The outbreak occurred between April 15 and June 13, 2017. It involved 11 human cases according to the national reference centre. The probable animal source of this outbreak could not be identified. The text was modified as follows (lines 211-213): " *Our research took place shortly after a Q fever outbreak, whose exact source remained unidentified despite intensive medical and veterinary efforts [49]. This outbreak occurred between April 15 and June 13, 2017, and involved 11 cases confirmed by the National Reference Centre for human Q fever [13, 49]*"

Line 185. What do we mean by: “whose farms were in contact with the public”. A farm cannot be in contact with the public, do we want to men “open to the public”? If yes, can you explain why this is important in the context of the study and why this type of farm was targeted?

Thank you. Indeed, we referred here to farm facilities open to the public. Zoonotic risks may exist for visitors because the disease is airborne. This section has been removed since these elements are extensively described in the original study (Ramillien et al. reference 25).

Lines 184-187. We'd like to know more about the study population. What do we mean by animal health, and human health professionals? how many farms recruited, how many people recruited in each stakeholder category? Did all individual invited agree to take part of the study? what is the link between the farms and the participants? do all the participants interviewed have a link with the selected farms? we'd also like to know how farms and participants were selected and have access to the interview guide (in supplementary file for example). line 187. What do we mean by anthropological analysis? How did this lead to the identification of the worlds?

This section has been removed since these elements are extensively described in the original study (Ramillien et al. reference 25).

Line 193: who are these “others”? what type of delegation is it?

We provided examples in the text of whom the 'others' may be - See line 280-281: *other stakeholders (e.g., veterinarians, goat feed advisers, dairy inspector).*

Lines 191-193: If “others” are veterinarians or vet labs, I do not see how it is possible for some farmers not to delegate health management to these stakeholders. Please better explain these two categories of farmers (high delegation versus low delegation).

We have explained the specificity of the goat farming situation in the study area - See lines 281-284: *This situation is somewhat specific of the goat farming sector, given the low economic value of an individual goat: the cost of a veterinary appointment (€80) far exceeds the market value of the goat (€8–10), meaning that farmers end up managing their animals’ health issues, especially when they have moderate-sized herds.*

Further details about the range for minor to major delegation (as ideal types) and its variable impact are provided in the manuscript from Ramillien et al. (see figure 3).

Line 197: I don't feel that the figure legend correctly illustrates the content of the figure and correctly explains what it represents. For example, in world A, we talk about death, sensory experience; etc. how is this illustrated in the figure? in addition, we miss information in this legend. What are the complex and challenging relationships? What do we mean by authentic and nurturing experiences? etc. conversely, the figure is mentioning “evil spirits”; this is not explained in the figure
Why are there two different types of farmers represented? The ones who delegate health management and the ones who don’t?

The figure was totally redrawn and the legend modified. He hope that we have gained in clarity.

Lines 210-212: the purpose of the narrative graphic needs to be better explained here; see previous comment about the need to summarize the methodology at the start of the methodology section to explain the different steps, how they relate to each other and how they contribute to the final objective of the study

We have written a whole § to explain the comic strip.

Line 224: the complete graphic narrative should be available in a supplementary file

Done. See Supplementary Material S1 Comic strip

Line 226: “2a: Public action in response to disease outbreak” disease outbreak in general or Q fever outbreak specifically?

This figure has been removed

Line 228: do we really want to mean “poorly” here? Poorly generally has a negative connotation. It implies a lack of quality. I think here we rather want to say that the delegation is low and not of bad quality

This figure has been removed

Lines 236- 238: I'm surprised to read this sentence. *Transforming research findings into tangible actions can be significantly facilitated by the design of a simulation device that can be implemented collectively through a structured methodology.* For me, and also from what I understand from reading this article, the aim of these participatory approaches is not to transform research results into action, but to collectively define actions that integrate scientific and non-scientific expertise.
“The device was designed to solicit insight from all team members, leveraging their unique
perspectives and areas of expertise.”

It is not just a matter of collectively defining actions that integrate scientific, empirical and practical knowledge. It is also about transforming the way research is conducted, given that research on livestock health intersects scientific fields that (1) require transdisciplinarity, and (2) require the alignment of different types of knowledge (scientific, empirical, practical). It is then a question of transforming research results into action, since it is a question of transforming public action to bring it more into line with the complexity of the reality of livestock farming and its health challenges, between animal health, human health, environmental health and social health.

We have added a paragraph to address this point - See lines 558-565: *As research on zoonotic diseases occurs at an intersection of many scientific fields and areas of expertise, it is imperative to adopt a transdisciplinary approach, in which scientific, empirical and practical knowledge align. Next, research findings must be translated into actions to ensure that public health policies truly address the complex challenges related to animal, human, environmental, but also societal health [44, 79]. An important aspect of this work is acknowledging that public health 'experts' are not the only ones with knowledge and skills: farmers are also highly knowledgeable and skilled even if they do not, at present, have any sort of official recognition.*

**Results**

**General comments, key criteria for acceptance**

**-Does the analysis presented match the analysis plan?**
Yes

**-Are the results clearly and completely presented?**
Yes

**-Are the figures (Tables, Images) of sufficient quality for clarity?**
Yes

Reviewer #1:

The presentation of figures as headers is confusing – clarify and separate hypotheses, methods and results being presented and describe how and by whom they are used. Present the results and then reference the figures. Figures should include standard figure legends and describe other aspects related to the figures as part of the (background? Result?) text.

Clarify steps and results of the game – currently they are vague and generic.

Done - See § 3.1.2 and 3.2

Present examples of the insights generated through the game.

Done - See § 3.2 and S3 Self-evaluation form

Authors assume that the reader understands how the game is played. Include a step by step discussion of this so we can then understand what ‘The culmination of the game was the three groups’ independent selection of the "Territorial Council for Global Health" figurine’ means

We included extensive description of the game stages. See 3.1.2, 3.2.1 as well as Figures 2 and 3
See also lines 712-718: *The fact that all three sets of players independently selected the figure representing the hypothetical Regional Council for Global Health reflects that stakeholders see a need for a regional body that considers the viewpoints of all stakeholders, not just those who are usually called upon by health authorities. Such a council would allow traditionally excluded stakeholders to be heard and engage in dialogue around concrete matters, such as measures that improve the welfare of living organisms, for whom livestock farmers care*.

Reviewer #2:

They are some partial results about the anthropological analysis, but any results about the outputs of the game. For instance, what are the recommendations that arose from the discussions

See comments in the Methods section.

Lines 277-278: can we break down the stakeholder categories a little more by profession and sector of activity (public or private? for example, who are the 12 animal health professionals? veterinary officer? private vets? microbiologists in vet lab? What is a patient-expert? are the participants the same as those interviewed for the anthropological analysis?

Done. See S3 self-evaluation form

Line 274: details of the different phases of the game and how they are facilitated are missing
For instance, how facilitators help participants in the formulation of proposals for enhanced
disease management strategies, based on the game outputs?

Thess aspects have been explained in the new version of the manuscript. See § 3.1.1 and 3.2.1

Lines 287-288: I do not understand the juxtaposition of these two sentences (*New actors intervened; certain farm entities were affected.*). What is the link between the news actors involved and new farms affected? They are examples of entities that were previously non-existent? Please clarify

The sentence has been removed

Lines 288-289: what do we mean with the redistribution of entities? In time? In space? In terms of responsibilities?

The objective of the game is to outline what entities/actors/action appear after the emergence of the outbreak depending on the diverse stakeholders. This redistribution can be indeed in time, in space, in terms of responsibilities... The cardboard figures displayed in Figure 4 and S4 brochure may be used as supports but the players may create any other entity to fit with their reality. See lines 480-484: *During the second stage (Board B), for all sets of players, the cluster of abortions among the goats led to the emergence of diverse entities that had not previously existed on the farm. New stakeholders intervened, and certain farm entities were affected. Overall, the presence of Q fever disrupted the farm and redistributed its entities. Each player chose whether or not to mobilise these new entities, which led to intense discussions and negotiations*.

Lines 294-295: I wonder if this sentence (*A figurine representing a fictitious Territorial Council for Global Health was mobilized in all teams*) doesn't belong higher up in the paragraph, as it is an additional entity (if I am right)

Thanks. The whole text has been revised

Line 294: what is a Territorial Council for Global Health? What are its role and missions? Why was it introduced in the game?

The hypothetical Regional Council for Global Health appeared as a missing piece in the current governance of zoonotic diseases. See lines 620-623: *(...) all three sets of players independently selected the figure representing the hypothetical Regional Council for Global Health as a missing piece in the current governance of zoonotic diseases. Discussions then took place to define the tasks and members of such a putative council.*

**Conclusions**

**General comments, key criteria for acceptance**

**-Are the conclusions supported by the data presented?**
Yes
**-Are the limitations of analysis clearly described?**
Yes
**-Do the authors discuss how these data can be helpful to advance our understanding of the topic under study?**
Yes
**-Is public health relevance addressed?**
Yes

Reviewer #1:

In the last section, include any impacts of the convening of the game, including commitments to or actual changes in behaviour relative to public and animal health.

Done. See §4 and S3 Self-evaluation form

Include who evaluated the game for its future use in these settings and what was the outcomes, and by what means have these conclusions been reached?

Done.

Reviewer #2:

Lines 300-301: can you provide examples of this realization? *The discourse among the participants was both engaging and insightful, culminating in moments of realization for the team members*

The term 'realization' has been replaced by 'discovery'. Examples have been provided (see lines 605-608): *The conversations among the players were both engaging and perceptive, culminating in moments of discovery (e.g., about the lack of scientific knowledge about the disease; or farmer workload during the goat kidding season; or the diagnostic odyssey of patients with chronic disorders).*

Line 305: what is this shared concept of risk that participants agreed on? *Similarly, discussing how risks are defined and perceived by each stakeholder helps clarify why different actions are taken by the different parties, thus allowing a reconsideration of a shared concept of risk*

The sentence has been revised as follows. See lines 611-613: *Similarly, discussing how risks are defined and perceived by each stakeholder can help clarify why different actions are taken by the different parties, thus prompting the group to reconsider the concept of shared risks*.

Lines 305-308: *By fostering mutual understanding and acknowledging the specific needs and experiences of all stakeholders, strategies for disease management inherently become more effective and respectful of individual perspectives.* I agree that strategies for disease management become more respectful of individual perspectives, but I'm not sure that taking into account the perspectives of different stakeholders necessarily makes strategies more effective, as other elements come into play. In any case, **this deserves a reference**

We have rephrased the sentence as follows. See lines 613-615: *By fostering mutual understanding and acknowledging the specific needs and experiences of all stakeholders, strategies for disease management strategies could become inherently more effective and respectful of individual perspectives.*
We have invited the reader to become aware of Community health experiences. See lines 616-617: *Community health experiences in West Africa and South Asia are inspiring in this regard [66, 75]*.

We have used the following references as examples:

-66: Bordier M, Duboz R, Sachet E, Antoine-Moussiaux N. Co-développer des stratégies de prévention et de contrôle des maladies animales par la recherche-action participative. In: Ducrot C, Fortané N, Paul M, editors. Approches interdisciplinaires en santé animale Dialogue entre sciences sociales et vétérinaires. Versailles: Quae editions; 2024. p. 102-16.

- Israel BA, Eng E, Schulz AJ, Parker EA. Methods in Community-Based Participatory Research for Health. San Francisco: Jossey-Bass; 2005. 479 p.

In addition, do you have evidence that participatory process was able to capture the perspectives of the different participants? Despite all efforts to encourage the various participants to raise their voice, it always remains power imbalance among some participants that will prevent some of them to express their opinions; moreover, the perspectives shared by the individuals present are not necessarily representative of those of the stakeholder category they represent.

See S3 Self-evaluation form. We have also added a few lines in the main text:

- see lines 682-689: *we have no evidence that our participatory process succeeded in capturing the stakeholders’ different perspectives. We created space for a magical religious model of disease prevention [82] to be expressed alongside other models of disease prevention, in parallel with or as a substitute for the profane constraint or Pasteurian models that would probably have been promoted by the veterinarians with health mandates. Although we clearly stated at the workshop, prior to gameplay, that everyone was free to voice their opinions about what they consider appropriate in terms of health measures, the presence of health authorities may have been enough to suppress the expression of non-conformist views.*
- see lines 704-711: *we did not assume that the players were acting as representatives for others of the same profession or social category. Rather, our approach takes into account the specific characteristics of the study context, which affects not only the stakeholders but also the ways in which regulations are applied or livestock farming is conducted. Furthermore, we have no evidence that the stakeholders who played the game were all similarly interested in a comprehensive approach to health. During gameplay, we witnessed multiple and not necessarily compatible perspectives on health and public health strategies, which fits with what was found by the anthropological research highlighted in the comic strip.*

**All this deserves to be discussed as limits to the participatory process**. **There is a whole body of literature available on the subject**

Done. See §5.

Actually, a whole review paper would be needed to tackle this issue properly.

Lines 311-313: why the three groups’ independent selection of the "Territorial Council for Global Health" figurine was considered as the culmination of the game was? What were the tasks defined for this Council?

*See lines 711-719: The fact that all three sets of players independently selected the figure representing the hypothetical Regional Council for Global Health reflects that stakeholders see a need for a regional body that considers the viewpoints of all stakeholders, not just those who are usually called upon by health authorities. Such a council would allow traditionally excluded stakeholders to be heard and engage in dialogue around concrete matters, such as measures that improve the welfare of living organisms, for whom livestock farmers care. We will continue to pursue the participatory process to further investigate this issue with a panel of stakeholders, including farmers, health and farming professionals, everyday citizens, and elected officials [84].*

Line 316: what do we mean by “the issue of asymmetric positions due to legal and regulatory frameworks”? *Regarding Q fever management specifically, there is a recognized need to continue discussions on a shared vision of risk and the issue of asymmetric positions due to legal and regulatory frameworks*

Lines 546-549: *Health policies related to livestock farming are often the work of professionals and government institutions or authorities, who, unlike farmers, have mandates to make decisions. One of ZOOJEU's challenges was to address these asymmetrical power dynamics by exploring alternative approaches to developing public health strategies.*

Lines 328-330: why is the tool more specifically adapted to neglected and emerging diseases?

Lines 530-532: Unlike highly regulated diseases, neglected and emerging diseases allow for greater collaboration among stakeholders when it comes to the possibility of developing comprehensive public health strategies.

Lines 84-87: *It is challenging yet crucial for public health strategies to account for all these perspectives while also addressing the unbalanced power dynamics that occur among stakeholders. Such is particularly important in the case of neglected and emerging diseases for which there are no specific regulations or dedicated sources of funding.*

And how its effectiveness has been evaluated?

See S3 Self-evaluation form

Line 330: “This engagement is reinforced by facilitators”. I'd go even further and say that the quality of facilitation plays a crucial role in the success of this type of approach, particularly when there is a wide diversity of stakeholders, some of whom feel less legitimate than others to express themselves

Indeed. We have clarified this point in the manuscript. See lines 639-645: *ZOOJEU's design appears to be particularly effective for addressing issues related to neglected or emerging infectious diseases, while simultaneously spurring the engagement of farmers. The latter is fostered by facilitators, who ensure that farmers' input receives specific attention: their role is crucial in the success of the approach, particularly when there is a wide diversity of stakeholders, some of whom feel less legitimate than others to express themselves. As a result, the serious game specifically tackles the power imbalance traditionally experienced by farmers in disease management contexts*.

Lines 334: is the term device appropriate?

Thank you for this comment. We have chosen to use instead the term “dispositive” (*dispositif*) with reference to the book of Valerie Larroche (2019). The Dispositif: A Concept for Information and Communication, Sciences. Hoboken, NJ: John Wiley & Sons. p. 83

**Editorial and Data Presentation Modifications?**

Use this section for editorial suggestions as well as relatively minor modifications of existing data that would enhance clarity. If the only modifications needed are minor and/or editorial, you may wish to recommend “Minor Revision” or “Accept”.

Reviewer #1: Not sure if formatting of sections conforms to standard criteria
Some type edits needed e.g. Bullets in intro from line 40

Modified

Submit evidence of prior informed consent obtained from all participants, including the photograph.

Done.

**Reviewer #2: (No Response)**

**Summary and General Comments**

Use this section to provide overall comments, discuss strengths/weaknesses of the study, novelty, significance, general execution and scholarship. You may also include additional comments for the author, including concerns about dual publication, research ethics, or publication ethics. If requesting major revision, please articulate the new experiments that are needed.

Reviewer #1:

The information itself is very interesting, and the rationale and outputs are important so worth trying to publish this.

- However, the presentation is chaotic and therefore difficult to understand exactly what was done.
- The background, methods and results should be presented more systematically including the stepwise processes for identification of participants, data collection, developing the figures, etc. Separate into sections for background, methods, etc . that could still be within the steps currently presented, or grouped across steps.

The manuscript has been entirely rewritten to fit with the reviewers' comments

Reviewer #2:

The study introduces ZOOJEU, a participatory public policy design tool in the form of a serious game aimed at improving the management of neglected zoonotic diseases, using Q fever as a case study.

The approach involves two main steps: (1) an anthropological study to capture the diverse experiences and knowledge of stakeholders (especially farmers), and (2) the design and implementation of a game to simulate disease management scenarios.

The authors argued that the game fostered collaboration between stakeholders, enabling them to jointly explore solutions tailored to the context and adapted to their perspectives. They also described ZOOJEU as a promising approach to promotes inclusive dialogue, mutual understanding, and health democracy, with potential applications beyond zoonoses.

- Although the approach presented is compelling, the article lacks sufficient detail regarding the methodology used and the results obtained.

Extensive details about the methods have been added

- Moreover, while the authors express strong enthusiasm about the game's potential to harness collective intelligence for developing effective, context-sensitive policy solutions, they provide no concrete examples of outputs and outcomes generated by the game, nor any evidence supporting its ability to achieve the intended goals—such as integrating diverse knowledge, fostering mutual understanding, building a shared vision, or designing effective and inclusive risk management strategies. Simply adopting a participatory approach does not guarantee relevant or effective results**; it must be complemented by a rigorous evaluation of its implementation, outputs, and impacts.**

A self-evaluation form is now available: S3 Self-evaluation form

I was also disturbed by the structure of the article adopted. The authors have chosen not to have specific sections dedicated to methodology, results and discussion, and as a result it is sometimes difficult to identify what is "materials and methods" or results specific to this study.

A Material and Method and Result sections have been added. See §2 and §3

The discussion is very limited, with no consideration of the potential biases associated with this type of approach, or of potential measures to improve the game.

An extensive discussion of the study has been added. See §5

As a non-native English speaker, I noticed what seem to be some Gallicisms that could lead to misinterpretations. I believe that further editing would enhance the clarity and readability of the text, as well as improve its overall quality. In addition, editing could help eliminate redundant sections and make the manuscript more concise.

Done (Jessica Pearce-Duvet)

The list of references should be reviewed; I've identified a few references that were incomplete (e.g., 44), with strange date format for the access date, or with small typos.

Done
