# Supplementary material for: ZOOJEU: Using Q fever to highlight a participatory science approach for fostering the co-design of management strategies for neglected zoonotic diseases
Source: PLoS Negl Trop Dis. 2026 Oct 15;20(10):e0014618. doi: 10.1371/journal.pntd.0014618 (PMC13671574; doi:10.1371/journal.pntd.0014618)
Supplement: Supplementary file 9 [file pntd.0014618.s011.docx]

**Response to reviewers - PNTD-D-25-00972R1**
ZOOJEU: using Q fever to highlight a participatory science approach for fostering the co-design of management strategies for neglected zoonotic diseases
***PLOS Neglected Tropical Diseases***

We would like to thank the editor and both reviewers for their helpful suggestions for improving our manuscript. Please find below our responses to the respective suggestions and comments.

**Additional Editor Comments (if provided):**

Thank you for revising this paper and responding to reviewer comments. I think the paper is much stronger on the technical side. However, at almost 9,000 words, it is much longer than similar papers published in PLOS NTDS. While recognising that different writing styles can vary among disciplinary traditions, I think the paper does require substantial revision to make its findings accessible and relevant to the readership of PLOS NTDS.

The manuscript now counts 6152 words. We hope that this will be acceptable for PLOS NTDS.

Some ways the paper narrative could be made stronger are suggested below.

Introduction: Retain focus on transdisciplinary aspects and condense discussion on Q-fever epidemiology

The changes made have reduced the length of the introduction from 1,636 to 870 words (removal of 766 words)

Methods section: This is overtly descriptive. I suggest revising section into a concise narrative focused on reproducibility rather than completeness. Information on recruitment and workshop logistics, etc. could be moved to supporting information.

The changes made have reduced the length of the MM section from 2,342 to 1,984 words (removal of 358 words). As suggested, a supplementary Material (**S4 Workshop preparation and implementation)** was added to provide a short description of the methodology followed for the recruitment of the participants and the multistakeholder workshop practical organisation.

Results: You might consider removing step-by-step descriptions of the game to supplementary material and retain only findings linked to your central claims.

The changes made have reduced the length of the results from 1,886 to 910 words (removal of 976 words). As suggested, a supplementary Material (**S3 Game description**) was added to provide a step-by-step description of the ZOOJEU game stages and facilitation directives.

Discussion: Streamline section by eliminating repetition with the Introduction and Results. Move detailed descriptions of tools (comic, game mechanics, workshop organisation) to supporting information.

The changes made have reduced the length of the discussion from 2,948 to 2,294 words (removal of 654 words). As suggested, detailed descriptions regarding applications as an educational tool were moved to a supplementary Material (**S8 Educational applications**) and we created a new Figure (Figure 7) to make verbatim quotes more visible. We did our best to limit the repetition with the previous sections (in this aim, we moved some text to the result section). Apart from these changes, we voluntarily limited the reduction of this section because many of the details included here had been added upon request of the reviewers. We consider that the discussion section is important to keep long for such an article. We hope that you will be satisfied with this new version. Of course, we remain open to making new changes if necessary.

Supporting information: It is good practice to ensure all supplementary materials are explicitly signposted in the main text, as done effectively for S3.

We checked that all supplementary material is appropriately cited.

Figures: If helpful, additional figures (e.g. flowcharts for methods and mind maps for analytic framework) could be used to streamline and advance narrative.

Thank you for this suggestion. We created Figure 1 to provide a clearer explanation of the approach's framework.

The authors should feel free to go beyond these suggestions or to consult a professional copy-editor to improve the overall clarity of their paper.

**Journal Requirements:**

If the reviewer comments include a recommendation to cite specific previously published works, please review and evaluate these publications to determine whether they are relevant and should be cited. There is no requirement to cite these works unless the editor has indicated otherwise.
1) Thank you for including an Ethics Statement for your study. Please include:
i) The approval number(s), or a statement that approval was granted by the named board(s) Done. Approval number is TRT-206. This number was added in the Ethics Statement section (§ 2.1, lines 324-325)

ii) A statement that formal consent was obtained (must state whether verbal/written) OR the reason consent was not obtained (e.g. anonymity). NOTE: If child participants, the statement must declare that formal consent was obtained from the parent/guardian.].

Done. Formal written consent was obtained. This information is written in the Ethics Statement section (2.1, lines 326-327)

**Reviewers' Comments:**

Reviewer's Responses to Questions

**Key Review Criteria Required for Acceptance?**

As you describe the new analyses required for acceptance, please consider the following:

**Methods**

-Are the objectives of the study clearly articulated with a clear testable hypothesis stated?

-Is the study design appropriate to address the stated objectives?

-Is the population clearly described and appropriate for the hypothesis being tested?

-Is the sample size sufficient to ensure adequate power to address the hypothesis being tested?

-Were correct statistical analysis used to support conclusions?

-Are there concerns about ethical or regulatory requirements being met?

**Reviewer #1**:

Please clarify whether the case study in the game was based on the actual outbreak in 2017 and whether this was in the same location as the research, and if so how this might have impacted results

Thanks for this question. The source of the outbreak in 2017 remained unidentified. It may be a goat farm, given the fact that the latter are numerous in the area, but this could not be documented. The case study in the game considered the occurrence of Q fever abortion in a goat farm. During the workshop, the considered farm differed between the 3 groups of players; in practice, the farmer(s) playing the game were invited to describe their farm. For 3 out of the 4 farmers, the farm was present within the exposure zone of the human cases in 2017. The 4th farmer had been personally sick with Q fever in the past. These points were clarified:

- lines 807-808: " *First, each set of players was asked to analyse the context of a particular goat farm that would serve as the case study. In practice, the farmer(s) playing the game were invited to describe their farm*."

- lines 1199-1201: " *The results may have been impacted by the fact that the study area had been hit by an outbreak of human Q fever, and that some participants had previously been infected with Q fever."*

Can condense by deleting repetition of how the game is meant to be played and then how it was actually played.

Done. The description of how the game is meant to be played was removed to supplementary material S4 Game description.

Please expand more specifically on how potential disparities and power imbalances among players in groups were handled? Do you think these played a role in outcomes?

Potential disparities and power imbalances among players in groups were handled by:

- using a nametag specifying only their first name and their specific area of expertise (goat farming, animal health, or human health); this choice clearly facilitated the interaction between the expert patient and the medical doctor: the expert patient said to the authors that she would not have spoken in the same way had she known she was speaking to the hospital doctor who had raised the alarm about the cluster of cases.

- by facilitating the session in a way that ensures everyone, including the famer(s) and expert-patient, has a chance to speak. In particular, special attention was given to ensuring that the farmer could speak first at each stage of the game, to counterbalance the implicit authority of veterinarians and healthcare professionals.

The effort made to address potential disparities clearly influenced the outcomes of the experiment. Emphasising this aspect is essential if the ZOOJEU 'dispositif' is to be broadcast and implemented in the absence of its creators.

The text above was included in S4 Workshop preparation and implementation in the section S4.4.

**Reviewer #2**: yes

**Results**
-Does the analysis presented match the analysis plan?
-Are the results clearly and completely presented?
-Are the figures (Tables, Images) of sufficient quality for clarity?

**Reviewer #1**: (No Response)

**Reviewer #2**: yes

**Conclusions**
-Are the conclusions supported by the data presented?
-Are the limitations of analysis clearly described?
-Do the authors discuss how these data can be helpful to advance our understanding of the topic under study?
-Is public health relevance addressed?

**Reviewer #1**: (No Response)

**Reviewer #2**: yes

**Editorial and Data Presentation Modifications?**

Use this section for editorial suggestions as well as relatively minor modifications of existing data that would enhance clarity. If the only modifications needed are minor and/or editorial, you may wish to recommend “Minor Revision” or “Accept”.

**Reviewer #1**: (No Response)

**Reviewer #2**: (No Response)

**Summary and General Comments**

Use this section to provide overall comments, discuss strengths/weaknesses of the study, novelty, significance, general execution and scholarship. You may also include additional comments for the author, including concerns about dual publication, research ethics, or publication ethics. If requesting major revision, please articulate the new experiments that are needed.

**Reviewer #1**:

The paper is now much better organized but remains extremely verbose, such that the main points are sometimes lost. Would be good to review and make more concise, bringing out the key aspects.
Thank you for this comment. The text was reduced and is now more concise. Details were transferred into supplementary material documents. A figure was also added to better bring out the ket aspects of the approach.

Can delete statements referring to format of the article (e.g. Lines 391-392)
Done

Please review and correct verb tense throughout (e.g. section 3.1.2).
Thank you for your feedback. We contacted the English-speaking professional scientific editor we consulted: she replied that the present tense is used in this section to describe how to play the game (‘the rules of the game’). She considers that the present tense is most appropriate for this.

Just to note that Q Fever is a WOAH listed disease, so much information on management as well as international guidelines, including on national reporting by all country governments, is available.

Thank you for this comment. The fact that Q fever is a WOAH listed disease was added in the introduction section (line 91). The discussion about Q fever epidemiology and management was reduced.

**Reviewer #2**:

I would like to thank the authors for carefully considering all of my comments. I also thank them for mentioning me in the article’s acknowledgments, though I do not believe this is necessary.

Thank you for your feed-back. We instead thanked both reviewers without mentioning their names.

English is not my native language, but it seems to me that certain terms and sentence structures should be revised; for example, I do not think the term “dispositive” can be used. I leave this comment to the editors' discretion.

- Thank you for your feedback. Our manuscript was reviewed by a professional scientific editor whose native language is English. We can provide a letter and/or request additional editing if there are terms and sentences structures that remain problematic.

- We agree that "dispositive" seems like an odd word. However, it is a specialized term that describes a concept in the information and communication sciences, which is why we decided to use it in our manuscript. Indeed, the definition that immediately follows the term's introduction comes from the book that we cite (ref. 45, Rittel *et al*. 1984, published in English).

PLOS authors have the option to publish the peer review history of their article ([what does this mean?](https://track.editorialmanager.com/CL0/https:%2F%2Fjournals.plos.org%2Fplosntds%2Fs%2Feditorial-and-peer-review-process%23loc-peer-review-history/1/010f019eee56d3b5-75a2ee07-876b-4901-a873-37486a3484ee-000000/GVLb6zwAbyWWLd50TvYqmR9EJSEXr9dJOO1KNLGMsKg=258)). If published, this will include your full peer review and any attached files.

If you choose “no”, your identity will remain anonymous but your review may still be made public.

**Do you want your identity to be public for this peer review?** For information about this choice, including consent withdrawal, please see our [Privacy Policy](https://track.editorialmanager.com/CL0/https:%2F%2Fwww.plos.org%2Fprivacy-policy/1/010f019eee56d3b5-75a2ee07-876b-4901-a873-37486a3484ee-000000/I6M2CWQ50df_dCYllSsLfKRxlNj5XPVtPsCsDdkS_t0=258).

Reviewer #1: **Yes:** Elizabeth Mumford

Reviewer #2: **Yes:** Marion Bordier

[NOTE: If reviewer comments were submitted as an attachment file, they will be attached to this email and accessible via the submission site. Please log into your account, locate the manuscript record, and check for the action link "View Attachments". If this link does not appear, there are no attachment files.]

**Figure resubmission:**

While revising your submission, we strongly recommend that you use PLOS’s NAAS tool (https://ngplosjournals.pagemajik.ai/artanalysis) to test your figure files. NAAS can convert your figure files to the TIFF file type and meet basic requirements (such as print size, resolution), or provide you with a report on issues that do not meet our requirements and that NAAS cannot fix.

After uploading your figures to PLOS’s NAAS tool - https://ngplosjournals.pagemajik.ai/artanalysis, NAAS will process the files provided and display the results in the "Uploaded Files" section of the page as the processing is complete. If the uploaded figures meet our requirements (or NAAS is able to fix the files to meet our requirements), the figure will be marked as "fixed" above. If NAAS is unable to fix the files, a red "failed" label will appear above. When NAAS has confirmed that the figure files meet our requirements, please download the file via the download option, and include these NAAS processed figure files when submitting your revised manuscript.

Done. The process failed for two files (Figures 4 and 6).

**Reproducibility:**

To enhance the reproducibility of your results, we recommend that authors of applicable studies deposit laboratory protocols in protocols.io, where a protocol can be assigned its own identifier (DOI) such that it can be cited independently in the future. Additionally, PLOS ONE offers an option to publish peer-reviewed clinical study protocols. Read more information on sharing protocols at https://plos.org/protocols?utm_medium=editorial-email&utm_source=authorletters&utm_campaign=protocols
