# Supplementary material for: ZOOJEU: Using Q fever to highlight a participatory science approach for fostering the co-design of management strategies for neglected zoonotic diseases
Source: PLoS Negl Trop Dis. 2026 Oct 15;20(10):e0014618. doi: 10.1371/journal.pntd.0014618 (PMC13671574; doi:10.1371/journal.pntd.0014618)
Supplement: Peer review history

# Peer review: Decision Letter 0

**DOI:** 10.1371/journal.pntd.0014618.r001

**Authors:** Stuart Blacksell

12 Sep 2025

ZOOJEU: a public policy design approach to facilitate the co-conception of management scenarios for neglected zoonotic diseases, based on the example of Q fever

PLOS Neglected Tropical Diseases

Dear Dr. Jourdain,

Thank you for submitting your manuscript to PLOS Neglected Tropical Diseases. After careful consideration, we feel that it has merit but does not fully meet PLOS Neglected Tropical Diseases’s publication criteria as it currently stands. Therefore, we invite you to submit a revised version of the manuscript that addresses the points raised during the review process.

Please submit your revised manuscript within 60 days Nov 11 2025 11:59PM. If you will need more time than this to complete your revisions, please reply to this message or contact the journal office at plosntds@plos.org. When you're ready to submit your revision, log on to https://www.editorialmanager.com/pntd/ and select the 'Submissions Needing Revision' folder to locate your manuscript file.

Please include the following items when submitting your revised manuscript:

* A rebuttal letter that responds to each point raised by the editor and reviewer(s). You should upload this letter as a separate file labeled 'Response to Reviewers'. This file does not need to include responses to any formatting updates and technical items listed in the 'Journal Requirements' section below.

* A marked-up copy of your manuscript that highlights changes made to the original version. You should upload this as a separate file labeled 'Revised Manuscript with Track Changes'.

* An unmarked version of your revised paper without tracked changes. You should upload this as a separate file labeled 'Manuscript'.

If you would like to make changes to your financial disclosure, competing interests statement, or data availability statement, please make these updates within the submission form at the time of resubmission. Guidelines for resubmitting your figure files are available below the reviewer comments at the end of this letter.

We look forward to receiving your revised manuscript.

Kind regards,

Stuart D. Blacksell

Section Editor

PLOS Neglected Tropical Diseases

Shaden Kamhawi

co-Editor-in-Chief

PLOS Neglected Tropical Diseases

orcid.org/0000-0003-4304-636XX

Paul Brindley

co-Editor-in-Chief

PLOS Neglected Tropical Diseases

orcid.org/0000-0003-1765-0002

**Journal Requirements:**

1) Please provide an Author Summary. This should appear in your manuscript between the Abstract (if applicable) and the Introduction, and should be 150-200 words long. The aim should be to make your findings accessible to a wide audience that includes both scientists and non-scientists. Sample summaries can be found on our website under Submission Guidelines:

https://journals.plos.org/plosntds/s/submission-guidelines#loc-parts-of-a-submission

2) Some material included in your submission may be copyrighted. According to PLOSu2019s copyright policy, authors who use figures or other material (e.g., graphics, clipart, maps) from another author or copyright holder must demonstrate or obtain permission to publish this material under the Creative Commons Attribution 4.0 International (CC BY 4.0) License used by PLOS journals. Please closely review the details of PLOSu2019s copyright requirements here: PLOS Licenses and Copyright. If you need to request permissions from a copyright holder, you may use PLOS's Copyright Content Permission form.

Please respond directly to this email and provide any known details concerning your material's license terms and permissions required for reuse, even if you have not yet obtained copyright permissions or are unsure of your material's copyright compatibility. Once you have responded and addressed all other outstanding technical requirements, you may resubmit your manuscript within Editorial Manager.

Potential Copyright Issues:

- Please confirm (a) that you are the photographer of Figure 3., or (b) provide written permission from the photographer to publish the photo(s) under our CC BY 4.0 license.

- Figures 1, 2A, 2B, 2C, and and 2D.. Please confirm whether you drew the images / clip-art within the figure panels by hand. If you did not draw the images, please provide (a) a link to the source of the images or icons and their license / terms of use; or (b) written permission from the copyright holder to publish the images or icons under our CC BY 4.0 license. Alternatively, you may replace the images with open source alternatives. See these open source resources you may use to replace images / clip-art:

- https://commons.wikimedia.org

- https://openclipart.org/.

**Reviewers' Comments:**

Reviewer's Responses to Questions

**Key Review Criteria Required for Acceptance?**

As you describe the new analyses required for acceptance, please consider the following:

**Methods:**

-Are the objectives of the study clearly articulated with a clear testable hypothesis stated?

-Is the study design appropriate to address the stated objectives?

-Is the population clearly described and appropriate for the hypothesis being tested?

-Is the sample size sufficient to ensure adequate power to address the hypothesis being tested?

-Were correct statistical analysis used to support conclusions?

-Are there concerns about ethical or regulatory requirements being met?

Reviewer #1: ‘Anthropological analysis’ as a method is vague. Describe exactly how analysis of the results of interviews and observations carried out and what qualitative methods were used.

Clarify how the citing of reference 25 relates to the current study methods and specify what was done in this study and what was previously done.

Explain by what process and by whom were the ‘anthropological’ results developed into the Worlds, whether they validated by any of the participants, and if so at what step, which participants and by what process

Specify the different groups of stakeholders involved in the anthropological part, who was involved, how many, how were they identified

More clearly contextualize and describe the worlds and clarify their relationship to each other e.g. do they represent co-existing realities? Separate ways of looking at the same reality?

Clarify whether ‘public policy design’ always include scenarios as implied or was this a choice, and clarify what parts of your description are established in PP design vs your own concepts of this design i.e. line 244, whether ‘this’ approach or ‘your’ approach

Include complete information on the nature of the device, who developed it, etc. Much of the generic/objectives/benefits text in step 2 should be in the introduction.

Reviewer #2: The methodology should be presented differently and should not include results. It should confine itself to defining the different steps followed, the conceptual and methodological frameworks in which they are rooted, explaining how the different steps relate to each other and what results are expected to meet the study's objective.

In addition, a certain amount of information and detail is lacking to enable the reader to fully understand the methodological approach followed.

The results are partially presented in the methodology section (anthropological analysis) and lack detail. It is difficult to understand whether Figure 1 is part of the material and method or of the results, as it has already been published in another study. In any case, it is presented somewhat disconnected from the text, and it's hard to understand what it represents and how it was used in this study. Similarly, we understand the rationale for using narrative graphs to communicate the results of an anthropological analysis to a wide audience; but in the context of a scientific article we'd like to know more about the raw data and methodology used to develop the messages. If this is presented elsewhere in another publication, then reference should be made to it and the narrative graphics should be presented in this study as materials used to facilitate the game (and not as results if this study per se).

In the interests of open science, raw data should be made available to the reader.

It seems that the words control, management, surveillance and prevention are used interchangeably when they do not mean the same thing. it would be desirable to always use the same words to refer to the same thing.

“its surveillance and control » ; « management and surveillance options » ; « the prevention and management of”, etc.

Authors need to better explain why participation in decision-making for disease surveillance and control is an issue specific to emerging and neglected zoonotic diseases. why doesn't this apply to diseases in general?

Lines 88-91: Reading this sentence, one gets the impression that the need for harmonization is necessary not only between sectors, but also between countries within the same sector; if this is the case, we would need to be more explicit on this point, emphasizing that the need for harmonization is not only between sectors, but also between geographical areas.

Lines 91-93: I'm a bit confused reading this sentence - I have the impression that there are two ideas here: on the one hand, the lack of comparability between data to get a robust overview of the international situation, and on the other, the difference in quality of surveillance data which gives a biased representation of the relative importance of the disease between countries.

Lines 119-123: This part of the text makes reference to recommendations for the management of Q fever in ruminant farms that are used to manage clusters of human cases occur. Can you clarify why recommendations dedicated to the livestock sector are used in case of human cases?

Then lines 129-130: if recommendations are used to deal with human cases, don’t we also need human health specialist around the table?

Lines 128-129: Can we have a reference here?

Lines 133-134: Putting the sentence this way gives the impression that the recommendations are a priori lacking in pragmatism and relevance; is it really what we mean? I rather expected that their pragmatism and relevance should be questioned with field actors, but not judged unsuitable in the first instance.

Line 136: “a perceived lack of knowledge”. Stated like this, it sounds like the lack of knowledge is perceived by farmers and not like public health authorities perceive a lack of knowledge among farmers

Lines 134-138: can we add a reference here?

Lines 138-142: expertise from farmers are not only about constraints - it's a bit reductionist and in contradiction to the rest of the paragraph, which describes the full extent of their expertise

Lines 167-170. In this introduction to the methodology, it would be interesting to specify how the two steps are articulated and their respective outputs to contribute to the overall objective of the study

Lines 171. what investigations are we talking about? just before, we're talking about investigation, but in the singular. is it the same thing? we need to be more precise.

Lines 171-173. when did this outbreak take place? how many farms, animals, human cases? please, briefly describe this outbreak.

Line 185. What do we mean by: “whose farms were in contact with the public”. A farm cannot be in contact with the public, do we want to men “open to the public”? If yes, can you explain why this is important in the context of the study and why this type of farm was targeted?

Lines 184-187. We'd like to know more about the study population.

What do we mean by animal health, and human health professionals? how many farms recruited, how many people recruited in each stakeholder category? Did all individual invited agree to take part of the study? what is the link between the farms and the participants? do all the participants interviewed have a link with the selected farms?

we'd also like to know how farms and participants were selected and have access to the interview guide (in supplementary file for example)

line 187. What do we mean by anthropological analysis? How did this lead to the identification of the worlds?

Line 193: who are these “others”? what type of delegation is it?

Lines 191-193: If “others” are veterinarians or vet labs, I do not see how it is possible for some farmers not to delegate health management to these stakeholders. Please better explain these two categories of farmers (high delegation versus low delegation).

Line 197: I don't feel that the figure legend correctly illustrates the content of the figure and correctly explains what it represents. For example, in world A, we talk about death, sensory experience; etc. how is this illustrated in the figure?

in addition, we miss information in this legend. What are the complex and challenging relationships? What do we mean by authentic and nurturing experiences? etc.

conversely, the figure is mentioning “evil spirits”; this is not explained in the figure

Why are there two different types of farmers represented? The ones who delegate health management and the ones who don’t?

Lines 210-212: the purpose of the narrative graphic needs to be better explained here; see previous comment about the need to summarize the methodology at the start of the methodology section to explain the different steps, how they relate to each other and how they contribute to the final objective of the study

Line 224: the complete graphic narrative should be available in a supplementary file

Line 226: “2a: Public action in response to disease outbreak” disease outbreak in general or Q fever outbreak specifically?

Line 228: do we really want to mean “poorly” here? Poorly generally has a negative connotation. It implies a lack of quality. I think here we rather want to say that the delegation is low and not of bad quality

Lines 236- 238: I'm surprised to read this sentence. For me, and also from what I understand from reading this article, the aim of these participatory approaches is not to transform research results into action, but to collectively define actions that integrate scientific and non-scientific expertise.

“The device was designed to solicit insight from all team members, leveraging their unique

perspectives and areas of expertise.”

**********

**Results**

-Does the analysis presented match the analysis plan?

-Are the results clearly and completely presented?

-Are the figures (Tables, Images) of sufficient quality for clarity?

Reviewer #1: The presentation of figures as headers is confusing – clarify and separate hypotheses, methods and results being presented and describe how and by whom they are used. Present the results and then reference the figures. Figures should include standard figure legends and dscribe other aspects related to the figures as part of the (background? Result?) text.

Clarify steps and results of the game – currently they are vague and generic. Present examples of the insights generated through the game.

Authors assume that the reader understands how the game is played. Include a step by step discussion of this so we can then understand what ‘The culmination of the game was the three groups’ independent selection of the "Territorial Council for Global Health" figurine’ means

Reviewer #2: They are some partial results about the anthropological analysis, but any results about the outputs of the game. For instance, what are the recommendations that arose from the discussions

See comments in the Methods section.

Lines 277-278: can we break down the stakeholder categories a little more by profession and sector of activity (public or private? for example, who are the 12 animal health professionals? veterinary officer? private vets? microbiologists in vet lab? What is a patient-expert? are the participants the same as those interviewed for the anthropological analysis?

Line 274: details of the different phases of the game and how they are facilitated are missing

For instance, how facilitators help participants in the formulation of proposals for enhanced

disease management strategies, based on the game outputs?

Lines 287-288: I do not understand the juxtaposition of these two sentences. What is the link between the news actors involved and new farms affected? They are examples of entities that were previously non-existent? Please clarify

Lines 288-289: what do we mean with the redistribution of entities? In time? In space? In terms of responsibilities?

Lines 294-295: I wonder if this sentence doesn't belong higher up in the paragraph, as it is an additional entity (if I am right)

Line 294: what is a Territorial Council for Global Health? What are its role and missions? Why was it introduced in the game?

**********

**Conclusions**

-Are the conclusions supported by the data presented?

-Are the limitations of analysis clearly described?

-Do the authors discuss how these data can be helpful to advance our understanding of the topic under study?

-Is public health relevance addressed?

Reviewer #1: In the last section, include any impacts of the convening of the game, including commitments to or actual changes in behaviour relative to public and animal health.

Include who evaluated the game for its future use in these settings and what was the outcomes, and by what means have these conclusions been reached?

Reviewer #2: Lines 300-301: can you provide examples of this realization?

Line 305: what is this shared concept of risk that participants agreed on?

Lines 305-308: I agree that strategies for disease management become more respectful of individual perspectives, but I'm not sure that taking into account the perspectives of different stakeholders necessarily makes strategies more effective, as other elements come into play. In any case, this deserves a reference

In addition, do you have evidence that participatory process was able to capture the perspectives of the different participants? Despite all efforts to encourage the various participants to raise their voice, it always remains power imbalance among some participants that will prevent some of them to express their opinions; moreover, the perspectives shared by the individuals present are not necessarily representative of those of the stakeholder category they represent.

All this deserves to be discussed as limits to the participatory process. There is a whole body of literature available on the subject

Lines 311-313: why the three groups’ independent selection of the "Territorial Council for Global Health" figurine was considered as the culmination of the game was? What were the tasks defined for this Council?

Line 316: what do we mean by “the issue of asymmetric positions due to legal and regulatory frameworks”?

Lines 328-330: why is the tool more specifically adapted to neglected and emerging diseases? And how its effectiveness has been evaluated?

Line 330: “This engagement is reinforced by facilitators”. I'd go even further and say that the quality of facilitation plays a crucial role in the success of this type of approach, particularly when there is a wide diversity of stakeholders, some of whom feel less legitimate than others to express themselves

Lines 334: is the term device appropriate?

**********

**Editorial and Data Presentation Modifications?**

Use this section for editorial suggestions as well as relatively minor modifications of existing data that would enhance clarity. If the only modifications needed are minor and/or editorial, you may wish to recommend “Minor Revision” or “Accept”.

Reviewer #1: Not sure if formatting of sections conforms to standard criteria

Some type edits needed e.g. Bullets in intro from line 40

Submit evidence of prior informed consent obtained from all participants, including the photograph

Reviewer #2: (No Response)

**********

**Summary and General Comments**

Use this section to provide overall comments, discuss strengths/weaknesses of the study, novelty, significance, general execution and scholarship. You may also include additional comments for the author, including concerns about dual publication, research ethics, or publication ethics. If requesting major revision, please articulate the new experiments that are needed.

Reviewer #1: The information itself is very interesting, and the rationale and outputs are important so worth trying to publish this. However, the presentation is chaotic and therefore difficult to understand exactly what was done. The background, methods and results should be presented more systematically including the stepwise processes for identification of participants, data collection, developing the figures, etc. Separate into sections for background, methods, etc . that could still be within the steps currently presented, or grouped across steps.

Reviewer #2: The study introduces ZOOJEU, a participatory public policy design tool in the form of a serious game aimed at improving the management of neglected zoonotic diseases, using Q fever as a case study. The approach involves two main steps: (1) an anthropological study to capture the diverse experiences and knowledge of stakeholders (especially farmers), and (2) the design and implementation of a game to simulate disease management scenarios. The authors argued that the game fostered collaboration between stakeholders, enabling them to jointly explore solutions tailored to the context and adapted to their perspectives. They also described ZOOJEU as a promising approach to promotes inclusive dialogue, mutual understanding, and health democracy, with potential applications beyond zoonoses.

Although the approach presented is compelling, the article lacks sufficient detail regarding the methodology used and the results obtained. Moreover, while the authors express strong enthusiasm about the game's potential to harness collective intelligence for developing effective, context-sensitive policy solutions, they provide no concrete examples of outputs and outcomes generated by the game, nor any evidence supporting its ability to achieve the intended goals—such as integrating diverse knowledge, fostering mutual understanding, building a shared vision, or designing effective and inclusive risk management strategies. Simply adopting a participatory approach does not guarantee relevant or effective results; it must be complemented by a rigorous evaluation of its implementation, outputs, and impacts.

I was also disturbed by the structure of the article adopted. The authors have chosen not to have specific sections dedicated to methodology, results and discussion, and as a result it is sometimes difficult to identify what is "materials and methods" or results specific to this study. The discussion is very limited, with no consideration of the potential biases associated with this type of approach, or of potential measures to improve the game.

As a non-native English speaker, I noticed what seem to be some Gallicisms that could lead to misinterpretations. I believe that further editing would enhance the clarity and readability of the text, as well as improve its overall quality. In addition, editing could help eliminate redundant sections and make the manuscript more concise.

The list of references should be reviewed; I've identified a few references that were incomplete (e.g., 44), with strange date format for the access date, or with small typos.

**********

PLOS authors have the option to publish the peer review history of their article (what does this mean?). If published, this will include your full peer review and any attached files.

If you choose “no”, your identity will remain anonymous but your review may still be made public.

**Do you want your identity to be public for this peer review?**  For information about this choice, including consent withdrawal, please see our Privacy Policy.

Reviewer #1: No

Reviewer #2: **Yes:**  Marion Bordier

[NOTE: If reviewer comments were submitted as an attachment file, they will be attached to this email and accessible via the submission site. Please log into your account, locate the manuscript record, and check for the action link "View Attachments". If this link does not appear, there are no attachment files.]

**Figure resubmission:**

**Reproducibility:**

To enhance the reproducibility of your results, we recommend that authors of applicable studies deposit laboratory protocols in protocols.io, where a protocol can be assigned its own identifier (DOI) such that it can be cited independently in the future. Additionally, PLOS ONE offers an option to publish peer-reviewed clinical study protocols. Read more information on sharing protocols at https://plos.org/protocols?utm_medium=editorial-email&utm_source=authorletters&utm_campaign=protocols

# Author comment: Author response to Decision Letter 1

**DOI:** 10.1371/journal.pntd.0014618.r002

27 Mar 2026

Attachment Submitted filename: Jourdain_Response to Reviewers 2026-03-13.docx

# Peer review: Decision Letter 1

**DOI:** 10.1371/journal.pntd.0014618.r003

**Authors:** Stuart Blacksell, Syed Abbas

22 Jun 2026

PNTD-D-25-00972R1

ZOOJEU: using Q fever to highlight a participatory science approach for fostering the co-design of management strategies for neglected zoonotic diseases

PLOS Neglected Tropical Diseases

Dear Dr. Jourdain,

Thank you for submitting your manuscript to PLOS Neglected Tropical Diseases. After careful consideration, we feel that it has merit but does not fully meet PLOS Neglected Tropical Diseases's publication criteria as it currently stands. Therefore, we invite you to submit a revised version of the manuscript that addresses the points raised during the review process.

Please submit your revised manuscript within by Jul 22 2026 11:59PM. If you will need more time than this to complete your revisions, please reply to this message or contact the journal office at plosntds@plos.org. When you're ready to submit your revision, log on to https://www.editorialmanager.com/pntd/ and select the 'Submissions Needing Revision' folder to locate your manuscript file.

Please include the following items when submitting your revised manuscript:

* A letter that responds to each point raised by the editor and reviewer(s). You should upload this letter as a separate file labeled 'Response to Reviewers'. This file does not need to include responses to any formatting updates and technical items listed in the 'Journal Requirements' section below.

* A marked-up copy of your manuscript that highlights changes made to the original version. You should upload this as a separate file labeled 'Revised Manuscript with Track Changes'.

* An unmarked version of your revised paper without tracked changes. You should upload this as a separate file labeled 'Manuscript'.

If you would like to make changes to your financial disclosure, competing interests statement, or data availability statement, please make these updates within the submission form at the time of resubmission. Guidelines for resubmitting your figure files are available below the reviewer comments at the end of this letter.

As the corresponding author, your ORCID iD is verified in the submission system and will appear in the published article. PLOS supports the use of ORCID, and we encourage all coauthors to register for an ORCID iD and use it as well. Please encourage your coauthors to verify their ORCID iD within the submission system before final acceptance, as unverified ORCID iDs will not appear in the published article. Only the individual author can complete the verification step; PLOS staff cannot verify ORCID iDs on behalf of authors.

We look forward to receiving your revised manuscript.

Kind regards,

Syed Shahid Abbas, MBBS, MPH, Ph.D.

Academic Editor

PLOS Neglected Tropical Diseases

Stuart Blacksell

Section Editor

PLOS Neglected Tropical Diseases

Shaden Kamhawi

co-Editor-in-Chief

PLOS Neglected Tropical Diseases

orcid.org/0000-0003-4304-636XX

Paul Brindley

co-Editor-in-Chief

PLOS Neglected Tropical Diseases

orcid.org/0000-0003-1765-0002

**Additional Editor Comments (if provided):**

Thank you for revising this paper and responding to reviewer comments. I think the paper is much stronger on the technical side. However, at almost 9,000 words, it is much longer than similar papers published in PLOS NTDS. While recognising that different writing styles can vary among disciplinary traditions, I think the paper does require substantial revision to make its findings accessible and relevant to the readership of PLOS NTDS.

Some ways the paper narrative could be made stronger are suggested below.

Introduction: Retain focus on transdisciplinary aspects and condense discussion on Q-fever epidemiology

Methods section: This is overtly descriptive. I suggest revising section into a concise narrative focused on reproducibility rather than completeness. Information on recruitment and workshop logistics, etc. could be moved to supporting information.

Results: You might consider removing step-by-step descriptions of the game to supplementary material and retain only findings linked to your central claims.

Discussion: Streamline section by eliminating repetition with the Introduction and Results. Move detailed descriptions of tools (comic, game mechanics, workshop organisation) to supporting information.

Supporting information: It is good practice to ensure all supplementary materials are explicitly signposted in the main text, as done effectively for S3.

Figures: If helpful, additional figures (e.g. flowcharts for methods and mind maps for analytic framework) could be used to streamline and advance narrative.

The authors should feel free to go beyond these suggestions or to consult a professional copy-editor to improve the overall clarity of their paper.

**Journal Requirements:**

If the reviewer comments include a recommendation to cite specific previously published works, please review and evaluate these publications to determine whether they are relevant and should be cited. There is no requirement to cite these works unless the editor has indicated otherwise.

1) Thank you for including an Ethics Statement for your study. Please include:

i) The approval number(s), or a statement that approval was granted by the named board(s)

ii) A statement that formal consent was obtained (must state whether verbal/written) OR the reason consent was not obtained (e.g. anonymity). NOTE: If child participants, the statement must declare that formal consent was obtained from the parent/guardian.].

**Reviewers' Comments:**

Reviewer's Responses to Questions

**Key Review Criteria Required for Acceptance?**

As you describe the new analyses required for acceptance, please consider the following:

**Methods**

-Are the objectives of the study clearly articulated with a clear testable hypothesis stated?

-Is the study design appropriate to address the stated objectives?

-Is the population clearly described and appropriate for the hypothesis being tested?

-Is the sample size sufficient to ensure adequate power to address the hypothesis being tested?

-Were correct statistical analysis used to support conclusions?

-Are there concerns about ethical or regulatory requirements being met?

Reviewer #1: Please clarify whether the case study in the game was based on the actual outbreak in 2017 and whether this was in the same location as the research, and if so how this might have impacted results

Can condense by deleting repetition of how the game is meant to be played and then how it was actually played.

Please expand more specifically on how potential disparities and power imbalances among players in groups were handled? Do you think these played a role in outcomes?

Reviewer #2: yes

**********

**Results**

-Does the analysis presented match the analysis plan?

-Are the results clearly and completely presented?

-Are the figures (Tables, Images) of sufficient quality for clarity?

Reviewer #1: (No Response)

Reviewer #2: yes

**********

**Conclusions**

-Are the conclusions supported by the data presented?

-Are the limitations of analysis clearly described?

-Do the authors discuss how these data can be helpful to advance our understanding of the topic under study?

-Is public health relevance addressed?

Reviewer #1: (No Response)

Reviewer #2: yes

**********

**Editorial and Data Presentation Modifications?**

Use this section for editorial suggestions as well as relatively minor modifications of existing data that would enhance clarity. If the only modifications needed are minor and/or editorial, you may wish to recommend “Minor Revision” or “Accept”.

Reviewer #1: (No Response)

Reviewer #2: (No Response)

**********

**Summary and General Comments**

Use this section to provide overall comments, discuss strengths/weaknesses of the study, novelty, significance, general execution and scholarship. You may also include additional comments for the author, including concerns about dual publication, research ethics, or publication ethics. If requesting major revision, please articulate the new experiments that are needed.

Reviewer #1: The paper is now much better organized but remains extremely verbose, such that the main points are sometimes lost. Would be good to review and make more concise, bringing out the key aspects.

Can delete statements referring to format of the article (e.g. Lines 391-392)

Please review and correct verb tense throughout (e.g. section 3.1.2).

Just to note that Q Fever is a WOAH listed disease, so much information on management as well as international guidelines, including on national reporting by all country governments, is available.

Reviewer #2: I would like to thank the authors for carefully considering all of my comments. I also thank them for mentioning me in the article’s acknowledgments, though I do not believe this is necessary.

English is not my native language, but it seems to me that certain terms and sentence structures should be revised; for example, I do not think the term “dispositive” can be used. I leave this comment to the editors' discretion.

**********

PLOS authors have the option to publish the peer review history of their article (what does this mean?). If published, this will include your full peer review and any attached files.

If you choose “no”, your identity will remain anonymous but your review may still be made public.

**Do you want your identity to be public for this peer review?**  For information about this choice, including consent withdrawal, please see our Privacy Policy.

Reviewer #1: **Yes:**  Elizabeth Mumford

Reviewer #2: **Yes:**  Marion Bordier

[NOTE: If reviewer comments were submitted as an attachment file, they will be attached to this email and accessible via the submission site. Please log into your account, locate the manuscript record, and check for the action link "View Attachments". If this link does not appear, there are no attachment files.]

**Figure resubmission:**

**Reproducibility:**

To enhance the reproducibility of your results, we recommend that authors of applicable studies deposit laboratory protocols in protocols.io, where a protocol can be assigned its own identifier (DOI) such that it can be cited independently in the future. Additionally, PLOS ONE offers an option to publish peer-reviewed clinical study protocols. Read more information on sharing protocols at https://plos.org/protocols?utm_medium=editorial-email&utm_source=authorletters&utm_campaign=protocols

# Author comment: Author response to Decision Letter 2

**DOI:** 10.1371/journal.pntd.0014618.r004

22 Jul 2026

Attachment Submitted filename: ZOOJEU_response-to-reviewers_2026-07-22-Submission.docx

# Editor report: Decision Letter 2

**DOI:** 10.1371/journal.pntd.0014618.r005

**Authors:** Richard Bowen, Stuart Blacksell

27 Jul 2026

Dear Dr. Jourdain,

We are pleased to inform you that your manuscript 'ZOOJEU: using Q fever to highlight a participatory science approach for fostering the co-design of management strategies for neglected zoonotic diseases' has been provisionally accepted for publication in PLOS Neglected Tropical Diseases.

Before your manuscript can be formally accepted you will need to complete some formatting changes, which you will receive in a follow up email. A member of our team will be in touch with a set of requests.

Please note that your manuscript will not be scheduled for publication until you have made the required changes, so a swift response is appreciated.

IMPORTANT: The editorial review process is now complete. PLOS will only permit corrections to spelling, formatting or significant scientific errors from this point onwards. Requests for major changes, or any which affect the scientific understanding of your work, will cause delays to the publication date of your manuscript.

Should you, your institution's press office or the journal office choose to press release your paper, you will automatically be opted out of early publication. We ask that you notify us now if you or your institution is planning to press release the article. All press must be co-ordinated with PLOS.

Thank you again for supporting Open Access publishing; we are looking forward to publishing your work in PLOS Neglected Tropical Diseases.

Best regards,

Richard A. Bowen, DVM PhD

Academic Editor

PLOS Neglected Tropical Diseases

Stuart Blacksell

Section Editor

PLOS Neglected Tropical Diseases

Shaden Kamhawi

co-Editor-in-Chief

PLOS Neglected Tropical Diseases

orcid.org/0000-0003-4304-636XX

Paul Brindley

co-Editor-in-Chief

PLOS Neglected Tropical Diseases

orcid.org/0000-0003-1765-0002

***********************************************************

Thank you for the second revision. I was not the original academic editor, but read the previous reviews and your responses and believe you have done quite a credible job of responding to reviewers, including reducing the length of the manuscript and moving some items to supplemental materials. I believe this is quite an interesting approach to education and that the manuscript is now acceptable.

# Editor report: Acceptance letter

**DOI:** 10.1371/journal.pntd.0014618.r006

**Authors:** Richard Bowen, Stuart Blacksell

Dear Dr. Jourdain,

We are delighted to inform you that your manuscript, "ZOOJEU: using Q fever to highlight a participatory science approach for fostering the co-design of management strategies for neglected zoonotic diseases," has been formally accepted for publication in PLOS Neglected Tropical Diseases.

We have now passed your article onto the PLOS Production Department who will complete the rest of the publication process. All authors will receive a confirmation email upon publication.

The corresponding author will soon be receiving a typeset proof for review, to ensure errors have not been introduced during production. Please review the PDF proof of your manuscript carefully, as this is the last chance to correct any scientific or type-setting errors. Please note that major changes, or those which affect the scientific understanding of the work, will likely cause delays to the publication date of your manuscript. Note: Proofs for Front Matter articles (Editorial, Viewpoint, Symposium, Review, etc...) are generated on a different schedule and may not be made available as quickly.

Soon after your final files are uploaded, the early version of your manuscript will be published online unless you opted out of this process. The date of the early version will be your article's publication date. The final article will be published to the same URL, and all versions of the paper will be accessible to readers.

For Research Articles, you will receive an invoice from PLOS for your publication fee after your manuscript has reached the completed accept phase. If you receive an email requesting payment before acceptance or for any other service, this may be a phishing scheme. Learn how to identify phishing emails and protect your accounts at https://explore.plos.org/phishing.

Thank you again for supporting open-access publishing; we are looking forward to publishing your work in PLOS Neglected Tropical Diseases.

Best regards,

Shaden Kamhawi

co-Editor-in-Chief

PLOS Neglected Tropical Diseases

Paul Brindley

co-Editor-in-Chief

PLOS Neglected Tropical Diseases
